# Post-Polymerization Modification of Polyethylene through Photochemical Oximation and Consecutive Ketonization

**DOI:** 10.1021/jacs.5c05212

**Published:** 2025-06-17

**Authors:** Maartje Otten, Inèz Klein Gebbink, Patrick J. Schara, Željko Tomović, Martin Lutz, Pieter C.A. Bruijnincx, Arnaud Thevenon

**Affiliations:** a Organic Chemistry & Catalysis, Institute for Sustainable and Circular Chemistry, Faculty of Science, 8125 Utrecht University, Universiteitsweg 99, 3584 CG Utrecht, The Netherlands; b Polymer Performance Materials, Department of Chemical Engineering and Chemistry, Technical University Eindhoven, 5600 MB Eindhoven, The Netherlands; c Structural Biochemistry, Bijvoet Centre for Biomolecular Research, Faculty of Science, 8125 Utrecht University, Universiteitsweg 99, 3584 CG Utrecht, The Netherlands

## Abstract

Post-polymerization modification of polyolefins provides access to functional polymers not readily available through bottom-up synthesis, thereby expanding the range of material properties available. Here, we demonstrate the clean and efficient functionalization of polyethylene through the solvent- and catalyst-free photochemical oximation, using *t*-butyl nitrite as an inexpensive and easy to handle NO radical source. Using various grades of polyethylene, we successfully incorporated oxime, ketone, and nitro groups on the polymer backbone, without radical cleavage or crosslinking. The functionalization degree of the three different functional groups is tuneable depending on the reaction atmosphere and system pressure, and the total functionalization degree can reach up to 2.9%. Detailed analysis of the post-modified polyethylene using ^15^N labeling revealed that the photochemical oximation preferentially functionalizes the pre-terminal carbon and that the functional groups are randomly spaced along the polymer backbone rather than adjacent. These results underscore the novelty and robustness of this methodology, enabling tailored polyolefins with properties that may expand their applications and potentially improve their recyclability.

## Graphical abstract

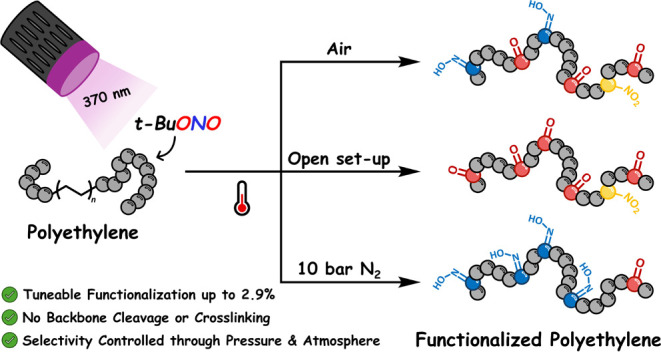

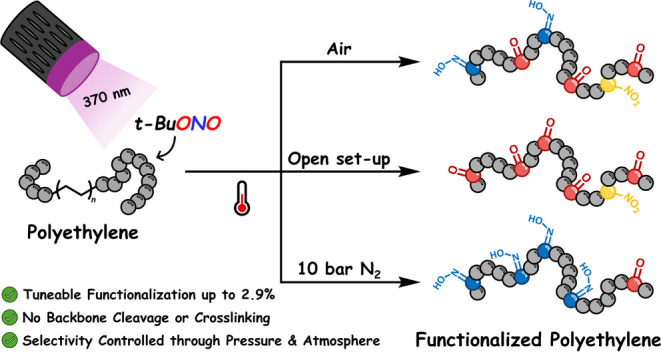

## Introduction

After more than a century of polymer science, lightweight, durable, and highly versatile synthetic polymers, produced on a multimillion-ton scale, have become essentially indispensable to our society and omnipresent in our daily lives.^1^ The predominant class of polymers that accounts for ∼50% of the global polymer production (and waste generation) is polyolefins such as low-density polyethylene (LDPE), high-density polyethylene (HDPE), and polypropylene (PP).^2,3^ Currently, these ubiquitous polyolefins are characterized by their high chemical resistance and nonpolarity due to the repeating nature of the carbon–carbon bonds in the backbone.^2,4^ Polar functional groups can be incorporated into this backbone via bottom-up syntheses, such as the catalytic copolymerization of ethylene and an α-functionalized olefin, but the range of polar groups remains limited due to catalyst poisoning despite continuous advances in method development.^2,5–8^ This results in a lack of structural diversity within the chemical space of readily accessible polyolefins. Alternative strategies to incorporate polar functional groups to tailor the polymer properties are, therefore, highly sought after. The degree of functionalization is a crucial parameter to control, as a high concentration of functional groups on the polymer backbone can compromise the desirable properties of the polyolefins.^9^ However, incorporating as little as 2% of polar functional groups can preserve the bulk properties while introducing new, beneficial functionalities.^10^

Post-polymerization modification (PPM) has emerged as a versatile late-stage strategy for synthetic polymer diversification.^11^ The concept itself was already introduced in 1922 by Staudinger with the pioneering research on hydrogenation of rubber and polystyrene (Scheme 1A).^12,13^ Soon after, the first PE example was reported by Osteraas and Olsen who demonstrated PE surface modification with carbenes and nitrenes.^14^ In the early 2000s, PPM re-emerged as a powerful strategy for polyolefin modification with the work of Hillmyer et al. on manganese porphyrin-catalyzed C–H bond oxidation as well as the work of Hillmyer and Hartwig on the Rh-catalyzed borylation of polyolefins.^15–18^ Spurred on by, among others, polymer circularity considerations, PPM has grown into a burgeoning field, which has enabled the introduction of various polar groups, such as alcohols, ketones, and halogens.^19–21^ Recent, notable examples are of the groups of Hartwig and de Vos who showed the oxidation of polyolefins using a ruthenium or a titanosilicate catalyst, respectively.^19,20^ The group of Hartwig subsequently demonstrated the iridium-catalyzed oxidation of the introduced hydroxyl groups into ketones (Scheme 1B).^19,22^ Both research groups oximated the introduced ketones thereafter using hydroxylamine (NH_2_OH), similar to the oximation of polyketone copolymers by the group of Nozaki.^23^ Oxime functionalization is attractive as a reactive handle, as they can be readily converted into other functional groups as for example, ketones, dinitro compounds, and amines, next to being a precursor for nitriles, nitrones, ureas, isoxazolines, and amides.^20,23,28,29^ The current state of the art for polyolefin oximation, however, still requires indirect methods that require several steps and makes use of expensive, precious metal-based catalysts.

**Scheme 1:**
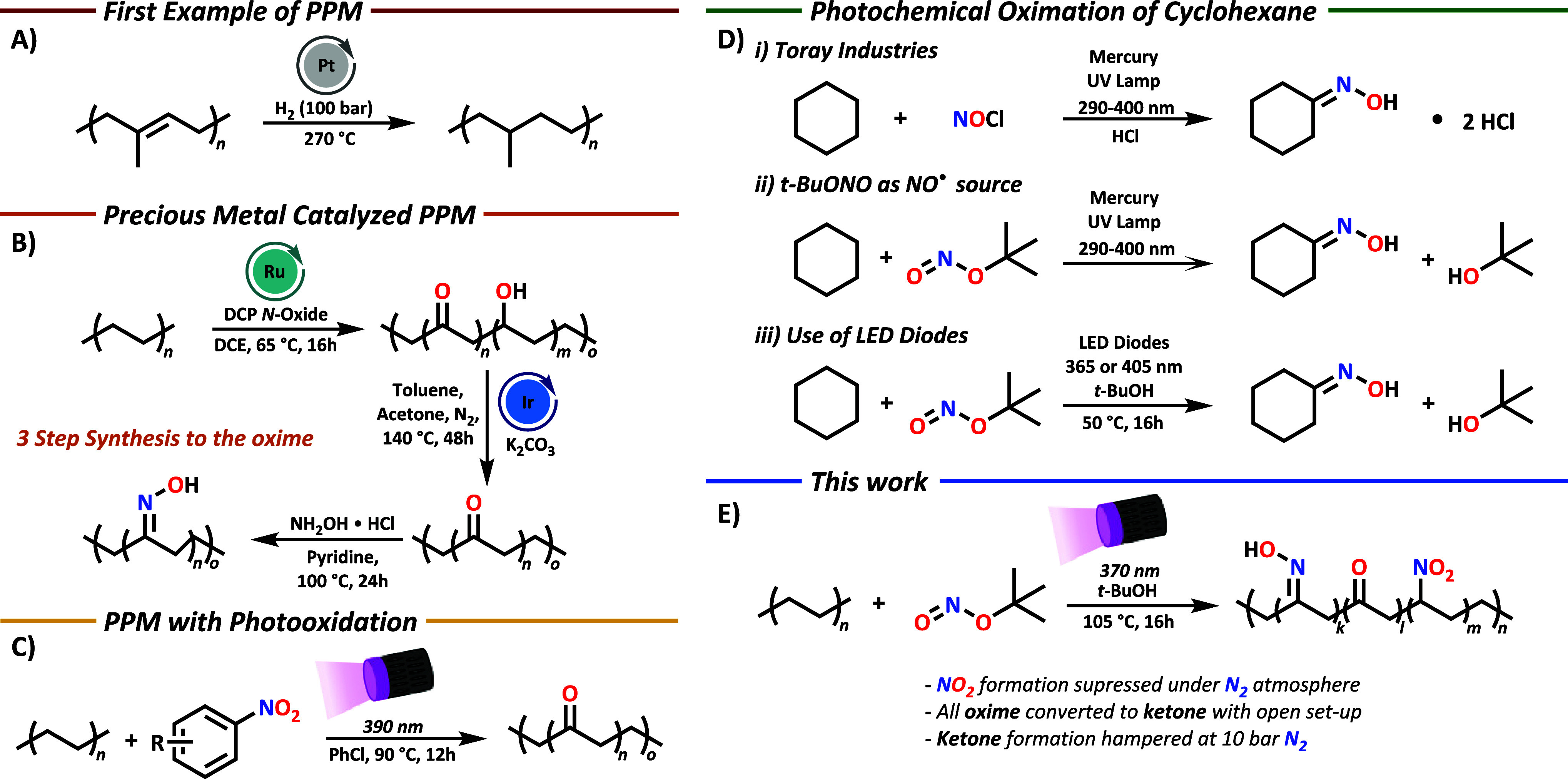
(A) Early Post-Polymerization Modification (PPM) Example of Rubber Hydrogenation,^12^ (B) Example of Precious Metal-Catalyzed PPM,^19^ (C) PPM by Photooxidation,^24^ (D) Photochemical Synthesis of Cyclohexanone Oxime Developed Originally by Toray Industries in 1963 (i)^25^ and Improvements Made by Mackor et al. Using *tert*-Butyl Nitrite as a NO Radical Source (ii)^26^ and a Light Source by Wysocki and Co-workers (iii),^27^ and (E) This Work on the Photochemical Oximation of PE

Robust methodologies that directly install chemically versatile functional groups in a straightforward and controlled manner, i.e., with no adventitious backbone cleavage or crosslinking, are highly desired. Metal-free strategies are particularly attractive to introduce functional groups on the backbone of polyolefins, but examples are limited. A prominent example reported by Leibfarth et al. involved amidyl radicals to introduce halogens and other electron-withdrawing functional groups on PE.^21^ The amidyl radical precursor could simply be activated by heating or, in some cases, by visible-light irradiation without the need of an additional initiator. The functional groups that can be installed with this methodology cover quite a large range of polar groups but do not include oxy-functional groups. A second notable example of metal-free PPM was reported by Miyake et al., in which PE materials are functionalized by photooxidation using nitrobenzene (Scheme 1C).^24^ With this approach, ketone functionalization degrees (up to 1.2%) can be achieved, albeit with some minor backbone cleavage.

Building on these developments, we aimed to develop a robust and efficient solvent-free photochemical approach to functionalize polyolefin backbones with polar moieties, targeting, in particular, the chemically versatile oxime. Here, early work by Toray Industries, who patented and commercialized a photochemical approach for the synthesis of cyclohexanone oxime from cyclohexane in 1963 (Scheme 1D­(i)), served as inspiration.^25^ Initially developed as a cost-effective alternative for ε-caprolactam synthesis, this process involved the utilization of a gas mixture comprising hydrogen chloride (HCl) and nitrosyl chloride (NOCl). Next to the use of corrosive HCl, the instability of NOCl and the use of a broadband UV Hg lamp posed significant operational challenges.^25^ A first improvement was demonstrated by Mackor et al., who used *t*-butyl nitrite (*t*-BuONO) as a more sustainable NO radical source (Scheme 1D­(ii)).^26^ The method thus avoids HCl generation and yields only *t*-butanol (*t*-BuOH) as a side product, which can be readily recycled back to *t*-BuONO (Scheme 2).^30^ Second, moving away from the mercury lamps to more sustainable and monochromatic high-power LED diodes, Wysocki and co-workers recently reported the photochemical synthesis of cyclohexanone oxime with *t*-BuONO using 365 or 405 nm LED diodes (Scheme 1D­(iii)).^27^ Addition of *t*-BuOH was found on top of that to prevent the formation of unwanted tarry byproducts, without interfering with the photochemical oximation itself when they followed the formation of the oxime during the reaction in detail (Scheme 2). While initially developed for direct oximation of cyclohexane, we hypothesized that this method can also be translated to linear hydrocarbons and be used to modify the backbone of polyolefins to yield new polymers; oxime functionalization is attractive as a reactive handle, as mentioned earlier. Analytically, oxime functional groups are convenient as they can be used as antennas to allow the regiochemistry of functionalization to be studied.

**Scheme 2:**
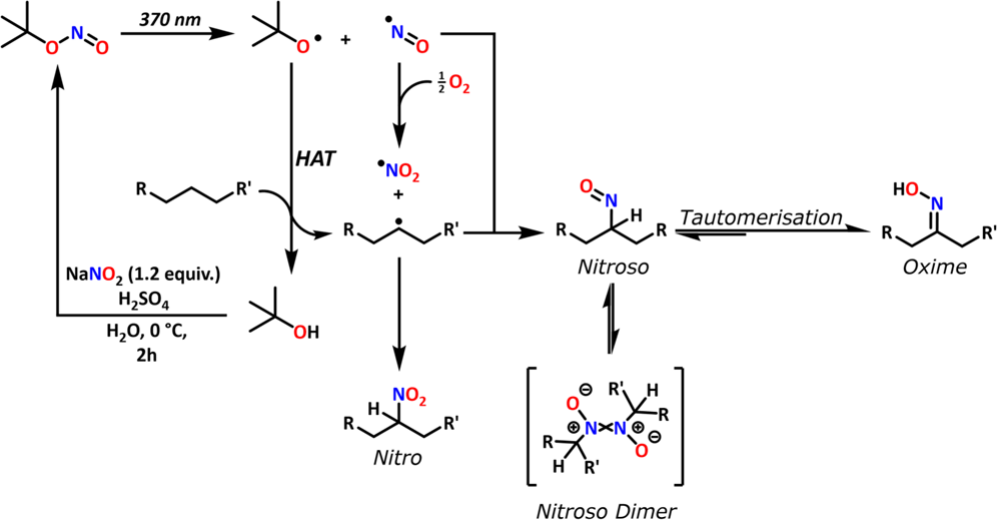
Reaction Pathway for the Photochemical Formation of the Oxime, the Effect of Oxygen on the NO Radical and Reaction to Resynthesize *t*-BuONO from the Formed *t*-BuOH after HAT.

Herein, we now report the first direct photochemical oximation of PE by photoexcitation of *t*-BuONO, in a readily scalable, solvent- and catalyst-free manner and show that polar groups, such as oximes, ketones, and/or nitro groups, can be introduced via a radical process. The chemoselectivity can be tuned depending on the used reaction conditions and without eliciting other changes on the polymer backbone, i.e., without undesired chain scission or crosslinking (Scheme 1E). Mechanistic insight provided by the oximation of small linear hydrocarbons with increasing backbone length and the use of ^15^N isotopically labeled *t*-BuO^15^NO for the oximation of PE provided insight into the chemo- and regioselectivity of the PE modification.

## Results and Discussion

To develop photochemical oximation as a PPM methodology for PE, we initially investigated the photochemical oximation of small linear alkane substrates of increasing backbone length as PE models. For this, we applied the best reaction conditions we found for the photochemical cyclohexane oximation, i.e., using a 370 nm LED light source at 50 °C in the presence of 0.1 equiv of *t*-BuONO and 0.8 equiv of *t*-BuOH (Table S2, entry 3), to *n*-undecane (Table 1, entry 1). An isolated yield of 84% for the *n*-undecanone oxime product was obtained, which is comparable with the previously reported yield for cyclohexanone oxime.^27^ Subsequently, we investigated the photochemical oximation of *n*-undecane at 105 °C to mimic reaction conditions where polyethylene would melt, keeping the other parameters the same (Table 1, entry 2). The reaction was monitored over time using ^1^H NMR spectroscopy, and the spectroscopic data show full consumption of *t*-BuONO after 2 h, together with the formation of the previously reported nitroso dimer species, which is readily identified by the resonance at 5.40 ppm.^27^ The nitroso dimer species is gradually converted over 16 h into the corresponding, thermodynamically more stable in-chain oxime, indicated by the resonances at 2.25 and 2.35 ppm for the α-protons, with no further change observed upon 22 h (Figure 1). Shorter irradiation times, i.e., 2 h of irradiation to allow full consumption of *t*-BuONO, followed by continuous heating for 14 h or pulsing the light source (cycling one min on, one min off), while keeping all other parameters constant (reaction time, temperature, and concentrations) result in lower oxime yields. In the absence of UVA light, only trace amounts of oxime are observed, likely due to the thermal decomposition of *t*-BuONO. This underscores the importance of continuous illumination for efficient product formation.

**Figure 1:**
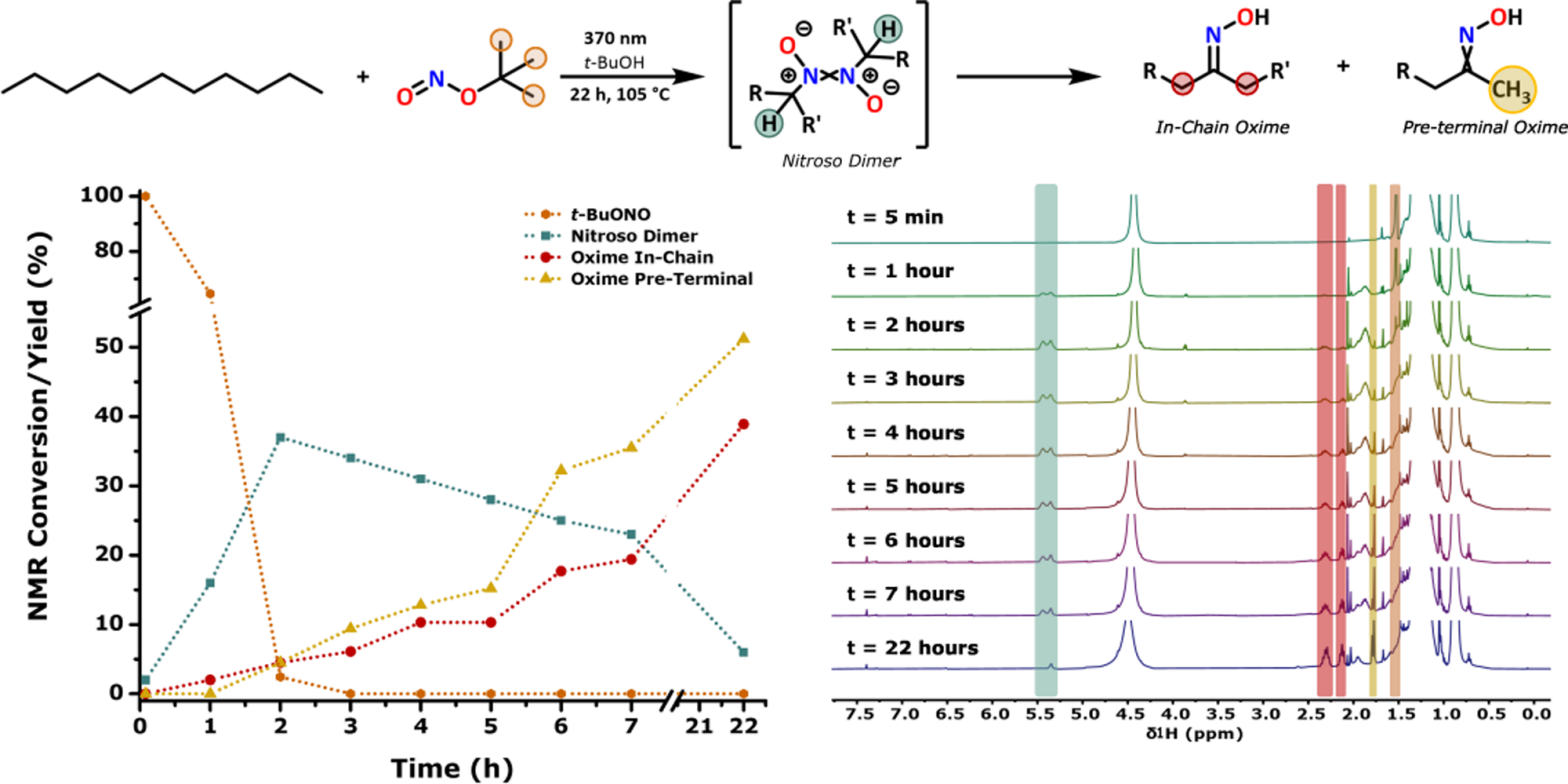
Photochemical oximation of small alkane model compounds and ^1^H NMR spectra of the reaction mixture measured over the course of 22 h.

**Table 1:**
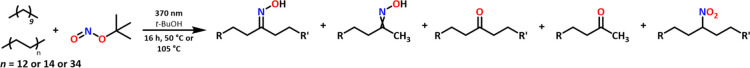
Investigation of the Photochemical Conditions for the Oximation of Linear Model Compounds.

entry	sample		temperature (°C)	isolated yield^a^ (%)	selectivity oxime:ketone:nitro	oxime selectivity (%)	regioselectivity^b^
1	*n*-undecane	(C_11_H_24_)	50	84	9:1:0	89	0.7:1
2	*n*-undecane	(C_11_H_24_)	105	82	8:1:0	88	0.7:1
3	*n*-tetradecane	(C_14_H_30_)	105	71	14:1:0	93	5.5:1
4	*n*-octadecane	(C_18_H_38_)	105	84	50:1:0	98	6.5:1
5	*n*-hexatriacontane	(C_36_H_74_)	105	60	1.8:2.3:1	36	1.5:1

a Isolated yield is defined as the percentage of functionalized material obtained after purification with respect to the amount of *t*-BuONO used.

b In-chain oxime:pre-terminal oxime functionalization

To further characterize the reaction products from the photochemical oximation, we developed an analytical toolbox to identify spectroscopic signatures, facilitating future characterization of PPM on PE, which is a recognized challenge.^31^ Next to ^1^H NMR, the typical analysis of a quaternary carbon with ^13^C NMR is difficult due to the lack of signal enhancement caused by the nuclear Overhauser effect (NOE) and peak broadening at the elevated temperature necessary for sample dissolution.^22,32,33^ ^1^H^13^C HSQC and HMBC experiments are therefore required to fully characterize the material but are often cumbersome at a low degree of functionalization (FG%, i.e., per 100 −CH_2_−). To overcome these challenges, we used the oxime functional group for ^1^H^15^N HMBC analysis, where we have the option to isotopically label the nitrogen of the installed oxime groups. Comparison of the ^1^H NMR spectrum of the products after the photochemical oximation reaction with various known oxime compounds already confirmed the formation of the in-chain oxime (with the characteristic resonances at 2.35 and 2.25 ppm) (Figure S10 vs Figures S56–S83). Additionally, the resonance at 1.85 ppm, which is part of the same spin system as the resonances overlapping at 2.35 and 2.25 ppm, demonstrates that the photochemical oximation reaction also occurs on the pre-terminal carbon atom (Figure S17). This was further supported by ^1^H^15^N HMBC, which shows that protons at 2.35, 2.25, and 1.85 ppm couple with two distinct oxime species at 332.9 and 334.3 ppm, consistent with in-chain and pre-terminal oximes (Figures S63, S71, and S77).^34^ In line with this, the ^1^H^13^C HMBC spectrum displays the expected coupling between the α-proton resonances of the in-chain oxime, with a quaternary carbon resonance between 163.0 and 162.0 ppm. Similarly, the α-proton resonances of the pre-terminal oxime show a cross peak with a quaternary carbon between 158.8 and 159.2 ppm. Furthermore, the absence of a cross peak at 367.1 ppm in ^1^H^15^N HMBC confirms the lack of oximes on adjacent carbons (Figure S85). Next to that, no resonances are observed in any of the NMR spectra, corresponding to functionalization on the terminal carbon. The low stability of a carbon-centered radical on this primary carbon in combination with the higher bond dissociation energy (BDE) of the C–H bond will make functionalization on this position on the backbone highly unlikely.^35^ Notably, an additional cross peak is observed at 211.9 ppm coupling with the multiplet at 2.45 ppm, indicating the presence of in-chain ketones, as confirmed by the ^1^H NMR spectrum of 6-undecanone (Figure S17). Although some spectral overlaps occur between the pre-terminal oxime, in-chain oxime, and ketone resonances in deuterated chloroform or 1,1,2,2-tetrachloroethane (required solvent for PE characterization), the spectra do allow for the regioselectivity of the reaction to be quantified: 59% of the oxime groups are on the pre-terminal carbon atom, and 41% are in-chain oxime groups. The observed regioselectivity, which is also typically observed in metal-catalyzed C–H bond oxidation reactions, is controlled by the BDE of the C–H bonds on the pre-terminal position compared to the in-chain C–H bonds.^35,36^ Additionally, the reaction shows high selectivity, with 88% for the oxime compared to 12% for the ketone (Table 1, entry 2).

*n*-Tetradecane and *n*-octadecane, with slightly longer carbon backbones, demonstrated comparable yields and selectivity toward the oxime formation. The regioselectivity is here lower toward the pre-terminal position as the result of statistically more available in-chain carbon atoms compared to pre-terminal carbon atoms (Table 1, entries 3 and 4). A slightly higher regioselectivity is observed again at the pre-terminal position when using the longest carbon backbone model, *n*-hexatriacontane (C_36_). Since this model compound results in a significantly higher viscosity of the reaction mixture as a result of the melting temperature (mp 74 °C), the chain folding in the melt of this model could result in a lower accessibility of the in-chain positions compared to the pre-terminal position and therefore a higher regioselectivity. The isolated yield and the oxime selectivity are for this model compound 60 and 36%, respectively, lower than those obtained with shorter aliphatic alkanes. For the shorter model compounds, isolated yields of 71–84% are obtained with oxime selectivities of 88–98%. On the longer backbone model compound (C_36_), we instead observe to our surprise a high functionalization with ketone functional groups (46% selectivity). Next to the ketone, a low amount of nitro groups is observed, showing a characteristic resonance at 4.47 ppm in the ^1^H NMR spectrum. This is further supported in the infrared spectrum, which shows the symmetric and asymmetric N–O vibrations at 1373 and 1553 cm^–1^, respectively (Figure S29). With the observed reactivity, we now have the opportunity to install three different polar functional groups on the backbone using a single method. This flexibility in functional groups will be investigated on the PE materials as it can enable finetuning of the polymeric properties in a controlled manner.

Having demonstrated the feasibility of photochemical oximation on the linear model compounds, we investigated photochemical oximation on various PE grades with increasing *M*_w_ and structural complexity. We started with PE that was synthesized in our laboratory using [Nd­(Cp*)_2_Cl_2_Li­(Et_2_O)_2_] as the polymerization catalyst (see SI Sections 2 and 3 for the synthesis and crystallographic data) in the presence of [Mg­(*n*-Bu)_2_] as a chain transfer agent to ensure the formation of linear low-molecular-weight HDPE with narrow dispersity (**PE-S**, *M*_w_ = 1.5 kDa and *Đ* = 1.28).^37^ We chose this sample as a well-defined model PE allowing us to identify any backbone cleavage or crosslinking events, which are generally observed in other PPM methodologies involving a free-radical mechanism.^11,24,33^ We also included commercially available HDPE samples (**HDPE-C**, *M*_w_ = 76.6 kDa and *Đ* = 11.15, and **HDPE-C** **High*M*_w_**, *M*_w_ = 114.2 kDa and *Đ* = 12.15) to further explore the applicability of the photochemical oximation method to more realistic pristine PE, as well as a second commercially available low molecular weight LDPE sample that already contained ketone functional groups on the backbone (**PE-C** **Low*M*_w_**, *M*_w_ = 4.2 kDa and *Đ* = 2.93). This, to simulate aged PE, typical for post-consumer material, FG_Ketone_% on this material was quantified with ^1^H NMR as 0.4%. This value is not taken into account in the determination of the selectivity and FG_Total_% after functionalization. Lastly, we used actual post-consumer LDPE (**PE-P**, *M*_w_ = 87.3 kDa and *Đ* = 7.15) to investigate the effect of additives and stabilizers on the photochemical oximation reaction. We tested the photochemical oximation on all these PE materials under similar conditions as used with the model compounds and performed them on different scales ranging from 0.5 g of PE to 10 g of PE used (0.1 equiv of *t*-BuONO and 0.8 equiv of *t*-BuOH, at 105 °C, except for HDPE-C High *M*_w_, which was functionalized at 125 °C, under a 370 nm LED light source, for 16 h). This resulted in the isolation of beige/pale yellow solids after the functionalization for all the PE materials in 95–99% recovered yields. With ^1^H NMR spectroscopy, we observe that the oximes are, similar to the small linear alkane products, installed on all the PE materials on both the in-chain and the pre-terminal position and not on adjacent carbon atoms, with a slight preference for the pre-terminal position (Figure 2A). In the ^1^H^13^C HMBC spectrum, we observe the coupling between the α-proton resonance of the in-chain ketone at 2.37 ppm with a quaternary carbon at 210.9 ppm. Similarly, the α-proton resonances of the pre-terminal ketone show a cross peak with a quaternary carbon atom at 208.4 ppm. Compared to the C11–C18 linear alkanes, the oxime to ketone functional group ratio is lower and more in line with the results seen for *n*-hexatriacontane, as also a small fraction of nitro groups is observed (Table 2). To improve the sensitivity of the ^1^H^15^N HMBC measurements, ^15^N isotopically labeled *t*-BuO^15^NO was synthesized and used during the photochemical oximation reaction on PE.^38^ Using *t*-BuO^15^NO enhances the signals of the expected ^1^H^15^N HMBC coupling for the oxime at both the in-chain as the pre-terminal carbon position; again, no coupling for adjacent oximes is observed. For **PE-S**, **PE-C** **Low*M*_w_**, and **HDPE-C**, we observe comparable FG_Oxime_ with 0.2, 0.5, and 0.3%, respectively. For **PE-C** **Low*M*_w_**, we, however, observe a slightly higher FG_Total_ with 2.1% compared to 1.2 and 1.7% for **PE-S** and **HDPE-C**, respectively. This indicates that the presence of the ketone on **PE-C** **Low*M*_w_** or the higher molecular weight for **HDPE-C** does not affect the photochemical oximation reaction. Interestingly, for **HDPE-C** **High*M*_w_** and **PE-P**, FG_Total_ are only 0.3 and 0.2%, respectively, but the selectivity toward the oxime is much higher (Table 2, entries 4 and 5). We speculate that these differences are due to the added stabilizers that are typically present in consumer plastics. Work is ongoing to further increase the FG% on post-consumer materials.

**Figure 2:**
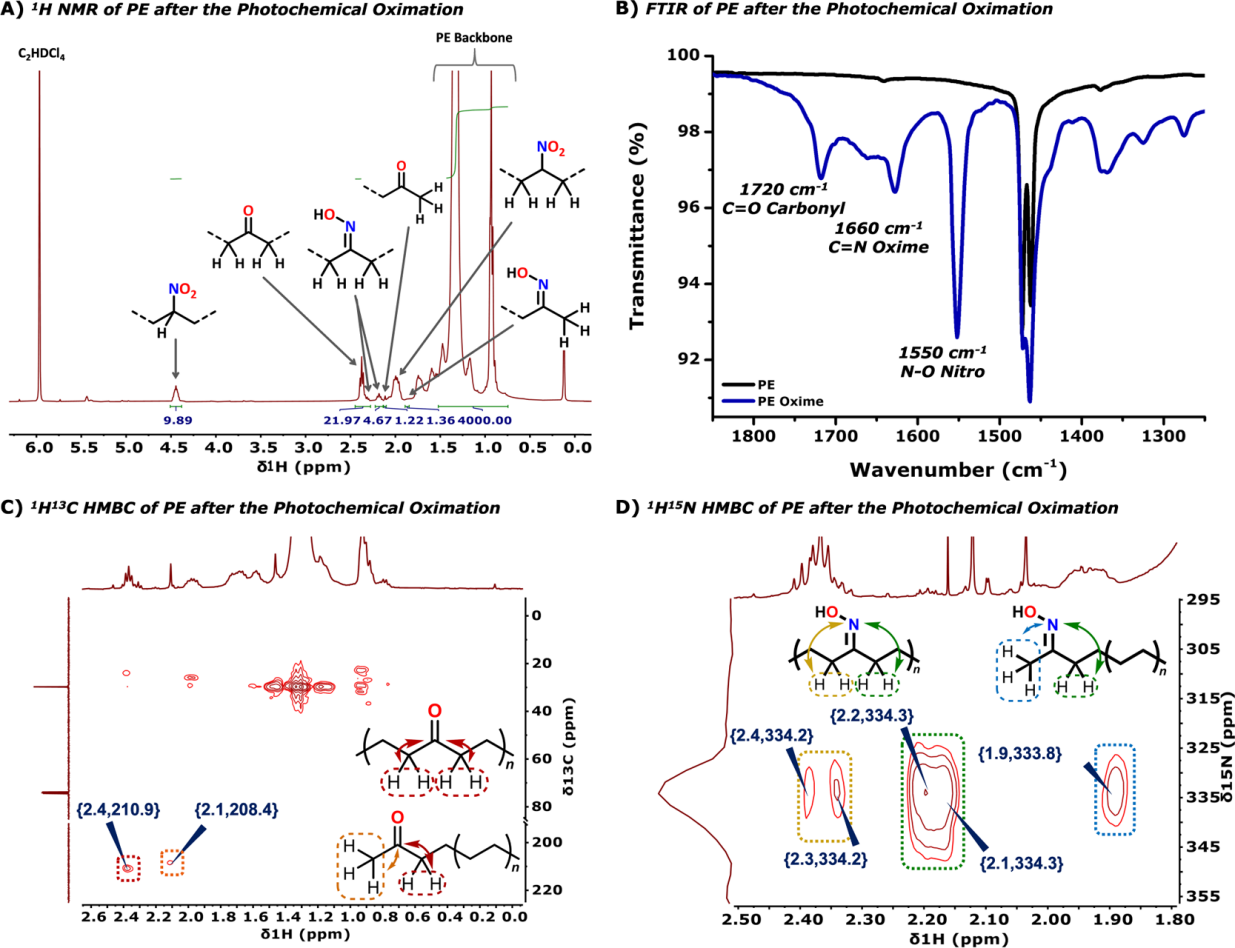
(A) ^1^H NMR spectrum of HDPE-C after the photochemical oximation reaction, measured in C_2_D_2_Cl_4_ at 120 °C. (B) FTIR spectrum of PE-C Low *M*_w_ before (black) and after (blue) the photochemical oximation reaction. (C) ^1^H^13^C HMBC spectrum of PE-S after the photochemical oximation reaction, measured in C_2_D_2_Cl_4_ at 120 °C (^3^ *J*-coupling with in-chain and pre-terminal ketone observed). (D) ^1^H^15^N HMBC spectrum of PE-C Low *M*_w_ after the photochemical oximation reaction, measured in C_2_D_2_Cl_4_ at 120 °C (^3^ *J*-coupling with in-chain and pre-terminal oxime observed).

**Table 2:**
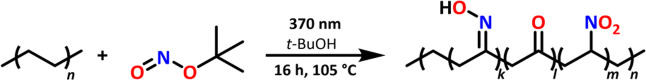
Investigation of Photochemical Conditions for Oximation on Polyethylene Materials

entry	sample	solvent	selectivity oxime:ketone:nitro	FG% oxime	FG% total	yield recovered material (%)
1	PE-S		2:9:1	0.2	1.2	99
2	HDPE-C		1:2:4	0.3	1.7	99
3	PE-C Low *M*_w_		3:10:1	0.5	2.1	98
4	HDPE-C High *M*_w_		3:1:2	0.2	0.3	99
5	PE-P		4:1:0	0.1	0.2	97
6^a^	PE-S		1:4:0	0.2	1.1	99
7^b^	PE-S		n.a.	n.a.	0.0	98
8^c^	PE-S		n.a.	n.a.	n.a.	98
9^d^	PE-S		n.a.	n.a.	n.a.	99
10^e^	PE-S		2:9:1	0.2	1.2	97
11	C_36_H_72_	1,1,2,2-tetrachloroethane	0:1:0	n.a.	trace amounts	95
12	C_36_H_72_	toluene	1.4:1:1.3	0.1	0.2	98
13^f^	PE-S		3:1:0	0.15	0.2	97
14^g^	PE-C Low *M*_w_		7:1:4	1.2	2.1	97
15^h^	PE-C Low *M*_w_		0:106:1	n.a.	2.9	98

a Reaction ran under a N_2_ atmosphere.

b No *t*-BuONO added.

c Di-*t*-BuOOBu added without *t*-BuOH and *t*-BuONO.

d *t*-BOOH added without *t*-BuOH and *t*-BuONO.

e *t*-BuOH and *t*-BuONO dried over 3 Å molsieves.

f Reaction ran under 10 bar N_2_.

g One-pot, two-step reaction in which the NH_2_OH·HCl and pyridine are added to the reaction vessel after 16 h of the photochemical oximation.

h Open reflux setup used for the reaction; n.a.: non applicable.

Rewardingly, gel permeation chromatography (GPC) analysis of all the photochemically modified PE materials and their corresponding parent PE materials shows no evidence of undesired radical backbone cleavage (nor crosslinking), which emphasizes that the system is well-behaved (Figure 3). There is also no indication for the formation of smaller alkane fractions up to 100 g/mol, nor were small alkanes observed in the collected supernatant after filtration of the PE obtained after the reaction. Taken together, these results show that the photochemical method developed is one of the first approaches where oximes, ketones, and nitro groups can be directly installed on different PE types via a radical pathway without backbone cleavage or crosslinking.

**Figure 3:**
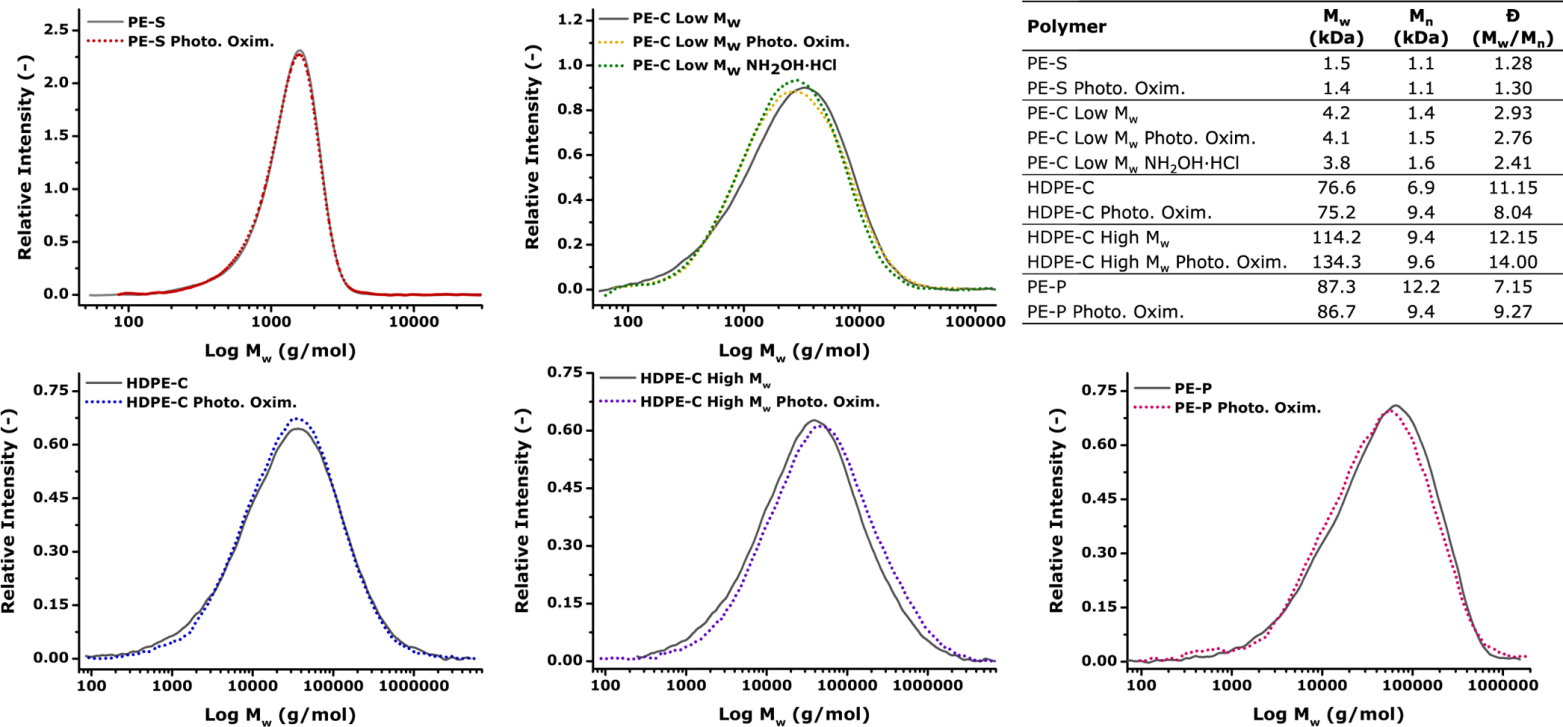
Gel permeation chromatography (GPC) traces of PE-S, PE-C Low *M*_w_, HDPE-C, HDPE-C High *M*_w_, and PE-P before and after the photochemical oximation (Photo. Oxim.), with the corresponding *M*_w_, *M*_n_ in kDa, and *Đ*.

Subsequently, we varied the reaction conditions to get a better understanding of the formation of the ketone and the nitro functional groups on the polymer backbone. First, the reaction with a degassed reaction mixture run under a strict N_2_ atmosphere showed that the formation of the nitro groups can be completely suppressed (Table 2, entry 6). This is in line with work on the nitration of olefins, where the formation of NO_2_^•^ is caused by the reaction of the NO^•^ from (*t*-BuONO) with half an equivalent of O_2_ (Scheme S1). This NO_2_^•^ can subsequently recombine with a carbon-centered radical on the polymer backbone to form the nitro functionality.^39^ To study the formation of the ketone, we first ran the reaction for only 2 h and already observed ketone formation next to the oxime, both in low yields, indicating that this reaction happens already at an early stage. Subsequently, we investigated the effect of reaction temperature, where we observed no differences in yield or selectivity between 105 and 125 °C. Hereafter, we explored the potential formation of peroxides by homocoupling of the alkoxy radicals from *t*-BuOH during the reaction to *t*-BuOOBu.^40^ We therefore tested the photochemical oximation reaction only in the presence of *t*-BuOH (0.8 equiv) under aerobic conditions, keeping all other reaction parameters the same (Table 2, entry 7). However, we observed no ketone formation or any other functionalization of the polymer backbone. The same result was obtained using *t*-BuOOBu directly or using *t*-BuOOH, under similar conditions (Table 2, entries 8 and 9). As strong inorganic acids are typically required to hydrolyze oxime moieties to ketone, hydrolysis caused by adventitious water is unlikely. To firmly exclude this though, we explored the photochemical reaction with dried *t*-BuONO and *t*-BuOH; the product obtained after the reaction with the dried reagents still contained ketone functional groups, indicating that hydrolysis is not causing the formation of the ketone (Table 2, entry 10).

This made us wonder if the formation of the ketone was indirectly caused by *t*-BuONO. To investigate this hypothesis, we subjected two of the model oximes, 6-undecanone oxime and 3-pentanone oxime, to photochemical reaction conditions. Upon opening the reaction vessel after the reaction, a significant amount of gaseous products was released, similar to what we observed for the reactions with PE. During analysis of the reaction mixture, we observed that the oxime is fully converted into the ketone as a major product and additionally a new multiplet at 1.70 ppm and two triplets part of the same spin system at 0.75 and 0.61 ppm are observed. The COSY spectrum indicates that this is a species separate from the ketone, and ^1^H^15^N HMBC and ^1^H^13^C HMBC show that the multiplet at 1.7 ppm couples with a nitrogen resonance at 363.0 ppm and a quaternary carbon at 180.3 ppm (Figures S118–S122). We assigned these peaks to a nitrimine product in line with studies on similar types of reactions.^40–42^ The product ratio between the ketone and nitrimine is 2.4:1 after 16 h (Figure 4B). To investigate if the conversion of the oxime to the ketone is more influenced by UVA light, temperature, or *t*-BuONO, we conducted multiple parallel reactions on the 6-undecanone oxime. In the first reactions, we tested its reactivity under irradiation at 370 nm, at 25 °C, or at 105 °C, in the presence of 1.0 equiv of *t*-BuONO and 0.8 equiv of *t*-BuOH (Figures S123–S126). In the second set of reactions, we tested the same reactivity at 25 and 105 °C without the light source. The results show that irradiation significantly accelerates the conversion to the ketone compared to elevated temperature (22 h vs 30 min). In the last set, we repeated both reactions in the absence of *t*-BuONO and *t*-BuOH. Under these conditions, only 10% of the oxime was converted to the ketone after 15 h in both cases (Figure S127).

**Figure 4:**
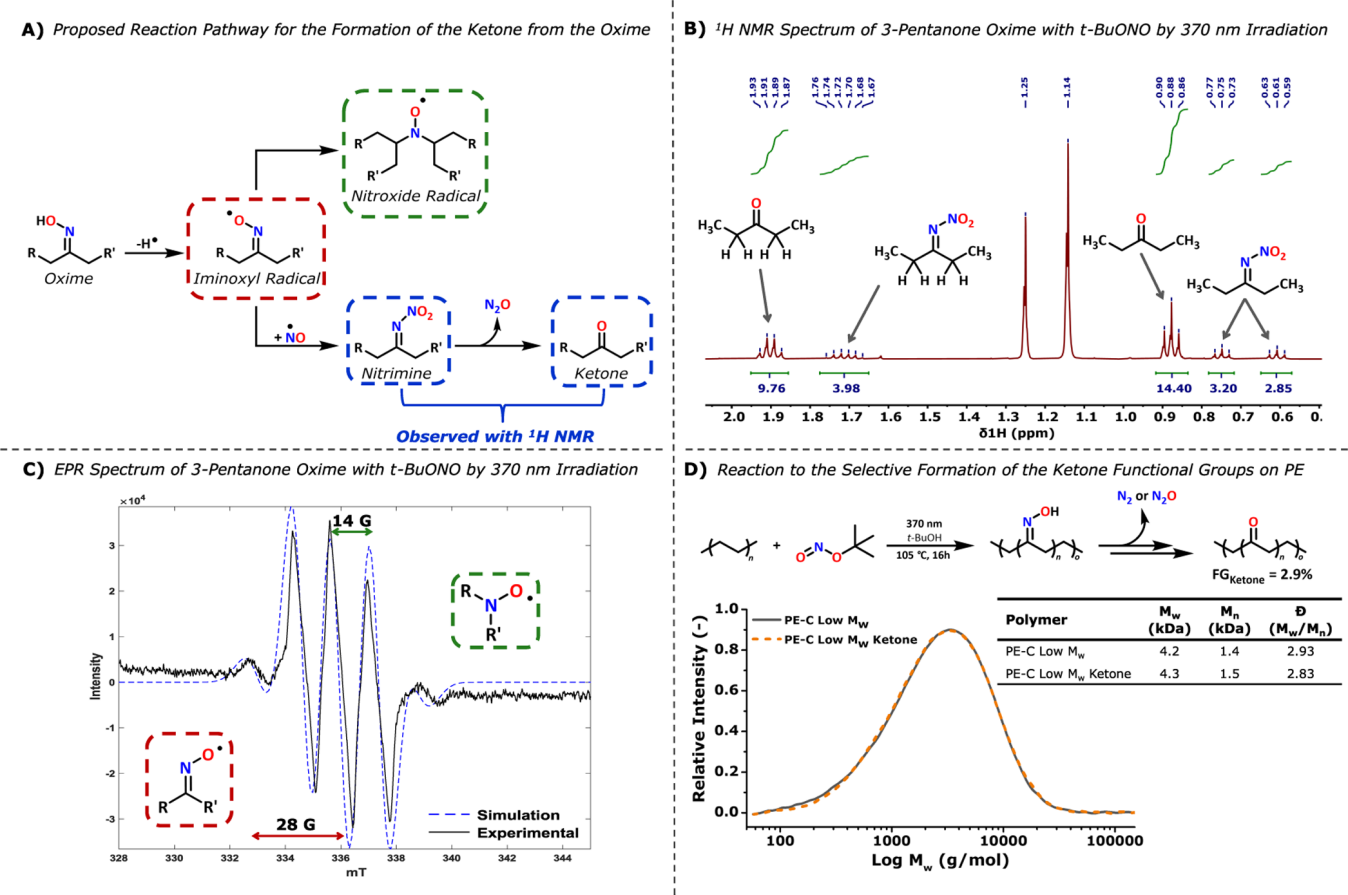
(A) Proposed reaction mechanism for the formation of the ketone from the parent oxime on the polymer backbone based on the observed nitrimine and ketone in NMR in combination with the work reported by Ingold.^42^ (B) ^1^H NMR spectrum of 3-pentanone oxime with *t*-BuONO and *t*-BuOH measured after 1 day in C_6_D_6_ at 25 °C; (C) EPR spectrum of 3-pentanone oxime with *t*-BuONO in which the ^14^N hyperfine coupling of 14 G for the nitroxide radical and 28 G for the iminoxyl radical is observed. (D) Ketone formation from the oxime pushed to completion using an open reaction vessel so the formed gases can escape and yields FG_Ketone_ of 2.9% on the polymer backbone, with the corresponding GPC trace.

Having confirmed that *t*-BuONO and UVA light are involved in ketone formation from the oxime, we looked into the possible reaction pathway for this reaction using 6-undecanone oxime, 2-butanone oxime, 3-pentanone oxime, and cyclohexanone oxime as model compounds to mimic the in-chain and pre-terminal oximes on the polymer backbone, as well as study the effect of their different carbon backbones lengths. Upon mixing any of these oximes with 1.0 equiv of *t*-BuONO, either neat or in solvents such as benzene and chloroform, and irradiating the samples at 370 nm at 25 °C, we observed a rapid color change from yellow (from *t*-BuONO) to blue accompanied by the release of gaseous products (Figure S111). This blue color is also, but to a lesser extent, observed during the photochemical reaction of PE. Furthermore, upon opening the reaction vessel after the photochemical oximation reactions with PE, we always observed the release of gaseous products, suggesting that a similar chemistry is occurring. Monitoring the reaction of 3-pentanone oxime with ^1^H NMR spectroscopy revealed that directly after the formation of the blue color and release of the gaseous products, a significant amount of the oximes had already been converted into the ketone and a smaller amount of nitrimine. This is similar to what we observed prior when 6-undecanone oxime was subjected to the photochemical reaction conditions. Aliphatic nitrimines are known to decompose further to the ketone, accompanied by the release of N_2_O gas. This explains why the nitrimine was not detected before on the polymer backbone as it is too instable to isolate.^43^

While this decomposition pathway accounts for ketone formation and gaseous product release during the photochemical oximation reaction, it does not explain the observed blue color. In the literature, the conversion of an oxime to a ketone has been reported to follow a pathway during which an iminoxyl radical intermediate species is formed and these iminoxyl radials are typically highly blue-colored (Figure 4A).^40,41,44,45^ We therefore investigated if, in the blue-colored solutions, low concentrations of organic radicals are present using electron paramagnetic resonance (EPR) spectroscopy. The EPR spectra of the blue-colored reaction mixture for 6-undecanone oxime and 3-pentanone oxime with 1.0 equiv of *t*-BuONO show two overlapping species with ^14^N hyperfine coupling of 16 and 32 G and 14 and 28 G, respectively (Figure 4C and Figures S114 and S115). The ^14^N hyperfine coupling of ∼28–32 G is in line with values reported for the iminoxyl radical, and the ^14^N hyperfine coupling of ∼14–16 G is in line with values reported for nitroxide-type radicals.^40–42,44–46^ The EPR spectra of cyclohexanone oxime and 2-butanone oxime with 1.0 equiv of *t*-BuONO show only one species with a ^14^N hyperfine coupling of ∼14–16 G, characteristic of the same nitroxide-type radicals (Figures S112 and S113). For sterically unhindered iminoxyl radicals, such as 2-butanone iminoxyl, the rate of decay of this radical is reported to be 400 s^–1^, which makes detection cumbersome.^41^ The iminoxyl radical is known to decay into a nitroxide-type radical by recombination with a carbon-based radical, which is much more stable and well-detectable with EPR spectroscopy.^40–42^ We therefore postulate that the observed nitroxide-type radical for cyclohexanone oxime and 2-butanone oxime is the result of fast decay of the iminoxyl radical species. We believe that the relatively low concentration of free radicals during the photochemical oximation reaction of PE makes the formation of the nitroxide radical on the polymer backbone highly unlikely. If nitroxide radicals would be formed during the reaction, then crosslinking of the PE material should take place and this is not observed with GPC. Additionally, the lack of backbone cleavage or crosslinking can be explained by the high rate of recombination of the carbon-centered radicals with NO radicals, which is expected to occur with rate constants close to the diffusion-controlled rate limit, i.e., 10^9^–10^10^ M^–1^ s^–1^.^47^

Based on all the obtained EPR data of the reaction between the model oxime compounds and *t*-BuONO, we propose that the formation of the ketone is the result of the formation of an iminoxyl radical species that is too reactive under the photochemical oximation reaction conditions and decomposes to the ketone, similar to what is observed in the literature.^40–42,44–46^ The formation of the iminoxyl radical during the photochemical oximation reaction is most likely the result of hydrogen atom transfer (HAT) by a high local alkoxy radical or NO^•^ concentrations near the oxime. The decomposition to the ketone requires a reaction pathway in which N_2_ and N_2_O are released, which is in line with our observations (Figure 4A).

With these mechanistic insights in hand, we tuned the reaction conditions to stir the selectivity toward either the oxime product or the ketone. The difference in oxime selectivity between the small linear alkanes, **PE-P**, and the other PE materials suggested that lower concentrations of free radicals in the reaction mixture could decrease the formation of the iminoxyl radical and therefore the formation of the ketone. Since the BDE of the O–H bond in the oxime (80–85 kcal/mol) is significantly lower than the BDE of the C–H bonds in the polymer backbone (e.g., 98.0 kcal/mol for heptane), the high local concentrations of the remaining alkoxy radical of *t*-BuONO could lead to the formation of the iminoxyl radical.^35,42^ For the small linear alkanes, an excess of the substrate was used, which resulted in a diluted reaction mixture, and to test this dissolution as well for the PE materials, we used toluene or 1,1,2,2-tetrachloroethane (C_2_H_2_Cl_4_) as a reaction solvent (Table 2, entries 11 and 12). While C_2_H_2_Cl_4_ shows no effect, toluene improved the selectivity, albeit with a decrease in the degree of functionalization, as we believe that toluene might be affected too by the reaction (BDE of 88.5 kcal/mol). Thereafter, we investigated a slow addition of *t*-BuONO, in which *t*-BuONO is added portionwise every 4 h to the neat reaction mixture. This approach, however, lowered the overpressure of the released N_2_ and N_2_O gases and drives the reaction toward the ketone. Next to that, the reaction becomes more exposed to air resulting in an increased loading of the nitro functional groups.

We suspected that performing the photochemical oximation under high pressure of N_2_ gas and possibly accompanied by slow addition of *t*-BuONO would significantly improve the selectivity for the formation of the oxime. Using a custom-made autoclave with UV light transmitting windows, we tested the photochemical oximation of **PE-S** at pressures of 1, 5, and 10 bar N_2_ gas (Figure S47). After the reaction with 1 or 5 bar N_2_, we do not observe any change in selectivity, but at 10 bar N_2_, we indeed observe the selectivity to be in favor of the oxime here with an FG_Oxime_ to FG_Ketone_ ratio being 3:1 and no formation of the nitro functional group (Table 2, entry 13). It should be noted that the design of the pressurized reaction vessel required the use of more *t*-BuOH, which dilutes the reaction mixture. Work is ongoing to further investigate this route for higher oxime functionalization, as the pressurized reaction required the use of more *t*-BuOH, which diluted the reaction mixture. Alternatively, a higher FG_Oxime_% can be achieved on the PE backbone by a one-pot, two-step approach. This involves oximation of the ketone functional groups by treating the post-polymerization modified PE with hydroxylamine hydrochloride (NH_2_OH·HCl) in pyridine, enabling an FG_Oxime_ of 1.2% (Table 2, entry 14).^19,23^

Hereafter, we explored whether the selectivity of the photochemical oximation reaction could be shifted solely toward the ketone. As N_2_ and N_2_O are released upon conversion of the oxime to the ketone, performing the reaction in an open reflux setup, rather than in a closed vessel, should allow the escape of these gases, which should favor the formation of the ketone (Figure 4D). Indeed, a ratio of 0:106:1 (oxime:ketone:nitro) was achieved with an impressive FG_Ketone_ of 2.9% (Table 2, entry 15). Notably, such high FG_Ketone_% levels have until now only been achieved with precious metal catalysts that required two reactions steps to selectively obtain ketone functional groups on the backbone or with titanosilicate-catalyzed oxidation.^19,20^ Moreover, the GPC traces of the fully ketone functionalized PE and the one-pot, two-step oxime functionalized PE both display that also under these conditions, no undesired radical backbone cleavage (or crosslinking) is observed, which makes this robust approach one of the first tuneable functionalization reactions for PE via a radical pathway (Figure 3 and Figure 4D).

Now that we have functionalized all PE materials and have determined the functional groups introduced on the polymer backbone, we looked into the thermal and physical properties of our post-polymerization modified PE (Figure 5). As previously observed in the literature, there is an inverse relationship between the degree of functionalization and the melting temperature (*T*_m_).^33^ As expected, the **PE-P** material after the photochemical oximation reaction presents a comparable *T*_m_ (0.3 °C increase) relative to the parent PE material attributed to the low functionalization degree (FG_Total_ = 0.2%). Surprisingly, **HDPE-C** **High*M*_w_** displays with an FG_Total_ = 0.3% a minor increase in *T*_m_ with 1 °C. **PE-S** and **PE-C** **Low*M*_w_** both display a decrease in *T*_m_ of 8 °C after photochemical oximation, while **HDPE-C** displays a decrease in *T*_m_ of 3 °C, which is most likely due to the nitro groups present on this polymer sample. The values for PE-S and PE-C **Low*M***_w_ are in line with previous reports on PE-oxime and PE-ketone materials with similar FG% where typically decreases of 3 and 12 °C are observed for PE-ketone and PE-oxime materials, respectively.^19,23^ When the oxime FG% is increased for **PE-C** **Low*M*_w_** after the reaction with NH_2_OH·HCl, the *T*_m_ remains comparable, but when the polymer is only functionalized with ketone groups, an almost identical *T*_m_ is observed compared to the parent PE. This is in line with the effects of ketone and oxime functionalization, where for more oxime functionalized PE, a larger decrease in *T*_m_ is typically observed.^19,23,33^ Fully nitro functionalized PE materials are not reported in the literature, and the effect of the nitro groups on the *T*_m_ of the photochemically functionalized PE can therefore not be fully determined.

**Figure 5:**
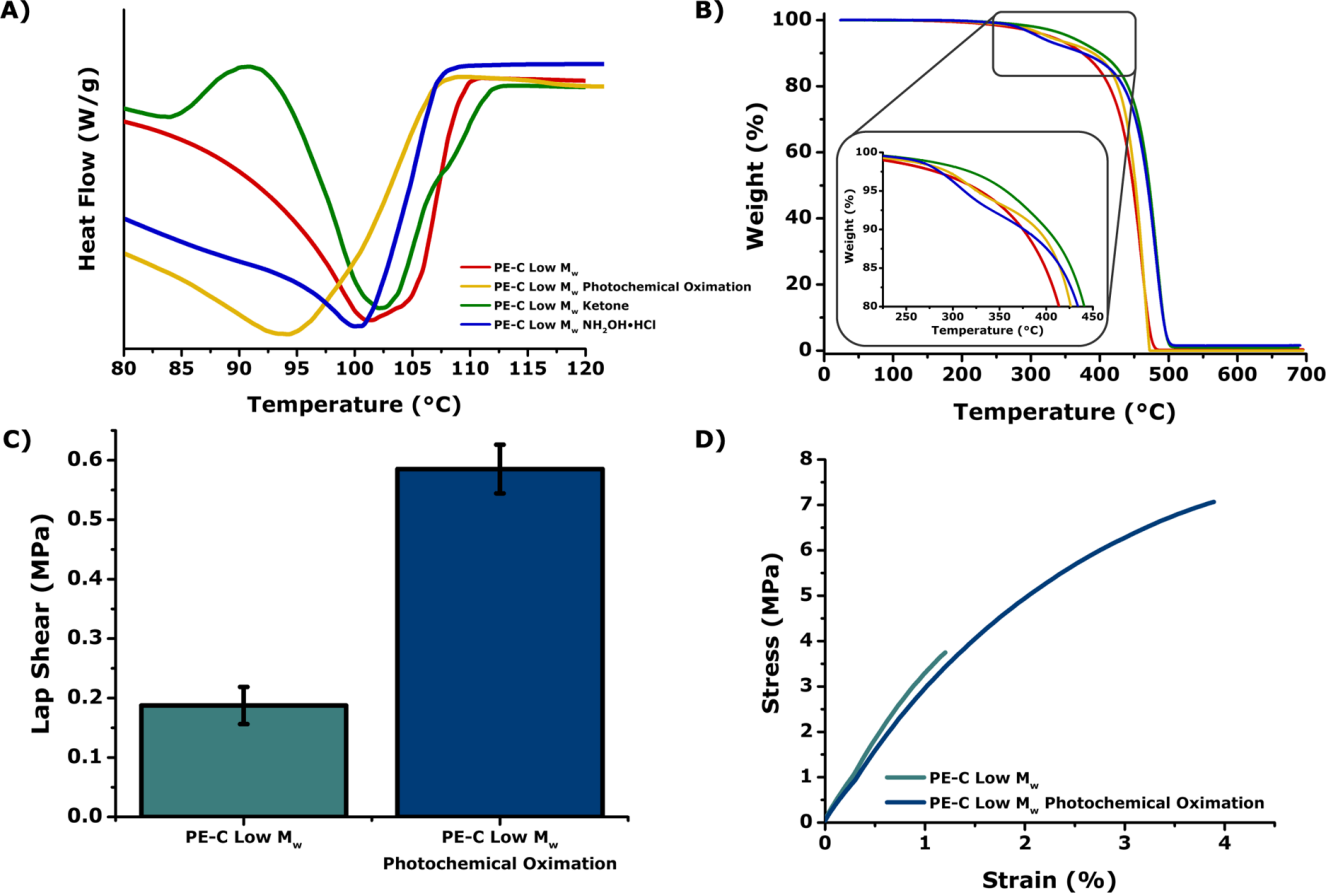
(A) DSC analysis of PE-C **Low*M***_w_ and the corresponding PE after the photochemical oximation reaction, the reflux reaction, and the two-step one-pot reaction with NH_2_OH·HCl. (B) TGA analysis of PE-C **Low*M*_w_** and the corresponding PE after the photochemical oximation reaction, the open reflux setup reaction, and the two-step one-pot reaction with NH_2_OH·HCl. (C) Lap-shear tests of PE **Low*M*_w_** before and after photochemical oximation. (D) Tensile tests of PE-C **Low*M*_w_** before and after photochemical oximation.

The thermal decomposition (*T*_d(90 wt %)_) of the oxime functionalized **PE-S** and **PE-C** **Low *M***_w_ increases by 44.3 and 15.5 °C, respectively, while the *T*_d(90 wt %)_ for the functionalized **PE-P**, **HDPE-C**, and **HDPE-C** high *M*_w_ decrease by 10.7, 76.9, and 2.0 °C, respectively. The higher decomposition temperature for **PE-S** and **PE-C** **Low *M*_w_** can primarily be attributed to the large amount of ketone functional groups present that are reported to increase the *T*_d_ of PE.^19,23^ The decrease in *T*_d_ for **PE-P**, **HDPE-C**, and **HDPE-C** **High *M***_w_ can be attributed to the relative higher oxime loading as well as a higher nitro loading in the case of **HDPE-C** since oximes and nitro groups have been reported to decrease the *T*_d_.^19,23^ For the **PE-C** **Low *M*_w_** sample that was reacted in one pot with NH_2_OH·HCl after the photochemical oximation, this effect is nicely displayed. There, only a minor decrease in *T*_d_ is observed with 2.2 °C. This is, however, much smaller compared to the 15–60 °C decrease observed in the literature and is expected to be caused by the nitro groups present on the backbone.^19,23^ Lastly, the fully ketone functionalized **PE-C** **Low *M*_w_** material displays an increase in *T*_d_ of 24.6 °C, which is in line with the increase observed in the literature between 5 and 45 °C.^19,23^

To conduct preliminary testing of the mechanical properties, the commercially available **PE-C** **Low *M*_w_** and its functionalized derivative (Table 2, entry 4) were evaluated as hot melt adhesives using polycarbonate substrates through lap-shear testing (Figure 5C). The PE materials were applied to the substrates preheated to 140 °C. In subsequent lap-shear tests, the functionalized PE demonstrated a three-fold increase in lap-shear strength compared to the unmodified PE. A similar trend was observed during tensile testing the PE films compression-molded at 140 °C, where the functionalized material exhibited a four-fold increase in elongation (1.1 ± 0.09% elongation prior to functionalization and after functionalized 3.9 ± 0.3%) and in strain at break and a two-fold increase in stress at break relative to the unmodified PE is observed (3.4 ± 0.3 and 7.1 ± 0.4 MPa) (Figure 5D). Despite the considerable enhancement in mechanical performance of the functionalized polymer compared to its parent PE, it should be noted that the absolute values remain relatively low due to the low molar mass of the PE used. Nevertheless, the substantial improvement following the postfunctionalization highlights the potential of this chemistry to enhance the strength and adhesion of PE-based materials. The observed effect of introducing polar groups onto the PE backbone on the mechanical performance is consistent with previously reported results in mechanical performance for oxime- and ketone functionalized PE materials.^19^

## Conclusions

This study presents the first method for direct introduction of oxime functional groups onto a polyethylene backbone. Unlike previous oximation approaches that require multiple steps, precious metal catalysts, harsh conditions, or chlorinated solvents, our robust photochemical methodology operates without a catalyst in the polymer melt and is readily scalable (demonstrated up to a preparative scale of 10 g). The approach achieves FG_Oxime_ as high as 0.5% across various polyethylene grades, including post-consumer materials. The chemistry is versatile, as beyond oximes, ketone and nitro groups can also be incorporated simply by adjusting the reaction conditions. For example, we achieved FG_Ketone_ values up to 2.9% on PE-C Low *M*_w_, a functionalization level previously attainable only through PPM with precious metal catalysis.

Notably, GPC analysis confirms that our photochemical oximation method does not cause radical cleavage of the backbone or crosslinking, making it one of the first radical-based pathways for directly installing oxime, ketone, and nitro groups on different PE types without compromising the backbone integrity. The introduced functional groups on the polymer backbone cause typical changes in the melting and decomposition temperature. At higher FG_Oxime_ and FG_Nitro_, the melting and decomposition temperatures decrease, and at higher FG_Ketone_, these temperatures increase. Initial investigations into the mechanical properties of the functionalized PE demonstrated significant increases in adhesive properties with respect to polycarbonate substrates, showing potential applications in hot melt PE adhesives. Regioselectivity studies, including experiments with ^15^N-labeled PE, reveal that functional groups are randomly distributed along the polymer chain with a slight preference for preterminal positions. Mechanistic insights proved to be key to achieving functional group control. Indeed, we demonstrate that oxime groups convert to ketones under UVA irradiation in the presence of *t*-BuONO through an iminoxyl radical intermediate, which subsequently forms an unstable nitrimine species that decomposes into ketones. Furthermore, we found that the nitro group arises from the formation of a NO_2_^•^, generated by the reaction of NO^•^ (produced from *t*-BuONO) with half an equivalent of O_2_. Promisingly, when the photochemical oximation is performed under a 10 bar N_2_ atmosphere, the selectivity is directed to the oxime as the predominant functional group with minor ketone formation and no formation of the nitro group.

Beyond polyethylene, these findings establish a solid framework for exploring organic-based photochemical methods for PPM of other polyolefins, enabling the direct incorporation of (oxime) functional groups not accessible through metal-catalyzed or bottom-up approaches.

## Supplementary Material



## Data Availability

The data related to the work described in this paper is available in the Yoda repository using the following link: https://doi.org/10.24416/UU01-WZN5HW.

## Notes

Experimental section, photochemical oximation reactions on small model compounds and PE materials as well as NMR spectra, FTIR spectra and thermogravimetric analysis (TGA), differential scanning calorimetry (DSC) traces, gel permeation chromatography (GPC) traces, and electron paramagnetic resonance (EPR) data with the EPR simulations ran with EasySpin in Matlab (PDF)

## Abbreviations

PPM: post-polymerization modification
PE: polyethylene
*t*-BuONO: *tert*-butyl nitrite
GPC: gel permeation chromatography
MDSC: modulated differential scanning calorimetry
TGA: thermal gravimetric analysis
EPR: electron paramagnetic resonance
