# Supplementary material for: Post-Polymerization Modification of Polyethylene through Photochemical Oximation and Consecutive Ketonization
Source: J Am Chem Soc. 2025 Jun 17;147(26):22827–38. doi: 10.1021/jacs.5c05212 (PMC12232331; doi:10.1021/jacs.5c05212)
Supplement: Supplementary file 1 [file ja5c05212_si_001.pdf]

Supporting Information

Post-Polymerization Modification of Polyethylene through Photochemical Oximation and Consecutive Ketonization

Maartje Otten[a], Inèz Klein Gebbink[a], Patrick J. Schara[b], Željko Tomović[b], Martin Lutz[c], Pieter C.A. Bruijnincx[a*] and Arnaud Thevenon[a*]

a) Organic Chemistry & Catalysis, Institute for Sustainable and Circular Chemistry, Faculty of Science, Utrecht University, Universiteitsweg 99, 3584 CG, Utrecht, The Netherlands

b) Polymer Performance Materials, Department of Chemical Engineering and Chemistry, Technical University Eindhoven, 5600 MB, Eindhoven, The Netherlands

c) Structural Biochemistry, Bijvoet Centre for Biomolecular Research, Faculty of Science, Utrecht University, Universiteitsweg 99, 3584 CG, Utrecht, The Netherlands

*Corresponding Authors: Pieter C.A. Bruijnincx and Arnaud Thevenon, Organic Chemistry & Catalysis, Institute for Sustainable and Circular Chemistry, Faculty of Science, Utrecht University, Universiteitsweg 99, 3584 CG Utrecht, The Netherlands; Email: p.c.a.bruijnincx@uu.nl; a.a.thevenon-kozub@uu.nl

Table of Contents

1. General 2
2. Experimental section 4
3. Crystal structure determination data 9
4. Characterization PE-S 10
5. Photochemical oximation of model linear hydrocarbons and polyethylene data 14
6. Reference oxime compounds data 42
7. DSC and TGA Data 58
8. GPC Data 68
9. Mechanical Properties 69
10. Data Of Iminoxyl Radical Investigation 70
References 79

1. General

Unless stated otherwise, all the photochemical reactions were performed in the presence of oxygen using an ACE pressure tube #15. For high pressure reactions a 50 mL custom made Parr window autoclave was used at 10 bar N_2, see **Section 5** for the used set-up. The 370 nm UVA LED PR160L lamp used during the experiments was purchased from Kessil and always used on 100% intensity at 6 cm distance from the reaction vessel. Polyethylene was either acquired form Sigma Aldrich with a M_w of 4000 g/mol (LDPE) or LyondellBasell with a M_w of 56000 g/mol and 114200 g/mol (HDPE), or self-synthesized according to the protocol in **section 2** with a M_w of 1500 g/mol (HDPE), or consumer packaging was used with a M_w of 87300 g/mol (LDPE). The solvents 1,2,4-trichlorobenzene, dichloromethane, 1,1,2,2-tetrachloroethane and methanol were purchased from commercial sources and used without further purification. Toluene, diethyl ether and hexane were taken from an MBraun MB SPS-80 purification system dried further over 4 Å molecular sieves, degassed and tested for water content by the Karl-Fisher titration before use. Tetrahydrofuran (THF) was dried over sodium benzophenone ketyl, distilled under inert N_2(g) and tested for water content by the Karl-Fisher titration before use. All reagents and starting materials were purchased from commercial sources and used without further purification, except when specified. Deuterated solvents were purchased from the Cambridge Isotope Laboratory Incorporation (Cambridge, USA) or Sigma-Aldrich and used as received.

NMR spectroscopy (^{1}H, $^{13}C\{^{1}H\}$, $^{1}H^{13}C$ and $^{1}H^{15}N$) was conducted using a 400 MHz Varian spectrometer equipped with AutoX probe and Agilent ProTune probe tuning accessory or a 400 MHz Jeol EZCL G spectrometer with a HFX probe. ^{1}H and ^{13}C NMR chemical shift are reported in the standard δ notation of part per million (ppm) and are referenced to a residual peak of the solvent, as determined relative to SiMe4. For the reported $^{1}H^{15}N$ Heteronuclear Multiple Bond Correlation spectra the signals showed are externally referenced to ammonia. Infrared spectroscopy was conducted using a PerkinElmer SpectrumTwo FT-IR Spectrometer equipped with an ATR-probe. Peaks are annotated by (w), (m) and (s) to indicate weak, medium and strong signals, respectively. ESI-MS spectrometry was conducted using a Advion Expression CMS spectrometer. EPR measurements were recorded on a Bruker EMX Plus 6000 Gauss spectrometer equipped with an ER 041 XG X-Band Microwave Bridge. Simulated EPR spectra were ran using EasySpin software.

Thermogravimetric analysis (TGA) measurements were performed on a TA Instruments TGA Q50 Thermographic Analyzer under nitrogen atmosphere on a platinum pan and analyzed using TA Instruments Universal Analysis 2000 software. The thermal degradation was investigated with approximately 10-20 mg sample, loaded onto the pan and heated from ambient temperature to 700 °C with a rate of 10 °C/min. Modulated Differential Scanning Calorimetry (MDSC) measurements were performed on a TA Instruments Discovery DSC equipped with a TA Instruments Refrigerated Cooling System 90 and analyzed with TA Instruments Trios Software (version 5.7.1.74). Tzero low mass aluminium pans were loaded with approximately 3-5 mg of sample and closed with a Tzero aluminium lid. Samples were first kept at 25 °C and thereafter cooled down at a rate of 2.00 °C/min to -90 °C. After 10 min at -90 °C, the sample was heated to 140 °C at a rate of 2.00 °C/min. After 10 min at 140 °C, the sample was cooled down to 25 °C. The modulation was set at 1 °C/min. All measurements were ran multiple times and no deviation larger than 0.2 °C for the T_m was observed.

Gel permeation chromatography (GPC) measurements were performed on a Polymer Char (Valencia Spain) GPC-IR instrument equipped with an infrared detector (IR4). Three PLgel Olexis columns (300 x 7.5 mm) from Agilent Technologies were used in series and a PLGel

Olexis guard column (50 x 7.5 mm) from Agilent Technologies was used. The GPC eluent used was 1,2,4-trichlorobenzene, containing 300 ppm butylated hydroxytoluene, at a flow rate of 1.0 mL/min with a column temperature of 150 °C. The PE samples were prepared with a concentration of 1.0 mg/mL with heptane as internal standard. The samples were dissolved at 160 °C under N_2 atmosphere for 1 hour while continuously shaking gently and thereafter filtered prior to injection. The molecular weight was calculated with respect to polystyrene standards (Polymer Laboratories, M_n = 5310 g/mol up to 1510000 g/mol) and converted to polyethylene equivalents.

For tensile testing samples were prepared by placing PE samples in a stainless-steel mold (5 mm thickness) and hot-pressing at 140 °C under a pressure of 20 kN for 15 minutes to ensure homogeneous, flat specimens. Tensile tests were conducted using a Zwick/Roell Intelligent Testing Machine equipped with a 200 N load cell. Dumbbell-shaped specimens with an effective length of 12 mm, width of 2 mm, and a measured thickness of approximately 0.5 mm were tested at a strain rate of 1 mm/min with a preload of 0.1 N. The results represent the mean values obtained from at least four independent samples.

Lap-shear testing was performed on glued polycarbonate substrates. Polycarbonate substrates (100 mm × 25 mm × 5 mm) were preheated in a 140 °C oven, and bonded using ~0.1 g of PE adhesive with a 2.5 × 2.5 cm² overlap, spacers were utilized to ensure an adhesive layer thickness of ~0.1 mm. A 600 g weight was placed on top of the assembly for one hour before cooling to room temperature. Lap-shear tests were performed using a Zwick/Roell Intelligent Testing Machine equipped with a 2.5 kN load cell at a strain rate of 1 mm/min and a preload of 1 N. Results are reported as the mean values of at least four independent measurements.

2. Experimental Section

Synthesis of $Cp^*_2NdCl_2Li(Et_2O)$

Lanthanocene chloride complexes, and specifically the neodymium analogue, have been reported several times to catalyze the chain growth reaction of ethylene when combined with an excess of a di-alkyl magnesium compound.[1–3] Long di-alkyl magnesium chains are obtained after the reaction, which upon quenching with methanol and hydrochloric acid give PE with a polydispersity index (PDI) corresponding to a Poisson distribution. While the synthesis for the $Cp^*_2NdCl_2Li(Et_2O)$ complex has been reported for over 40 years and used for the polymerization of ethylene, the crystal structure was never resolved. In this work we present the crystal structure of the Nd-complex that displays a comparable geometry to its cerium, samarium, ytterbium, and lutetium.[4–7] For all four analogues the bond lengths between the lanthanide and Cp* are in line with the atomic radius of the lanthanide. The crystal structure also displays the second largest distance between the lanthanide and lithium which is in line with the atomic radii of the atoms, due to the increasing effective nuclear charge.

Lithium pentamethylcyclopentadienyl (LiCp)*[8]

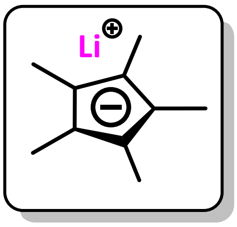

Pentamethylcyclopentadiene (2 g; 14.7 mmol) was dissolved in dry and degassed hexane (70 mL) under a N_2 atmosphere in a Schlenk. The reaction mixture was cooled down to -78 °C using a dry ice/acetone bath whereafter *n*-butyllithium (1.6 M in hexane) (9.6 mL; 15.4 mmol) was added dropwise. After stirring the reaction mixture for 30 minutes at -78 °C, the reaction mixture was allowed to warm up to ambient temperature and the colorless solution was left to stir for 16 hours. The solids were washed twice with 10 mL dry and degassed hexane. The obtained white solids after filtration were dried in *vacuo* and stored under N_2 atmosphere. The title compound was obtained as a white solid (1.76 g; 84%).

Lithium Bis(pentamethylcyclopentadienyl)dichlorobis(diethyl ether)Neodymate(III)[9]

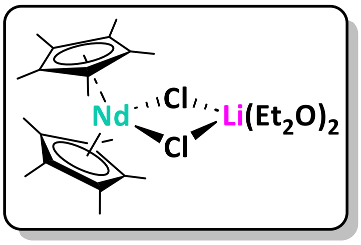

Neodymium trichloride (176 mg; 0.7 mmol) and lithium pentamethylcyclopentadienyl (201 mg; 1.4 mmol) were dissolved in dry and degassed THF (7.5 mL) under a N_2 atmosphere in a Schlenk. The brown suspension was refluxed for 16 hours, whereafter the mint green suspension was allowed to cool down to ambient temperature. The THF was removed in *vacuo* and the remaining mint green solids were washed twice with dry and degassed diethyl ether (5 mL). The white solids were filtered off, whereafter a green/blue ether extract was obtained and cooled to -40 °C to yield large dark blue crystals suitable for X-ray crystallography. The title compound was obtained as dark blue solids (269 mg; 60%).

Polyethylene synthesis

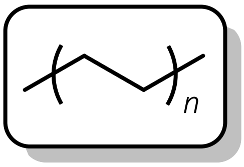

Lithium Bis(pentamethylcyclopentadienyl)dichlorobis(diethyl ether)-Neodymate(III) (2.6 mg; 4.1 μmol) was mixed with dry and degassed toluene (10 mL). di-*n*-Butyl magnesium (0.5 M in heptane) (0.4 mL; 0.2 mmol) was added to the mixture and stirred for 30 minutes. To a 500 mL Schlenk, dry and degassed toluene (20 mL) was added whereafter the toluene was freeze-pump-thawed before applying 1 bar of ethylene. The Schlenk with 1 bar of ethylene was heated to 80 °C, whereafter the 10 mL Neodymium catalyst solution with di-*n*-butyl magnesium was added to the Schlenk. With a manometer the ethylene flow was monitored and after 30 minutes the reaction was cooled down to ambient temperature. The reaction mixture was

thereafter quenched with methanol (150 mL) while vigorously stirring. The formed white solid flakes were thereafter filtered and washed extensively with methanol. The obtained white solids were dried in a vacuum oven at 75 °C for 24 hours, whereafter the solids were grinded with a mortar and pestle. The title compound was obtained as a white powder (1.6 g).

^{1}H NMR (400 MHz, Toluene-*d8*, 100 °C) δ = 1.34 (s, 357H, CH_2), 0.89 (t, 6H, $-CH_3$) ppm.

^{13}C NMR (101 MHz, Toluene-*d8*, 100 °C) δ = 14.3, 30.0, 30.5, 32.6 ppm.

Synthesis of Tert-Butyl Nitrite ^{15}N-labeled

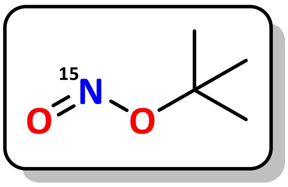

A solution of sodium nitrite {98% atom ^{15}N labelled} (1.0 g; 14.3 mmol) in 3.6 mL water and *t*-butanol (1.1 mL; 11.9 mmol) were mixed in a round bottom flask and cooled down to 0 °C. Sulfuric acid (1.62 mL, 35% solution in water) was added over the course of 2 hours at the bottom of the flask using a syringe pump. The reaction mixture was allowed to warm up to ambient temperature whereafter the organic layer was washed with water and subsequently with a 5% sodium bicarbonate solution. The crude product was thereafter further purified using distillation. The title compound was obtained as a yellow liquid (0.42 mL; 25%).

Cyclohexanone oxime

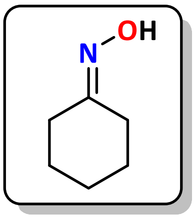

Hydroxylamine hydrochloride (1.56 g; 22.4 mmol) was dissolved in water (2.0 mL) and methanol (10.0 mL). Cyclohexanone (2.11 mL; 20.4 mmol) was added to the mixture whereafter sodium bicarbonate (1.88 g; 22.4 mmol) was added portion wise to the mixture until gas formation was no longer observed. The reaction mixture was left to stir for 24 hours at ambient temperature and thereafter water (60 mL) was added. The mixture was washed with dichloromethane and the organics were thereafter washed with water. The organics were subsequently dried over magnesium sulphate and the product was concentrated in vacuo. The title compound was obtained as white crystals (2.24 g; 97%).

^{1}H NMR (400 MHz, $CDCl_3$, 25 °C) δ = 7.86 (s, 1H, -OH), 2.50 (t, 2H, HONC-**CH_2**), 2.21 (t, 2H, HONC-**CH_2**), 1.62 (m, 6H, $-CH_2$) ppm.

^{13}C NMR (101 MHz, $CDCl_3$, 25 °C) δ = 161.1, 32.3, 27.1, 26.0, 25.8, 24.5 ppm.

IR (ATR): ν = 3182 (s), 3108 (s), 2929 (s), 2892 (s), 2859 (s), 1663 (m), 1479 (s), 1448 (s), 1436 (s), 1350 (w), 1339 (w), 1318 (m), 1253 (w), 1225 (m), 1140 (w), 1105 (m), 986 (m), 953 (s), 924 (s), 840 (w), 808 (w), 757 (m), 729 (s), 647 (m), 597 (w) cm^{-1}.

Synthesis of 6-undecanone oxime

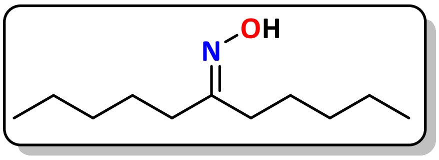

Hydroxylamine hydrochloride (450 mg; 6.46 mmol) was dissolved in water (0.5 mL) and methanol (2.5 mL). 6-undecanone (1.2 mL; 5.87 mmol) was added to the mixture whereafter sodium bicarbonate (543 mg; 6.47 mmol) was added portion wise to the mixture until gas formation was no longer observed. The reaction mixture was left to stir for 24 hours at ambient temperature and thereafter water (30 mL) was added. The mixture

was washed with dichloromethane and the organics were thereafter washed with water. The organics were subsequently dried over magnesium sulphate and the product was concentrated in vacuo. The title compound was obtained as a colorless oil (0.99 g; 91%).

^{1}H NMR (400 MHz, $CDCl_3$, 25 °C) δ = 8.23 (s, 1H, -OH), 2.33 (t, 2H, HONC-**CH_2**), 2.16 (t, 2H, HONC-**CH_2**), 1.50 (quint, 4H, -CH_2), 1.30 (m, 8H, -CH_2), 0.89 (t, 6H, -CH_3) ppm.

^{13}C NMR (101 MHz, $CDCl_3$, 25 °C) δ = 161.9, 34.2, 32.2, 31.6, 27.6, 26.2, 25.4, 22.5, 22.5, 14.0 ppm.

ESI-MS (acetonitrile): m/z = 170.8 {$[M+H^+-OH^-]^+$, calc. 170.1}.

IR (ATR): ν = 3230 (s), 3099 (s), 2955 (s), 2916 (s), 2871 (s), 2859 (s), 2731 (w), 2668 (w), 1655 (m), 1465 (s), 1459 (s), 1378 (m), 1352 (w), 1338 (w), 1310 (w), 1271 (w), 1249 (w), 1213 (w), 1184 (w), 1115 (m), 988 (m), 950 (s), 920 (s), 840 (w), 809 (w), 759 (m), 727 (s), 647 (m), 597 (w) cm^{-1}.

Synthesis of 3-pentanone oxime

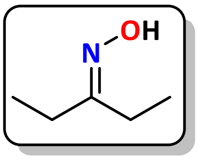

Hydroxylamine hydrochloride (1.77 g; 25.5 mmol) was dissolved in water (2.0 mL) and methanol (10.0 mL). 3-pantanone (2.46 mL; 23.2 mmol) was added to the mixture whereafter sodium bicarbonate (2.14 g; 25.5 mmol) was added portion wise to the mixture until gas formation was no longer observed. The reaction mixture was left to stir for 24 hours at ambient temperature and thereafter water (60 mL) was added. The mixture was washed with dichloromethane and the organics were thereafter washed with water. The organics were subsequently dried over magnesium sulphate and the product was concentrated in vacuo. The title compound was obtained as a colorless oil (1.91 g; 81%).

^{1}H NMR (400 MHz, $CDCl_3$, 25 °C) δ = 9.63 (s, 1H, -OH), 2.36 (q, 2H, HONC-**CH_2**), 2.21 (q, 2H, HONC-**CH_2**), 1.07 (t, 6H, -CH_3) ppm.

^{13}C NMR (101 MHz, $CDCl_3$, 25 °C) δ = 163.6, 27.1, 20.9, 10.9, 10.7 ppm.

IR (ATR): ν = 3244 (s), 3099 (s), 2974 (s), 2938 (s), 2879 (s), 1659 (m), 1462 (s), 1375 (m), 1335 (w), 1267 (w), 1254 (w), 1220 (w), 1206 (w) 1100 (m), 1083 (w), 998 (m), 950 (s), 920 (s), 840 (w), 809 (w), 759 (m), 727 (s), 647 (m), 597 (w) cm^{-1}.

Photochemical oximation of n-Undecane

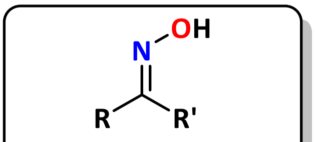

Starting from *n*-undecane

n-Undecane (4.22 mL; 20 mmol) and *t*-butanol (1.5 mL; 16 mmol) were mixed in a pressure tube equipped with a stir bar. *t*-Butyl nitrite (0.24 mL; 2 mmol) was added to the mixture and heated to 105 °C whereafter the UVA lamp was turned on. The reaction mixture was left to stir for 16 hours and cooled down prior to adding 250 mg activated charcoal. The mixture was reheated to 50 °C and left to stir for 2 hours. The resulting slurry was filtered over a silica plug and washed with dichloromethane. The product was thereafter concentrated in vacuo whereafter the crude was further purified using vacuum distillation. The title compound was obtained as a pale yellow liquid (312 mg; 84%).

^{1}H NMR (400 MHz, $CDCl_3$, 25 °C) δ = 9.15 (s, 1H, -OH), 2.35 (m, 3H, HONC-**CH_2**), 2.19 (m, 3H, HONC-**CH_2**), 1.84 (d, 3H, HONC-**CH_3**), 1.48 (m, 9H, -CH_2), 1.24 (m, 47H, -CH_2), 0.86 (t, 19H, -CH_3) ppm.

^{13}C NMR (101 MHz, $CDCl_3$, 25 °C) δ = 163.1, 163.0 162.2, 162.0, 159.2, 158.8, 85.3, 85.1, 43.0, 42.6, 36.3, 36.0, 34.2, 34.2, 33.9, 33.9, 32.2, 32.1, 32.0, 31.9, 31.7, 31.6, 30.0, 30.0, 29.9, 29.7, 29.6, 29.5, 29.5, 29.4, 29.4, 29.3, 29.3, 29.2, 29.2, 28.7, 28.6, 27.9 27.7, 27.6, 27.6, 27.4, 26.5, 26.4, 26.1, 25.8, 25.6, 25.5, 23.1, 22.8, 22.7, 22.6, 20.9, 19.7, 14.5, 14.2, 14.0, 13.9, 13.4, 10.9 ppm.

ESI-MS (acetonitrile): m/z = {[M+H$^+$-OH$^-$]$^+$, calc. 170.1}.

IR (ATR): ν = 3252 (s), 3102 (s), 2955 (s), 2923 (s), 2871 (s), 2854 (s), 2730 (w), 2666 (w), 1716 (m), 1658 (m), 1626 (s), 1551 (m), 1460 (s), 1378 (m), 1365 (m), 1337 (w), 1274 (m), 1113 (m), 1059 (w), 1006 (w) 952 (s), 867 (m), 756 (w), 722 (m), 698 (w), 670 (w), 647 (m), 608 (w), 576 (w) cm^{-1}.

Photochemical oximation of n-Tetradecane

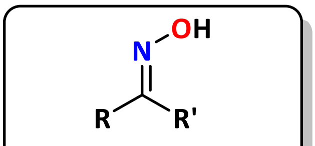

Starting from *n*-tetradecane

n-Tetradecane (5.20 mL; 20 mmol) and *t*-butanol (1.5 mL; 16 mmol) were mixed in a pressure tube equipped with a stir bar. *t*-Butyl nitrite (0.24 mL; 2 mmol) was added to the mixture and heated to 105 °C whereafter the UVA lamp was turned on. The reaction mixture was left to stir for 16 hours and the resulting slurry was filtered over a silica plug and washed with dichloromethane. The product was thereafter concentrated in vacuo whereafter the crude was further purified using vacuum distillation. The title compound was obtained as a pale yellow liquid (324 mg; 71%).

^{1}H NMR (400 MHz, $CDCl_3$, 25 °C) δ = 9.05 (s, 4H, -OH), 2.39 (m, 13H, HONC-**CH_2**), 2.18 (m, 13H, HONC-**CH_2**), 1.86 (d, 3H, HONC-**CH_3**), 1.52 (m, 28H, -CH_2), 1.27 (m, 572H, -CH_2), 0.89 (t, 138H, -CH_3) ppm.

^{13}C NMR (101 MHz, $CDCl_3$, 25 °C) δ = 161.9, 158.5, 89.2, 83.7, 44.0, 43.0, 36.3, 36.1, 36.0, 35.4, 34.3 34.1, 33.8, 32.3, 32.2, 32.0, 31.8, 31.7, 30.1, 29.9, 29.8, 29.7, 29.6, 29.5, 29.4, 29.3, 29.2, 28.9, 28.6, 28.0, 27.7, 27.5, 27.4, 26.5, 26.2, 26.0, 25.9, 25.8, 24.1, 23.2, 22.9, 22.8, 22.6, 19.8, 19.3, 14.5, 14.2, 14.1, 14.0, 13.4, 10.9, 10.4, 1.1 ppm.

IR (ATR): ν = 3271 (w), 3105 (w), 2957 (s), 2921 (s), 2872 (s), 2853 (s), 2732 (w), 2677 (w), 1719 (m), 1660 (w), 1630 (m), 1552 (s), 1466 (s), 1459 (s), 1377 (m), 1352 (w), 1338 (w), 1304 (w), 1261 (w), 1097 (m), 1018 (m), 957 (w), 944 (s), 862 (w), 804 (m), 722 (m), 662 (w) cm^{-1}.

Photochemical oximation of n-Octadecane

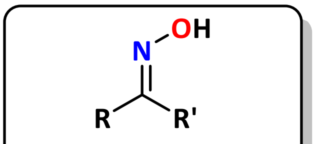

Starting from *n*-octadecane

n-Octadecane (6.55 mL; 20 mmol) and *t*-butanol (1.5 mL; 16 mmol) were mixed in a pressure tube equipped with a stir bar. *t*-Butyl nitrite (0.24 mL; 2 mmol) was added to the mixture and heated to 105 °C whereafter the UVA lamp was turned on. The reaction mixture was left to stir for 16 hours and the resulting slurry was filtered over a silica plug and washed with dichloromethane. The product was thereafter concentrated in vacuo whereafter the crude was further purified using vacuum distillation. The title compound was obtained as a pale yellow liquid (479 mg; 84%).

^{1}H NMR (400 MHz, $CDCl_3$, 25 °C) δ = 9.20 (s, 6H, -OH), 2.39 (m, 14H, HONC-**CH_2**), 2.20 (m, 14H, HONC-**CH_2**), 1.88 (d, 3H, HONC-**CH_3**), 1.54 (m, 40H, -CH_2), 1.30 (m, 1300H, -CH_2), 0.91 (t, 240H, -CH_3) ppm.

^{13}C NMR (101 MHz, $CDCl_3$, 25 °C) δ = 162.6, 162.4, 161.7, 161.6, 161.5, 158.7, 158.3, 89.1, 85.0, 43.0, 36.4, 36.1, 34.4, 34.3, 34.1, 32.7. 32.4, 32.3, 32.1, 31.9, 31.8, 30.2, 30.0, 29.9, 29.8, 29.7, 29.6, 29.5, 29.4, 29.3, 28.7, 28.1, 27.9, 27.8, 27.7, 27.5, 26.6, 26.3, 26.1, 26.0, 25.9, 25.6, 23.3, 23.0, 22.8, 22.7, 21.1, 20.0, 19.8, 19.4, 14.5, 14.3, 14.2, 14.1, 14.0, 13.4, 10.9, 10.23, 1.2 ppm.

IR (ATR): ν = 3206 (w), 2961 (s), 2953 (s), 2912 (s), 2870 (s), 2847 (s), 2729 (w), 2634 (w), 1722 (w), 1682 (w), 1630 (w), 1558 (w), 1470 (s), 1452 (m), 1369 (m), 1338 (w), 1306 (w), 1300 (w), 1284 (w), 1270 (w), 1260 (w), 1230 (w), 1190 (w), 1120 (w), 1062 (w), 1052 (w), 1043 (w), 1025 (w), 997 (w), 977 (w), 930 (w), 891 (m), 858 (w), 794 (w), 749 (w), 715 (m) cm^{-1}.

Photochemical oximation n-Hexatriacontane and PE materials

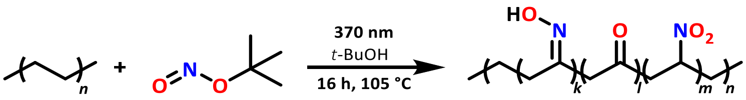

n-Hexatriacontane or PE (100-10000 mg; 1.0 equivalent) and *t*-butanol (0.8 equivalent) were mixed in a pressure tube equipped with a stir bar. *t*-Butyl nitrite (0.1 equivalent) was added to the mixture and heated to 105 °C or 125 °C for HDPE-C and HDPE-C High M_w, while vigorously stirring, whereafter the UVA lamp was turned on.* The reaction mixture was left to stir for 16 hours and the resulting suspension upon cooling was filtered. (Upon opening the pressure tube a significant release of pressure was observed). The obtained solids were washed with dichloromethane and methanol. The solids were thereafter extensively dried in a vacuum oven at 80 °C for 4 days. *n*-Hexatriacontane oxime was obtained as a off-white solid (321 mg; 60%). All PE materials were reobtained as beige/pale yellow solids with 97-99% wt%.

*If the reaction mixture is not stirred, the reagents are not mixing with the polymer and form a layer on top of the molten polymer layer.

3. Crystal Structure Determination Data

X-ray crystal structure determinations were performed on a Bruker Kappa ApexII diffractometer with sealed tube and Triumph monochromator (λ = 0.71073 Å) at a temperature of 150(2) K up to a resolution of (sin θ/λ)max = 0.71 $Å^{-1}$. The Eval15 software was used for the intensity integration.[10] A numerical absorption correction and scaling was performed with SADABS (correction range 0.62-0.83).[11] A total of 51545 reflections were measured, 4673 reflections were unique (R_{int} = 0.028), 4412 reflections were observed [$I>2\sigma(I)$]. Initial coordinates were taken from the isostructural Lu complex.[6] Structure refinement was performed with SHELXL-2018 on F^2 of all reflections.[12] Non-hydrogen atoms were refined freely with anisotropic displacement parameters. Hydrogen atoms were introduced in calculated positions and refined with a riding model. 162 Parameters were refined with no restraints. R1/wR2 [$I > 2\sigma(I)$]: 0.0178 / 0.0480. R1/wR2 [all refl.]: 0.0191 / 0.0483. S = 1.119. Residual electron density between -0.27 and 1.62 e/$Å^3$ (in proximity to the Nd atom). Geometry calculations and checking for higher symmetry was performed with the PLATON program.[13]

$C_{28}H_{50}Cl_2LiNdO_2$, Fw = 640.76, light-blue block, 0.32 × 0.18 × 0.14 mm^3, monoclinic, C2/c (no. 15), a = 16.4439(2), b = 13.85472(19), c = 13.96713(18) Å, β = 90.988(1) °, V = 3181.59(8) $Å^3$, Z = 4, D_x = 1.338 g/cm^3, μ = 1.82 mm^{-1}.

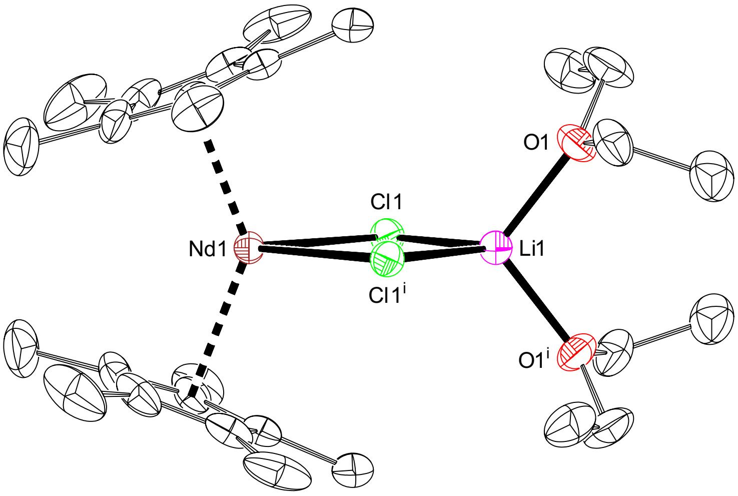

Figure S1: *Molecular structure of $Nd(Cp^*)_2Cl_2Li(Et_2O)_2$ in the crystal (50% probability level). All hydrogen atoms have been omitted for clarity. Symmetry code i: 1-x, y, ½-z. The crystal structure is isostructural to the corresponding Lu complex.*[6]

Table S1: *Selected bond lengths, atom distances and bond angles.*

Bond/distances	Length (Å)	Bond	Angle (°)
Nd1-Cl1	2.7149(3)	Cp^*-Nd1-Cp^*(i)	137.73(3)
Nd1-Cp^* centroid	2.4762(7)	Cp^*(i)-Nd1-Cl1	104.00(2) or 107.00(2)(i)
Nd1---Li1	3.589(3)	Cl1-Li1-O1(i)	109.66(4) or 117.14(4)(i)
Li1-Cl1	2.411(2)	Cl1-Li1-Cl1(i)	98.26(12)
Li1-O1	1.946(2)	O1-Li1-O1(i)	105.46(18)

"CCDC 2386378 contains the supplementary crystallographic data for this paper. These data can be obtained free of charge from The Cambridge Crystallographic Data Centre via www.ccdc.cam.ac.uk/data_request/cif."

4. Characterization PE-S

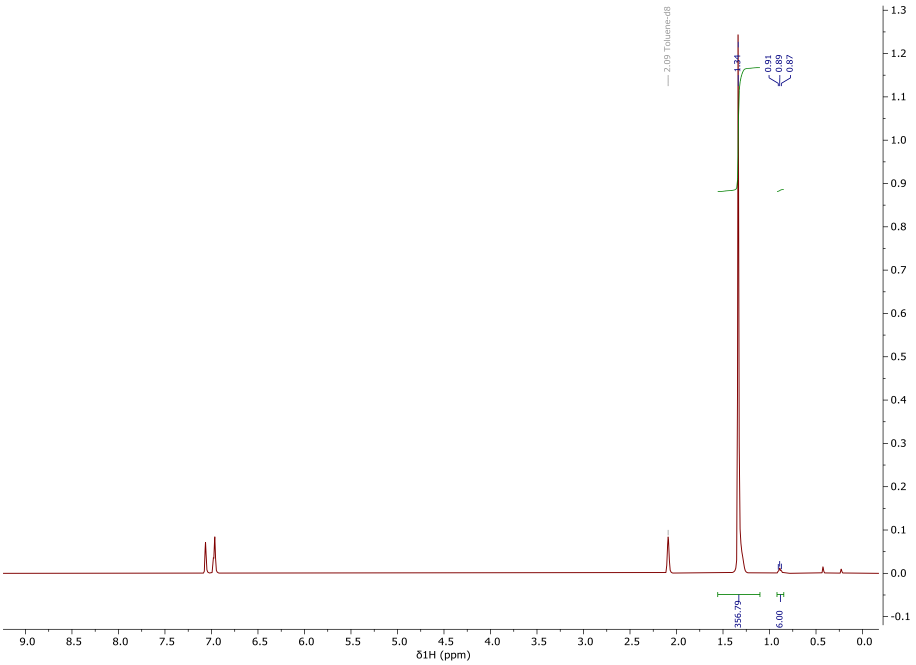

Figure S2: *¹H NMR spectrum of polyethylene in Toluene-d8 at 100 °C.*

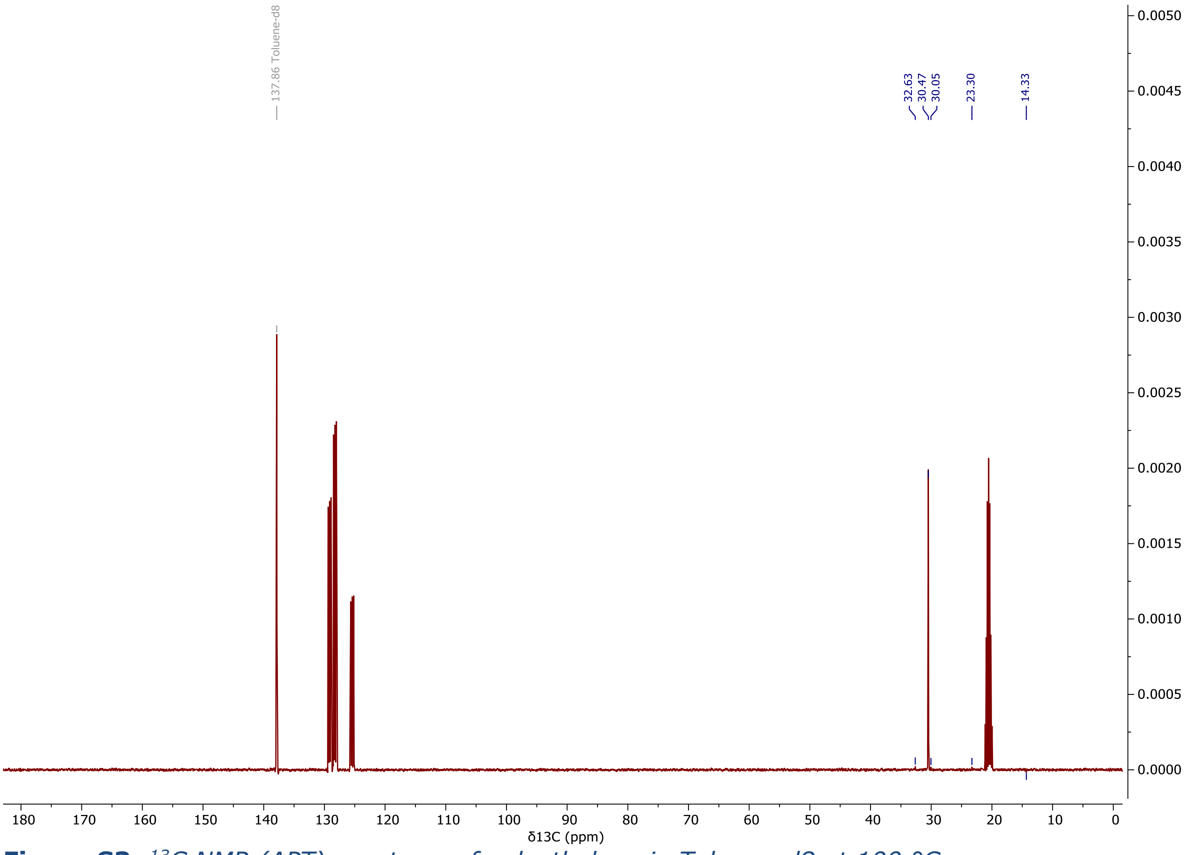

Figure S3: *¹³C NMR (APT) spectrum of polyethylene in Toluene-d8 at 100 °C.*

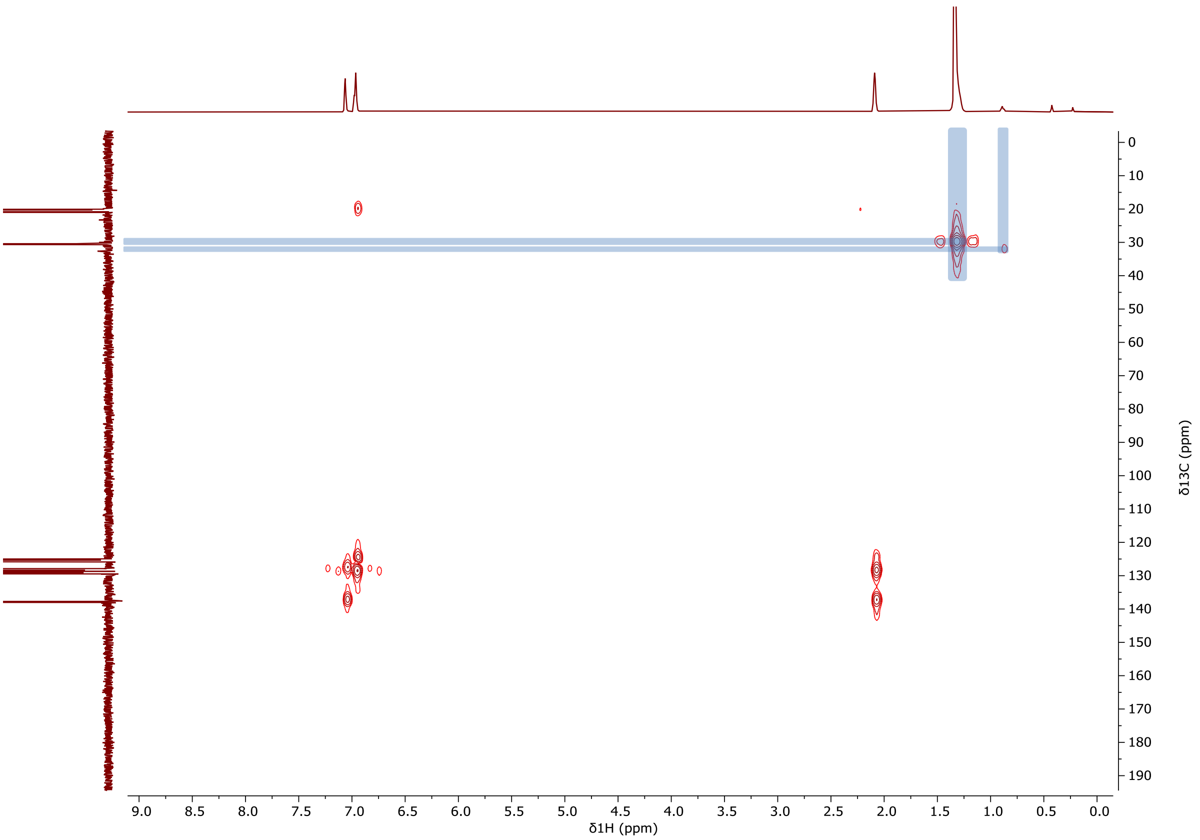

Figure S4: *¹H-¹³C Heteronuclear Multiple Bond Correlation (HMBC) spectrum of polyethylene in Toluene-d8 at 100 °C.*

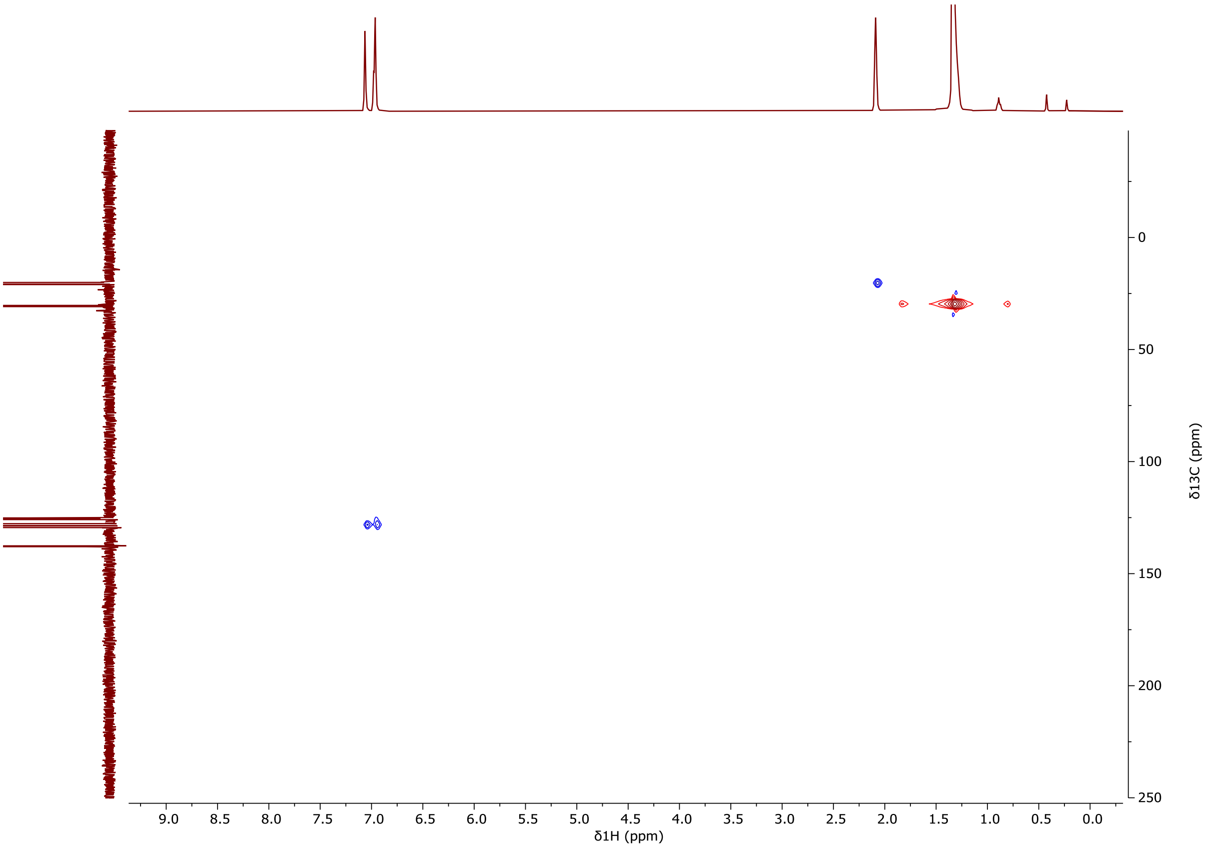

Figure S5: *¹H-¹³C Heteronuclear Single Quantum Coherence (HSQC) spectrum of polyethylene in Toluene-d8 at 100 °C.*

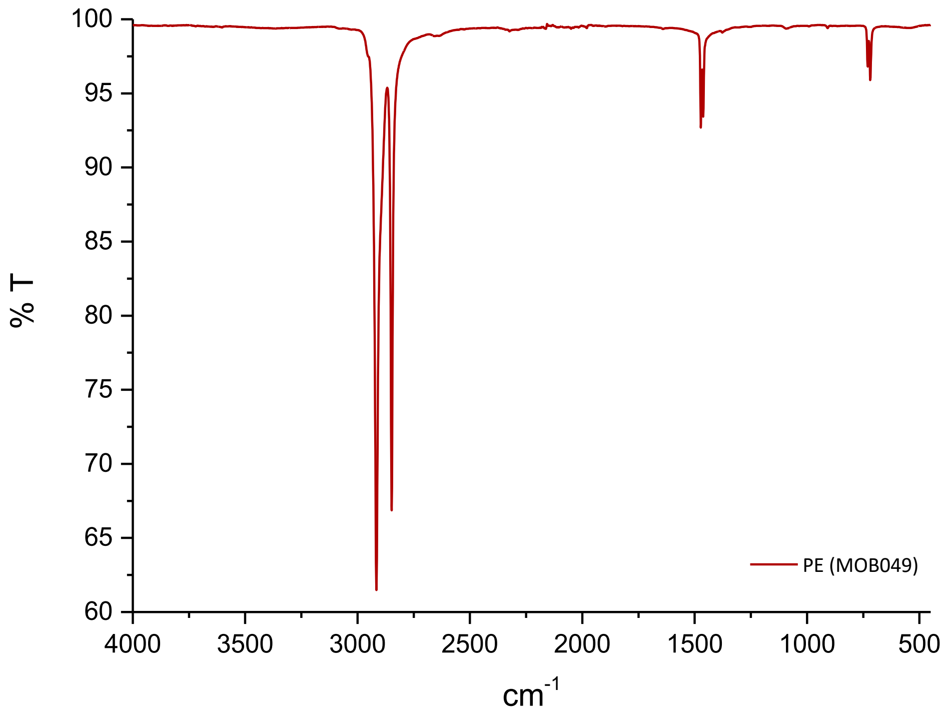

Figure S6: *FTIR (ATR) Spectrum of Polyethylene.*

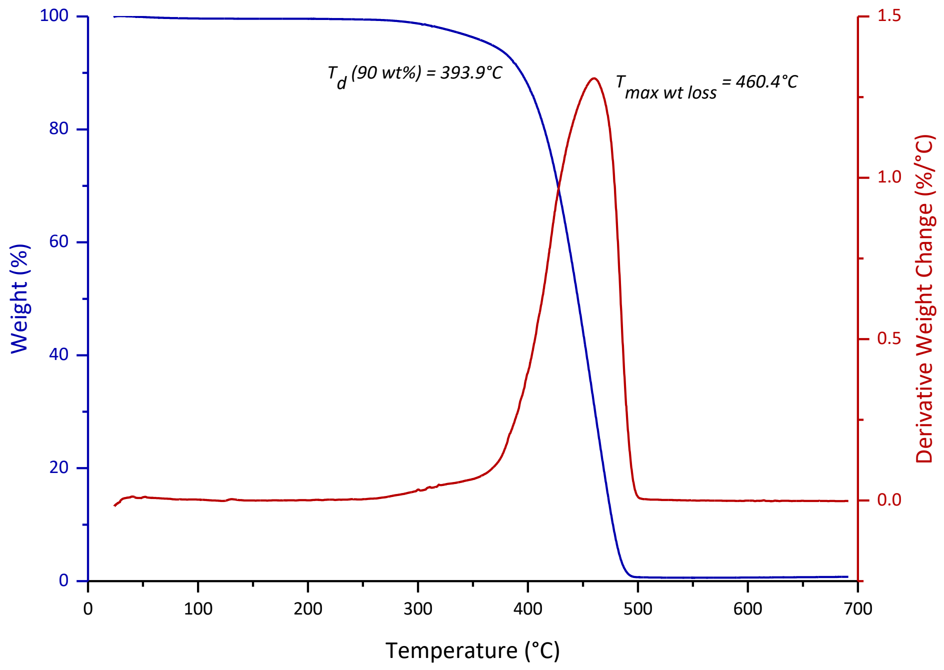

Figure S7: *Thermogravimetric Analysis (TGA) of synthesized Polyethylene. The blue trace depicts the weight loss percentage with a decomposition temperature of 383.9 °C at 90 wt%. The red trace depicts the derivative weight change with a maximum weight loss at 445.4 °C.*

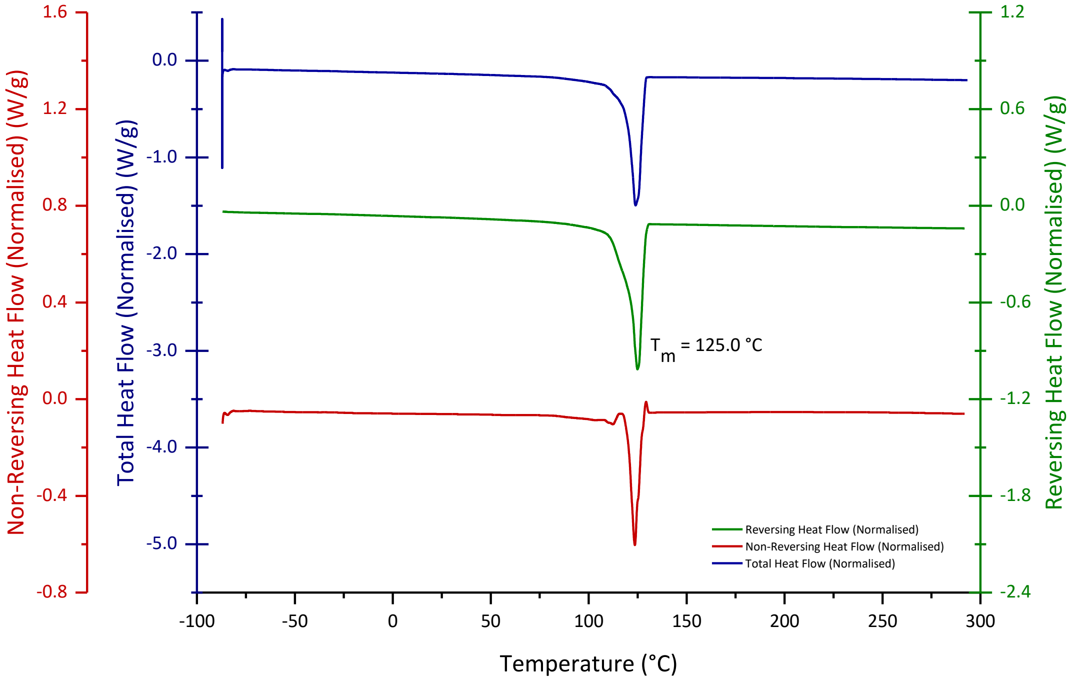

Figure S8: *Differential Scanning Calorimetry (DSC) of synthesized Polyethylene. The Blue trace depicts the normalized total heat flow, the green trace depicts the normalized reversing heat flow, and the red trace depicts the normalized non-reversed heat flow.*

5. Photochemical Oximation of model linear hydrocarbons and polyethylene Data

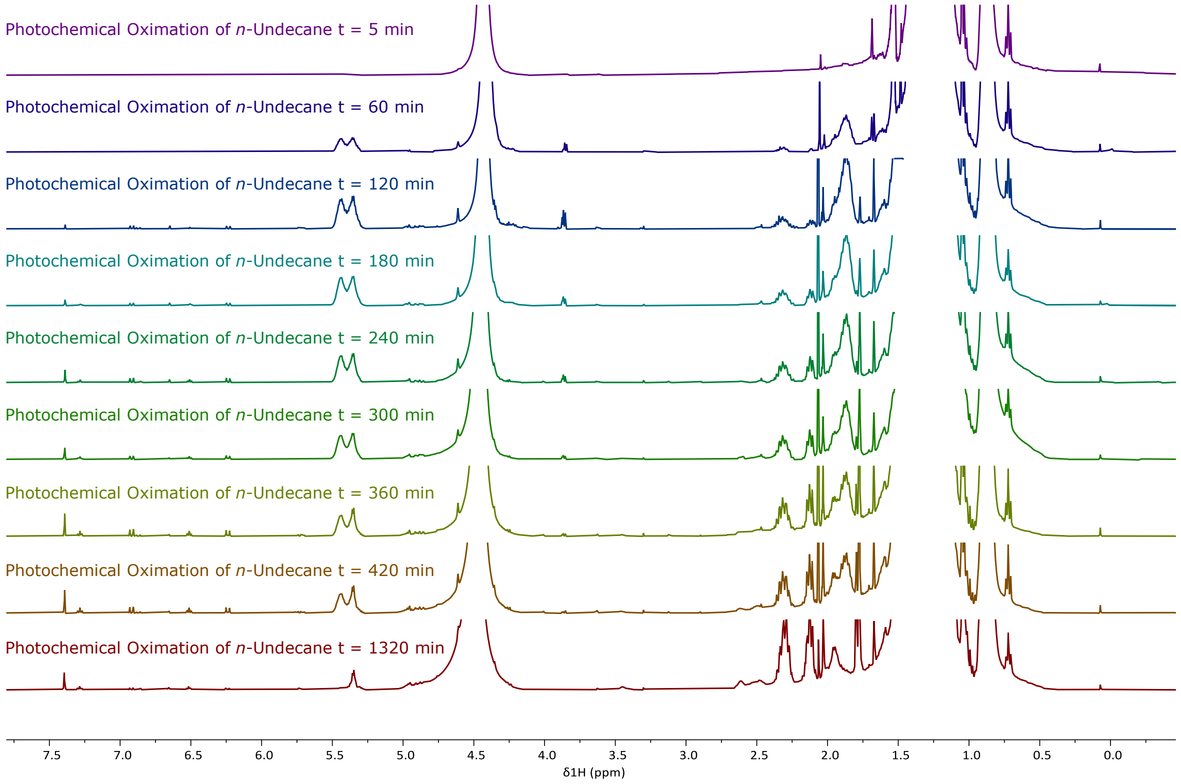

Figure S9: *^{1}H NMR spectra of the photochemical oximation of n-undecane followed over time (no deuterated solvent was added) at 25 °C.*

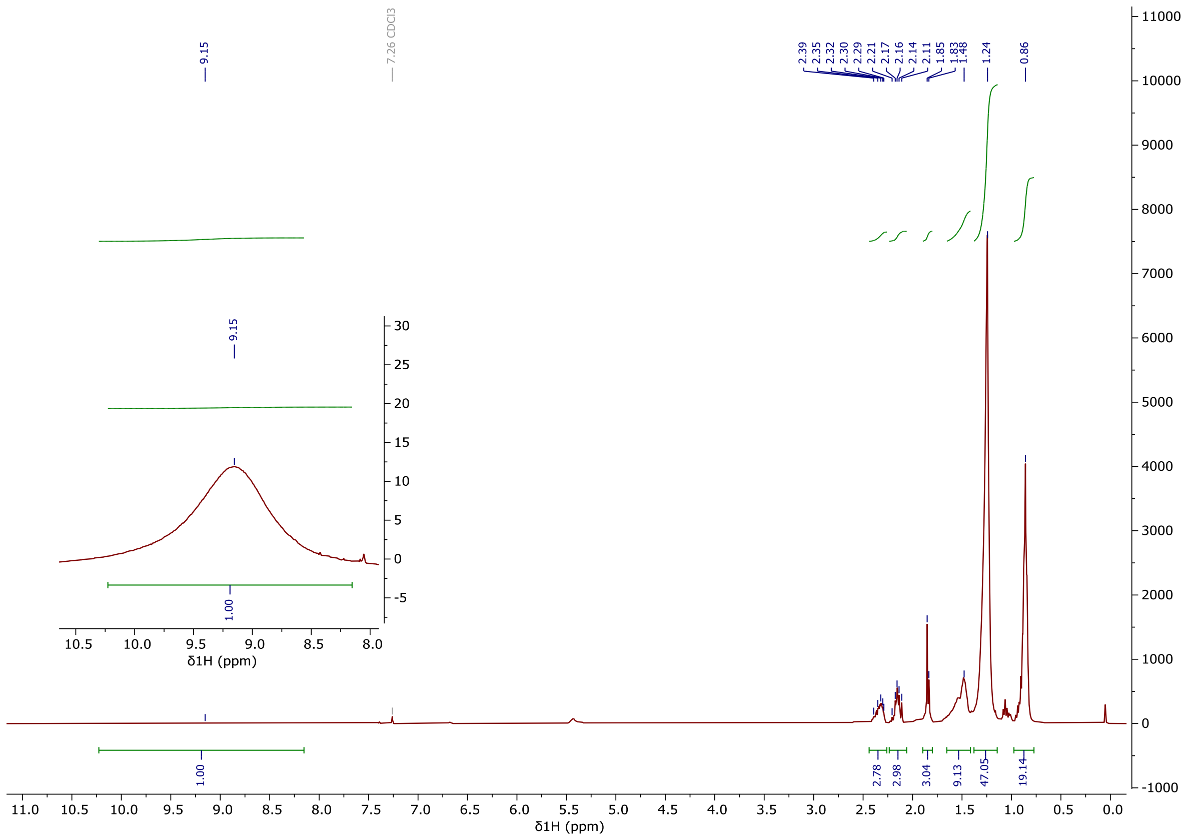

Figure S10: *^{1}H NMR spectrum of the isolated product after the photochemical oximation of n-undecane in $CDCl_3$ at 25 °C.*

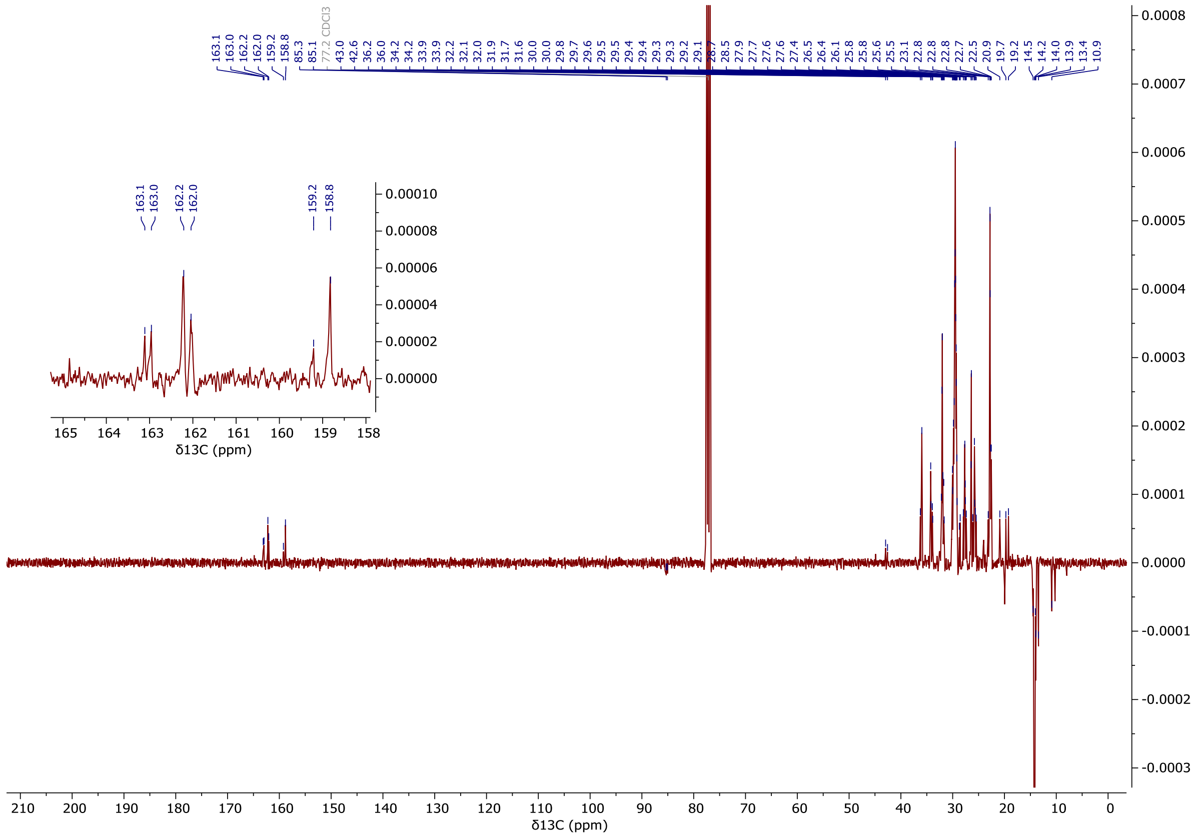

Figure S11: *^{13}C (APT) spectrum of the isolated product after the photochemical oximation of n-undecane in $CDCl_3$ at 25 °C, with a zoom in of the region between 158 ppm and 165 ppm.*

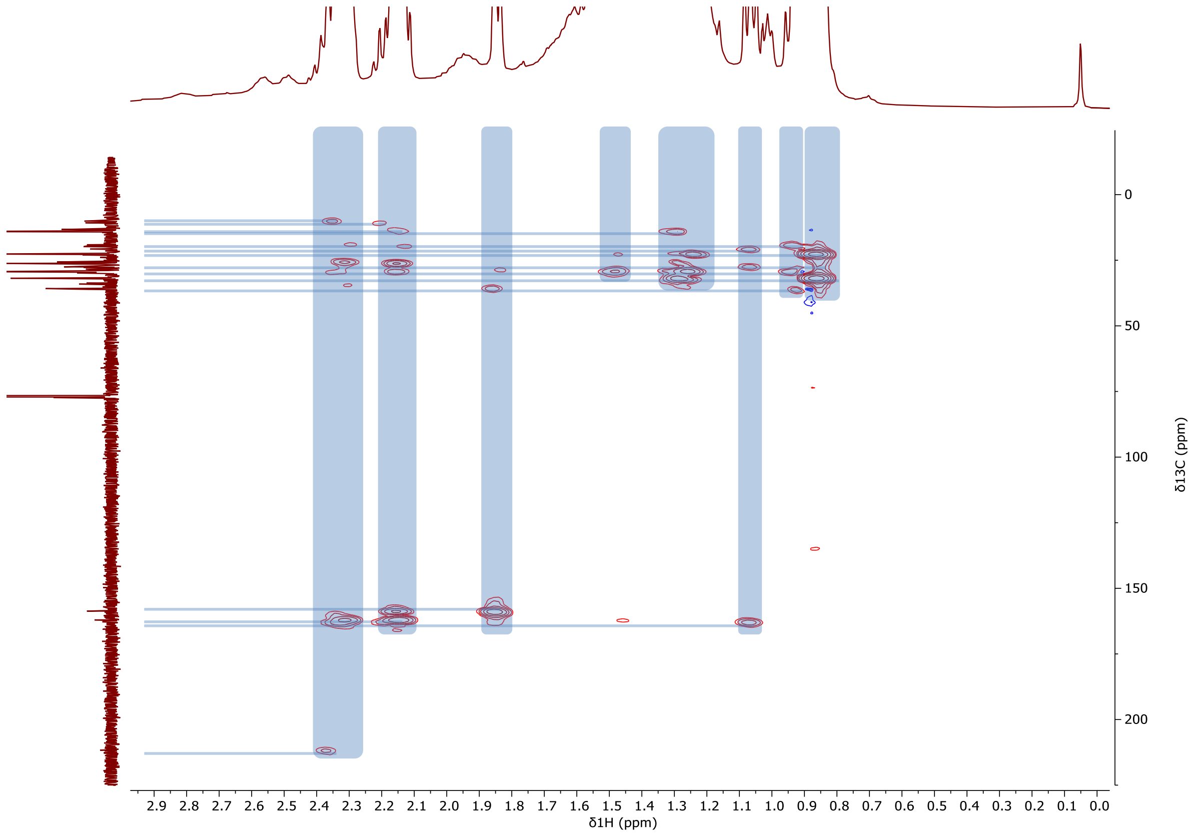

Figure S12: *^{1}H-[13]C Heteronuclear Multiple Bond Correlation (HMBC) spectrum of the isolated product after the photochemical oximation of n-undecane in $CDCl_3$ at 25 °C.*

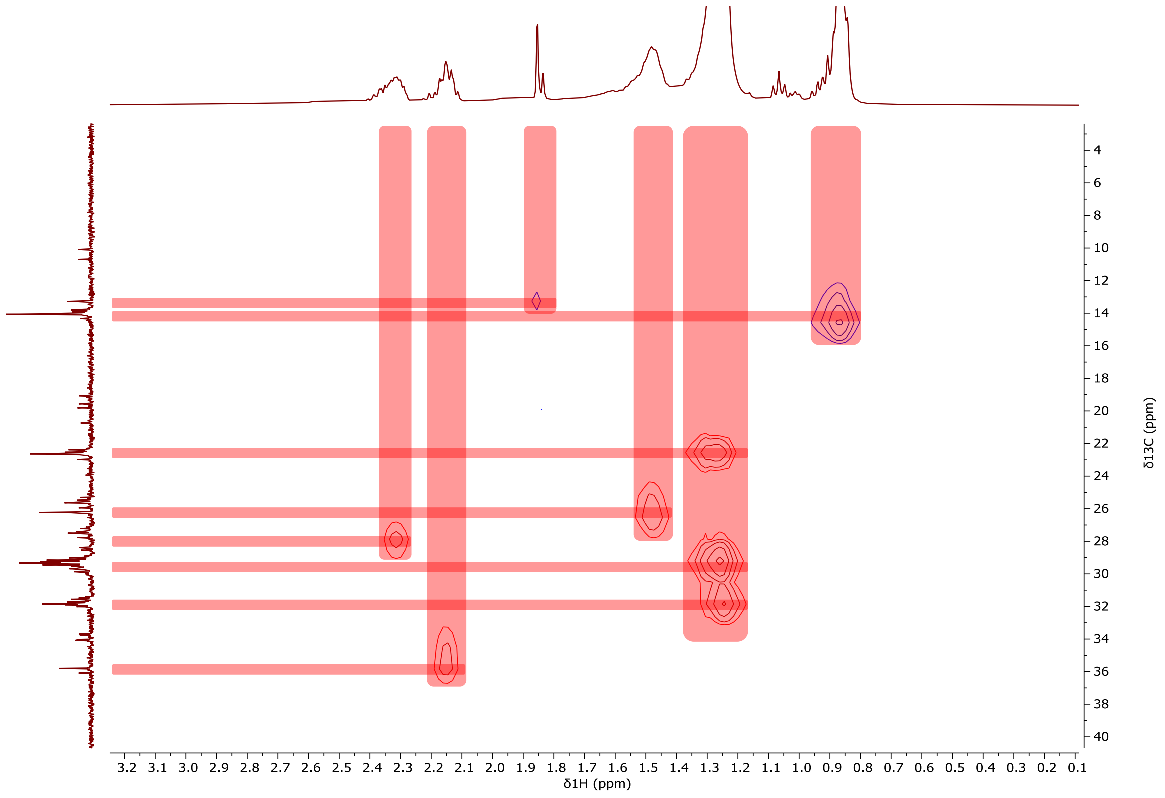

Figure S13: : *^{1}H-[13]C Heteronuclear Single Quantum Coherence (HSQC) spectrum of the isolated product after the photochemical oximation of n-undecane in $CDCl_3$ at 25 °C.*

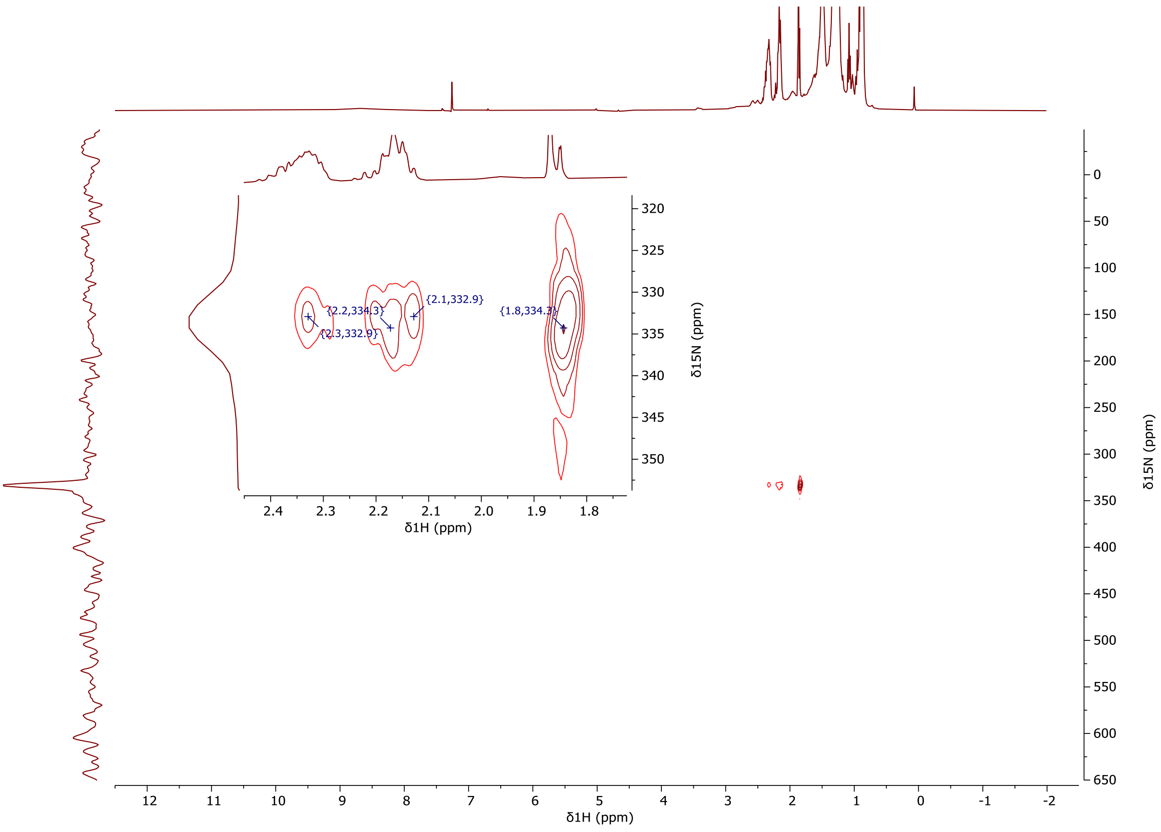

Figure S14: *^{1}H-^{15}N Heteronuclear Multiple Bond Correlation (HMBC) spectrum of spectrum of the isolated product after the photochemical oximation of n-undecane in $CDCl_3$ at 25 °C, with a zoom in of the coupling.*

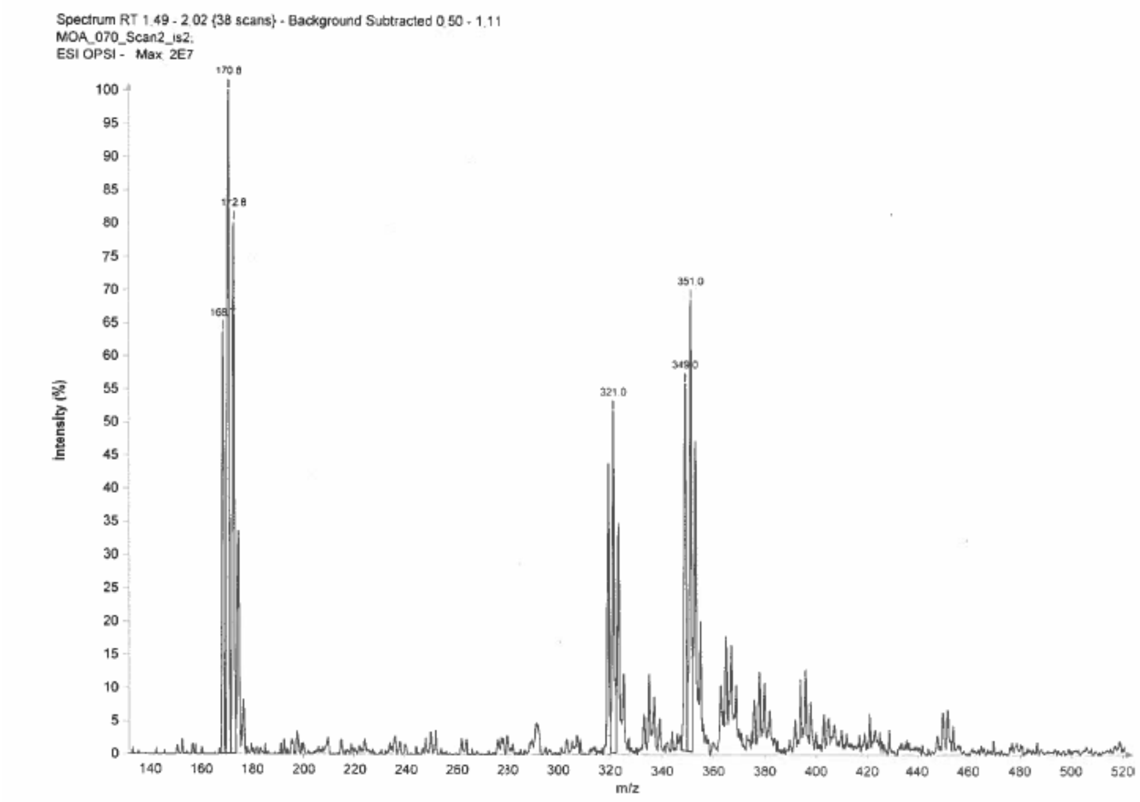

Figure S15: *ESI-MS spectrum of the isolated product after the photochemical oximation of n-undecane, measured in acetonitrile.*

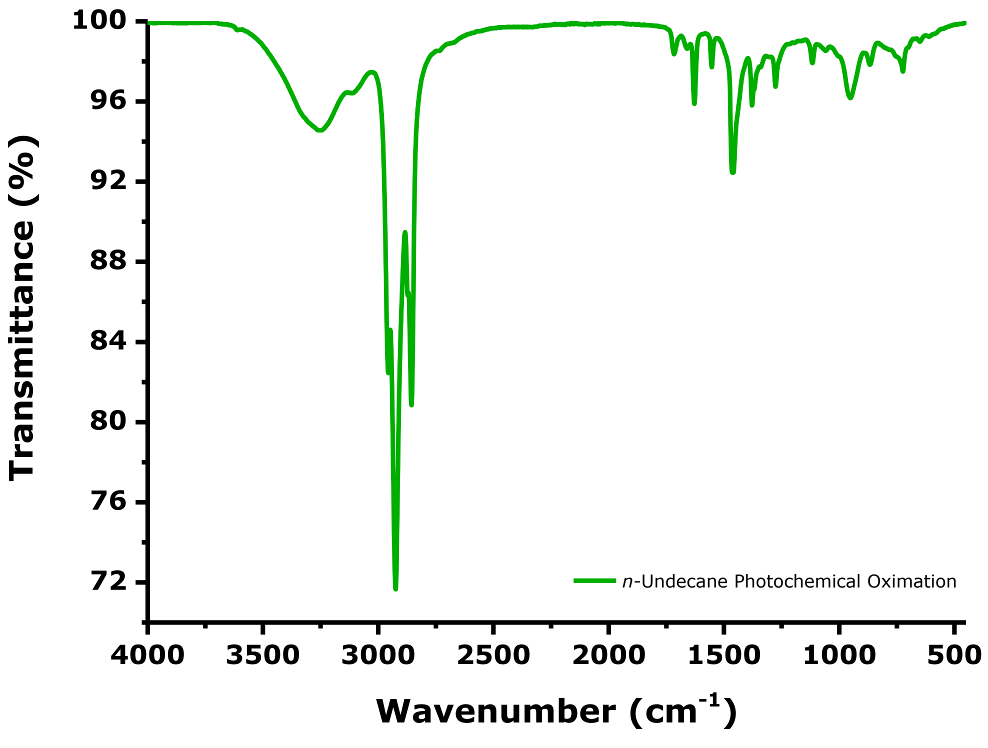

Figure S16: *FTIR (ATR) Spectrum of the isolated product after the photochemical oximation of n-undecane.*

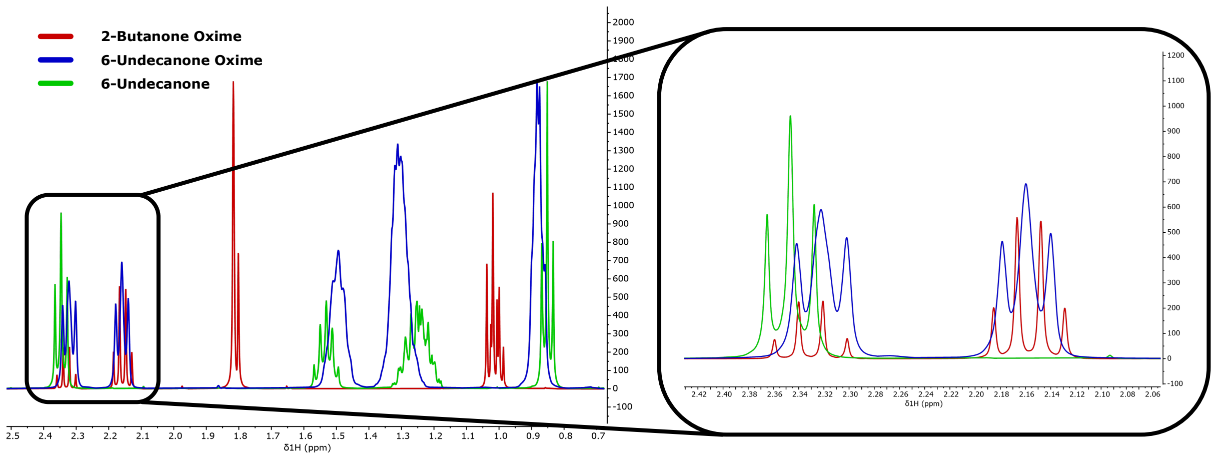

Figure S17: *^{1}H NMR overlay of 2-butanone oxime (red trace), 6-undecanone oxime (blue trace) and 6-undecanone (green trace) to highlight the peak overlap observed in the ^{1}H NMR spectrum in the aliphatic region after the photochemical oximation reaction, all spectra were recorded in $CDCl_3$ at 25 °C.*

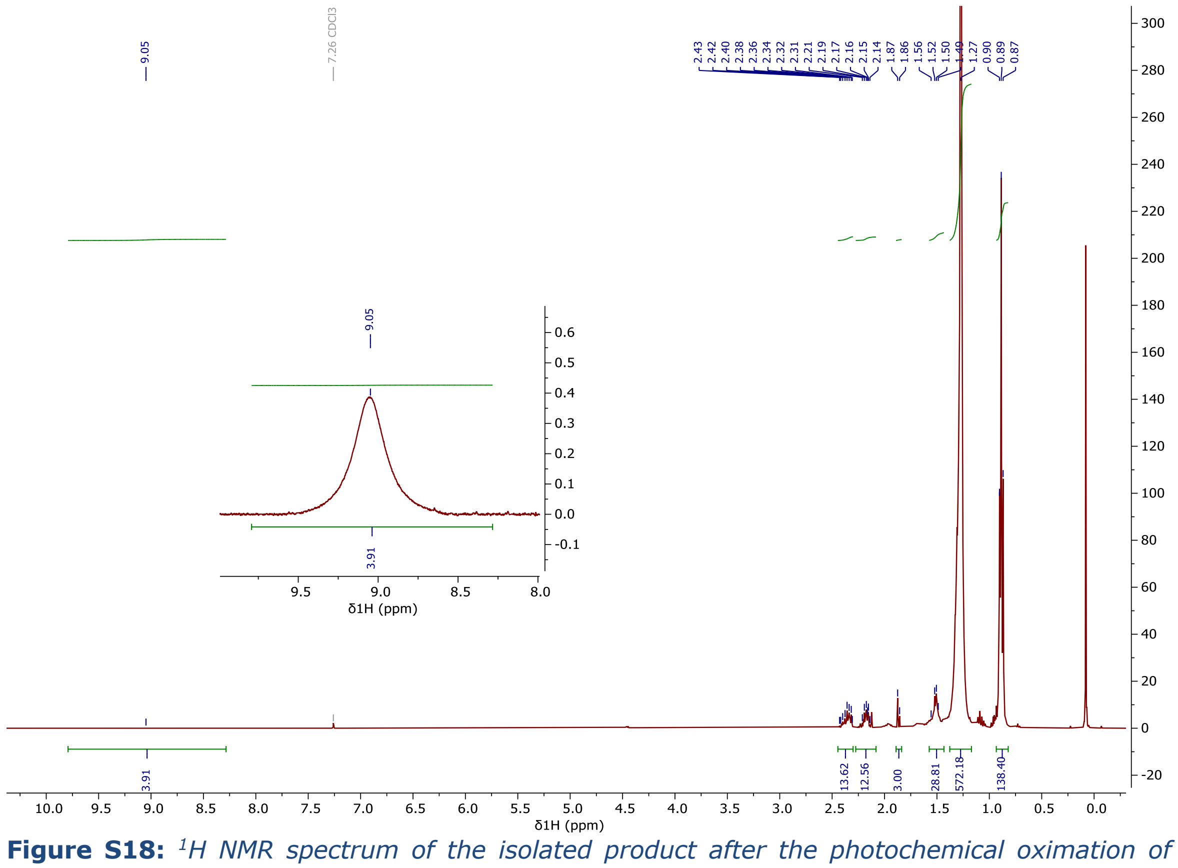

Figure S18: *^{1}H NMR spectrum of the isolated product after the photochemical oximation of n-tetradecane in $CDCl_3$ at 25 °C.*

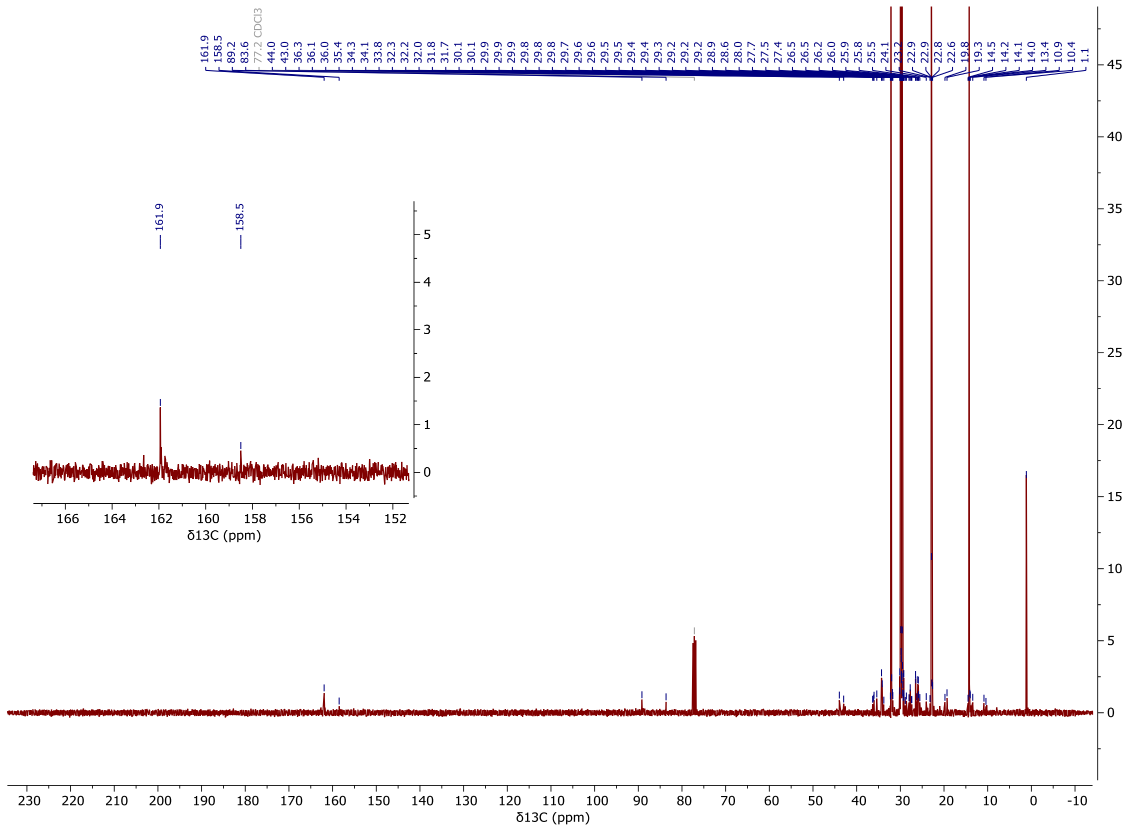

Figure S19: ^{13}C NMR spectrum of the isolated product after the photochemical oximation of n-tetradecane in $CDCl_3$ at 25 °C.

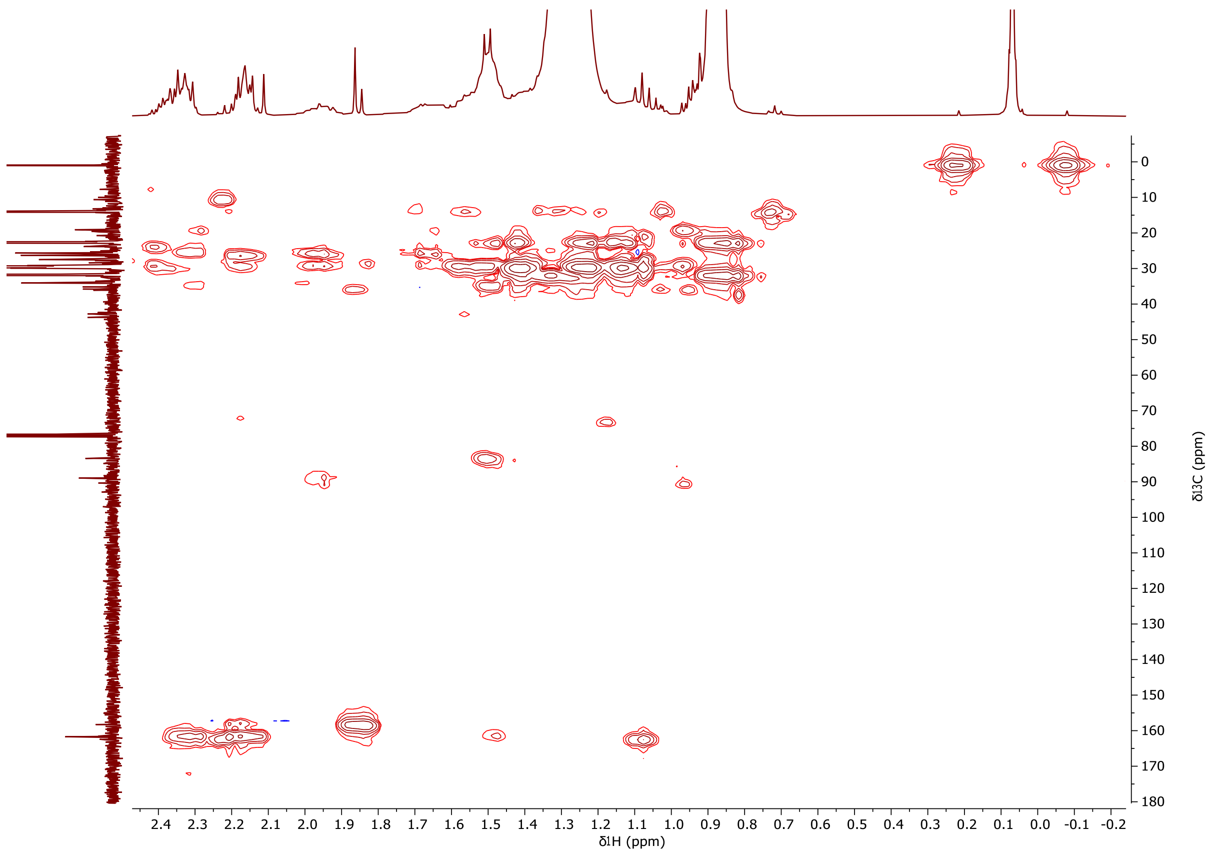

Figure S20: ^{1}H-^{13}C Heteronuclear Multiple Bond Correlation (HMBC) spectrum of the isolated product after the photochemical oximation of n-tetradecane in $CDCl_3$ at 25 °C.

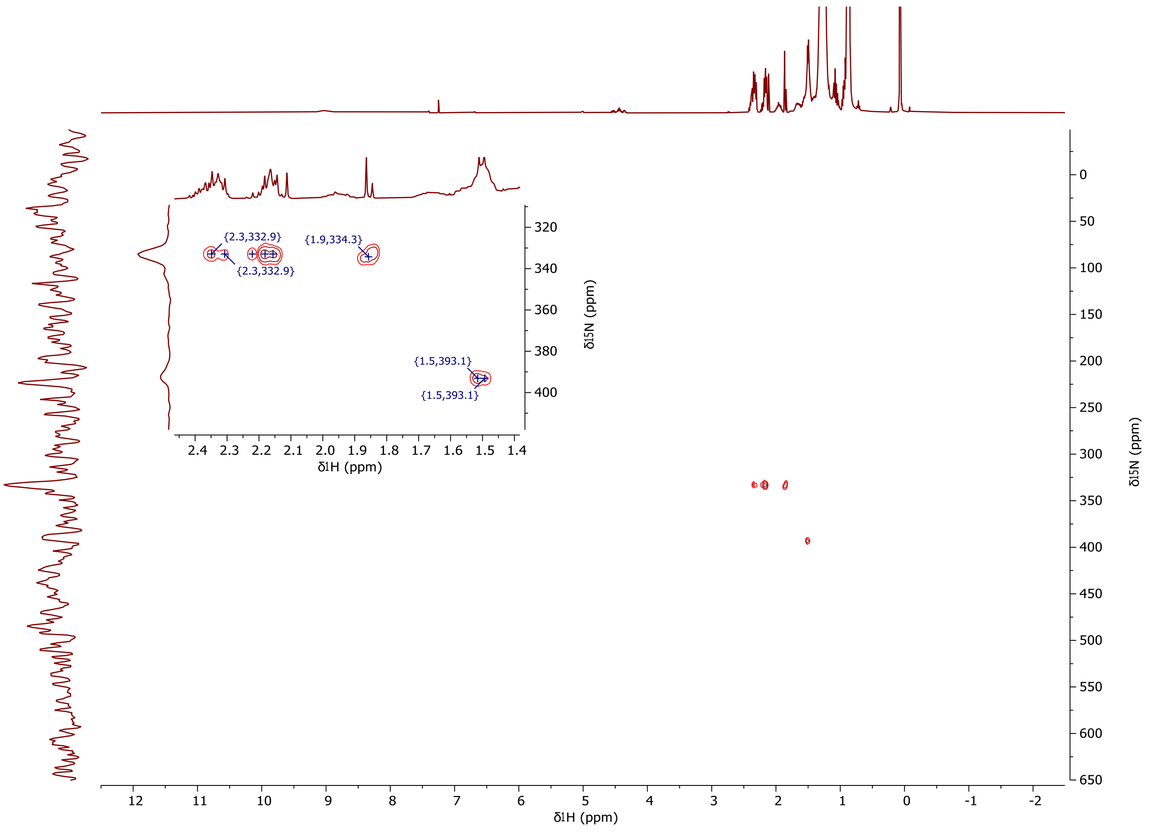

Figure S21: *^{1}H-^{15}N Heteronuclear Multiple Bond Correlation (HMBC) spectrum of the isolated product after the photochemical oximation of n-tetradecane in $CDCl_3$ at 25 °C, with a zoom in on the coupling.*

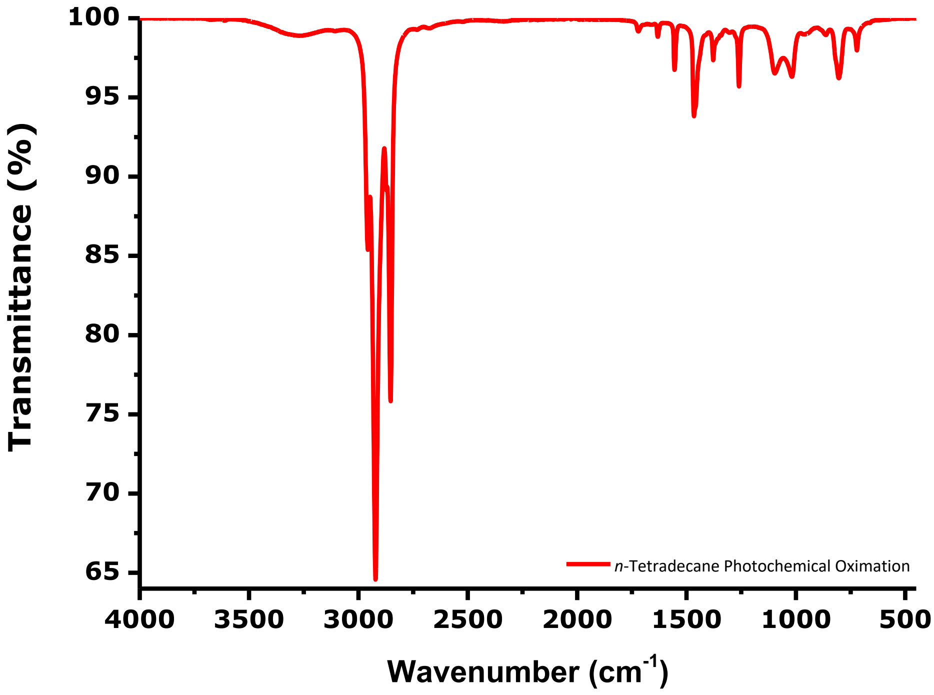

Figure S22: *FTIR (ATR) Spectrum of the isolated product after the photochemical oximation of n-tetradecane.*

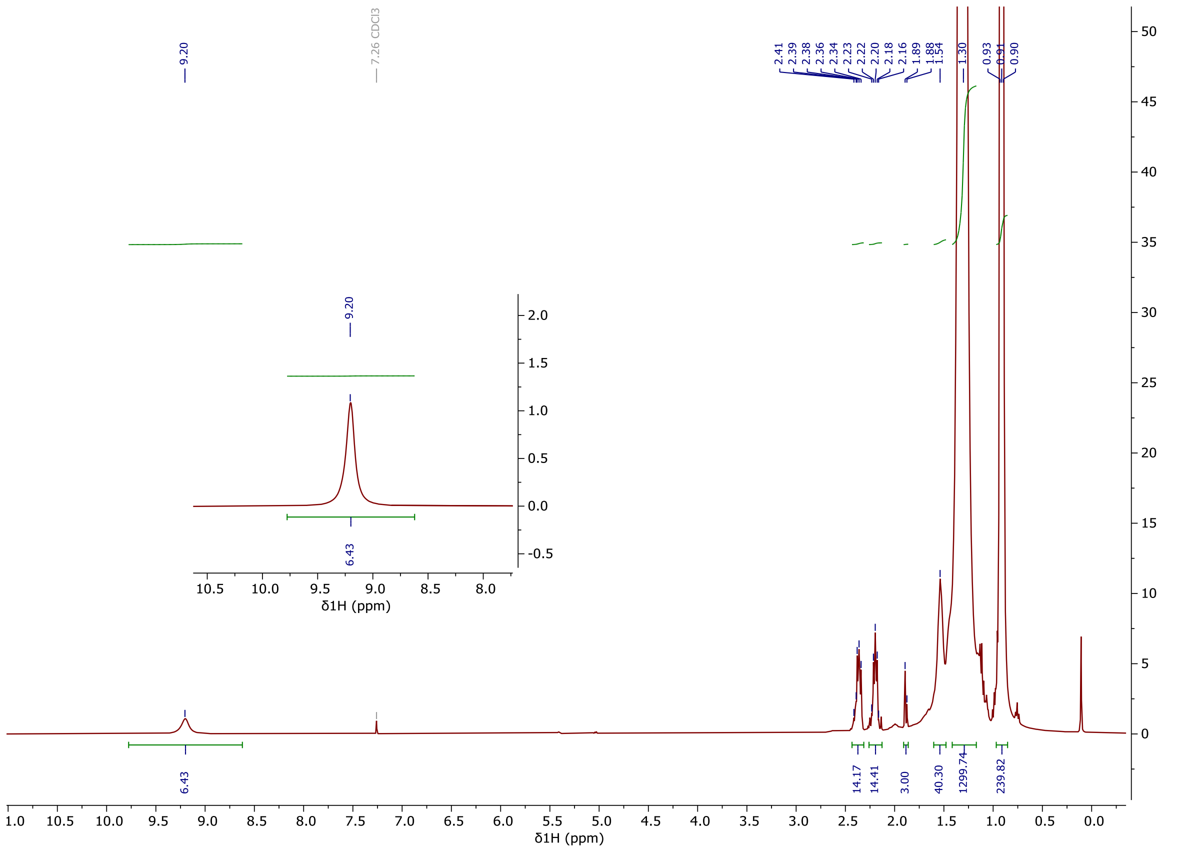

Figure S23: *^{1}H NMR spectrum of the isolated product after the photochemical oximation of n-octadecane in $CDCl_3$ at 25 °C.*

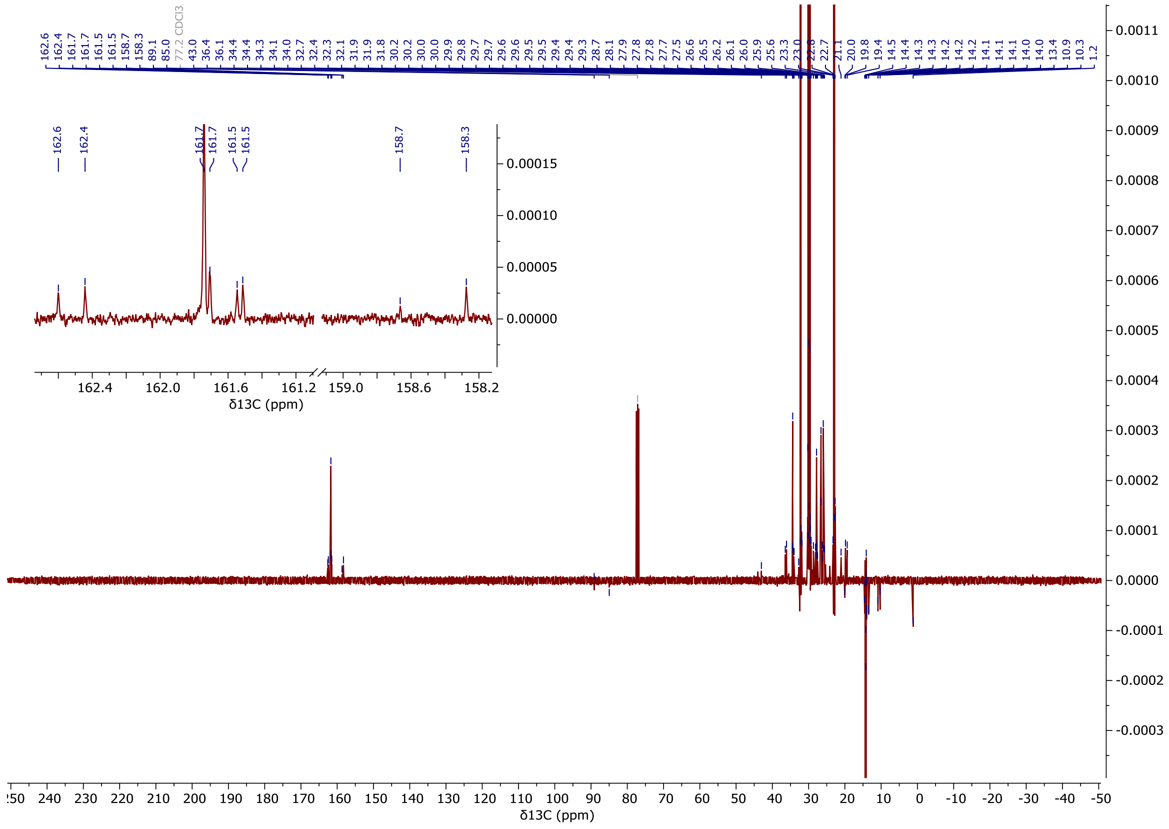

Figure S24: *^{13}C NMR (APT) spectrum of the isolated product after the photochemical oximation of n-octadecane in $CDCl_3$ at 25 °C.*

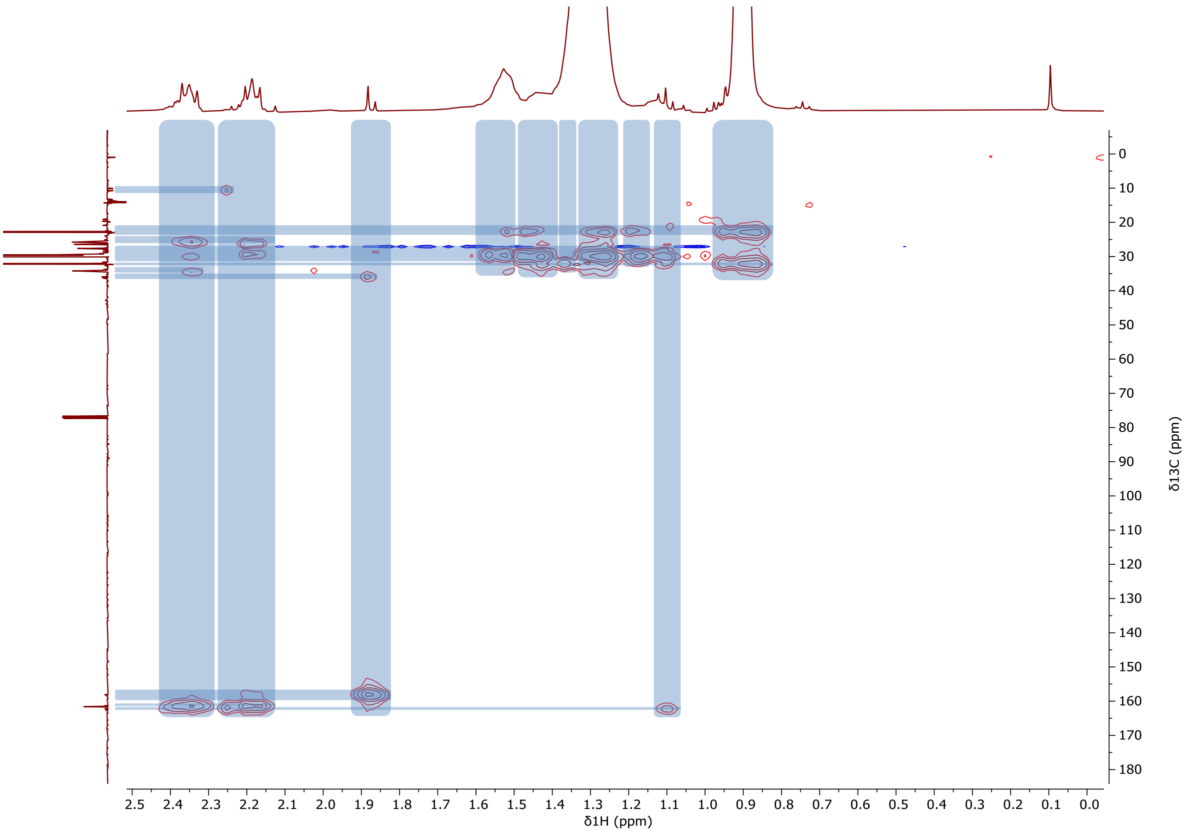

Figure S25: *^{1}H-[13]C Heteronuclear Multiple Bond Correlation (HMBC) spectrum of the isolated product after the photochemical oximation of n-octadecane in $CDCl_3$ at 25 °C.*

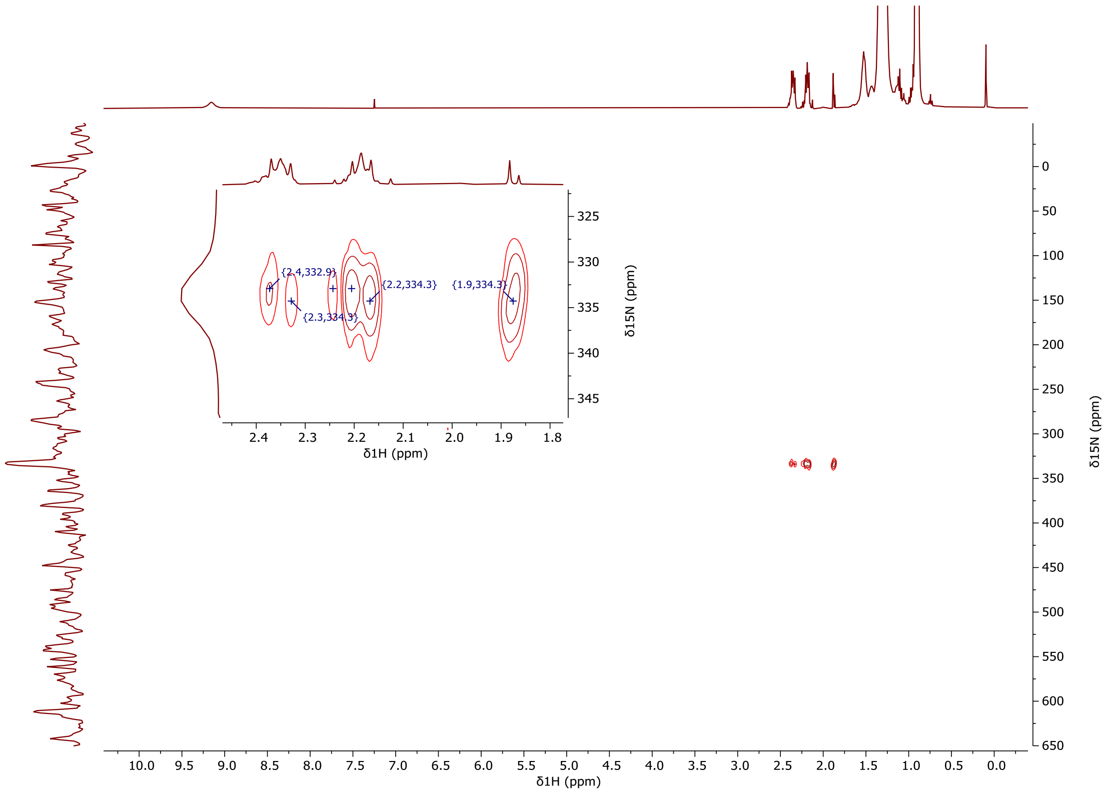

Figure S26: *^{1}H-[15]N Heteronuclear Multiple Bond Correlation (HMBC) spectrum of the isolated product after the photochemical oximation of n-octadecane in $CDCl_3$ at 25 °C, with a zoom in on the coupling.*

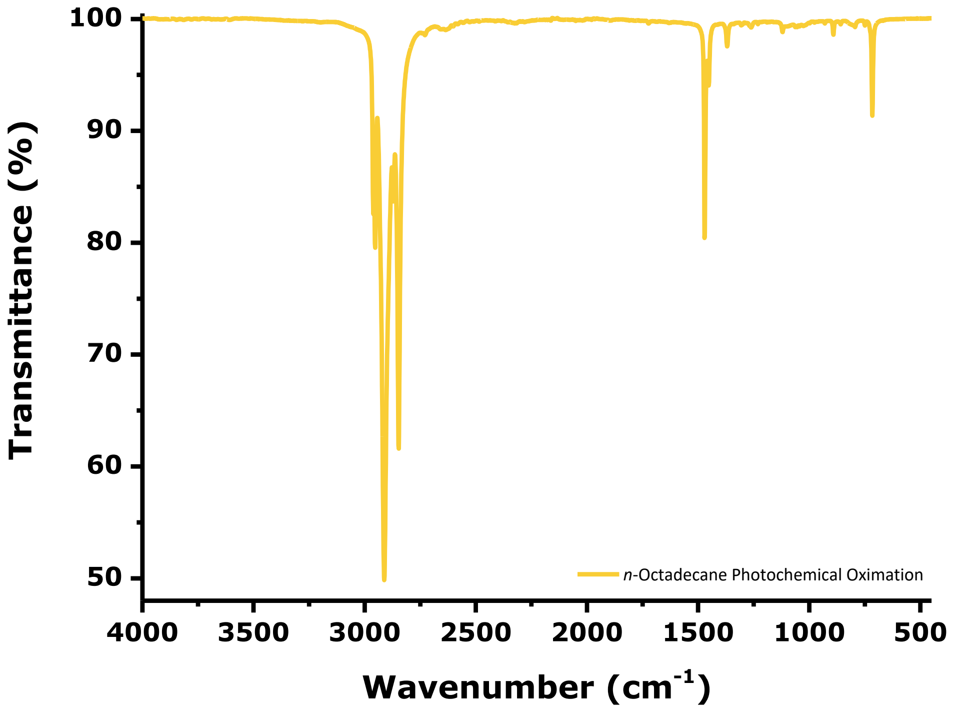

Figure S27: *FTIR (ATR) Spectrum of the isolated product after the photochemical oximation of n-octadecane.*

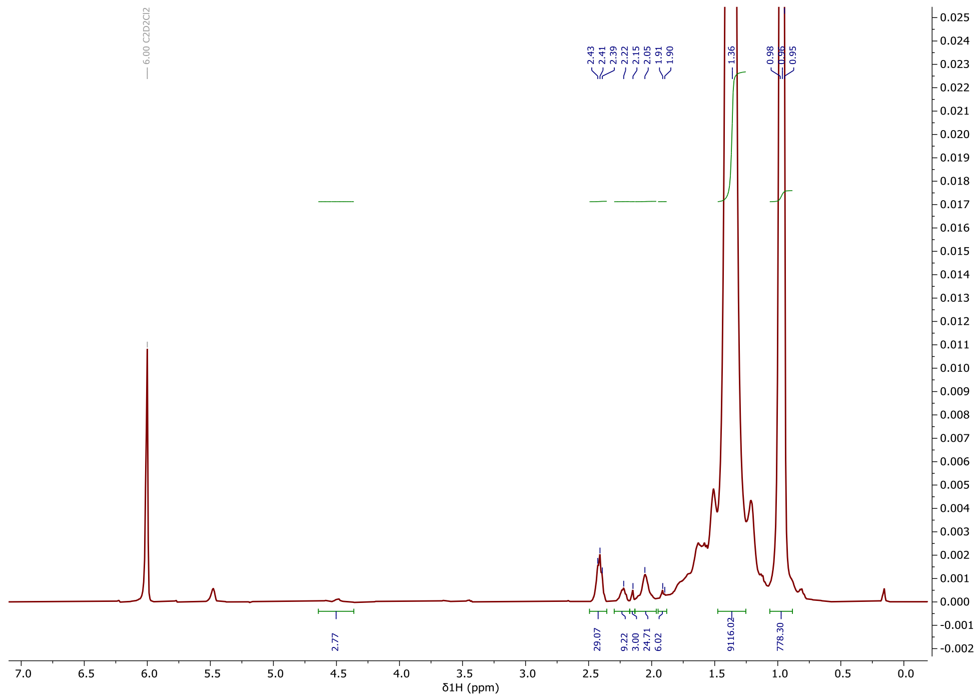

Figure S28: *^{1}H NMR Spectrum of the isolated product after the photochemical oximation of n-hexatriacontane in $C_2D_2Cl_4$ at 120 °C.*

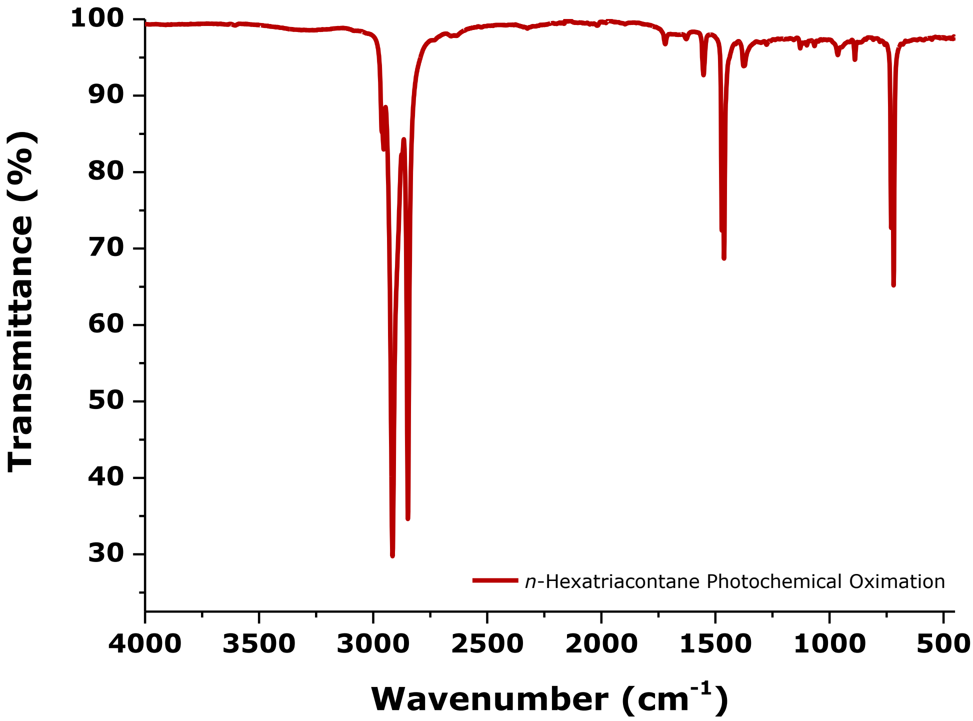

Figure S29: *FTIR (ATR) Spectrum of the isolated product after the photochemical oximation of n-hexatriacontane.*

PE-S (Table 2, Entry 1)

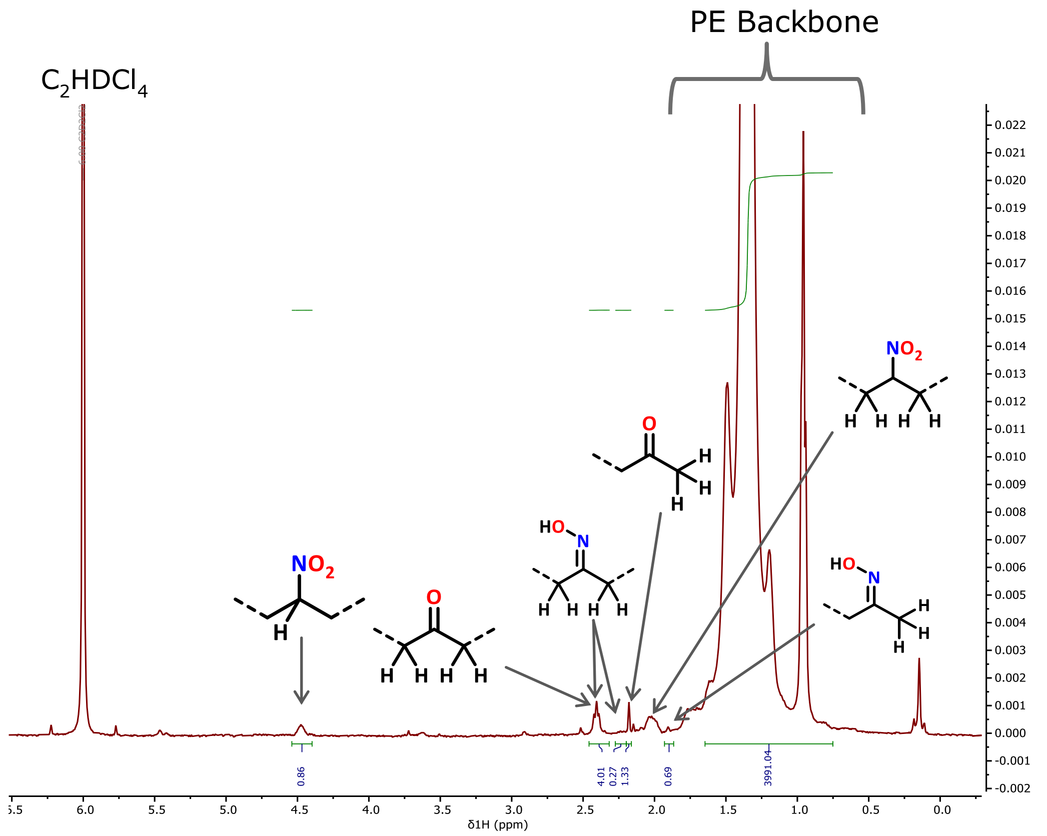

Figure S30: *^{1}H NMR Spectrum of the isolated product after the photochemical oximation of Self-Synthesized PE (PE-S) in $C_2D_2Cl_4$ at 120 °C.*

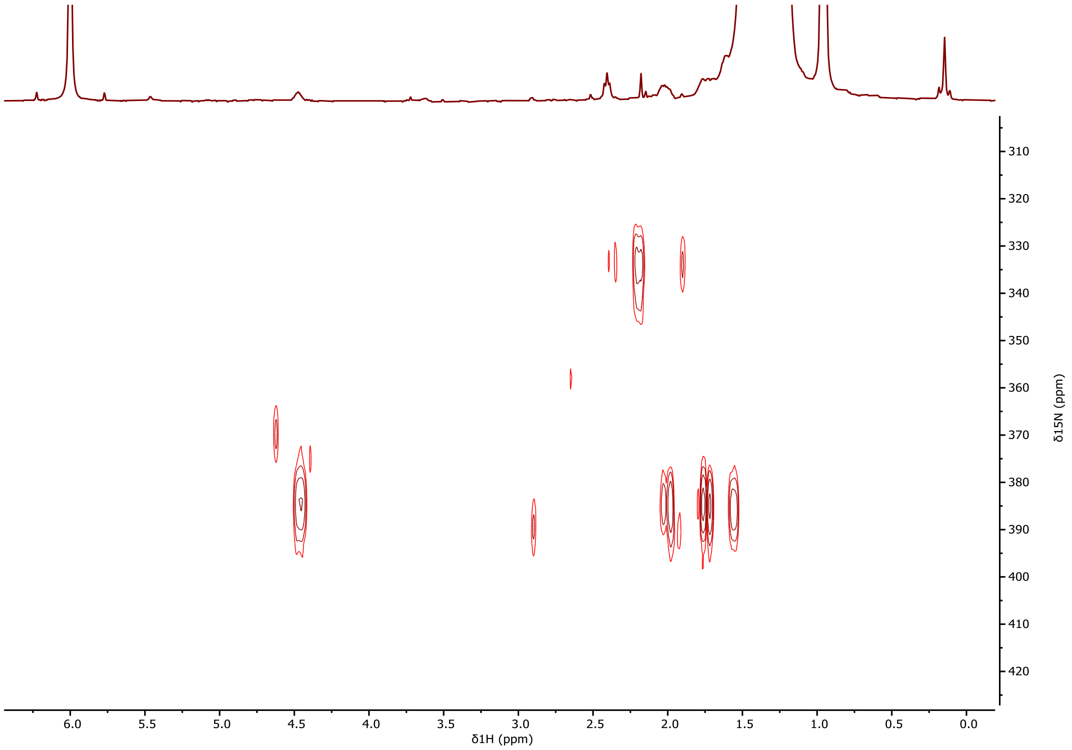

Figure S31: *^{1}H-[15]N Heteronuclear Multiple Bond Correlation (HMBC) spectra zoomed in on the oxime and nitro peaks of the isolated product after the photochemical oximation of Self-Synthesized Polyethylene (PE-S) in $C_2D_2Cl_4$ at 120 °C.*

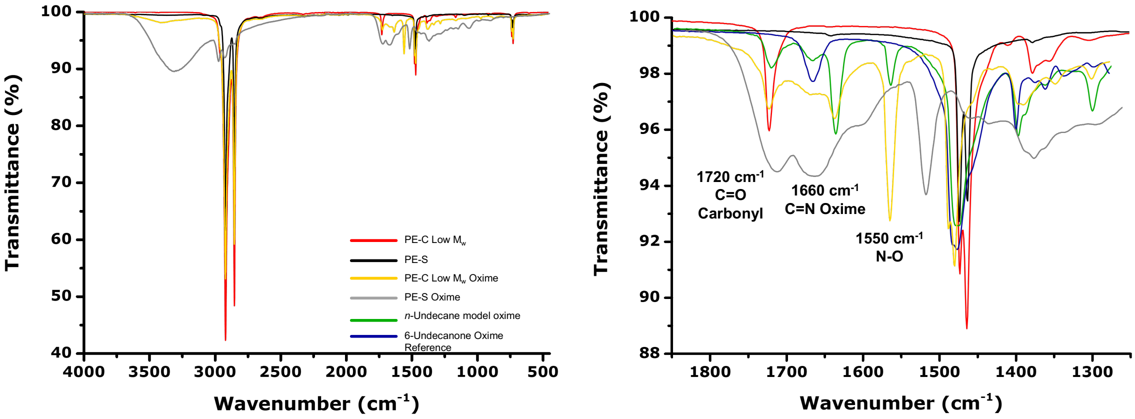

Figure S32: *FTIR (ATR) Spectrum of the isolated polyethylene products (PE-C Low M_w) and (PE-S) after the photochemical oximation and before photochemical oximation (left). On the right hand side is a zoom in with also two reference compounds overlaying.*

HDPE-C (Table 2, Entry 2)

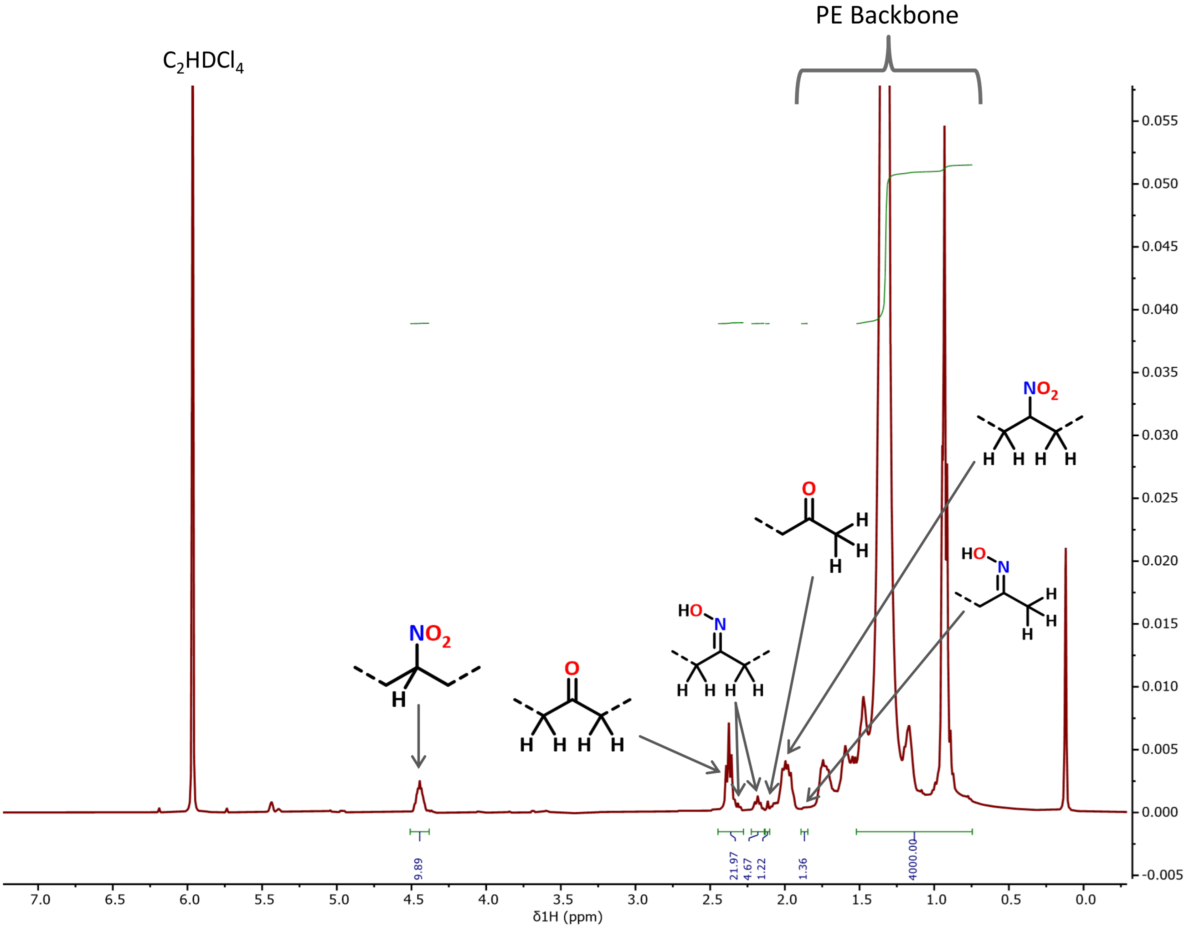

Figure S33: *^{1}H NMR Spectrum of the isolated product after the photochemical oximation of HPDE-C in 1,1,2,2-tetrachloroethane, measured in $C_2D_2Cl_4$ at 120 °C.*

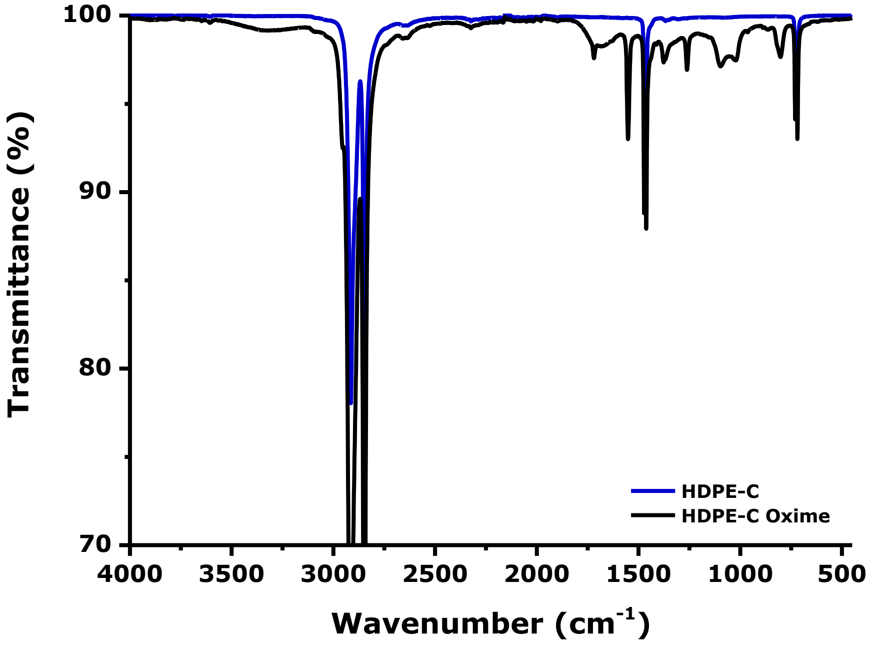

Figure S34: *FTIR (ATR) Spectrum of the isolated product after the photochemical oximation and before photochemical oximation of HDPE (HDPE-C).*

PE-C Low M_w (Table 2, Entry 4)

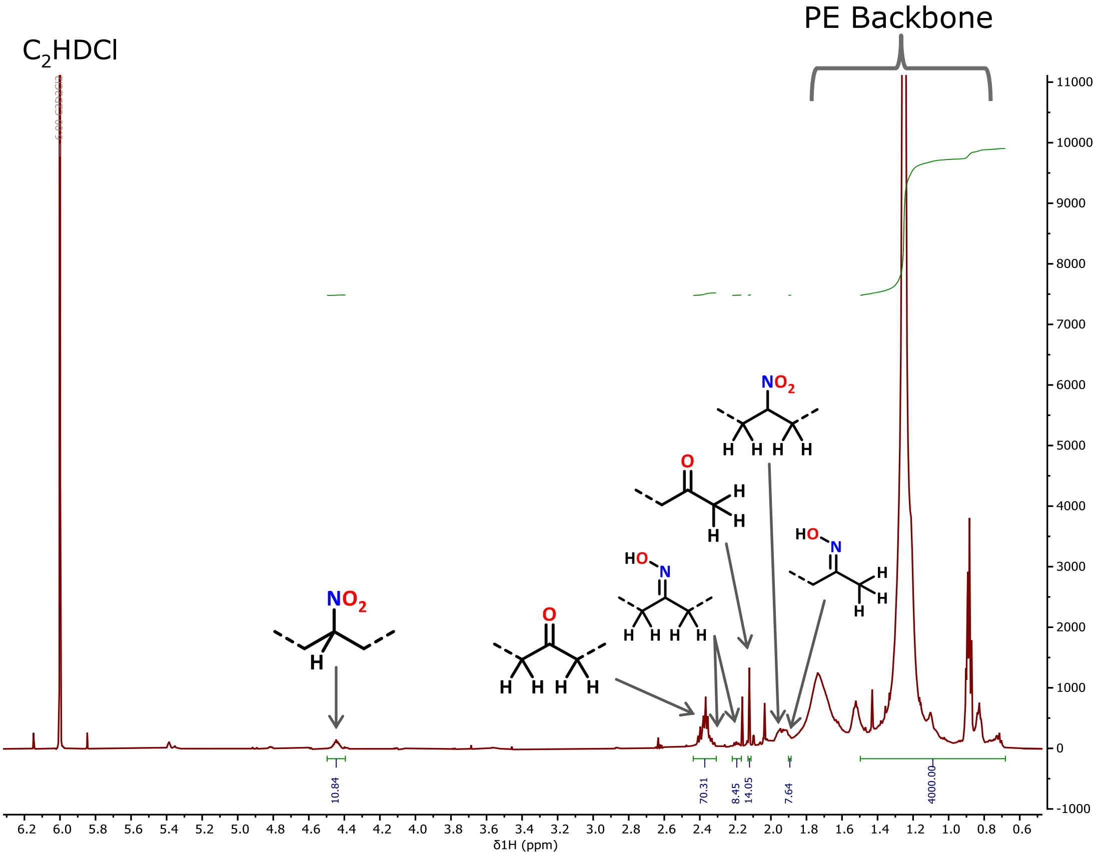

Figure S35: *^{1}H NMR Spectrum of the isolated product after the photochemical oximation Commercial Vendor PE (PE-C) in $C_2D_2Cl_4$ at 120 °C.*

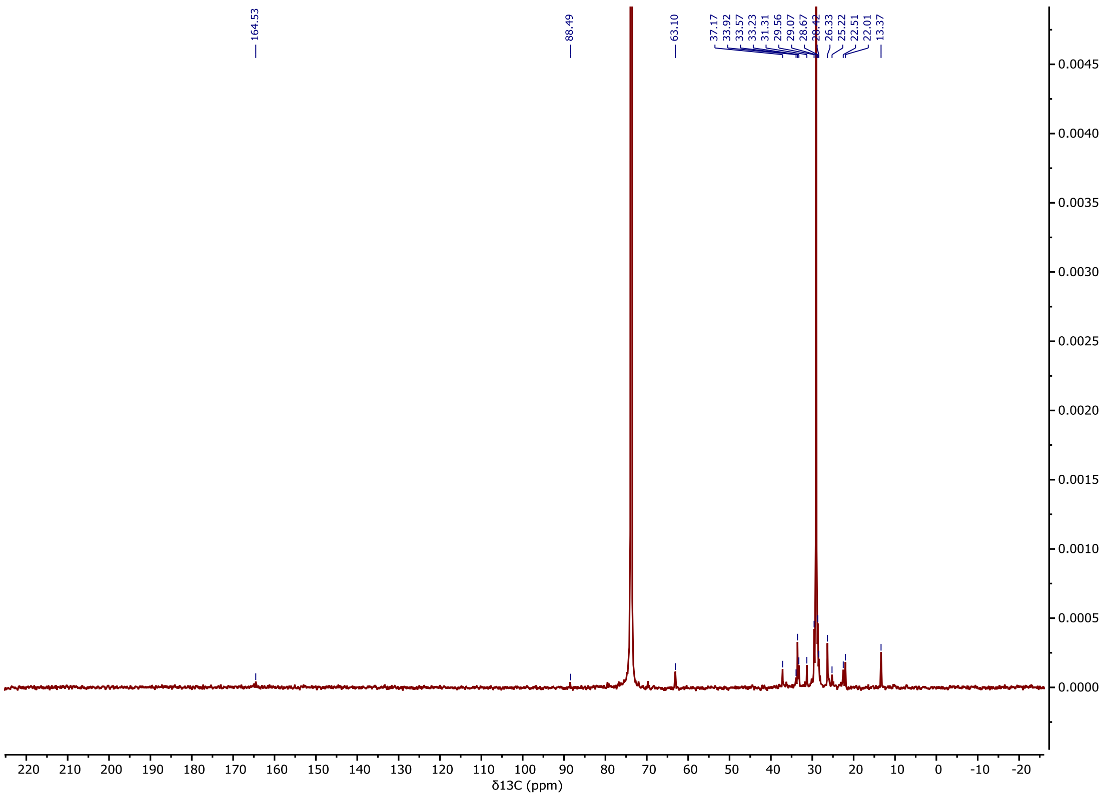

Figure S36: *^{13}C NMR Spectrum of the isolated product after the photochemical oximation Commercial Vendor PE (PE-C Low M_w) in $C_2D_2Cl_4$ at 120 °C.*

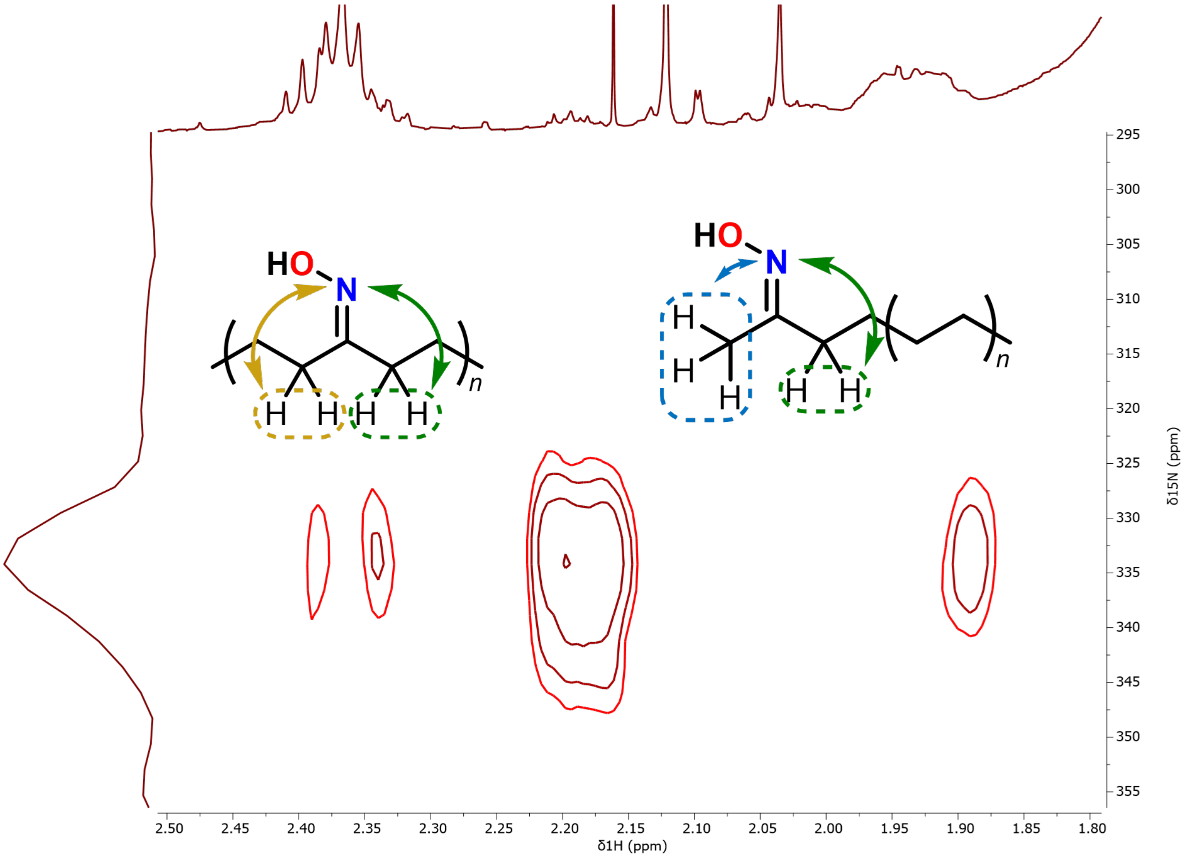

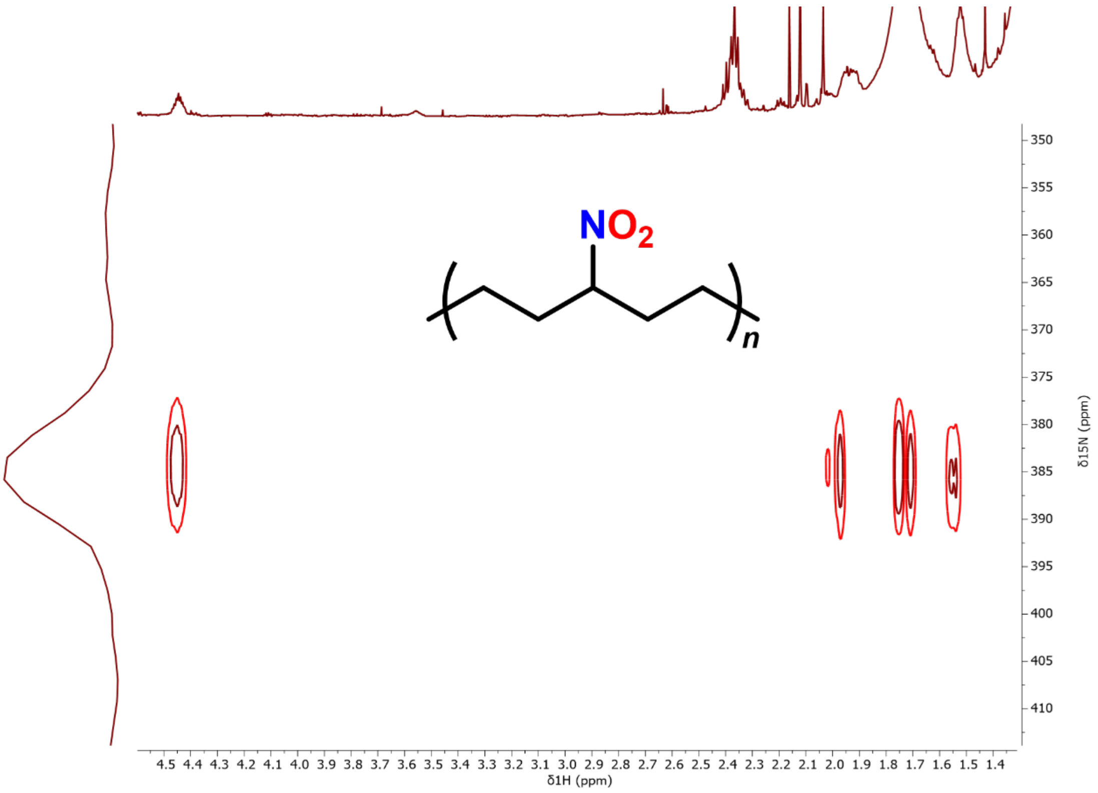

Figure S37: *^1H-^{15}N Heteronuclear Multiple Bond Correlation (HMBC) spectra zoomed in on the oxime and nitro peaks of the isolated product after the photochemical oximation of (PE-C Low M_w) in $C_2D_2Cl_4$ at 120 °C.*

HDPE-C High M_w (Table 2, Entry 4)

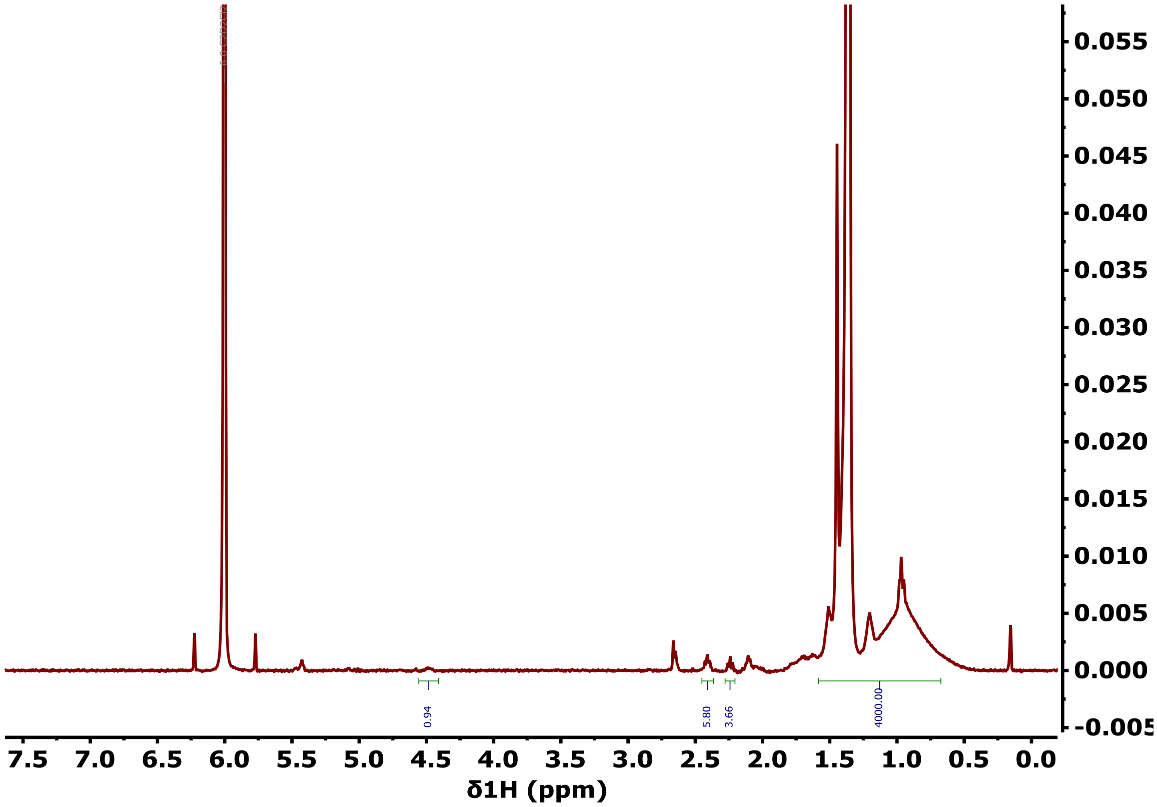

Figure S38: *^1H NMR Spectrum of the isolated product after the photochemical oximation HDPE-C High M_w in $C_2D_2Cl_4$ at 120 °C.*

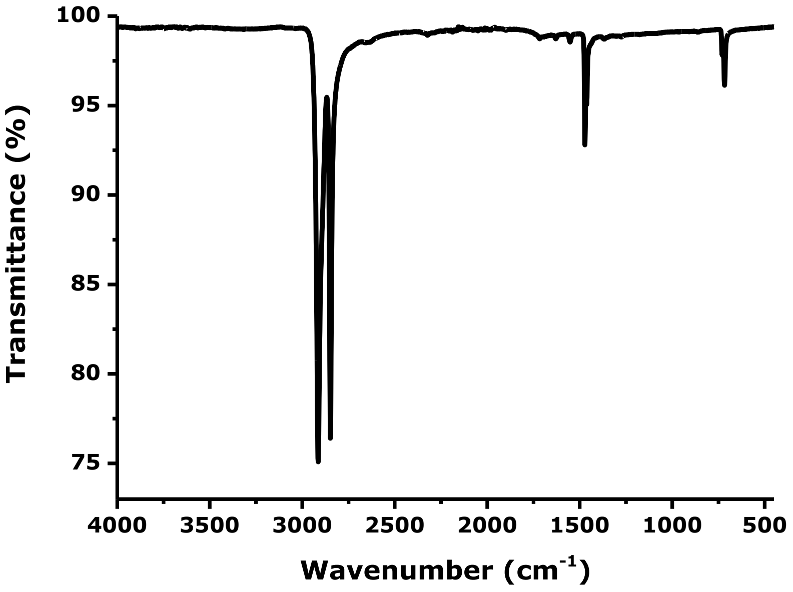

Figure S39: *FTIR (ATR) Spectrum of the isolated product after the photochemical oximation of HDPE (HDPE-C High M_w).*

PE-P (Table 2, Entry 5)

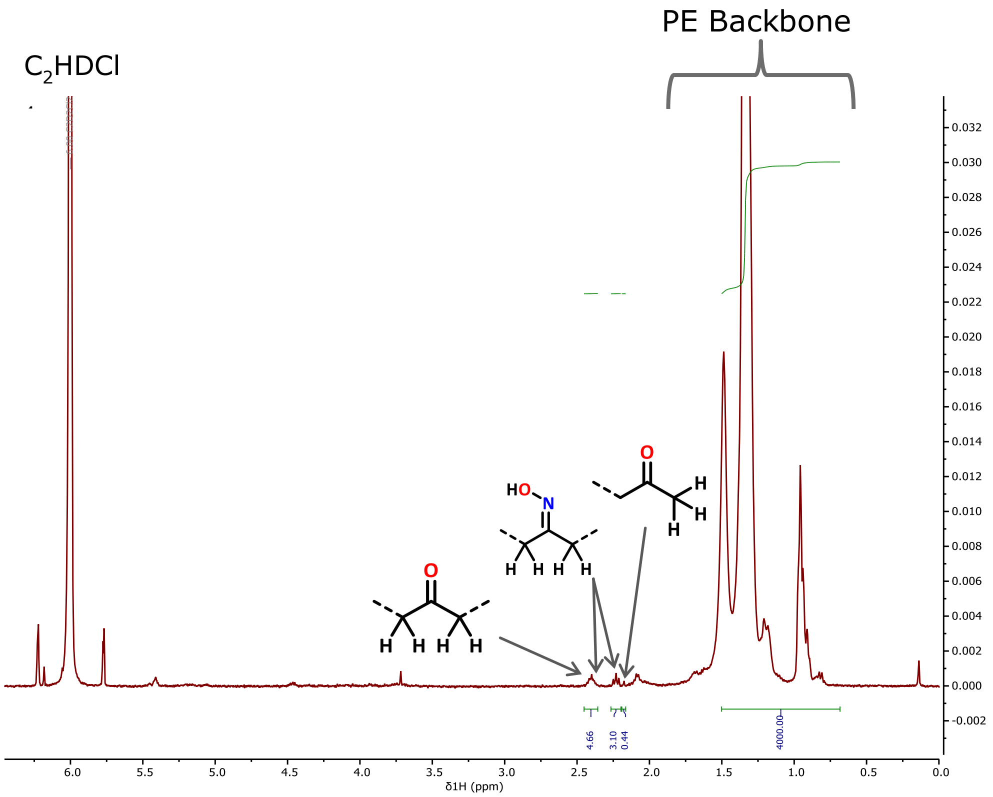

Figure S40: *^1H NMR Spectrum of the isolated product after the photochemical oximation of Post Consumer PE (PE-P) in $C_2D_2Cl_4$ at 120 °C.*

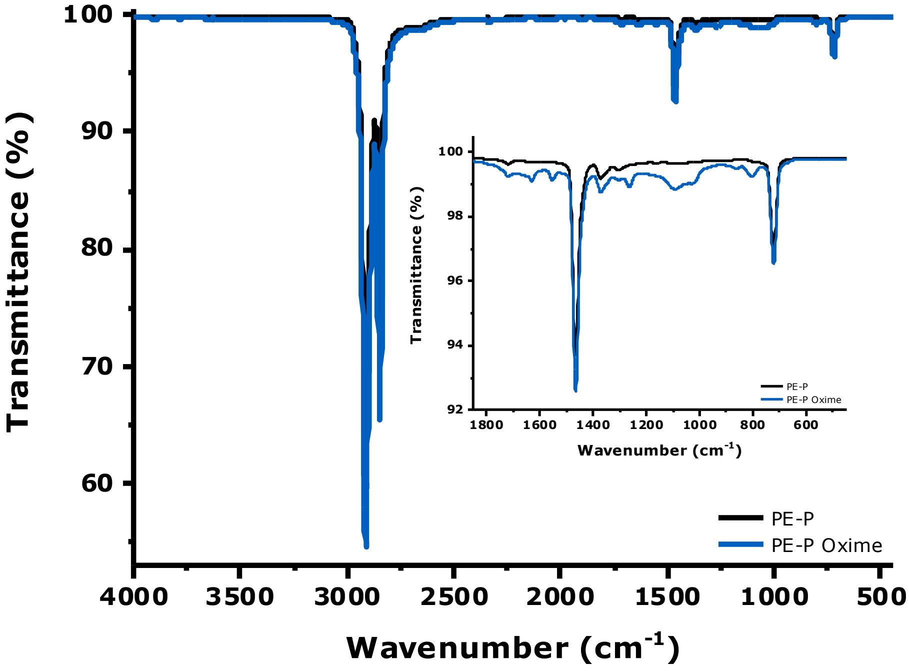

Figure S41: *FTIR (ATR) Spectrum of the isolated product after the photochemical oximation and before photochemical oximation of post consumer polyethylene (PE-P) with a zoom in between 1900 cm^{-1} and 450 cm^{-1}.*

Photochemical reaction in 1,1,2,2-tetrachloroethane (Table 2, Entry 11)

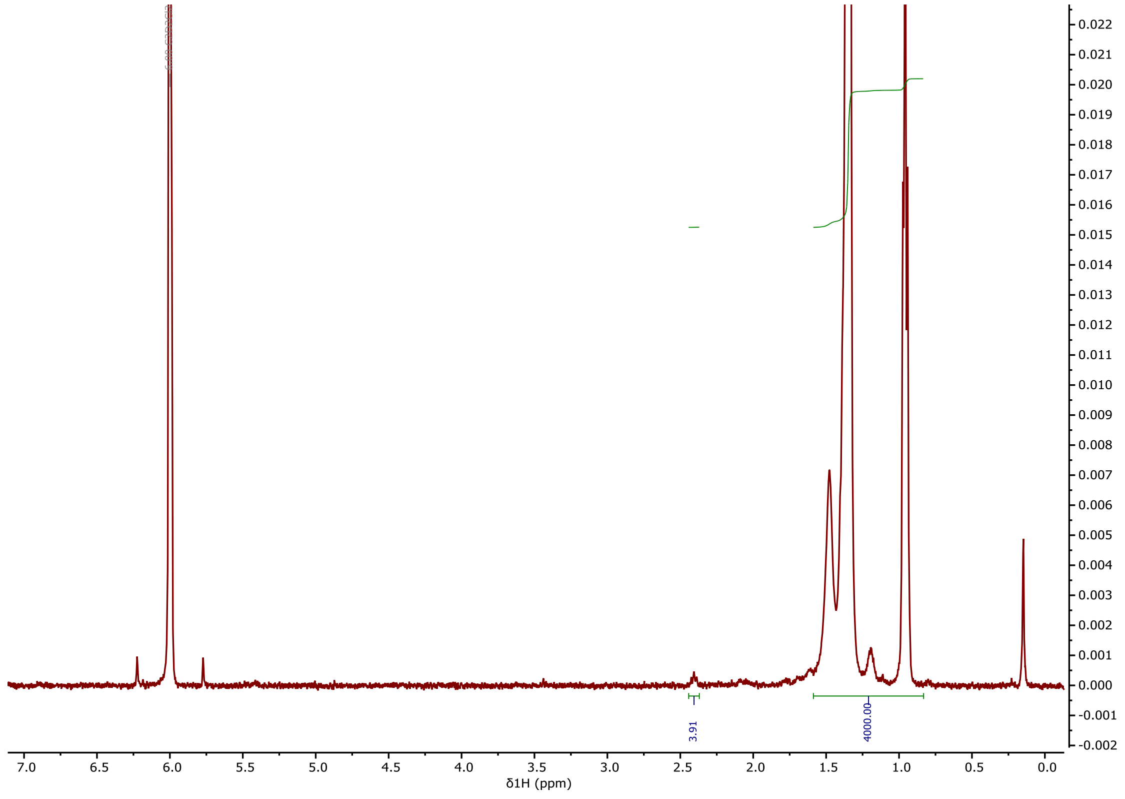

Figure S42: *^{1}H NMR Spectrum of the isolated product after the photochemical oximation of n-hexatriacontane in 1,1,2,2-tetrachloroethane, measured in $C_2D_2Cl_4$ at 120 °C.*

Photochemical reaction in Toluene (Table 2, Entry 12)

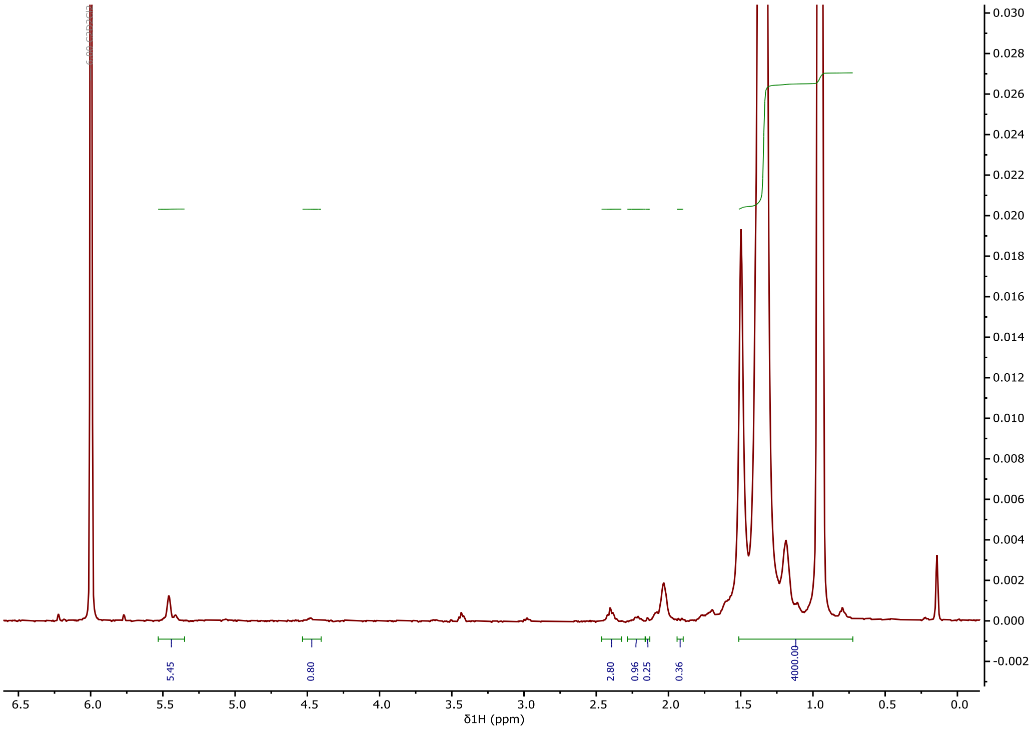

Figure S43: *^{1}H NMR Spectrum of the isolated product after the photochemical oximation of n-hexatriacontane in toluene, measured in $C_2D_2Cl_4$ at 120 °C.*

Photochemical reaction with Acetic Acid

The addition of 1.0 equiv. (relative to *t*-BuONO) of acetic acid (AcOH) as a proton source to reprotonate the oxime decreased the formation of the ketone on the backbone, but simultaneously increased the nitro functional groups, most likely as a result of an oxidation reaction of the oxime species in the presence of AcOH.[14]

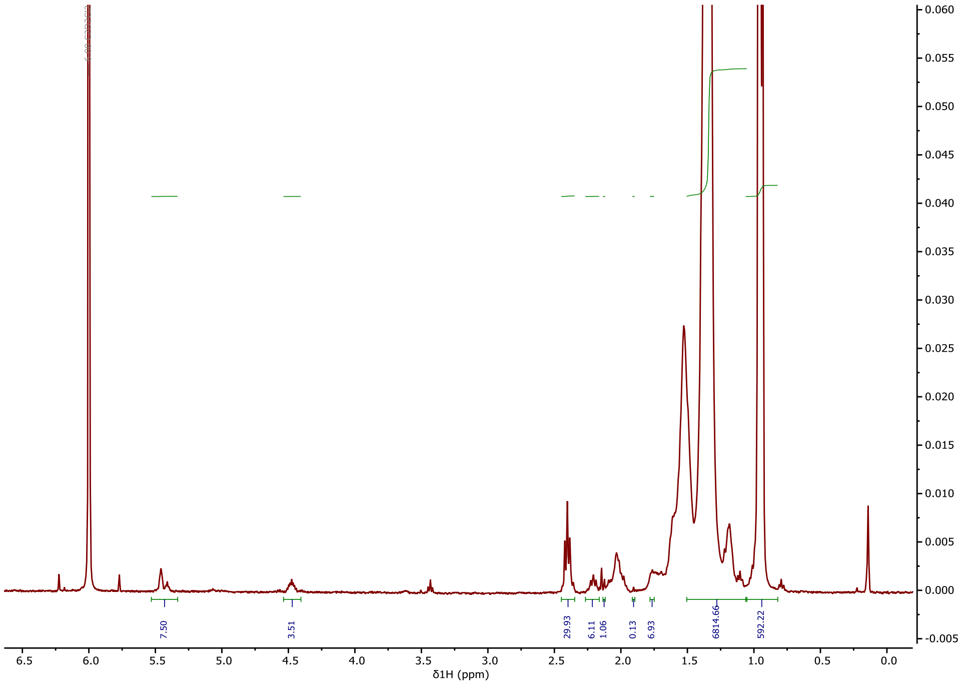

Figure S44: *^{1}H NMR Spectrum of the isolated product after the photochemical oximation of n-hexatriacontane with Acetic Acid in $C_2D_2Cl_4$ at 120 °C.*

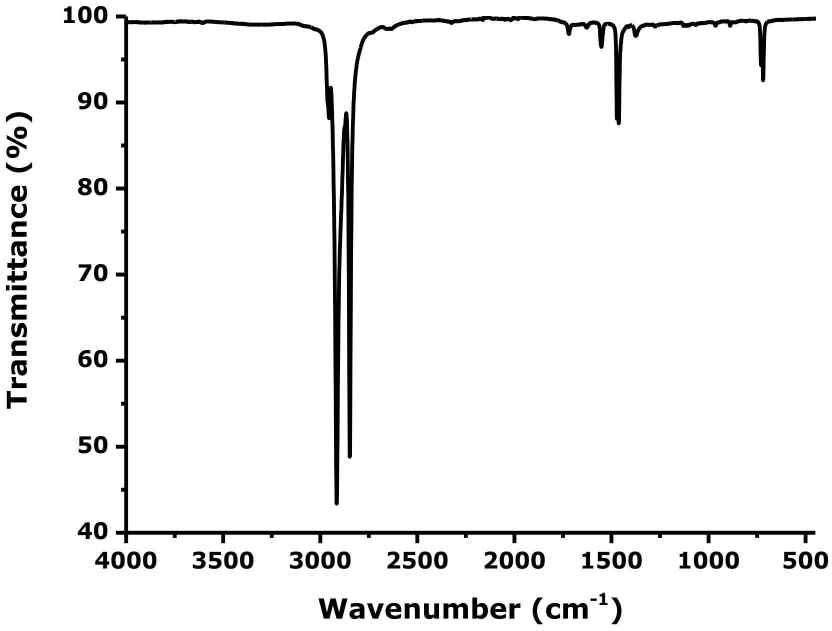

Figure S45: *FTIR (ATR) Spectrum of the isolated product after the photochemical oximation of n-hexatriacontane with Acetic Acid.*

Photochemical reaction with 1,4-Cyclohexadiene

We also explored 1,4-cyclohexadiene as H• donor to reform the oxime. However, the addition of 1,4-cyclohexadiene resulted in a low degree of functionalization. ^{1}H NMR spectroscopy of the crude reaction mixture showed that 1,4-cyclohexadiene was fully consumed and converted into benzene, but most likely hinders the HAT from the alkoxy radical to polymer backbone more then the decay of the iminoxyl radical.

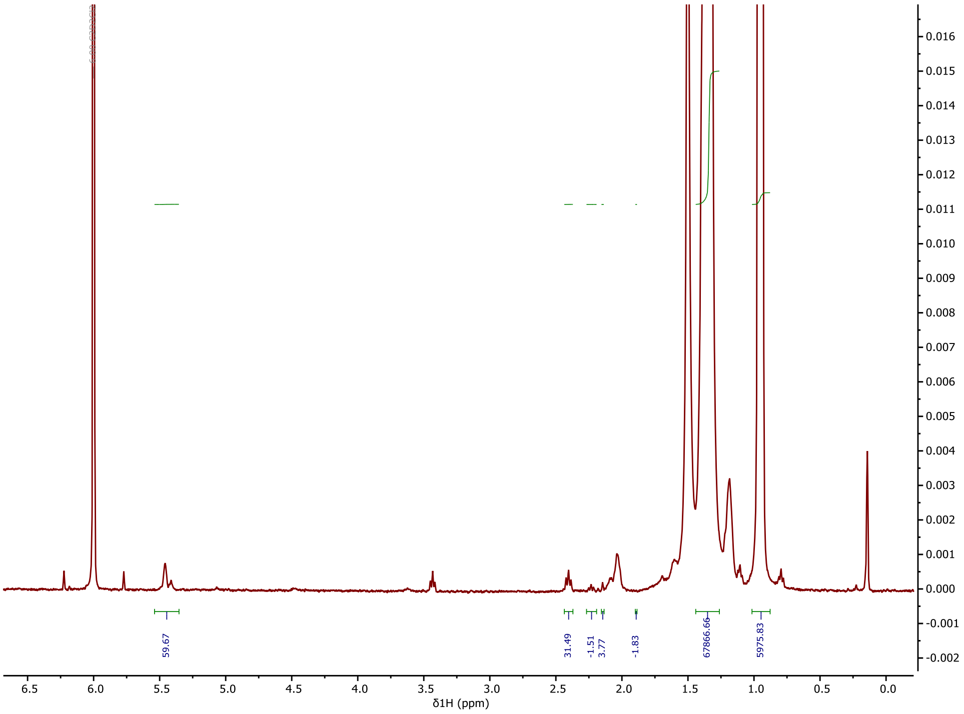

Figure S46: *^{1}H NMR Spectrum of the isolated product after the photochemical oximation of n-hexatriacontane with 1,4-cyclohexadiene in $C_2D_2Cl_4$ at 120 °C.*

Photochemical oximation at 10 bar N_2 in Parr window autoclave (Table 2, Entry 13)

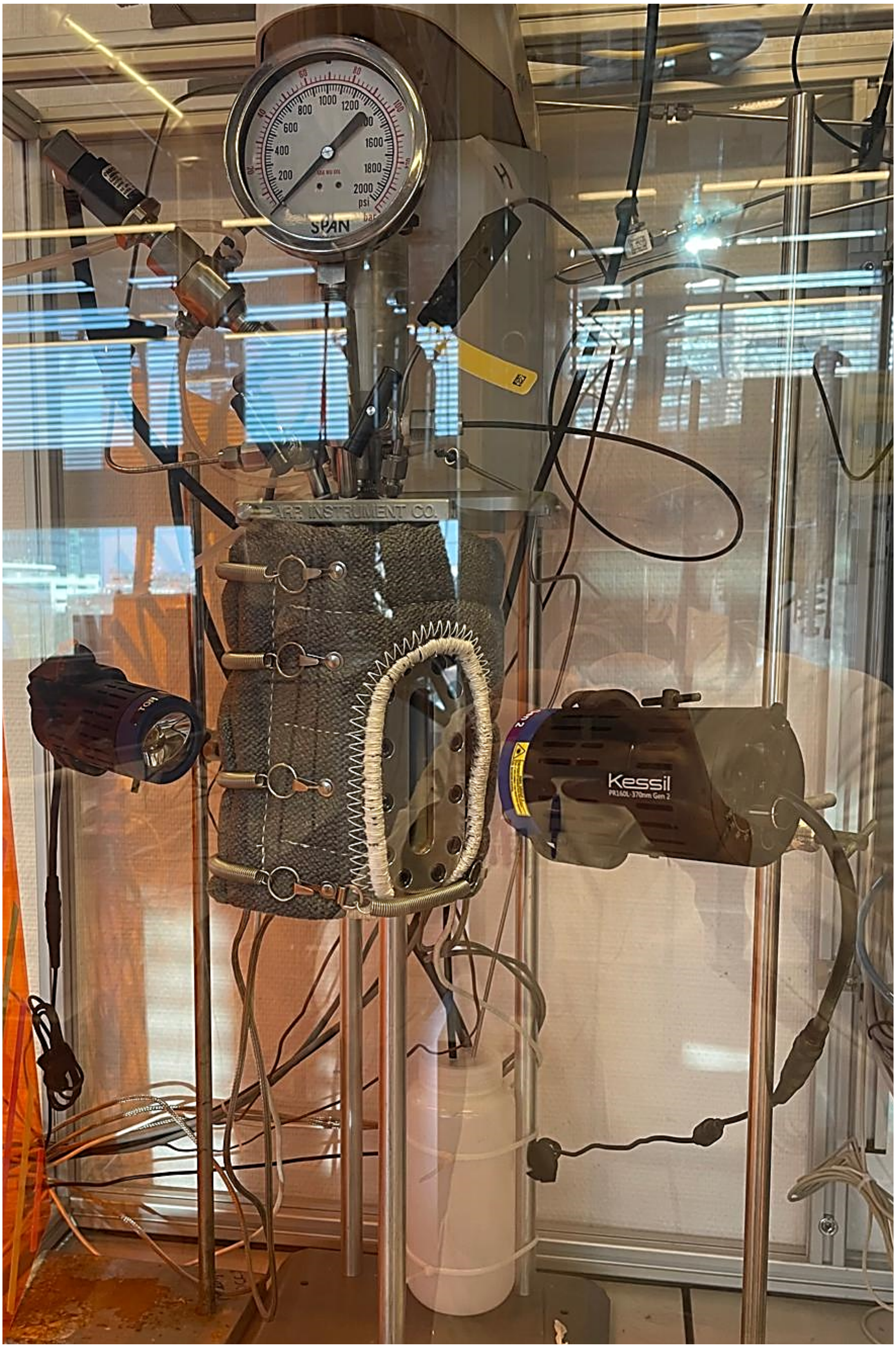

Figure S47: *Custom made Parr window autoclave set-up used for the photochemical oximation of PE (PE-S) at 10 bar N_2 atmosphere.*

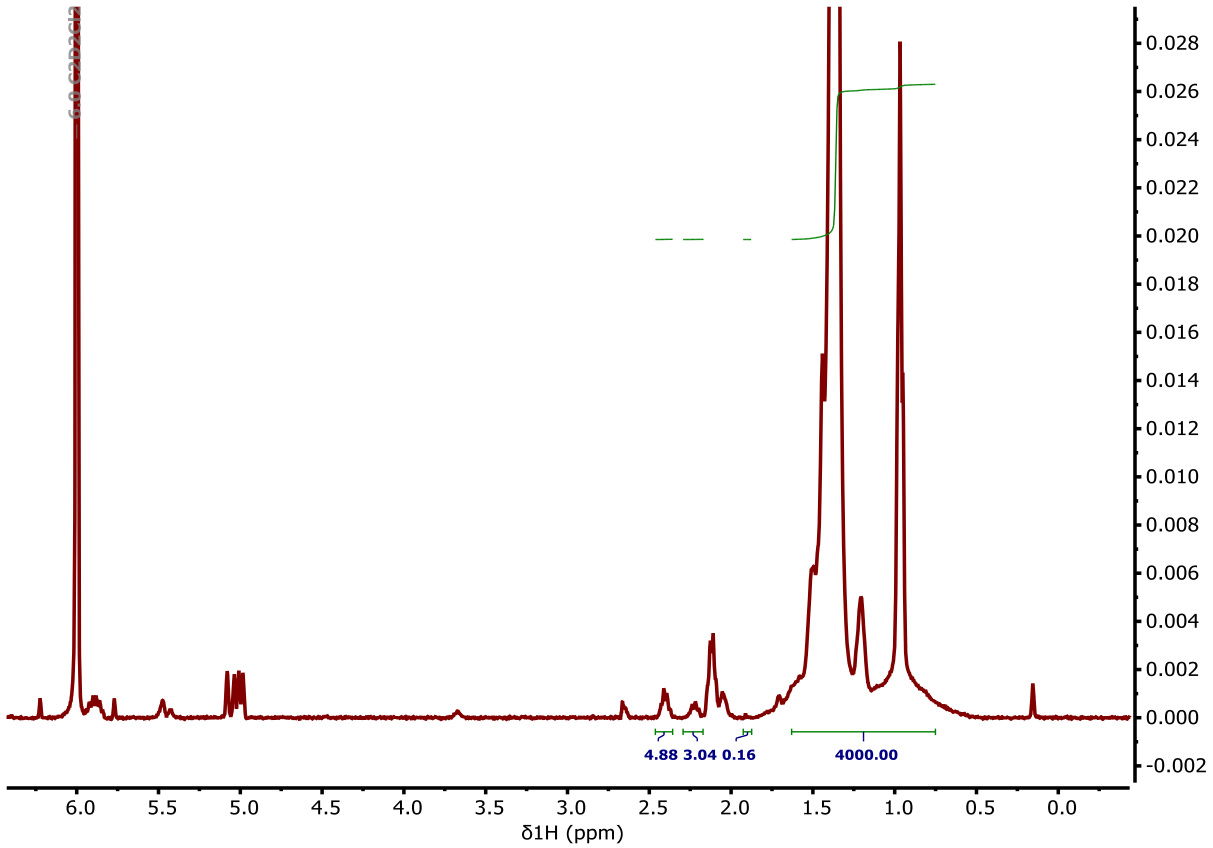

Figure S48: *^1H NMR Spectrum of the isolated product after the photochemical oximation of PE (PE-S) at 10 bar N_2 atmosphere, measured in $C_2D_2Cl_4$ at 120 °C.*

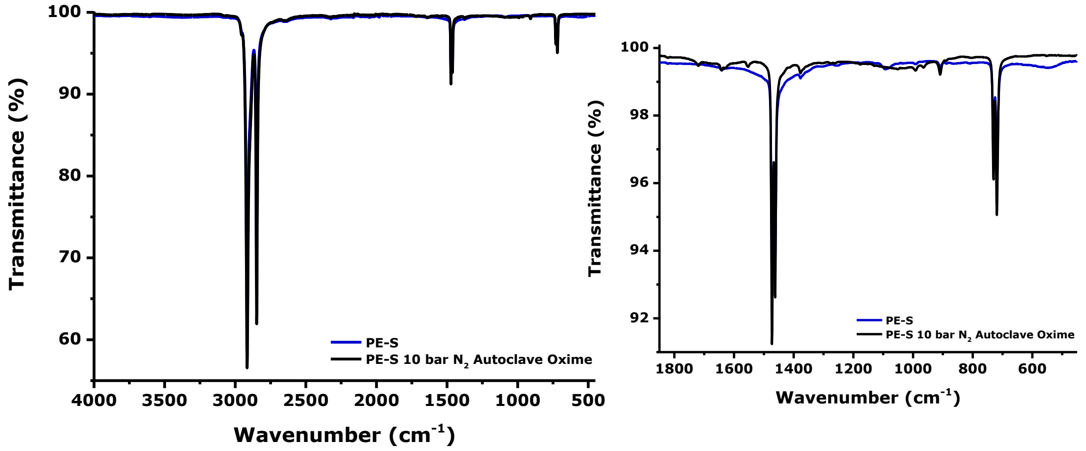

Figure S49: *FTIR (ATR) Spectrum of the isolated product after the photochemical oximation of PE (PE-S) at 10 bar N_2 atmosphere, measured in $C_2D_2Cl_4$ at 120 °C.*

Photochemical oximation with subsequent reaction with NH_2OH•sHCl in one pot (Table 2, Entry 14)

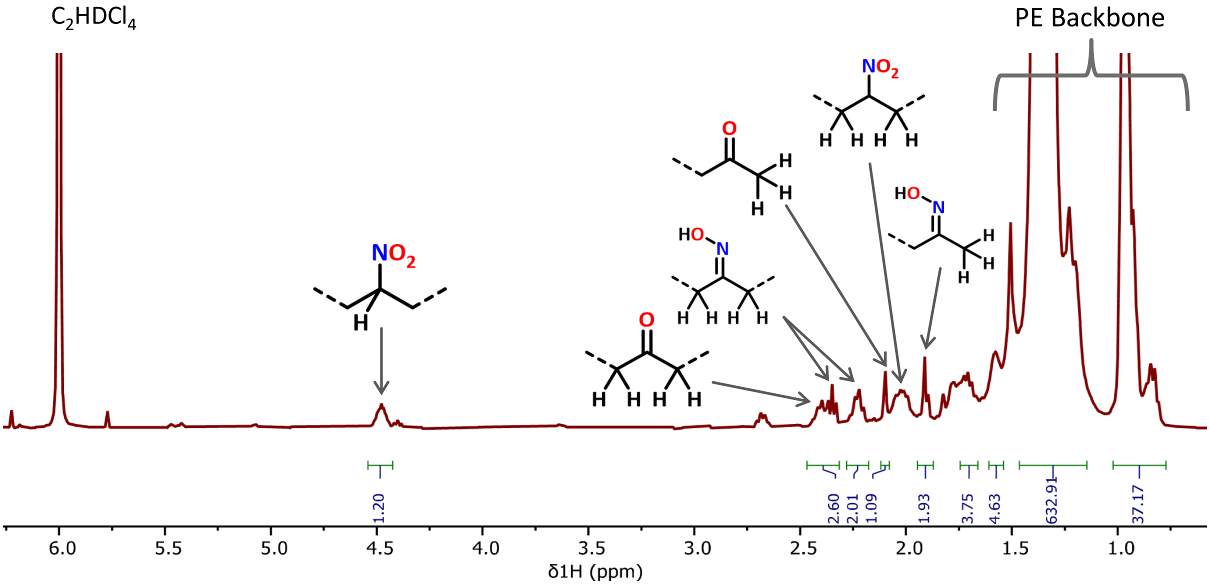

Figure S50: *^{1}H NMR Spectrum of the isolated product after the one-pot photochemical oximation of PE (PE-C Low M_w) and the reaction with NH_2OH•HCl in pyridine, measured in $C_2D_2Cl_4$ at 120 °C.*

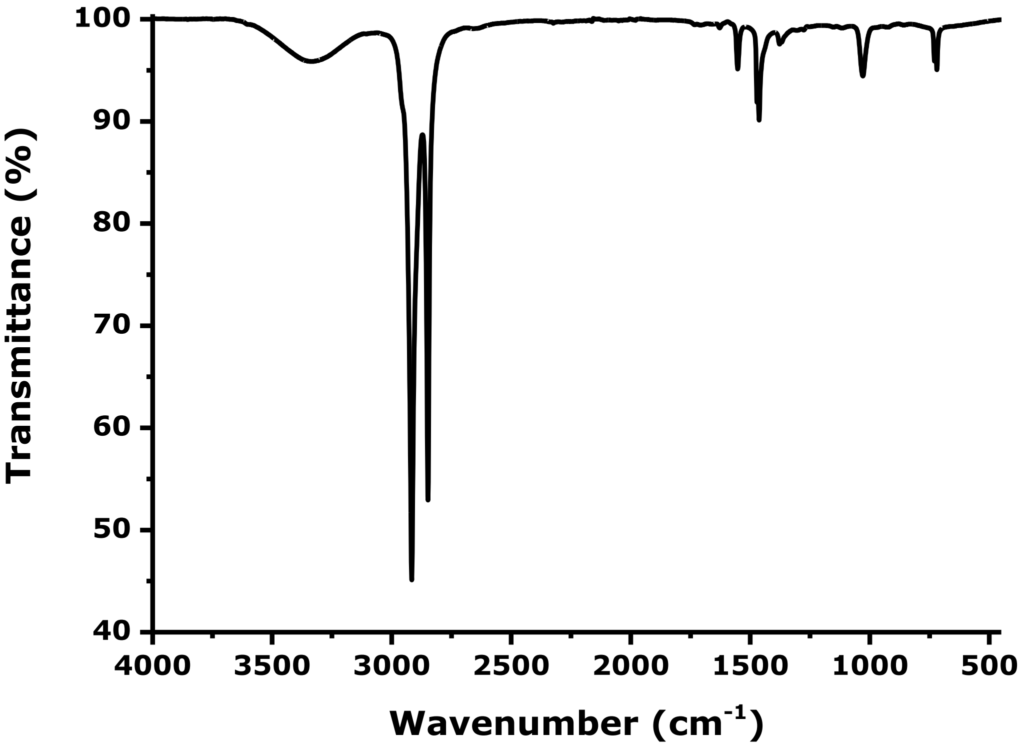

Figure S51: *FTIR (ATR) Spectrum of the isolated product after the one-pot photochemical oximation of PE (PE-C Low M_w) and the reaction with NH_2OH•HCl in pyridine.*

Reflux set-up used for the Photochemical reaction (Table 2, Entry 15)

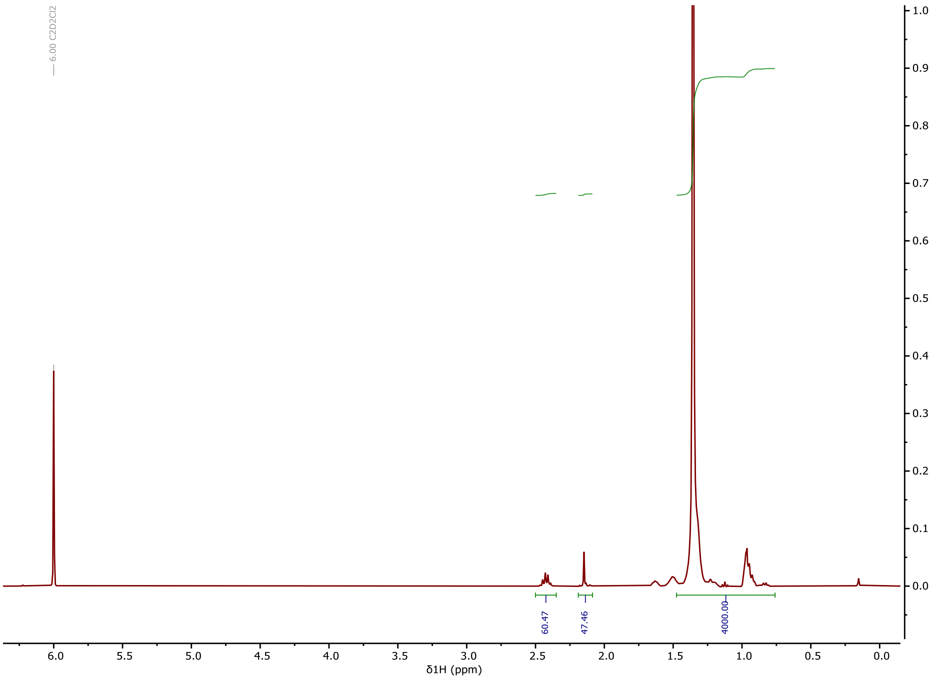

Figure S52: *^{1}H NMR Spectrum of the isolated product after the photochemical oximation of PE (PE-C) with a reflux set-up in $C_2D_2Cl_4$ at 120 °C.*

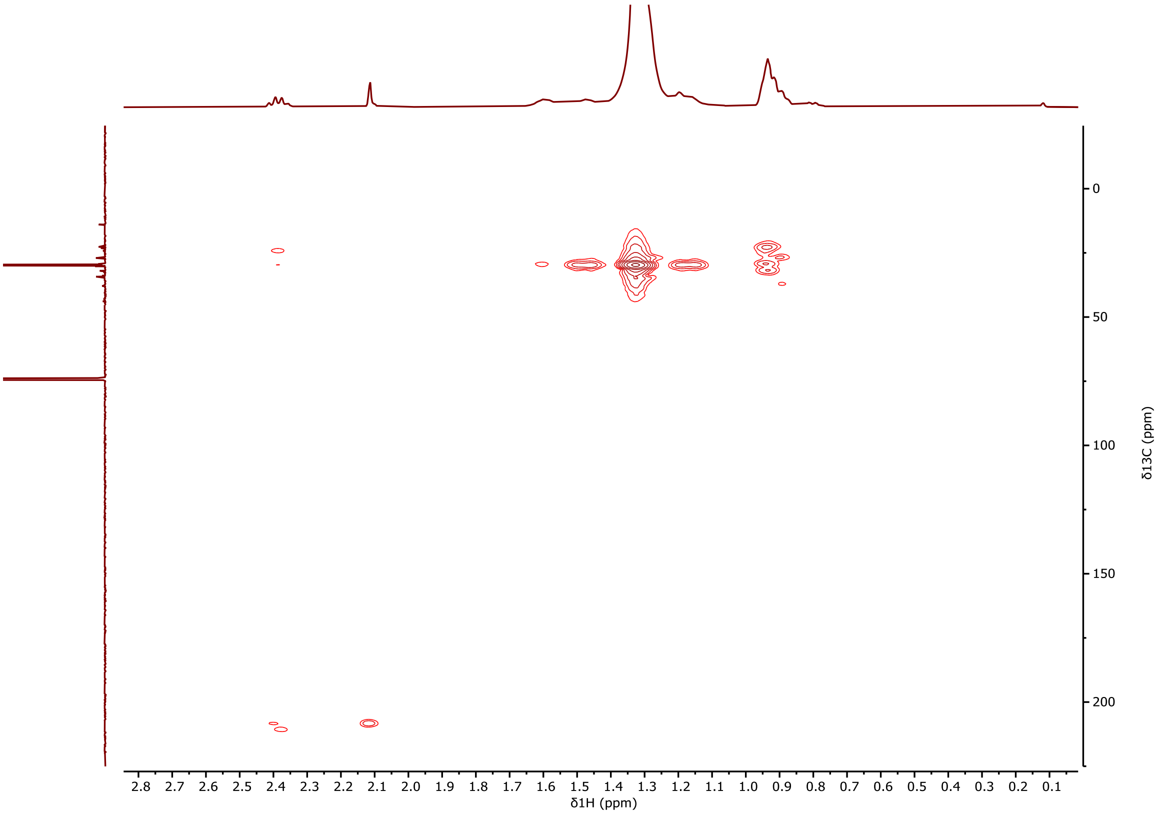

Figure S53: *^{1}H-[13]C Heteronuclear Multiple Bond Correlation (HMBC) spectrum of the isolated product after the photochemical oximation of PE (PE-C Low M_w) with a reflux set-up in $C_2D_2Cl_4$ at 120 °C.*

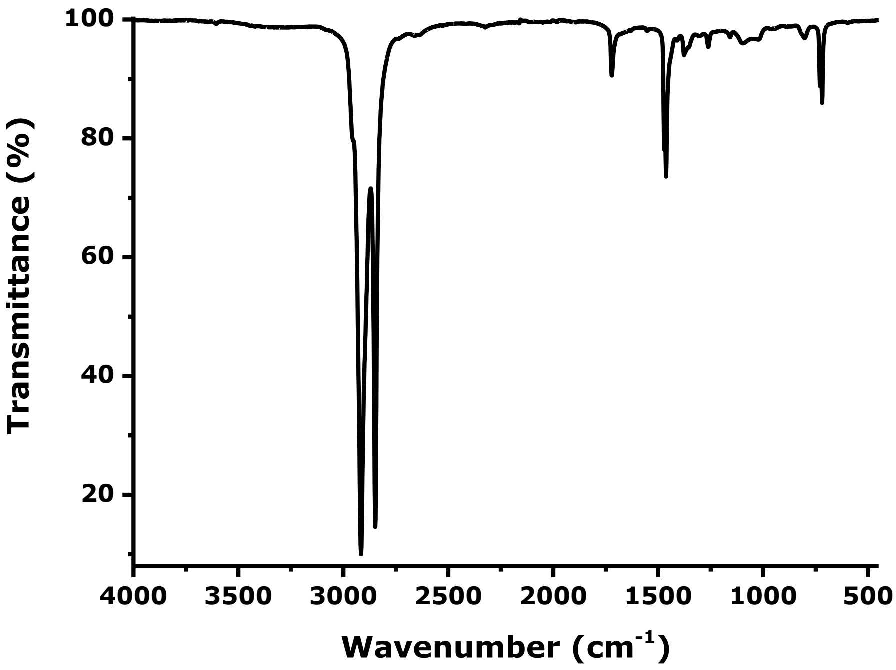

Figure S54: *FTIR (ATR) Spectrum of the isolated product after the photochemical oximation of PE (PE-C Low M_w) with a reflux set-up.*

Cyclohexane oximation

To develop the PE photochemical PPM methodology, we first revisited the work of Wysocki and co-workers, with the photochemical oximation of cyclohexane cleanly leading to the formation of cyclohexanone oxime when using a 370 nm LED light source (**Table S2, entry 3**). Using longer wavelength light sources (**Table S2, entries 1,2**) (440 nm or a 525 nm) showed a lack of selective product formation. Using a broad illumination UV light 250-400 nm resulted in no product formation, due to complete decomposition of *t*-BuONO below 320 nm, and ^{1}H NMR analysis of the reaction mixture showed that C-C bond cleavage of the substrate occurred to give a complex mixture of small alkane fractions (**Table S2, entry 4**). The formation of cyclohexanone using the lower energy wavelengths is hypothesized to be the result of two things. First, the *t*-BuONO does not have any absorption bands at 440 nm which hinders the selective formation of the NO radical with only heat.[15] Second, the formation of these other products possibly decompose the oxime.

Table S2: *Investigation of Photochemical Conditions for the Oximation of Cyclohexane with the use of different wavelengths.*

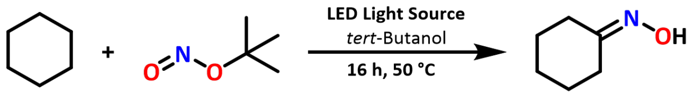

Entry	LED Light Source (nm)	Time (h)	Oxime Yield (%)
1	525	16	Trace amounts
2	440	16	36
3	370	16	80
4	250-400	16	0

Table S2, Entry 1

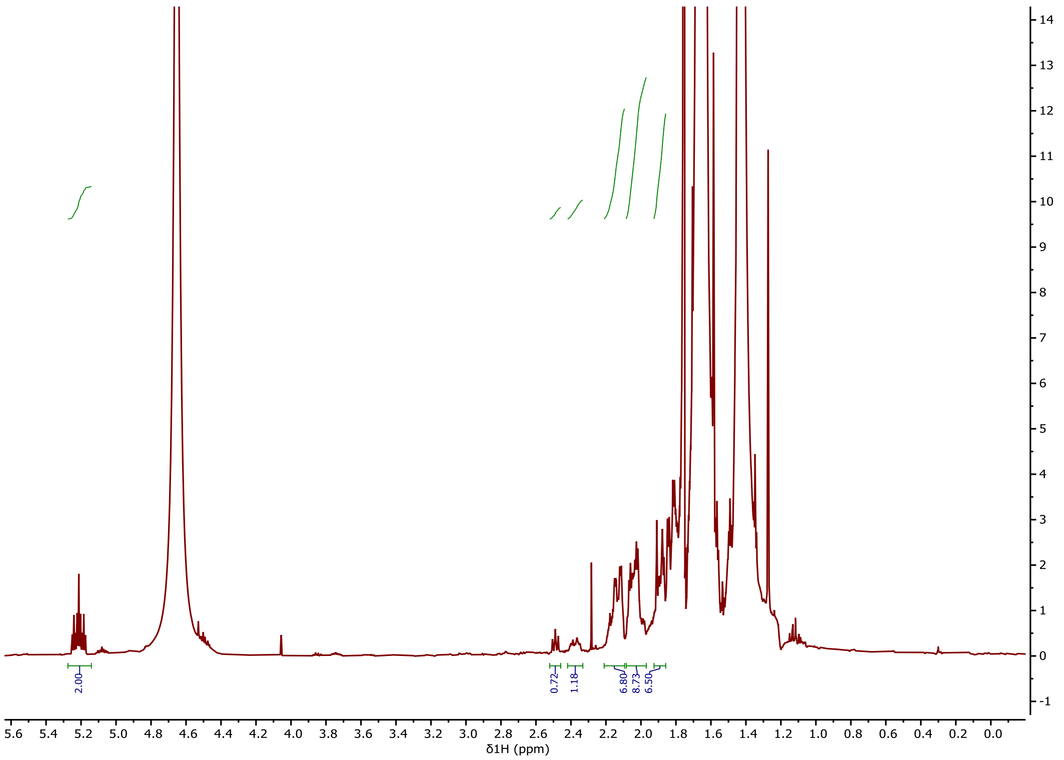

Figure S55: *^{1}H NMR Spectrum of the isolated product after the photochemical oximation of cyclohexane with the 525 nm light source in $CDCl_3$ at 25°C. Zoomed in on the cyclohexanone and cyclohexanone oxime product region between 6 and 0 ppm.*

Table S2, Entry 2

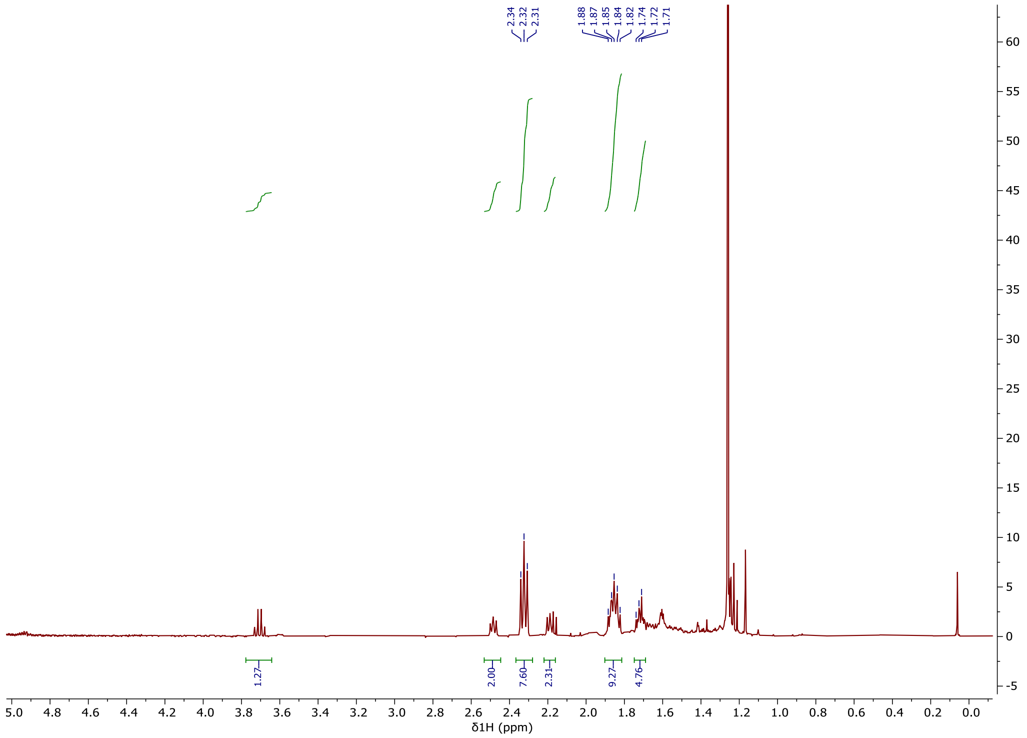

Figure S56: *^{1}H NMR Spectrum of the isolated product after the photochemical oximation of cyclohexane with the 440 nm light source in $CDCl_3$ at 25°C. Zoomed in on the cyclohexanone and cyclohexanone oxime product region between 5 and 0 ppm.*

Table S2, Entry 3

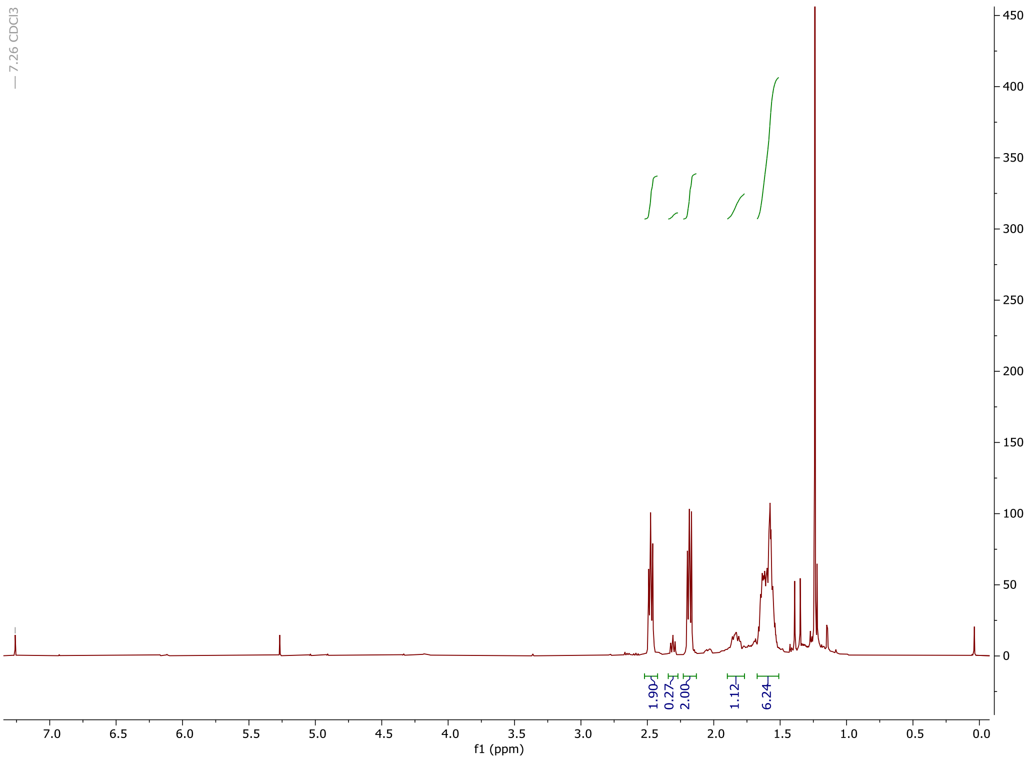

Figure S57: *^{1}H NMR Spectrum of the isolated product after the photochemical oximation of cyclohexane with the 370 nm light source in $CDCl_3$ at 25°C.*

Oximation using a higher boiling organic nitrite source

We investigated replacing *t*-BuONO (bp. 61 °C) for the higher boiling isopentyl nitrite (bp. 99 °C), but this resulted in a decreased yield. This lower yield is attributed to the Barton reaction taking place on isopentyl nitrite in which the alkoxy radical abstracts a hydrogen from the δ-carbon instead of recombining with the H• abstracted from the substrate backbone.[16]

6. Reference Oxime Compounds Data

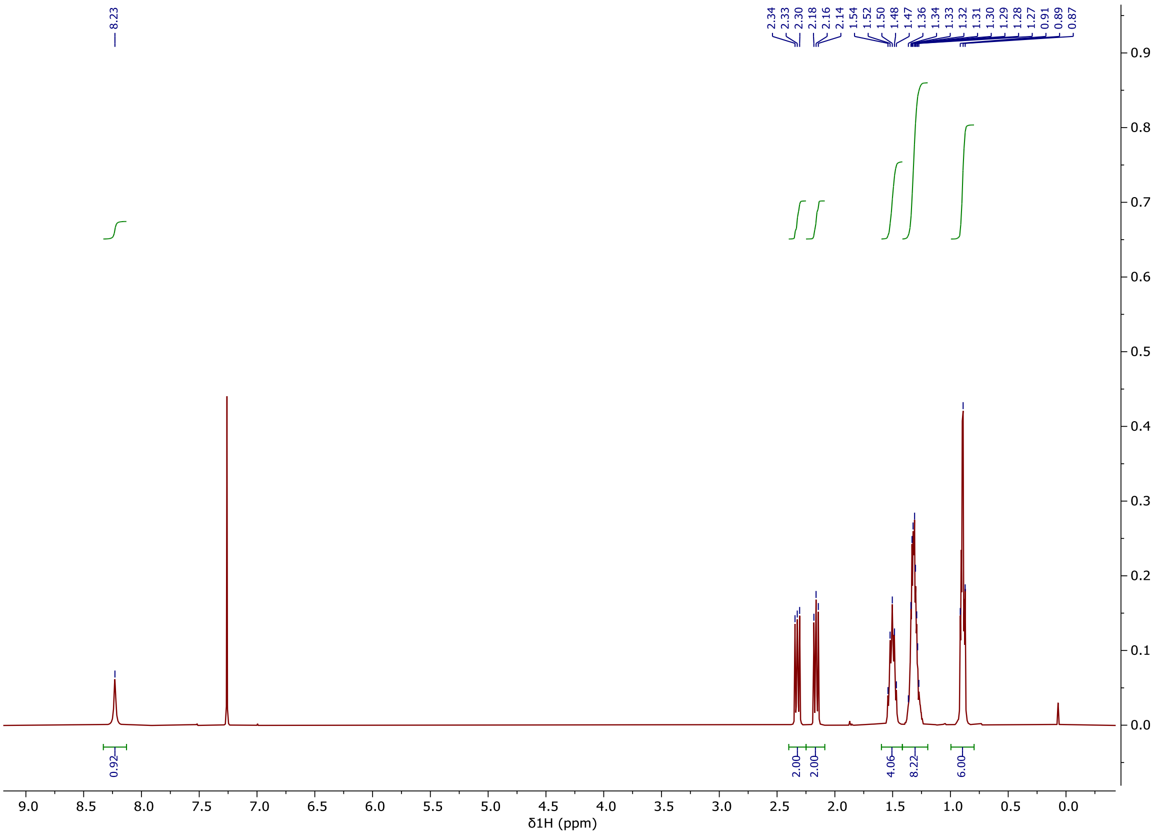

Figure S58: *^{1}H NMR spectrum of 6-undecanone oxime in $CDCl_3$ at 25 °C.*

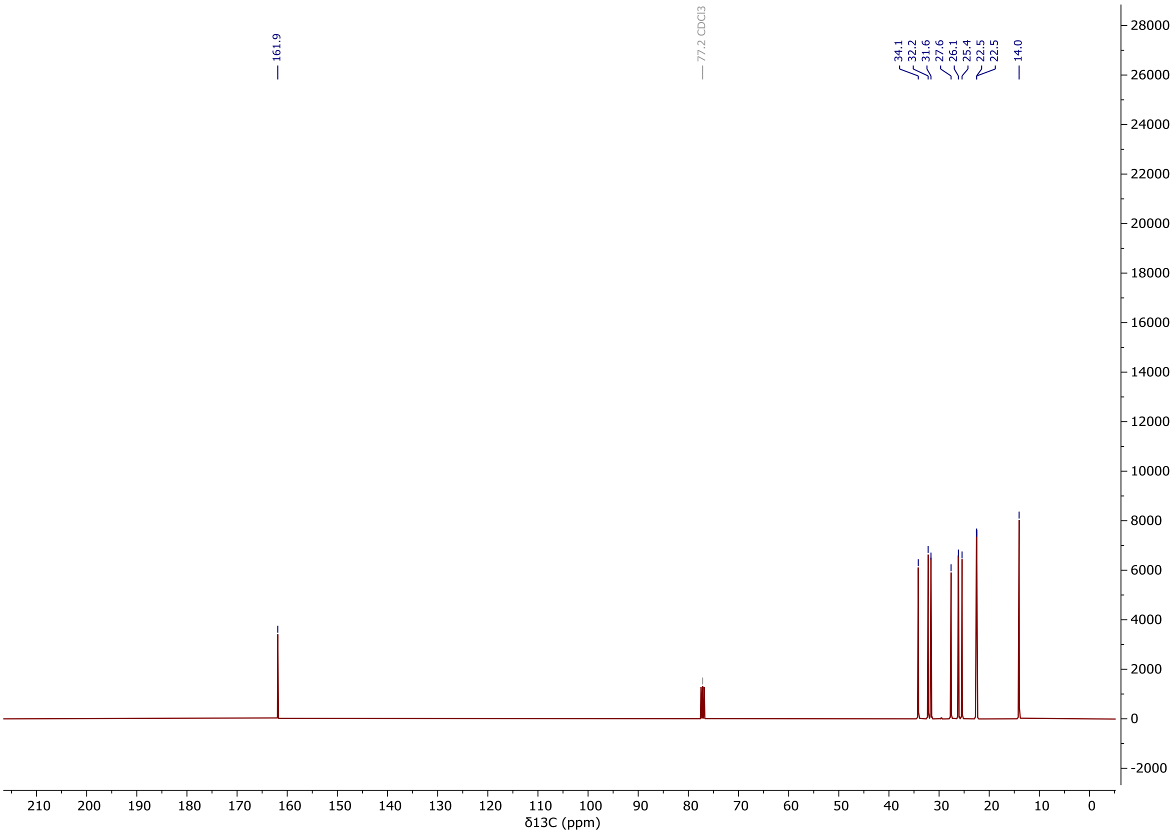

Figure S59: *^{13}C NMR spectrum of 6-undecanone oxime in $CDCl_3$ at 25 °C.*

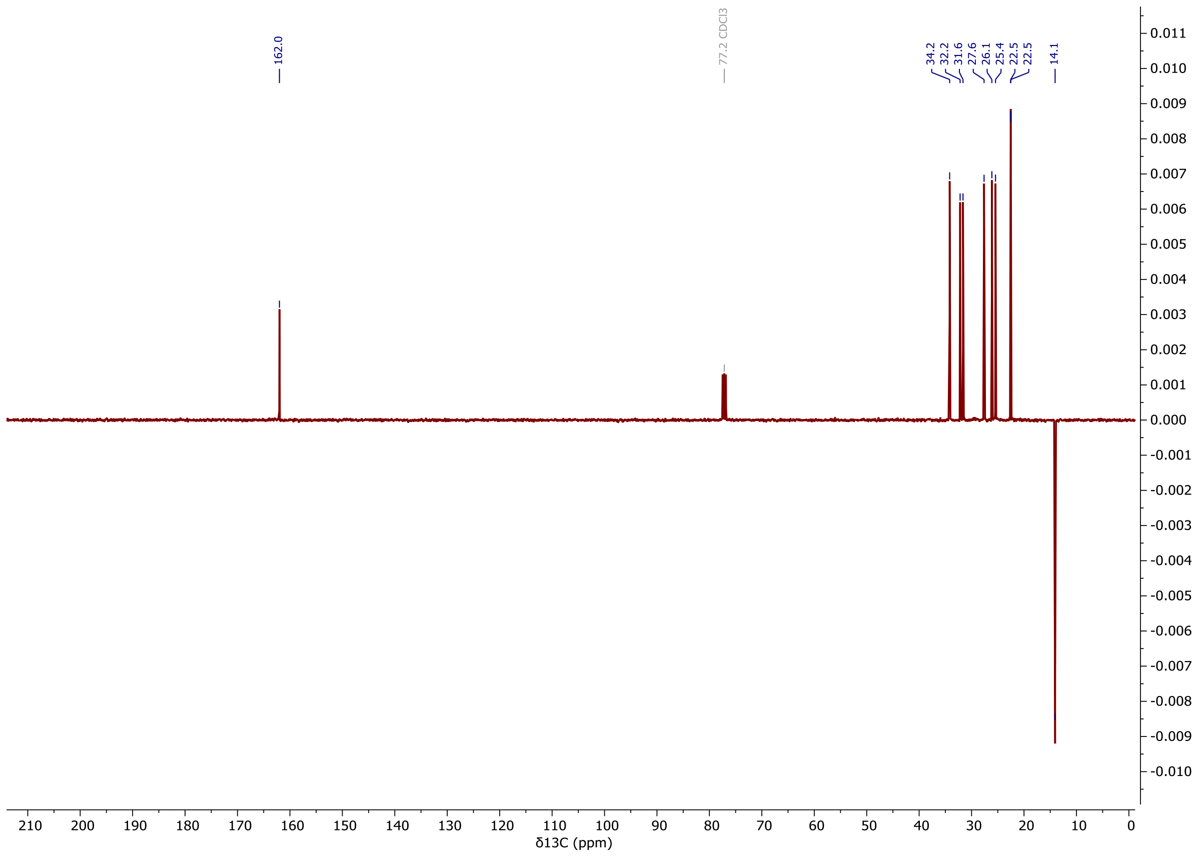

Figure S60: *^{13}C NMR (APT) spectrum of 6-undecanone oxime in $CDCl_3$ at 25 °C.*

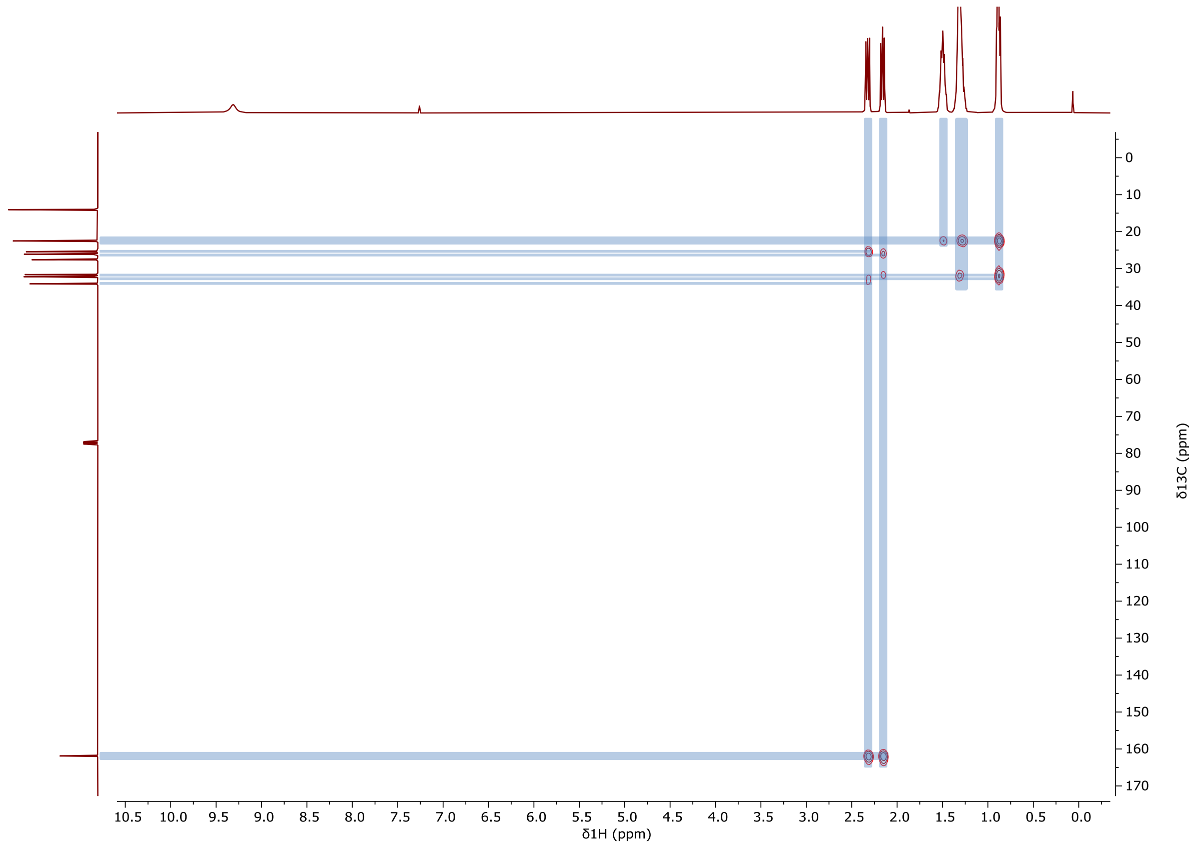

Figure S61: *^{1}H-^{13}C Heteronuclear Multiple Bond Correlation (HMBC) spectrum of 6-undecanone oxime in $CDCl_3$ at 25 °C.*

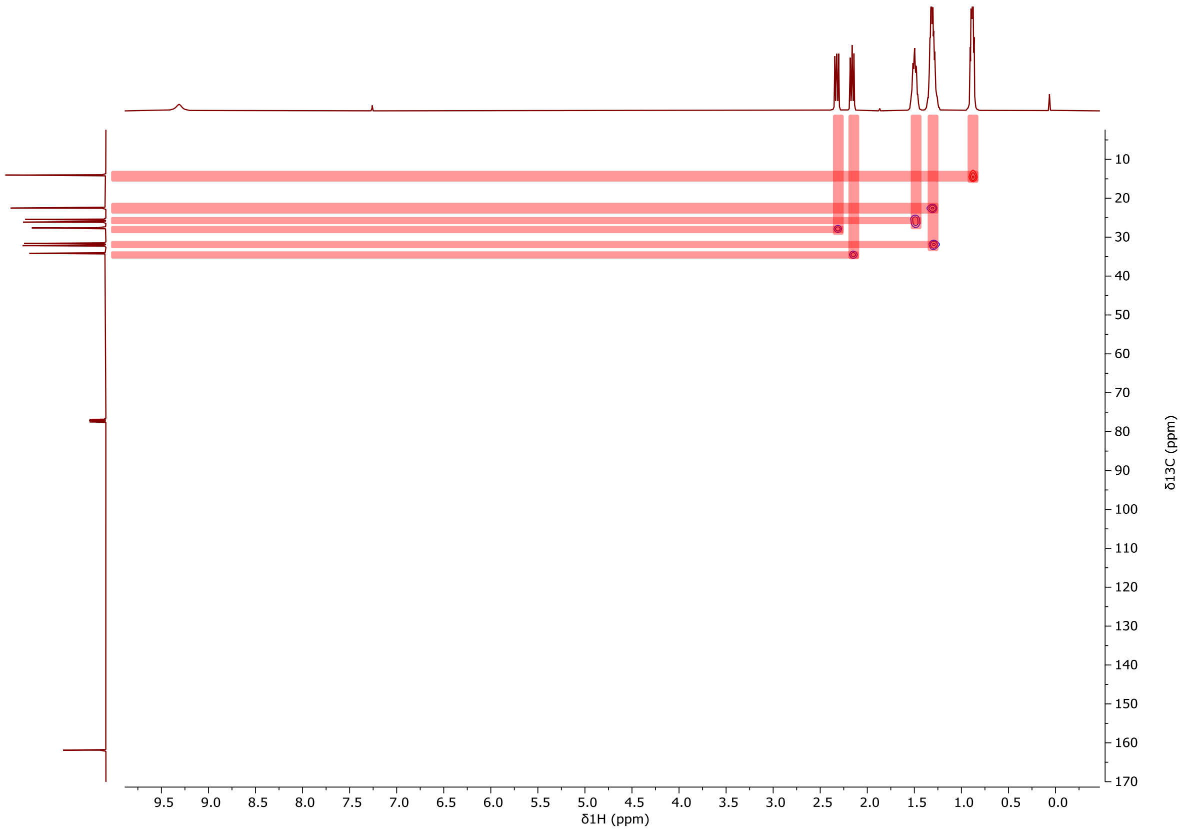

Figure S62: *^{1}H-^{13}C Heteronuclear Single Quantum Coherence (HSQC) spectrum of 6-undecanone oxime in $CDCl_3$ at 25 °C.*

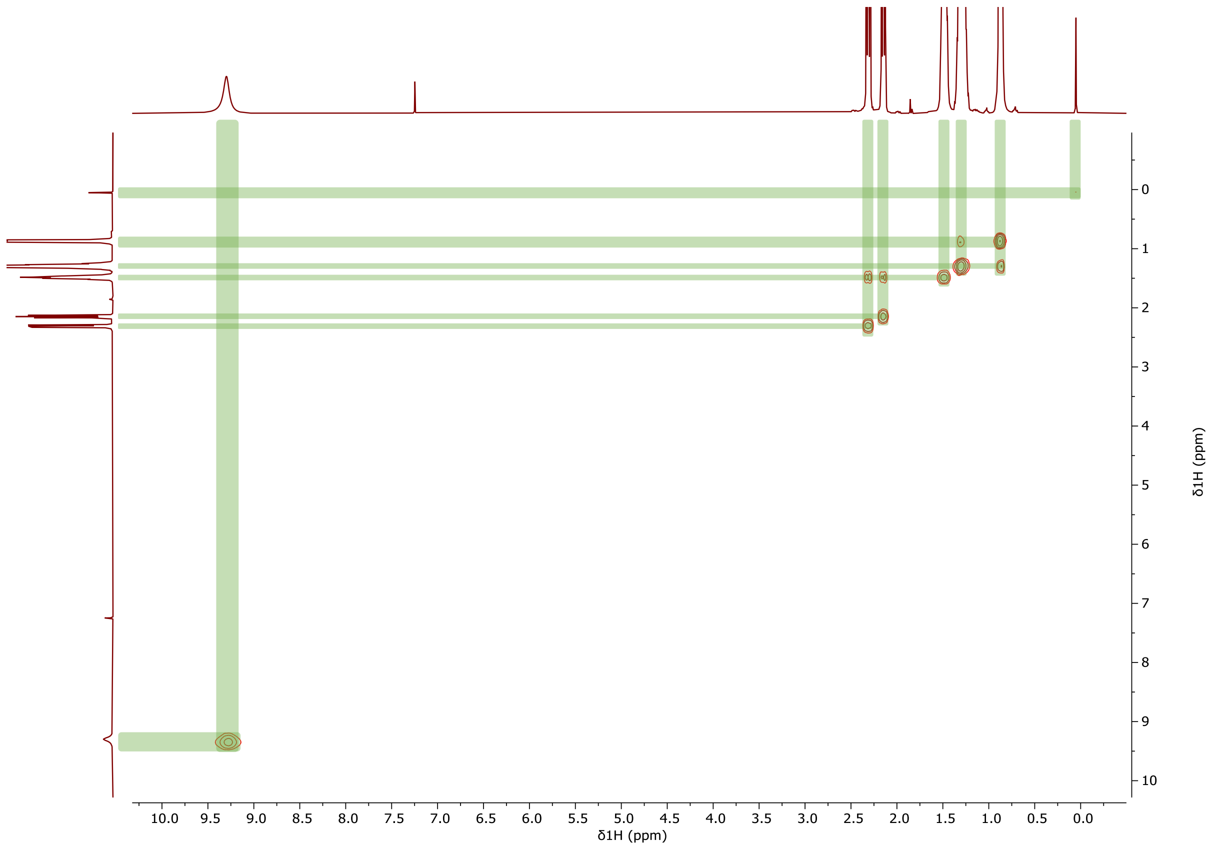

Figure S63: *^{1}H-^{1}H Correlated Spectroscopy (COSY) spectrum of 6-undecanone oxime in $CDCl_3$ at 25 °C.*

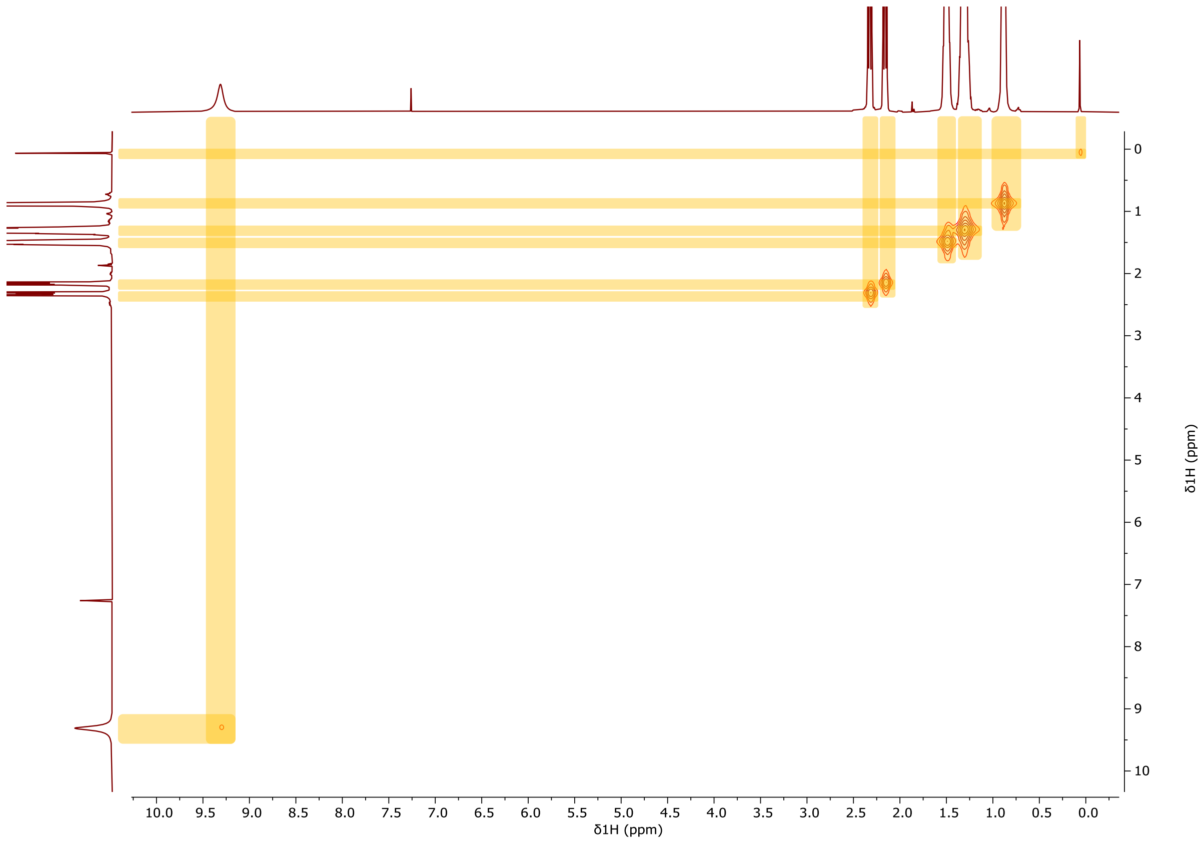

Figure S64: ^{1}H-^{1}H Nuclear Overhauser Effect Spectroscopy (NOESY) spectrum of 6-undecanone oxime in $CDCl_3$ at 25 °C.

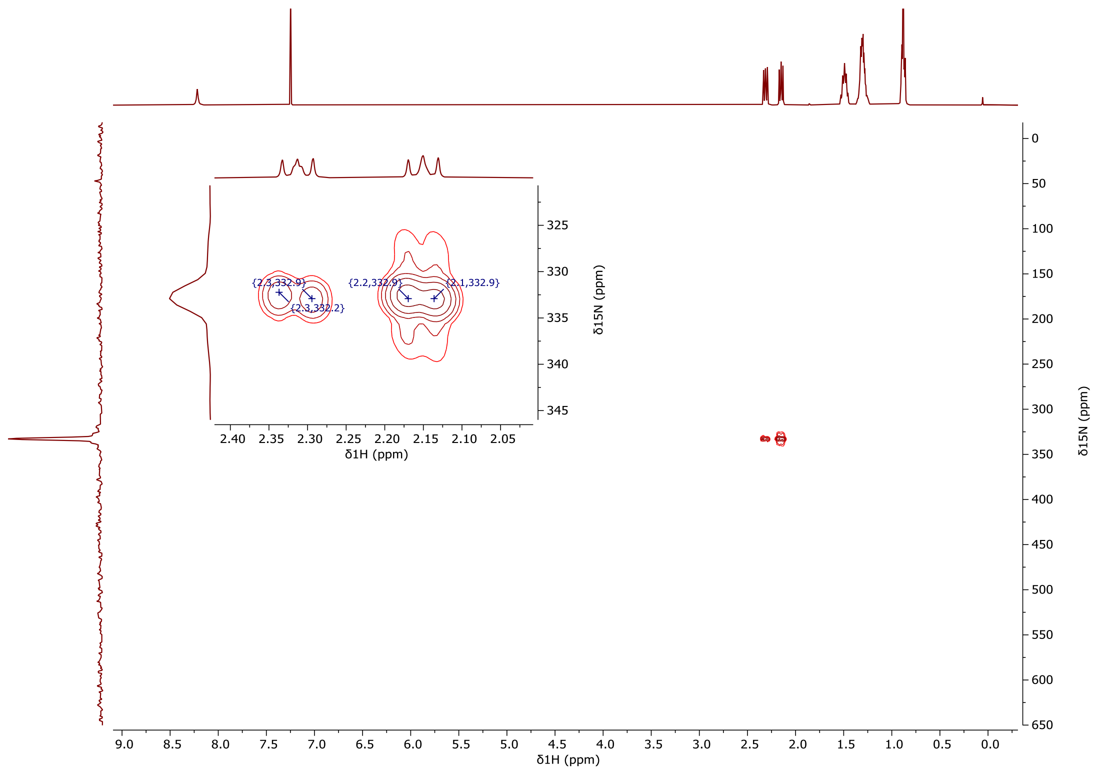

Figure S65: ^{1}H-^{15}N Heteronuclear Multiple Bond Correlation (HMBC) spectrum of 6-undecanone oxime in $CDCl_3$ at 25 °C, with a zoom in on the coupling.

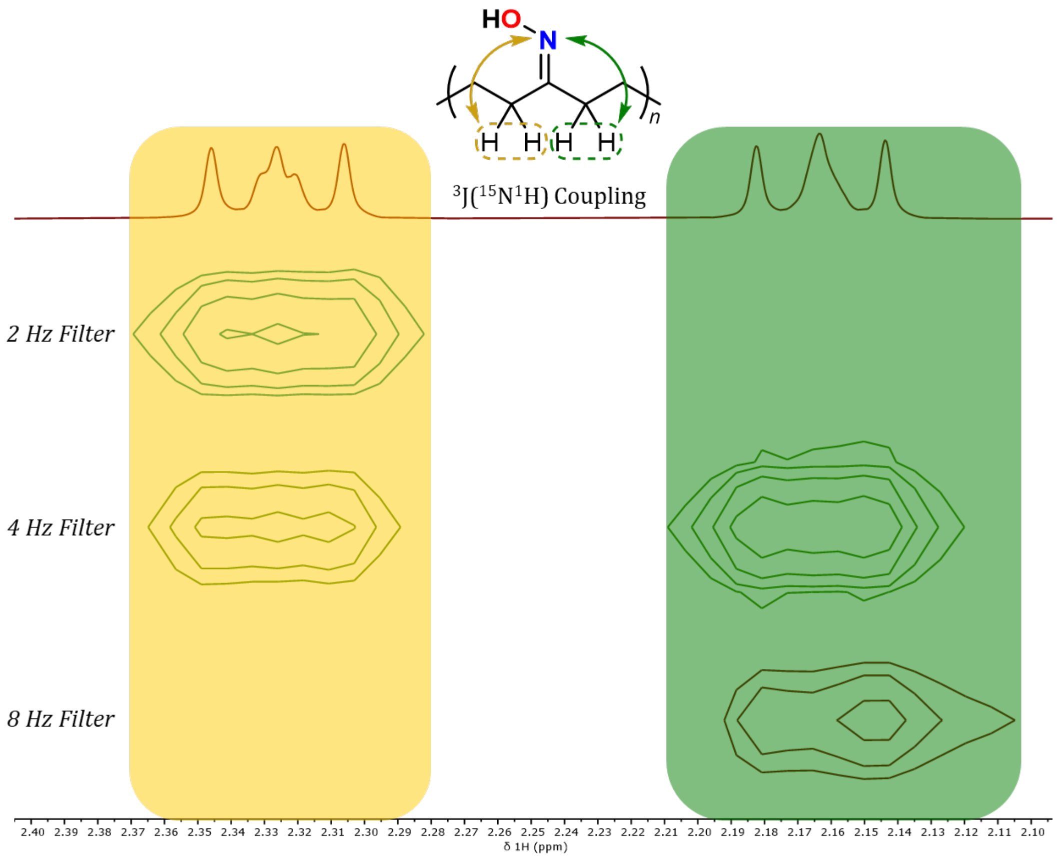

Figure S66: : *^{1}H-^{15}N Heteronuclear Multiple Bond Correlation (HMBC) spectrum of 6-undecanone oxime in $CDCl_3$ at 25 °C, measured with various coupling filters to determine the J-couplings.*

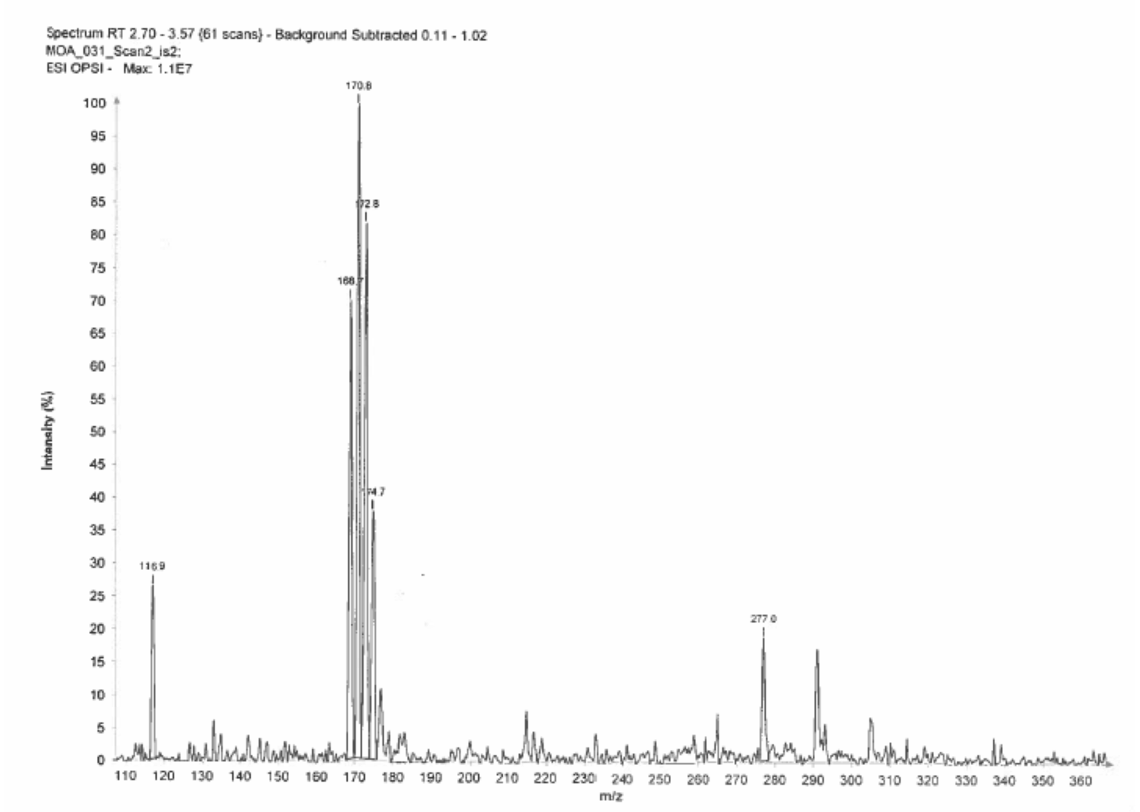

Figure S67: *ESI-MS spectrum of 6-undecanone oxime, measured in acetonitrile.*

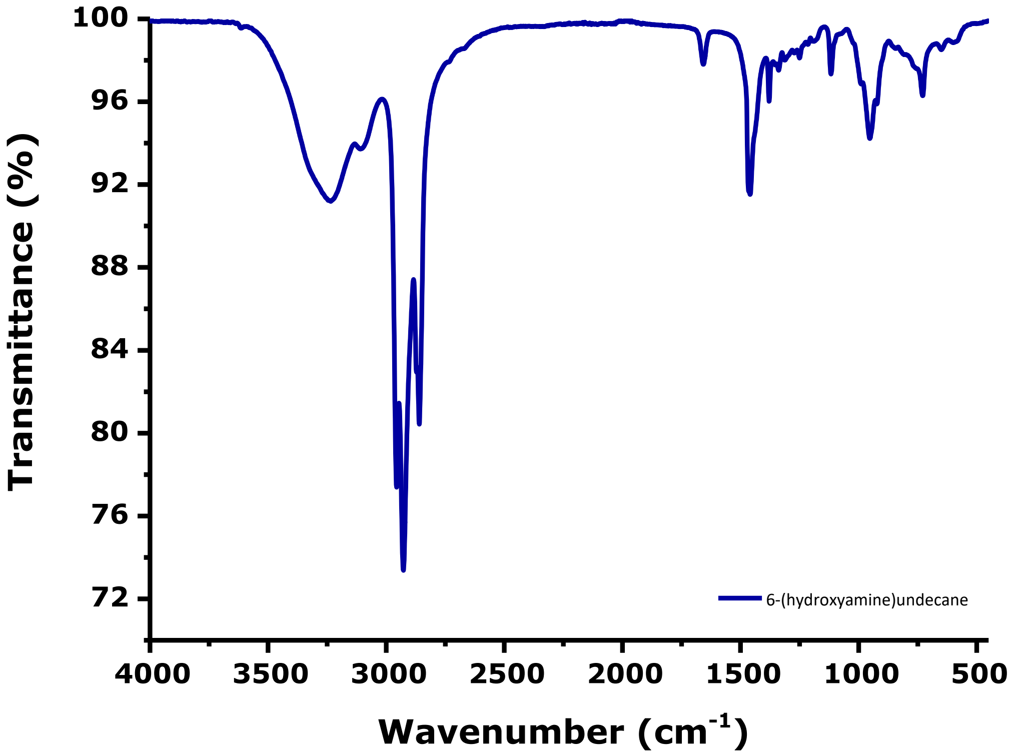

Figure S68: *FTIR (ATR) Spectrum of 6-undecanone oxime.*

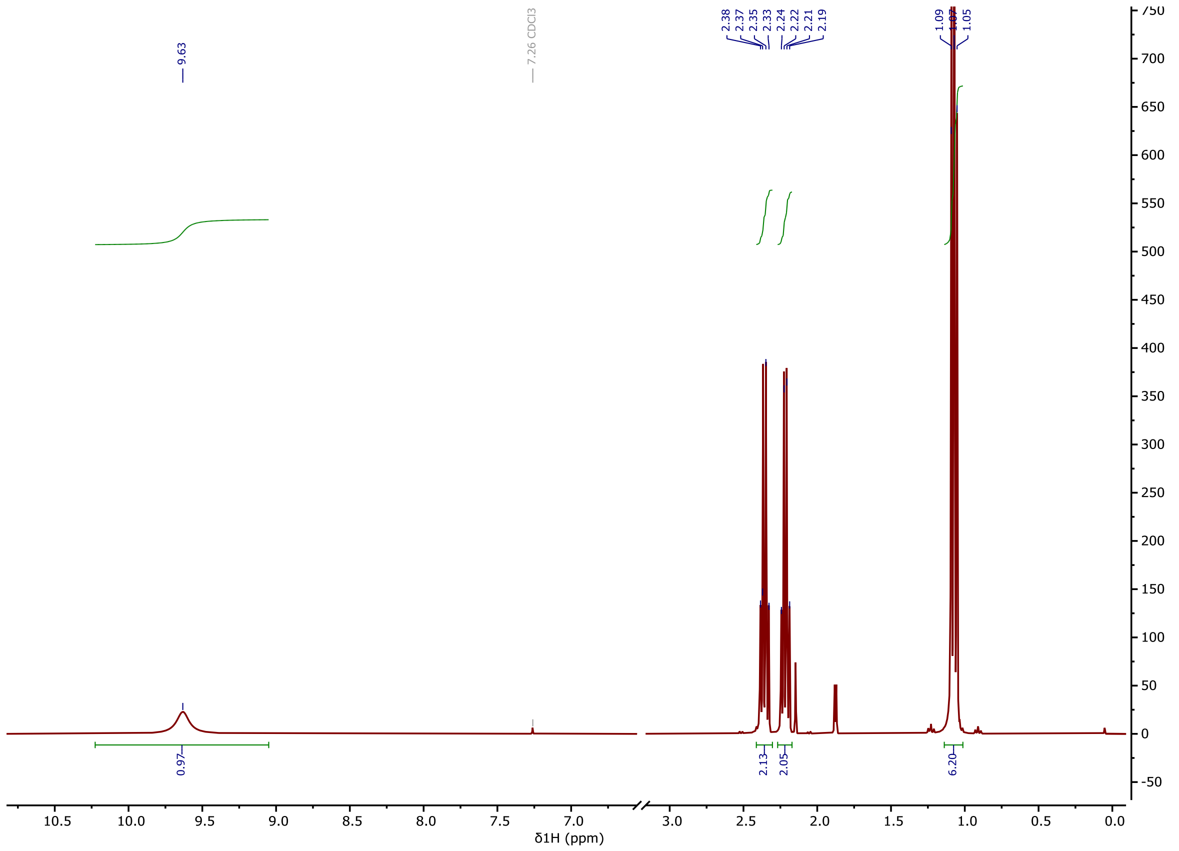

Figure S69: *^{1}H NMR spectrum of 3-pentanone oxime in $CDCl_3$ at 25 °C.*

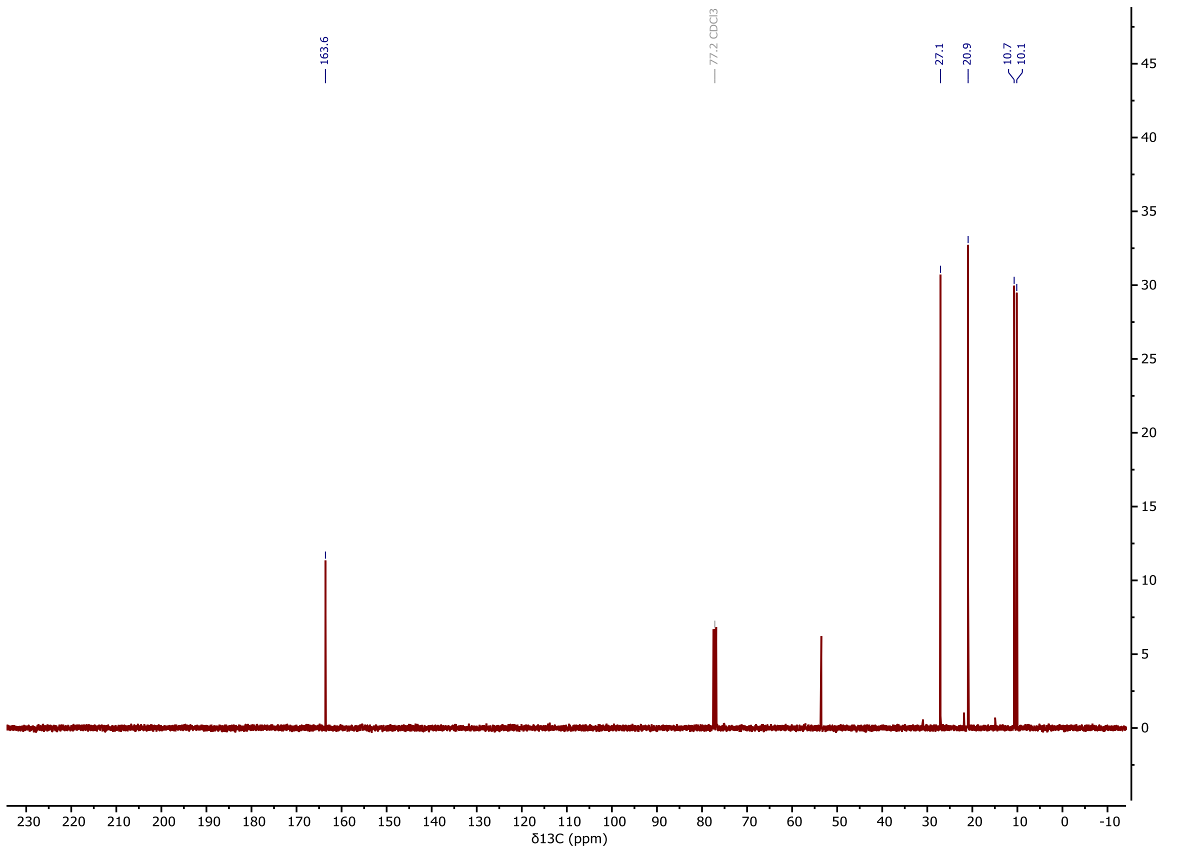

Figure S70: *^{13}C NMR spectrum of 3-pentanone oxime in $CDCl_3$ at 25 °C.*

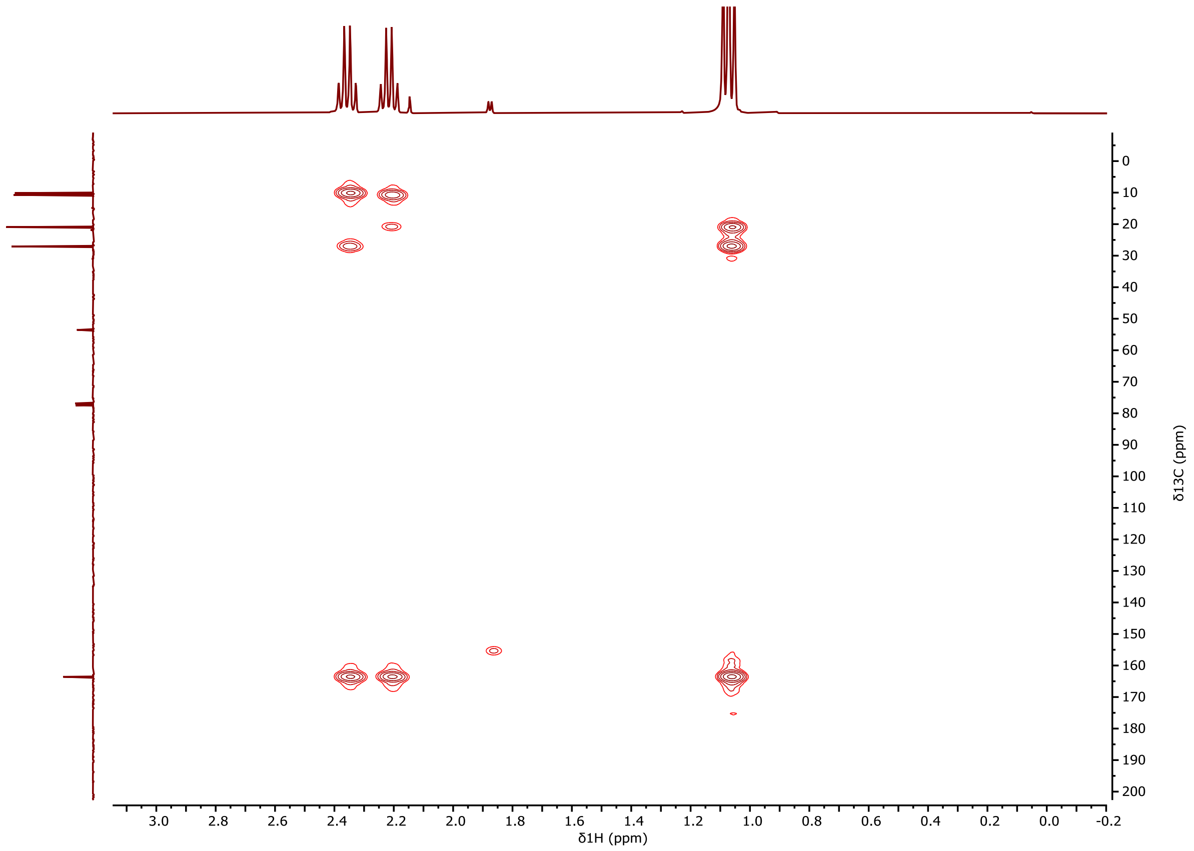

Figure S71: *^{1}H-[13]C Heteronuclear Multiple Bond Correlation (HMBC) spectrum of 3-pentanone oxime in $CDCl_3$ at 25 °C, with a zoom in on the coupling.*

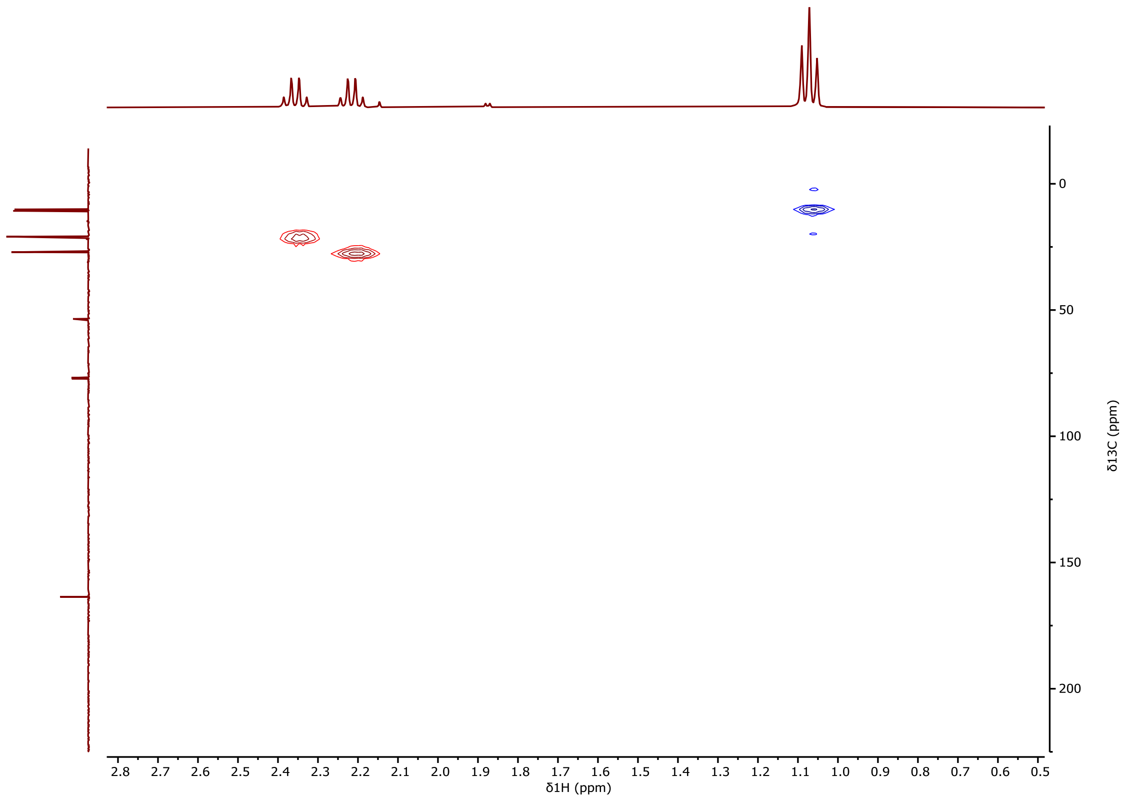

Figure S72: *^{1}H-[13]C Heteronuclear Single Quantum Coherence (HSQC) spectrum of 3-pentanone oxime in $CDCl_3$ at 25 °C, with a zoom in on the coupling.*

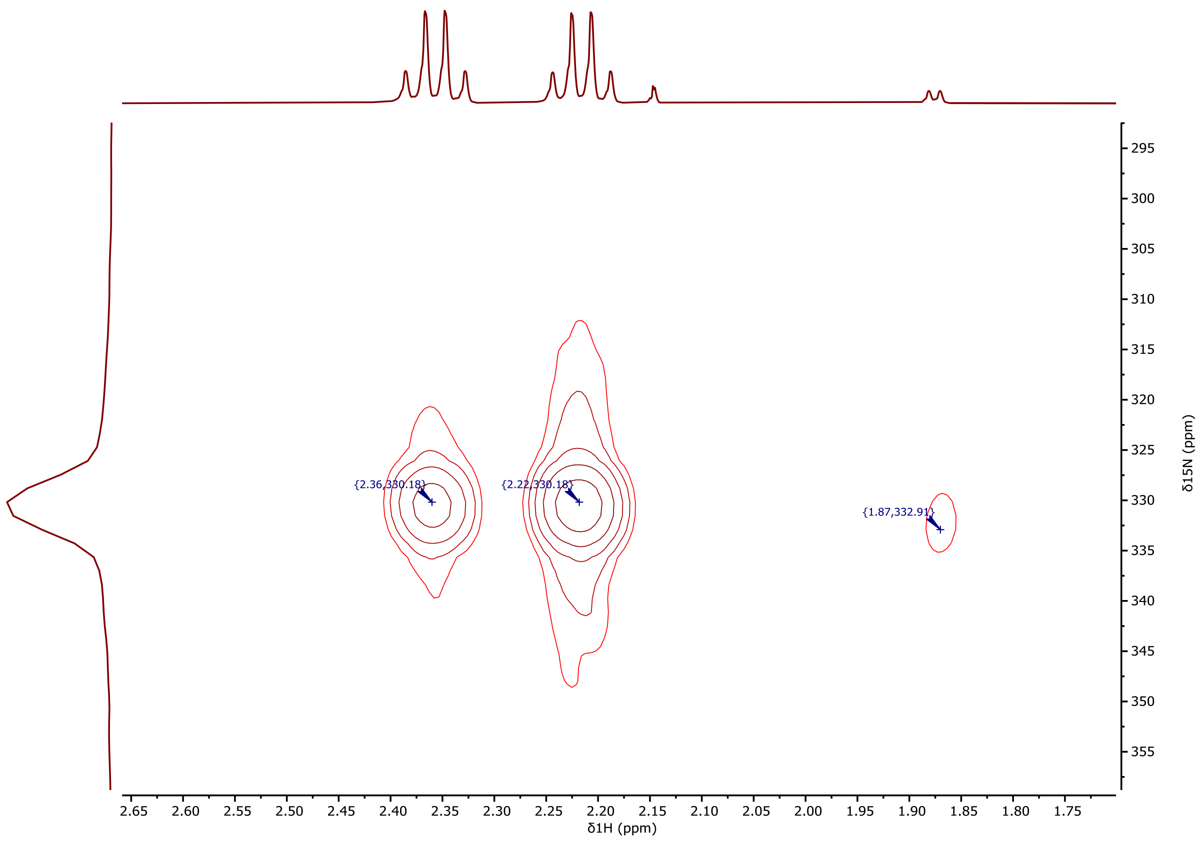

Figure S73: *^{1}H-^{15}N Heteronuclear Multiple Bond Correlation (HMBC) spectrum of 3-pentanone oxime in $CDCl_3$ at 25 °C, with a zoom in on the coupling.*

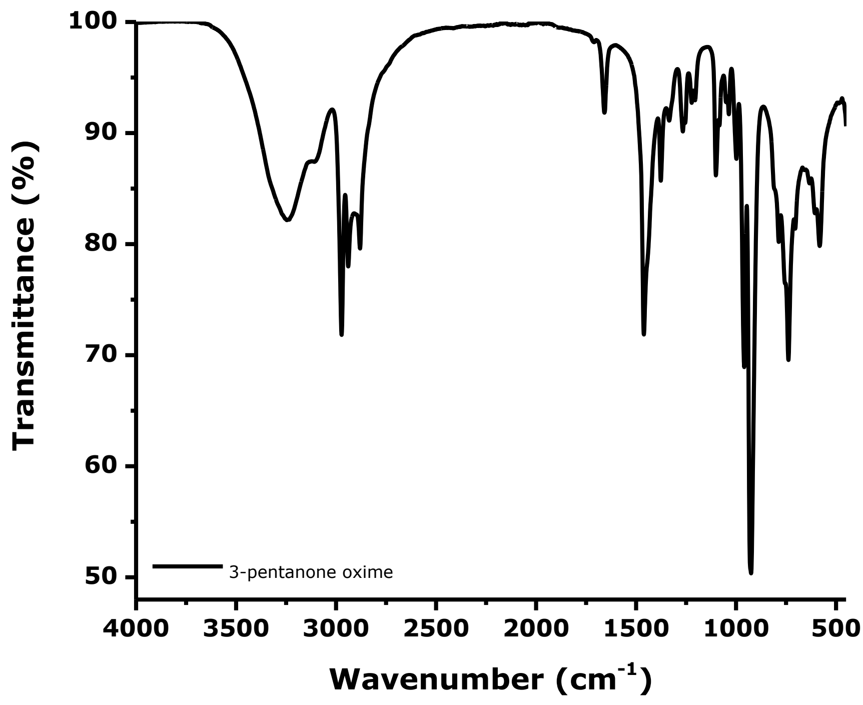

Figure S74: *FTIR (ATR) Spectrum of 3-pentanone oxime.*

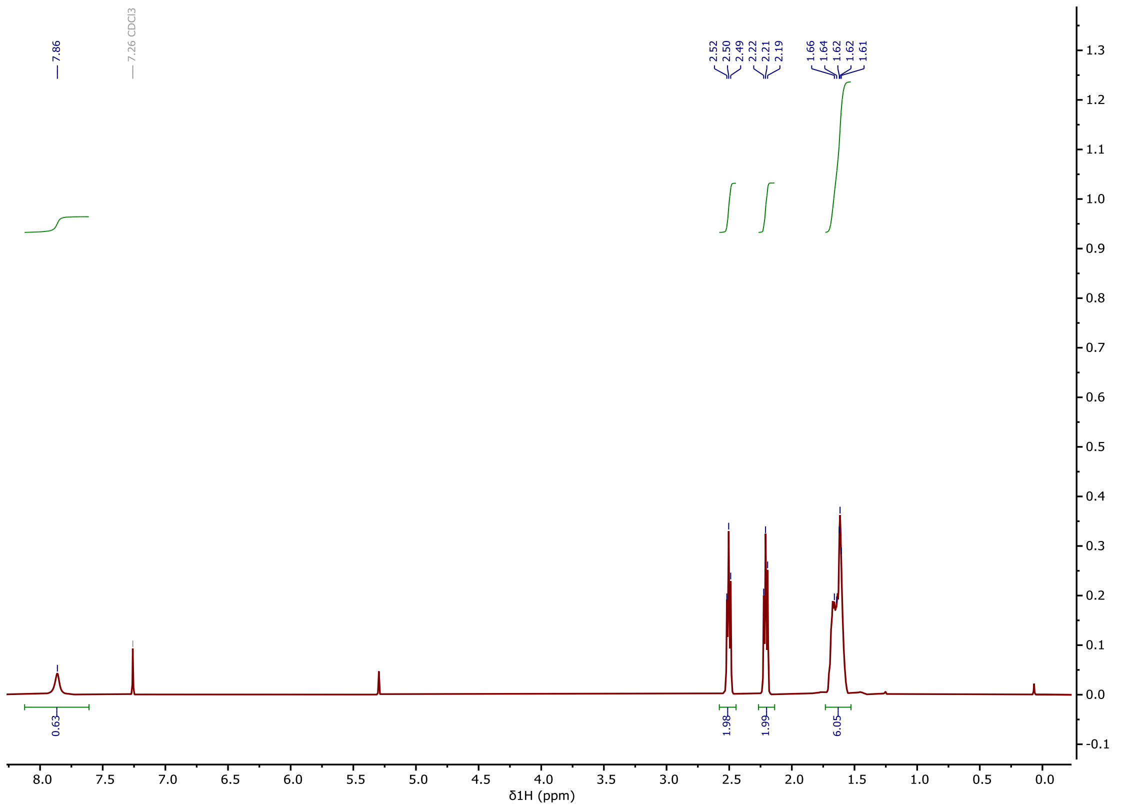

Figure S75: *^{1}H NMR spectrum of cyclohexanone oxime in $CDCl_3$ at 25 °C.*

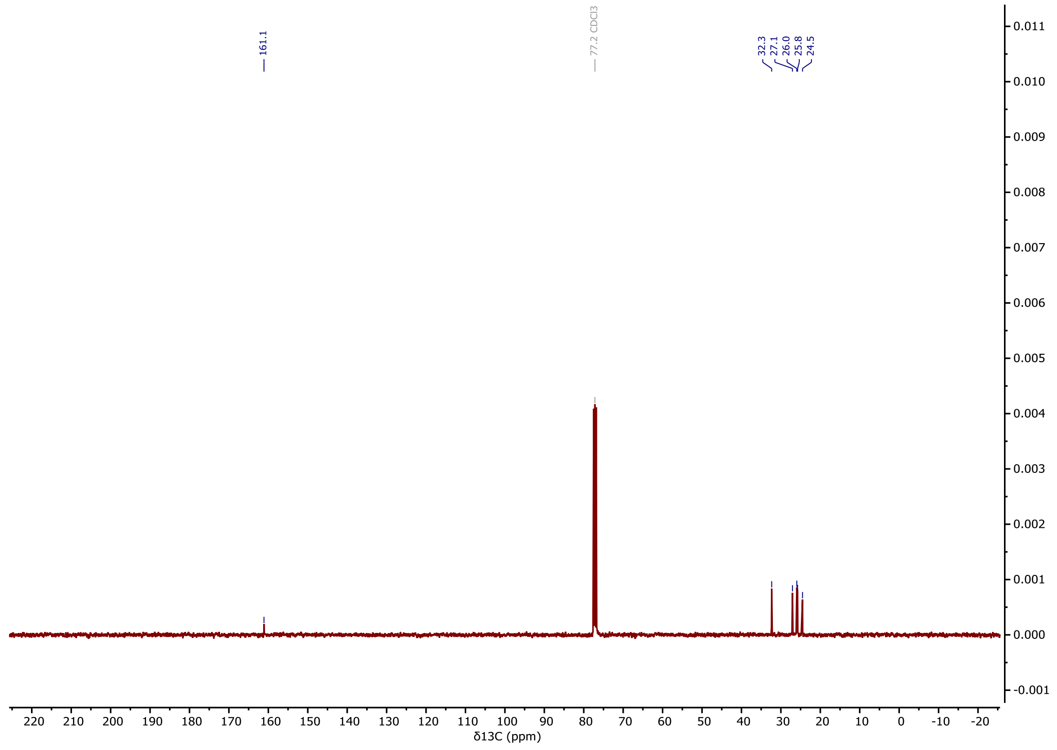

Figure S76: *^{13}C NMR spectrum of cyclohexanone oxime in $CDCl_3$ at 25 °C.*

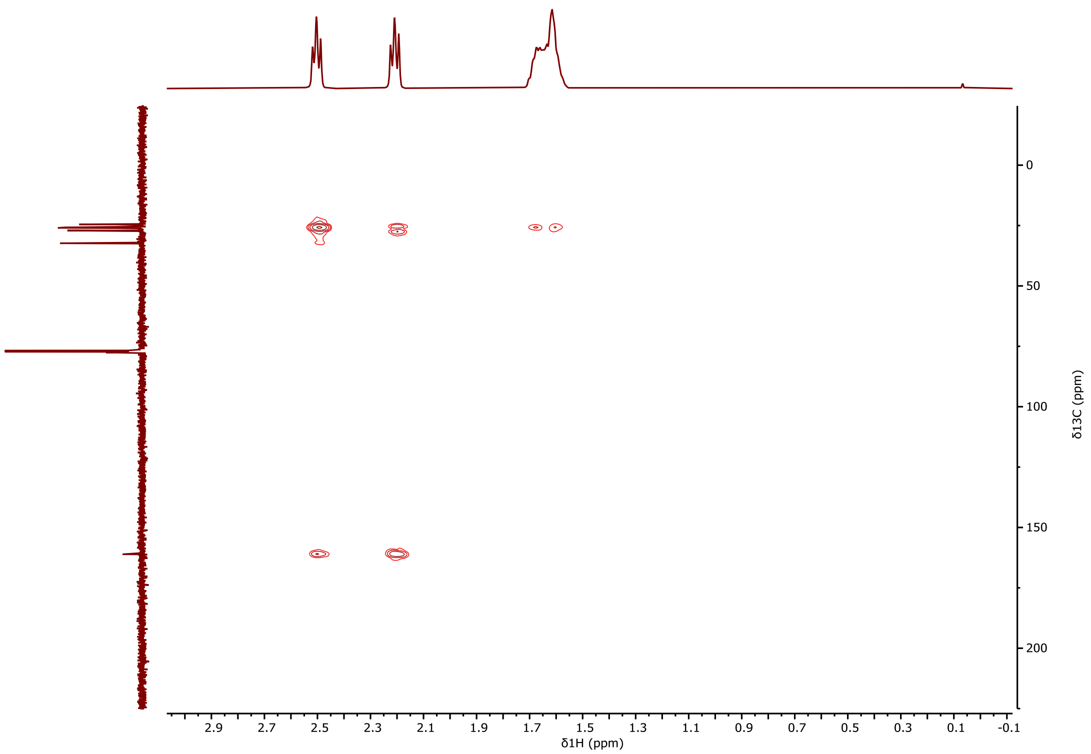

Figure S77: *^{1}H-[13]C Heteronuclear Multiple Bond Correlation (HMBC) spectrum of cyclohexanone oxime in $CDCl_3$ at 25 °C, with a zoom in on the coupling.*

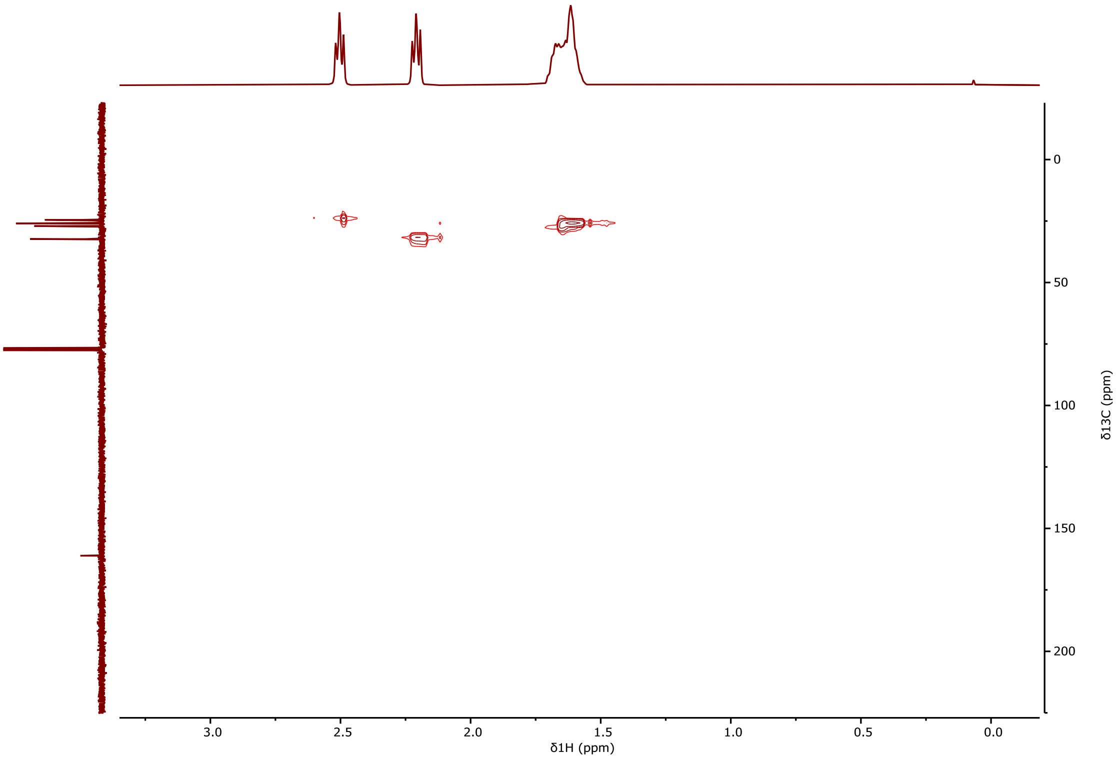

Figure S78: *^{1}H-[13]C Heteronuclear Single Quantum Coherence (HSQC) spectrum of cyclohexanone oxime in $CDCl_3$ at 25 °C, with a zoom in on the coupling.*

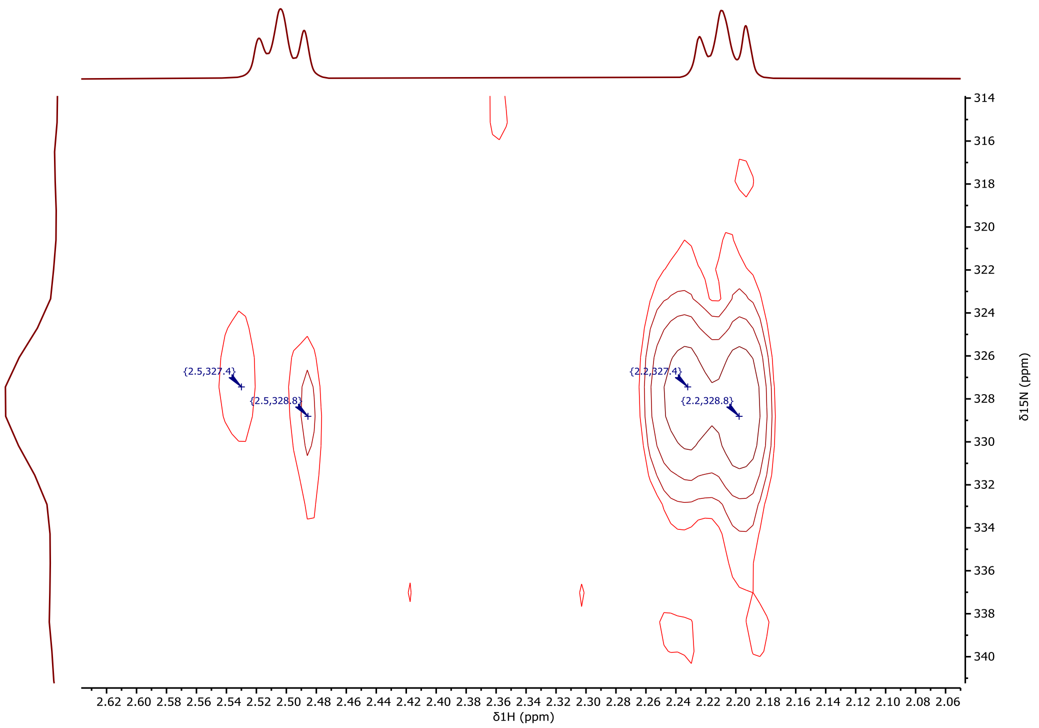

Figure S79: *^{1}H-[15]N Heteronuclear Multiple Bond Correlation (HMBC) spectrum of cyclohexanone oxime in $CDCl_3$ at 25 °C, with a zoom in on the coupling.*

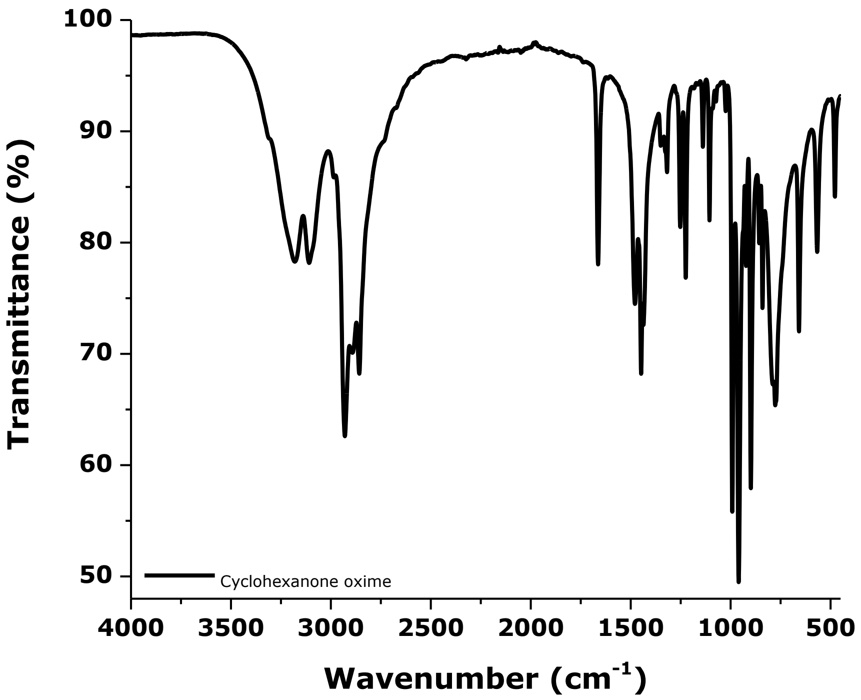

Figure S80: *FTIR (ATR) Spectrum of cyclohexanone oxime.*

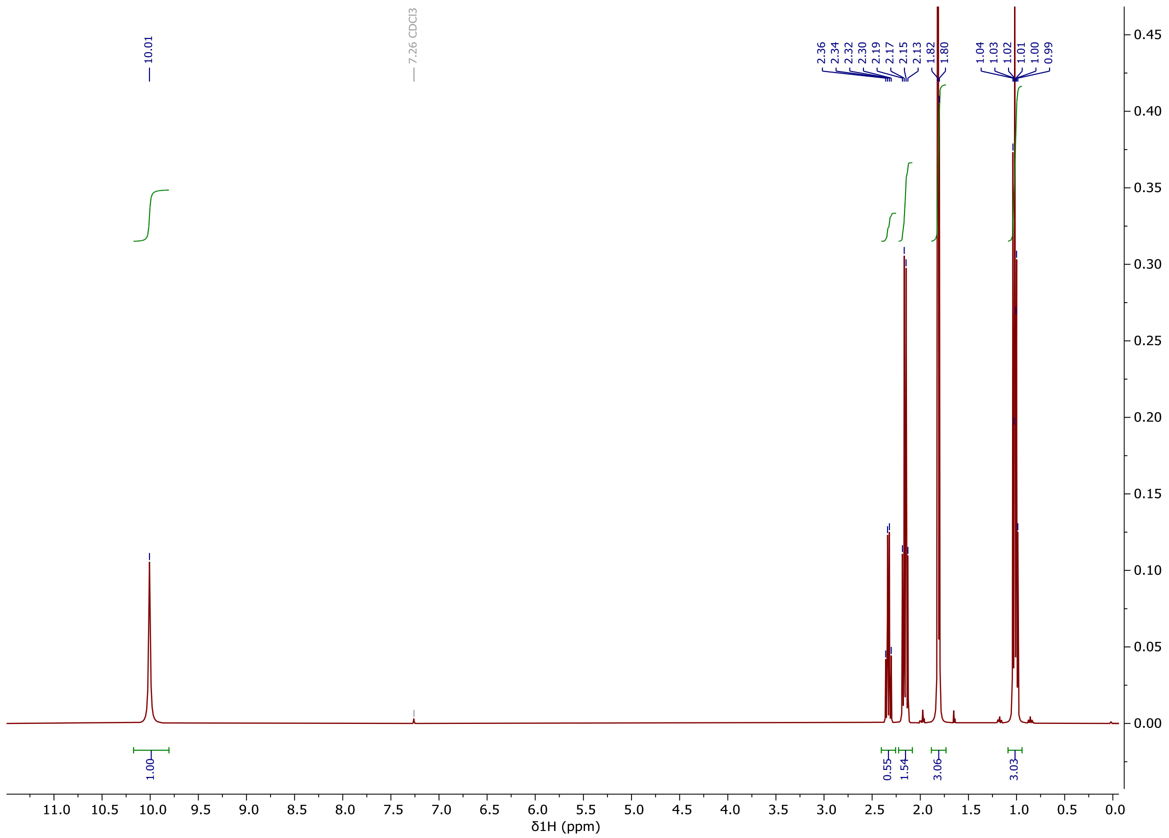

Figure S81: *^1H NMR Spectrum of 2-butanone oxime in $CDCl_3$ at 25 °C.*

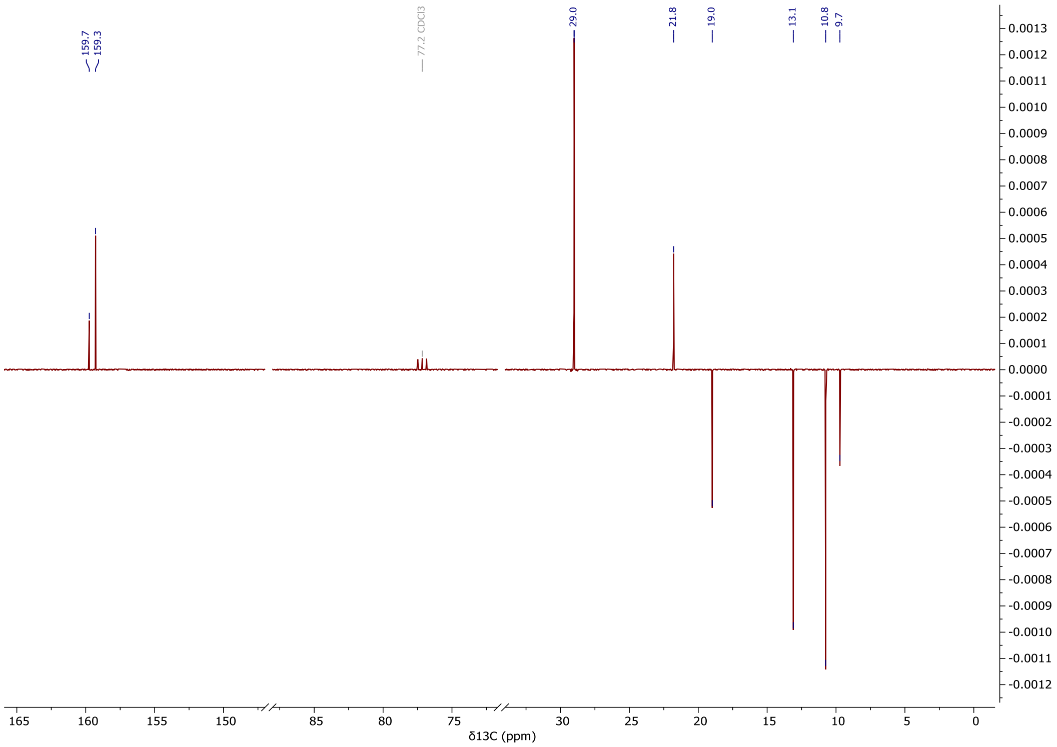

Figure S82: *^{13}C NMR (APT) Spectrum of 2-butanone oxime in $CDCl_3$ at 25 °C.*

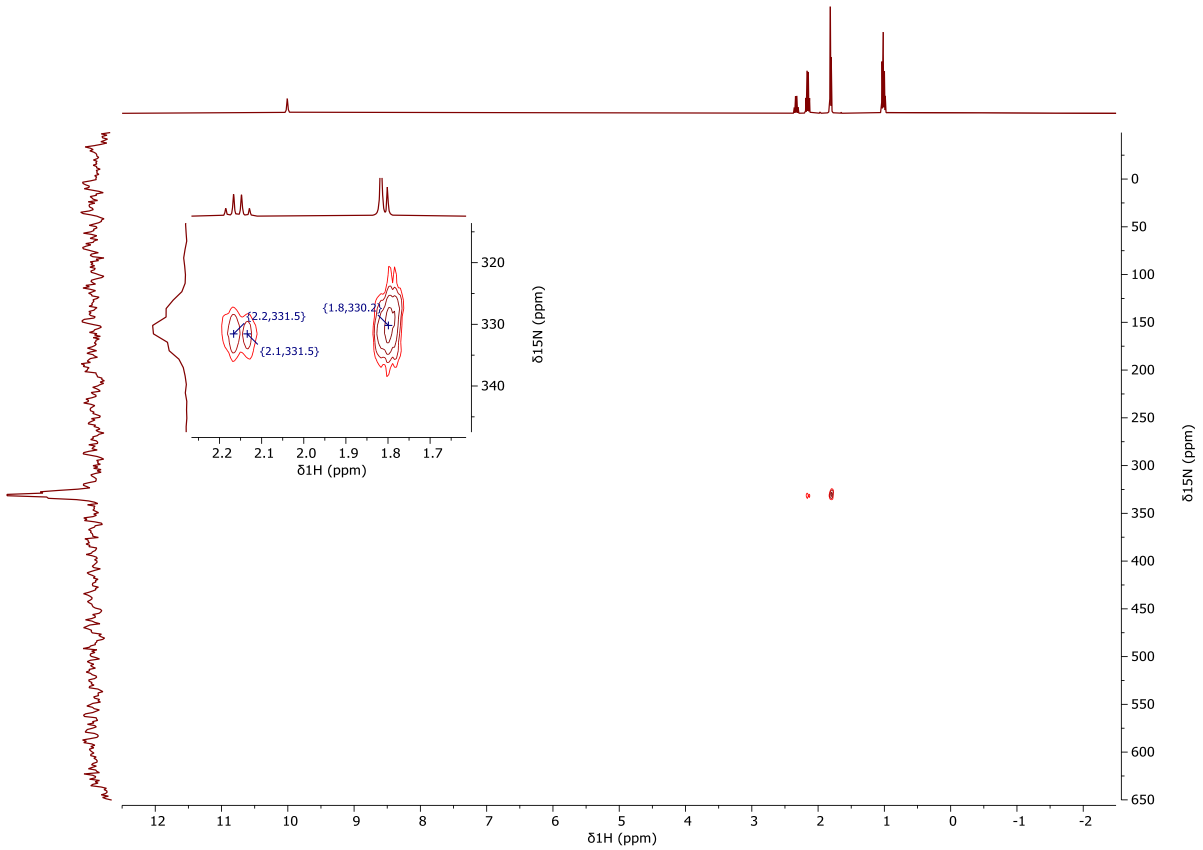

Figure S83: *^{1}H-^{15}N Heteronuclear Multiple Bond Correlation (HMBC) spectrum of 2-butanone oxime in $CDCl_3$ at 25 °C, with a zoom in on the coupling.*

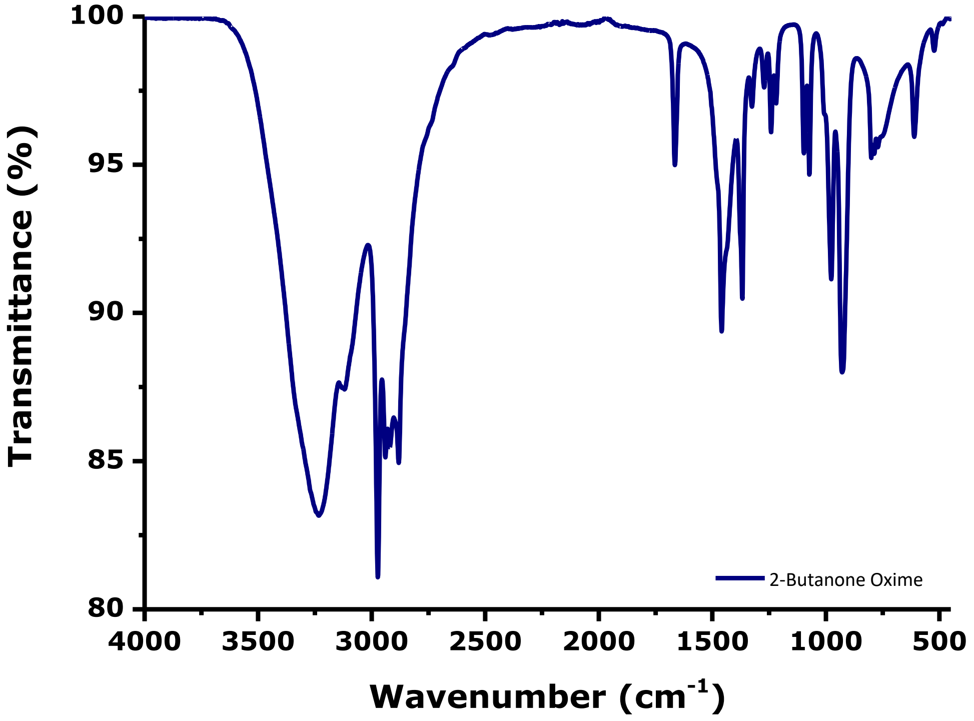

Figure S84: *FTIR (ATR) Spectrum of 2-butanone oxime.*

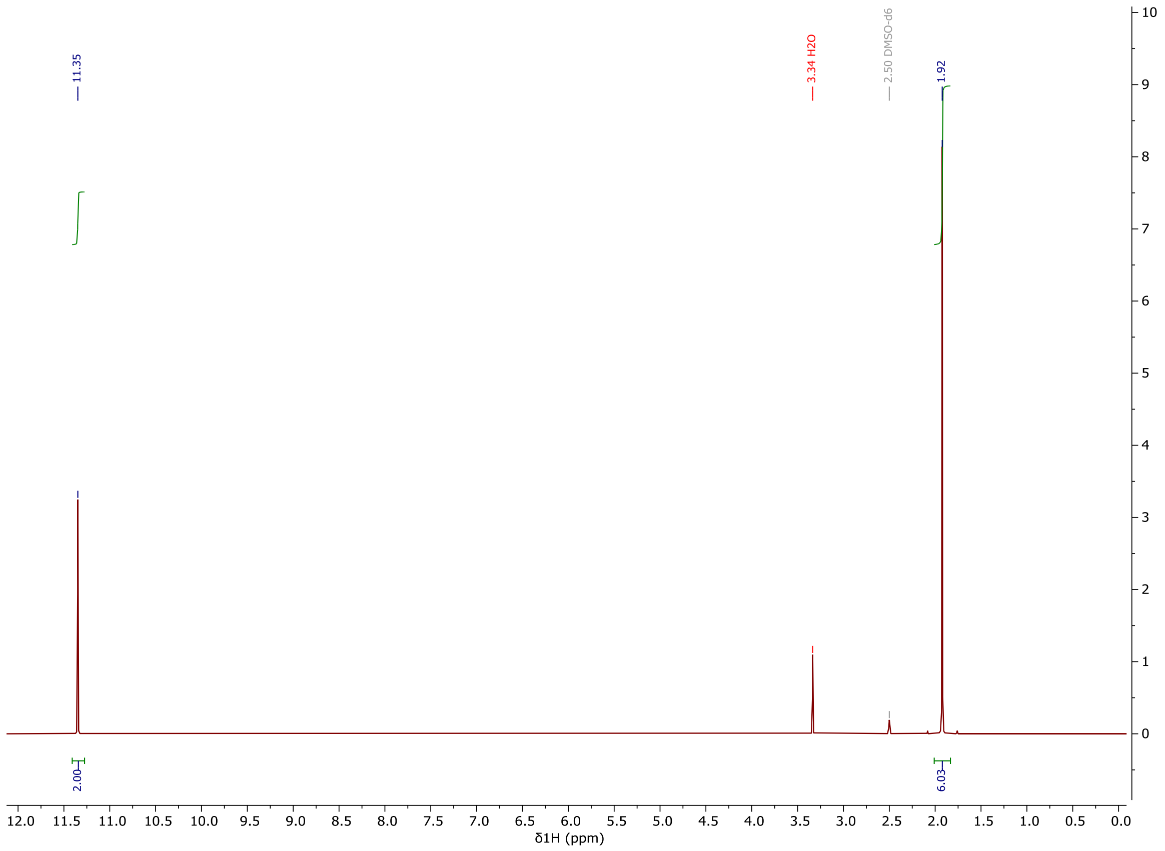

Figure S85: *^{1}H NMR Spectrum of dimethylglyoxime in DMSO-d_6 at 25°C.*

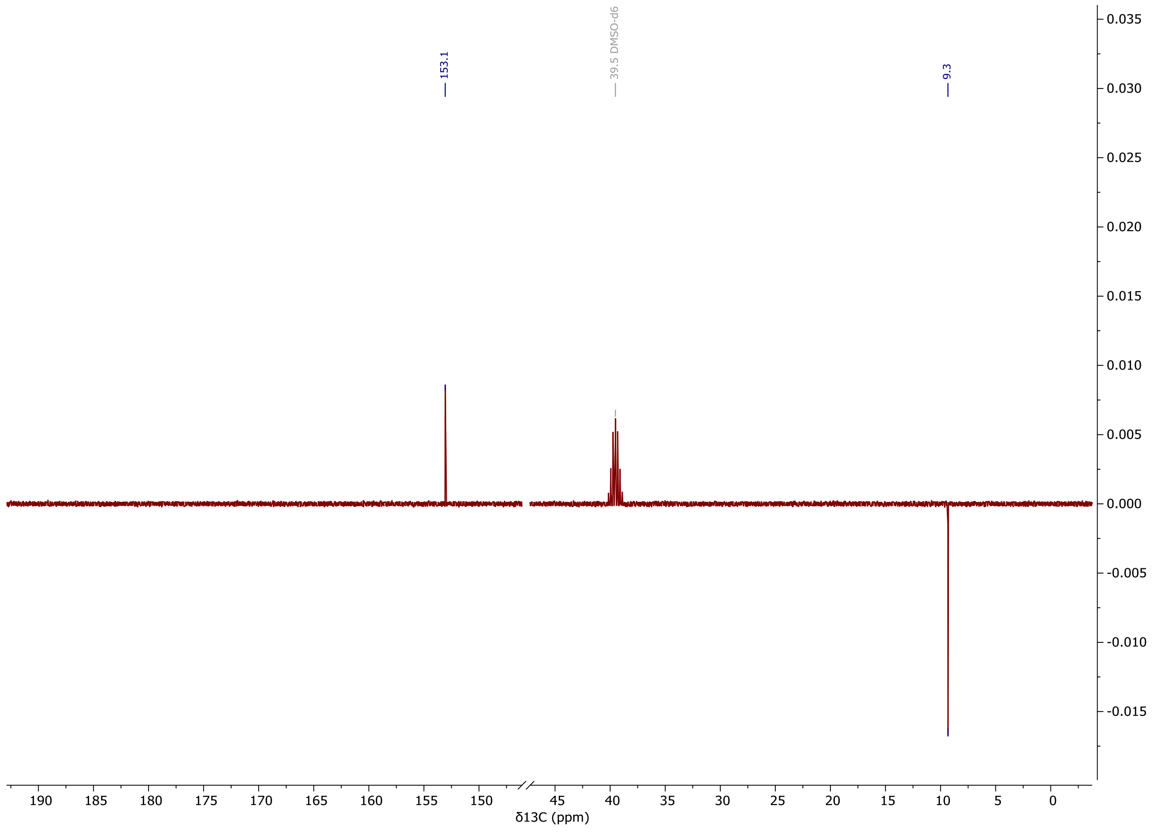

Figure S86: *^{13}C (APT) Spectrum of dimethylglyoxime in DMSO-d_6 at 25 °C.*

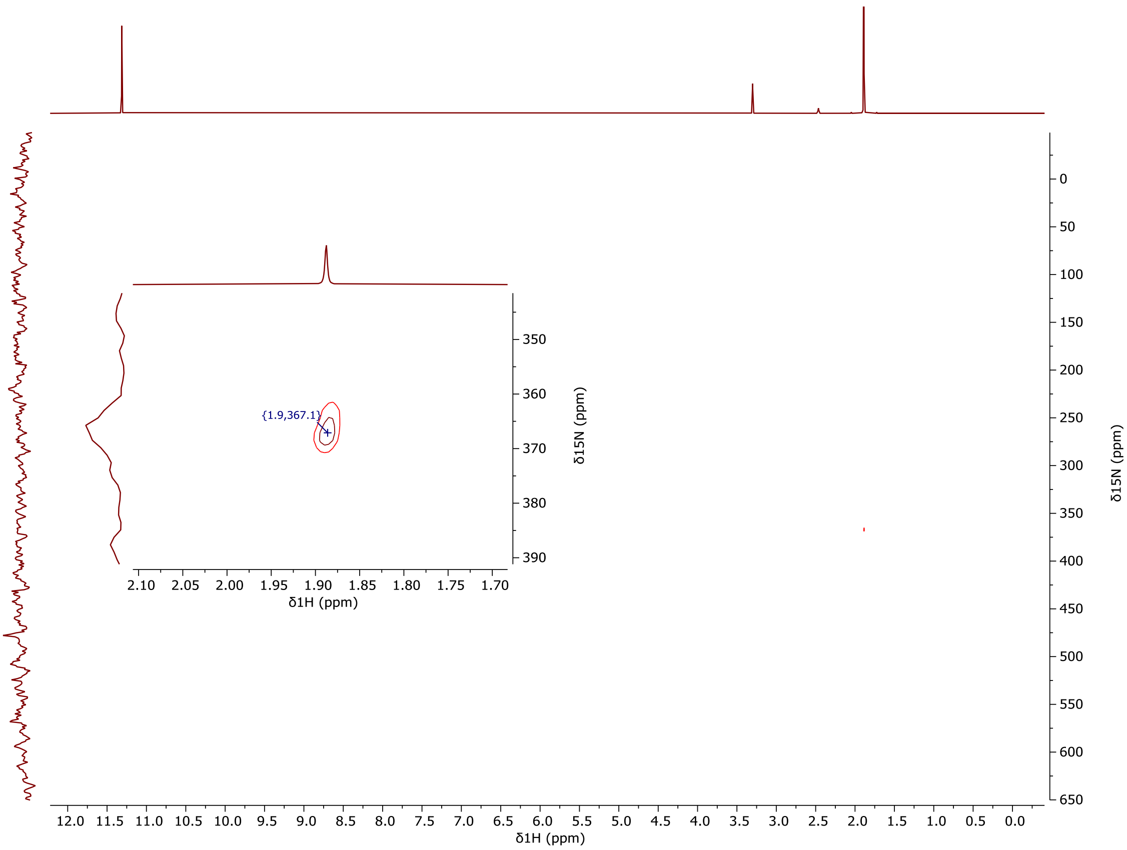

Figure S87: *^{1}H-^{15}N Heteronuclear Multiple Bond Correlation (HMBC) spectrum of dimethylglyoxime in DMSO-d_6 at 25°C, with a zoom in on the coupling.*

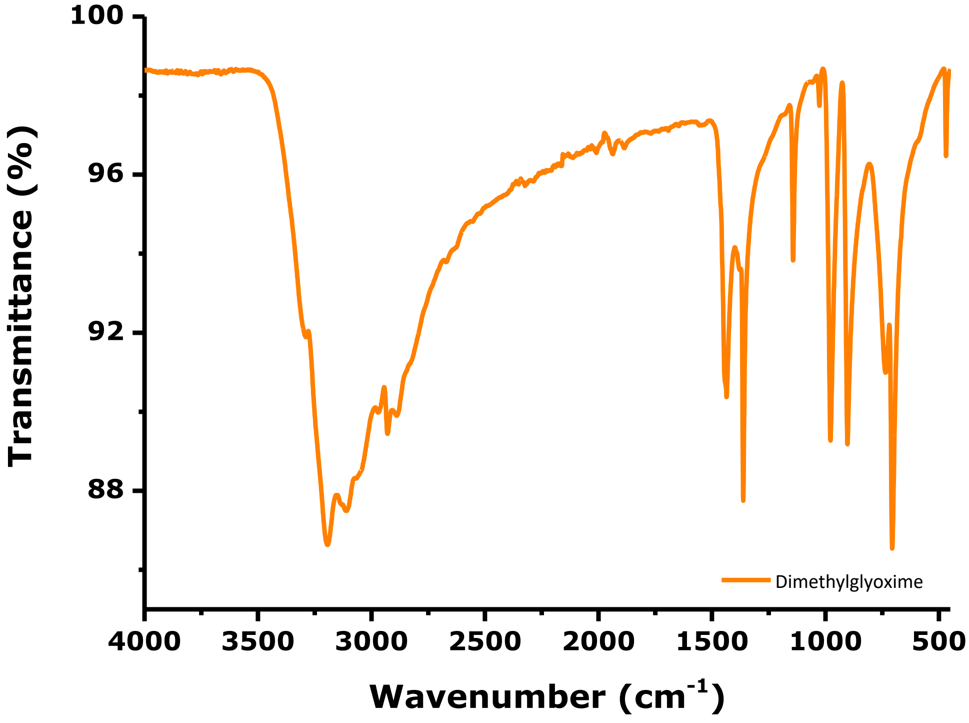

Figure S88: *FTIR (ATR) Spectrum of dimethylglyoxime.*

7. DSC and TGA Data

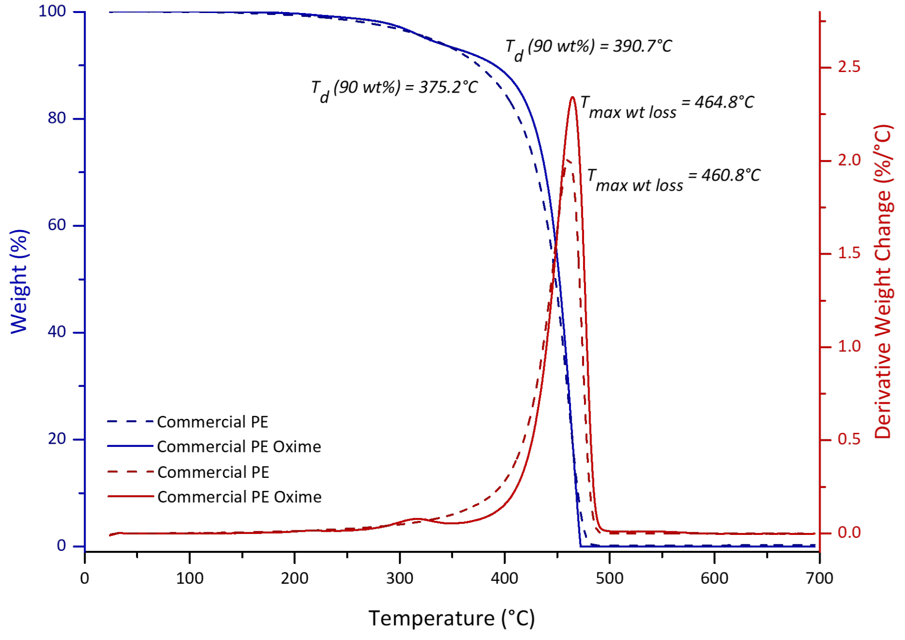

Figure S89: *Thermogravimetric Analysis (TGA) of Commercial Polyethylene (PE-C Low M_w) before and after photochemical oximation. The blue traces depict the weight loss percentage and the red traces depict the derivative weight change.*

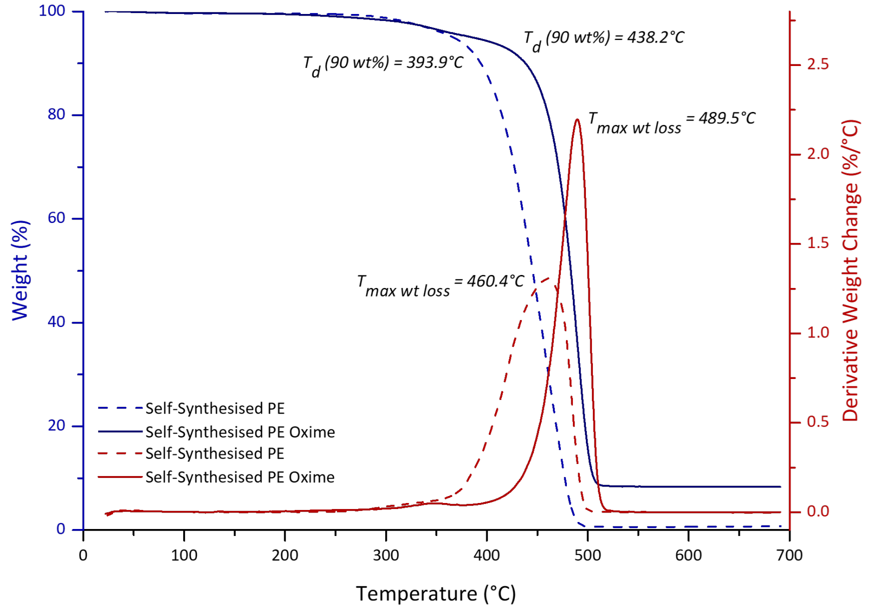

Figure S90: *Thermogravimetric Analysis (TGA) of the self synthesized polyethylene (PE-S) before and after the photochemical oximation. The blue trace depicts the weight loss percentage and the red trace depicts the derivative weight changes.*

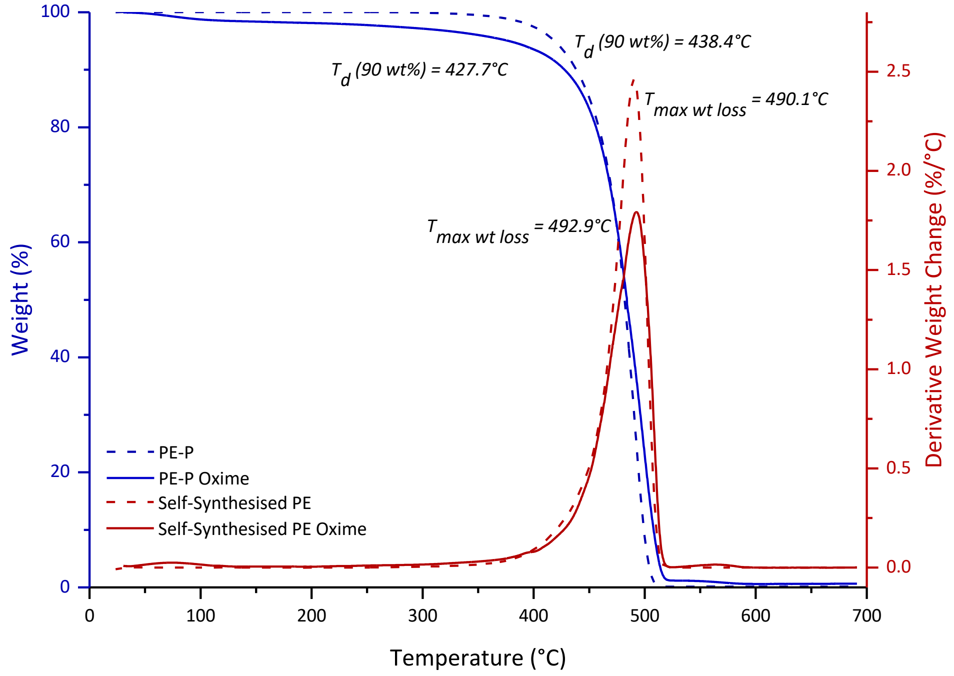

Figure S91: *Thermogravimetric Analysis (TGA) of the post-consumer polyethylene (PE-P) before and after the photochemical oximation. The blue trace depicts the weight loss percentage and the red trace depicts the derivative weight changes.*

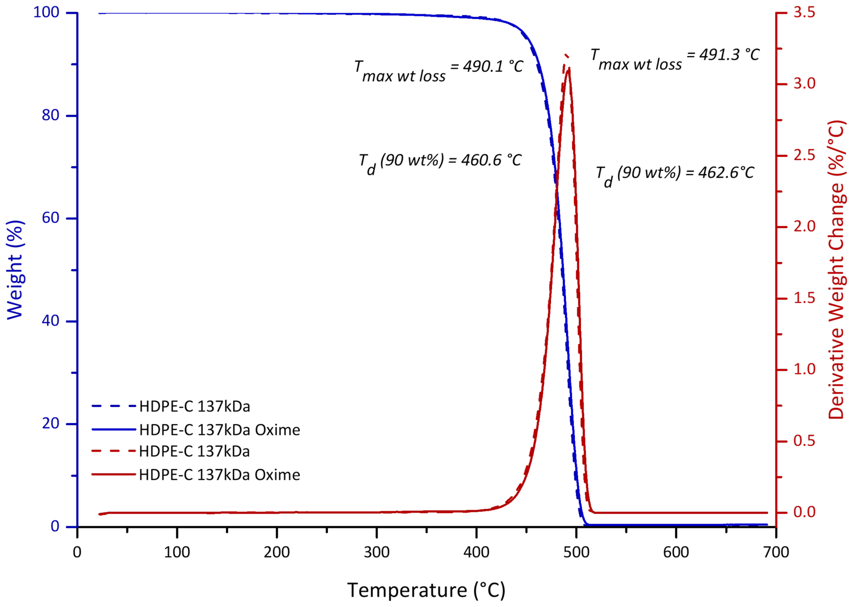

Figure S92: *Thermogravimetric Analysis (TGA) of the High molecular weight HDPE before and after the photochemical oximation. The blue trace depicts the weight loss percentage and the red trace depicts the derivative weight changes.*

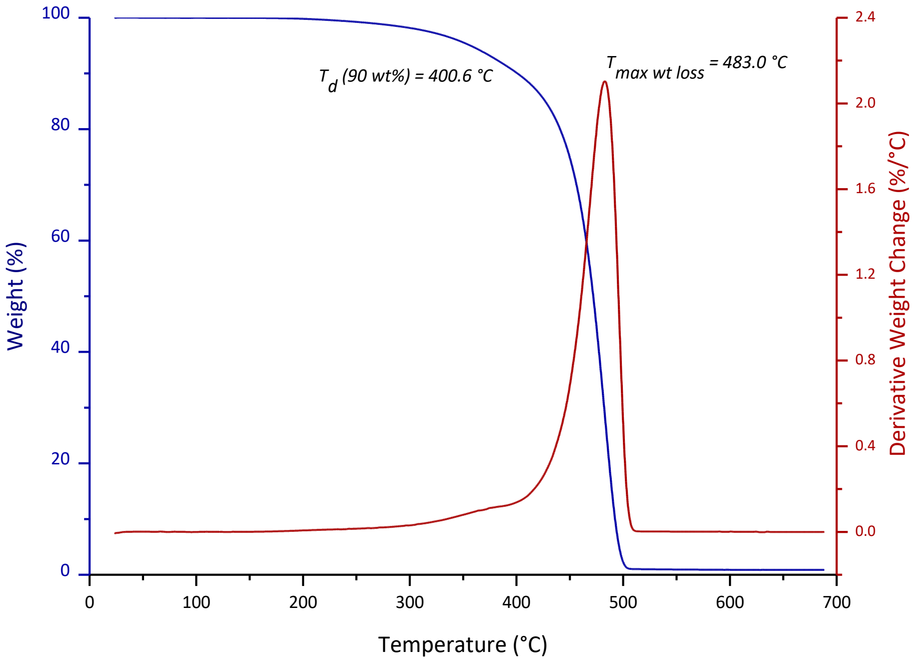

Figure S93: *Thermogravimetric Analysis (TGA) of the commercial vendor polyethylene (PE-C Low M_w) after the photochemical oximation with an open reflux set up to obtain only ketone. The blue trace depicts the weight loss percentage and the red trace depicts the derivative weight changes.*

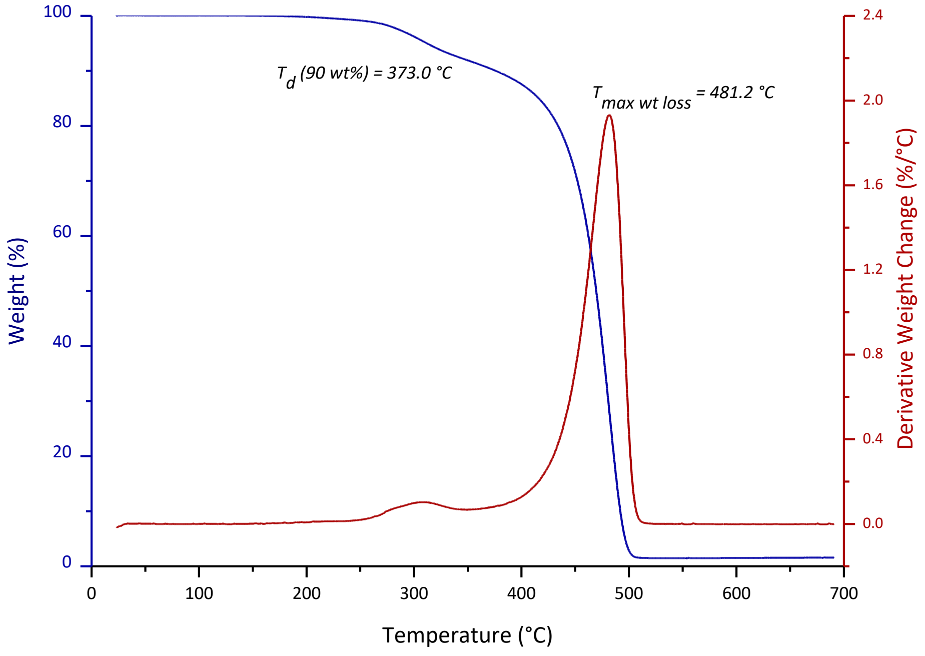

Figure S94: *Thermogravimetric Analysis (TGA) of the commercial vendor polyethylene (PE-C Low M_w) after the one-pot photochemical oximation and the reaction with hydroxylamine hydrochloride in pyridine. The blue trace depicts the weight loss percentage and the red trace depicts the derivative weight changes.*

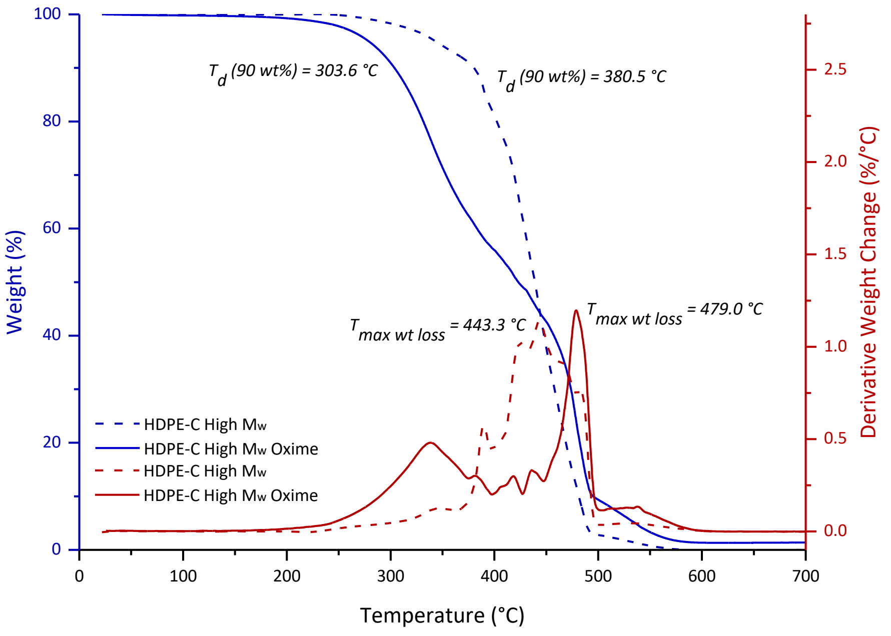

Figure S95: *Thermogravimetric Analysis (TGA) of the commercial vendor polyethylene (HDPE-C) before and after the photochemical oximation. The blue trace depicts the weight loss percentage and the red trace depicts the derivative weight changes.*

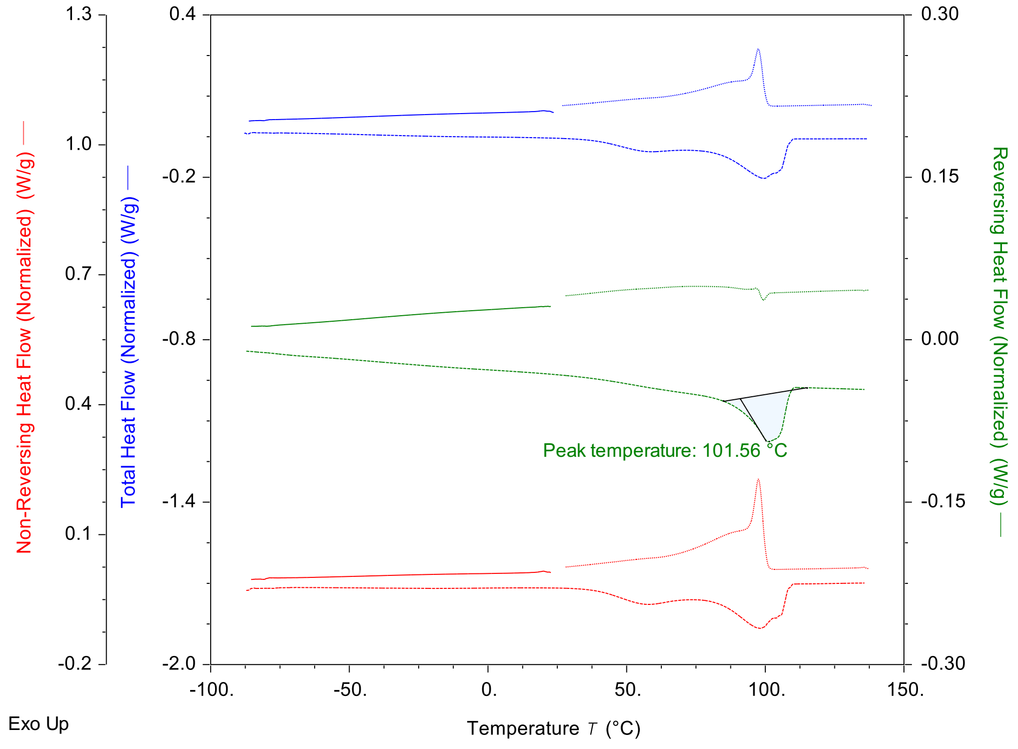

Figure S96: *Differential Scanning Calorimetry (DSC) of the commercial vendor polyethylene (PE-C Low M_w). The Blue trace depicts the normalized total heat flow, the green trace depicts the normalized reversing heat flow, and the red trace depicts the normalized non-reversed heat flow.*

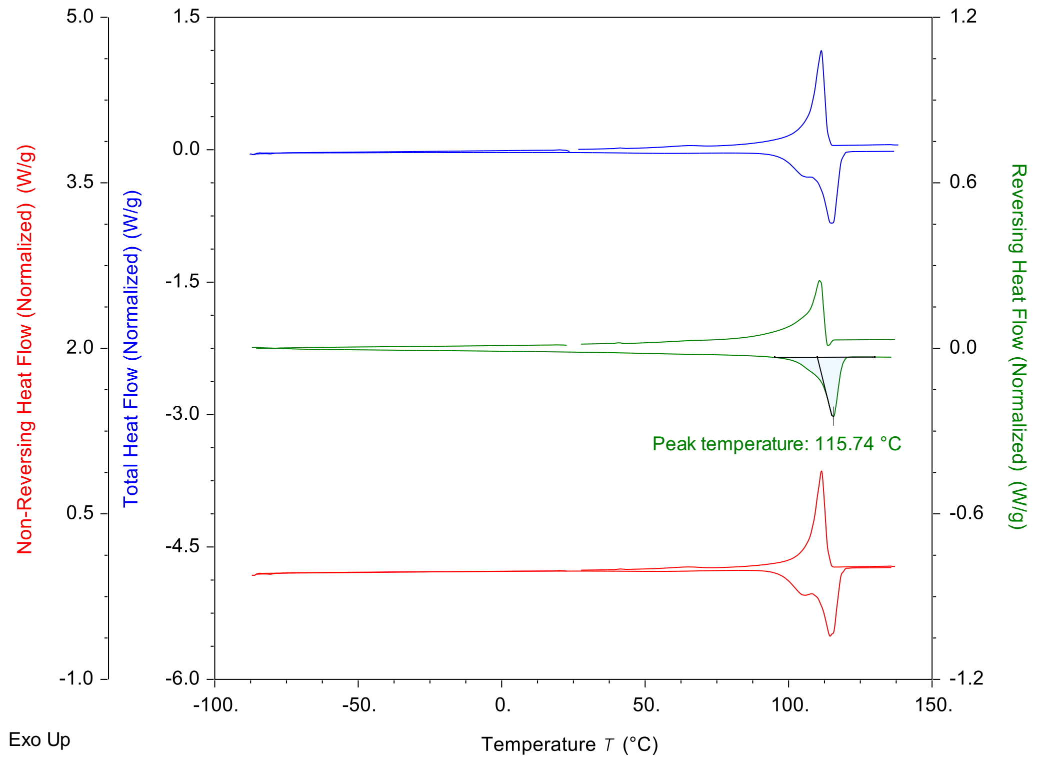

Figure S97: *Differential Scanning Calorimetry (DSC) of the self synthesized polyethylene (PE-S). The Blue trace depicts the normalized total heat flow, the green trace depicts the normalized reversing heat flow, and the red trace depicts the normalized non-reversed heat flow.*

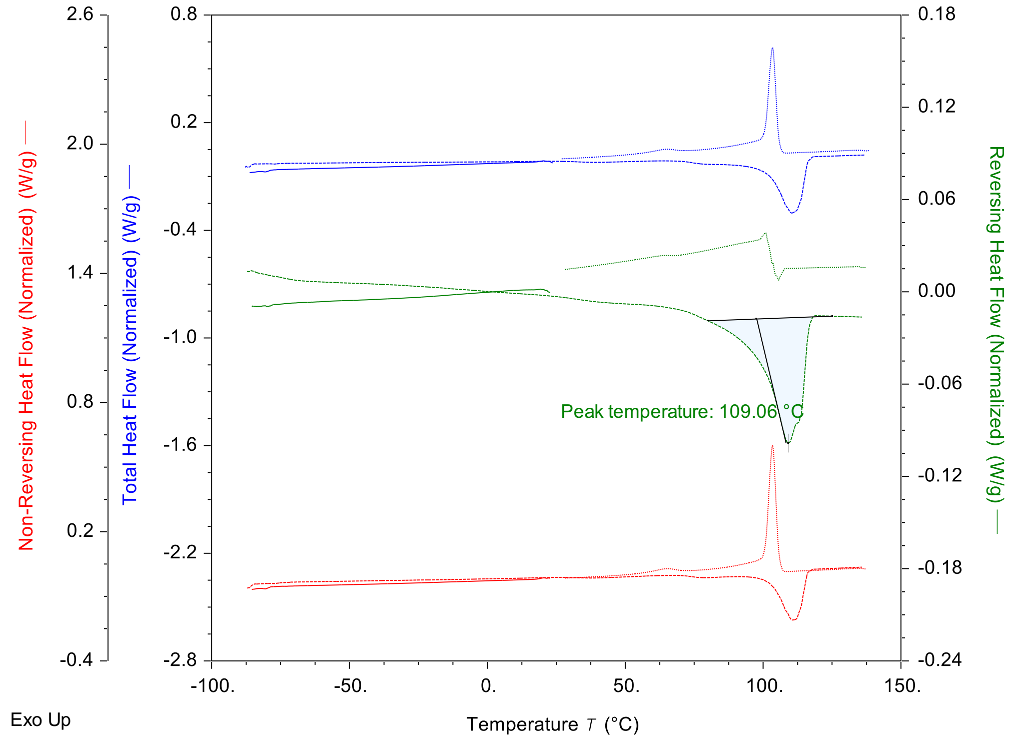

Figure S98: *Differential Scanning Calorimetry (DSC) of the post consumer polyethylene (PE-P). The Blue trace depicts the normalized total heat flow, the green trace depicts the normalized reversing heat flow, and the red trace depicts the normalized non-reversed heat flow.*

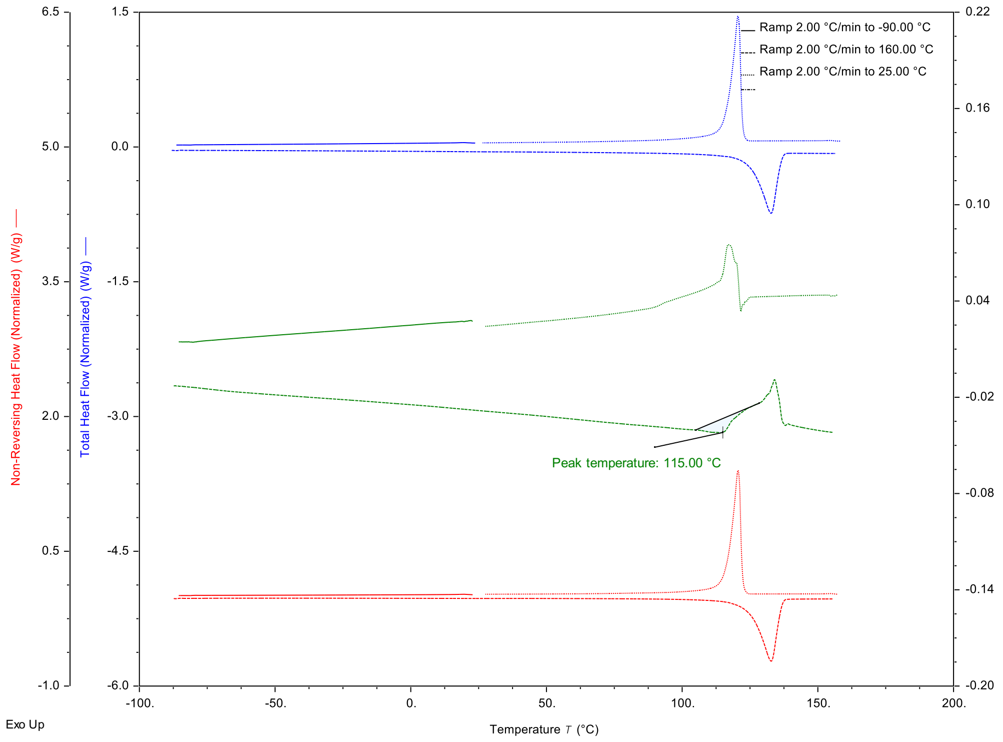

Figure S99: *Differential Scanning Calorimetry (DSC) of the high density polyethylene (HDPE-C). The Blue trace depicts the normalized total heat flow, the green trace depicts the normalized reversing heat flow, and the red trace depicts the normalized non-reversed heat flow.*

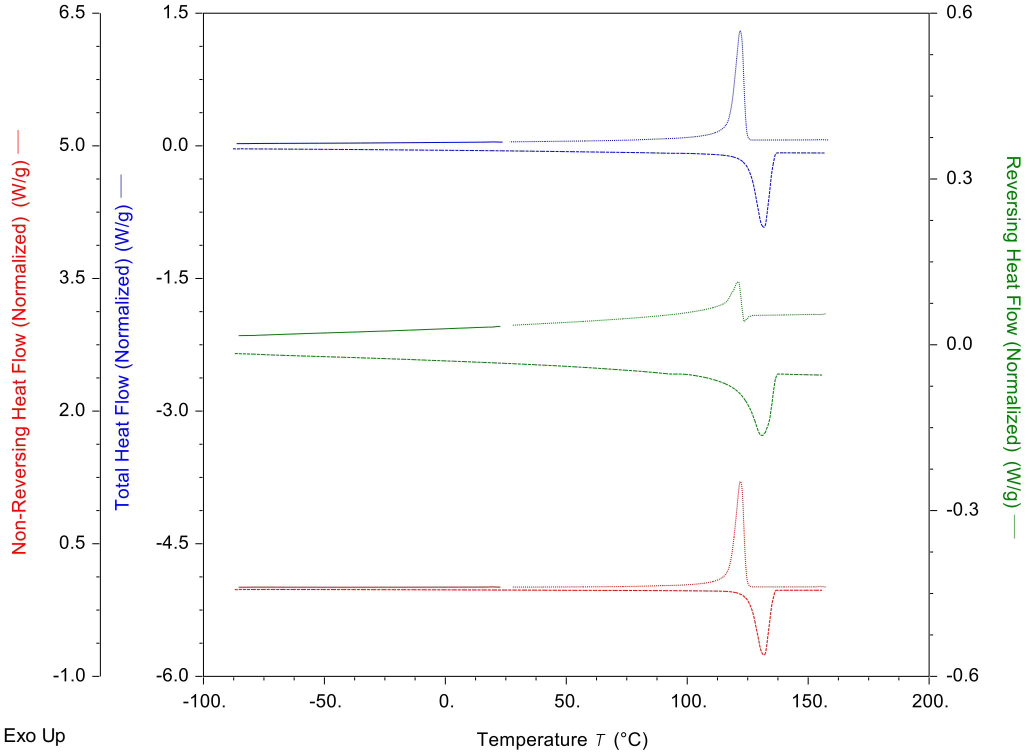

Figure S100: *Differential Scanning Calorimetry (DSC) of the high density polyethylene (HDPE-C High M_w). The Blue trace depicts the normalized total heat flow, the green trace depicts the normalized reversing heat flow, and the red trace depicts the normalized non-reversed heat flow.*

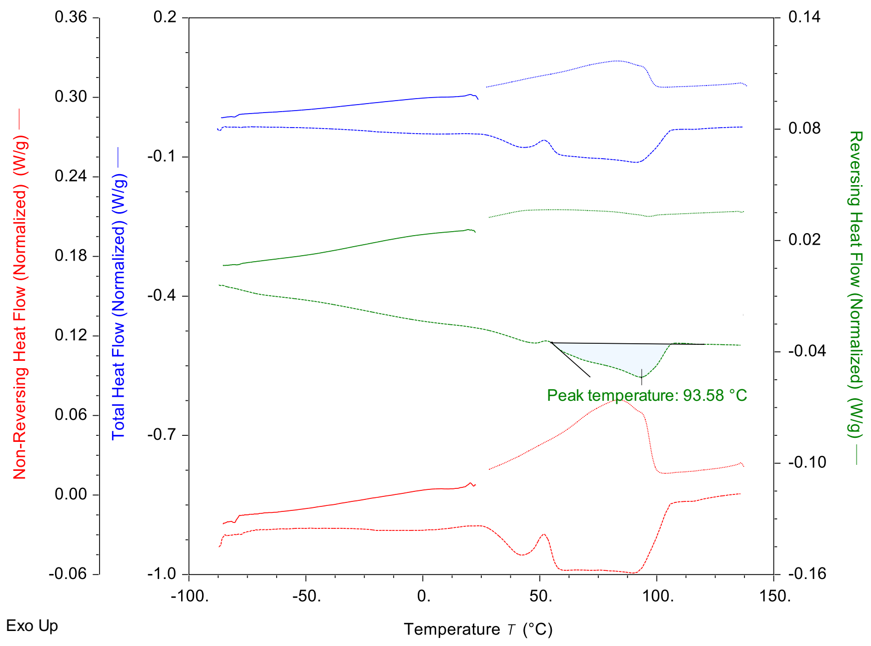

Figure S101: *Differential Scanning Calorimetry (DSC) of the post consumer polyethylene (PE-C Low M_w) after the photochemical oximation. The Blue trace depicts the normalized total heat flow, the green trace depicts the normalized reversing heat flow, and the red trace depicts the normalized non-reversed heat flow.*

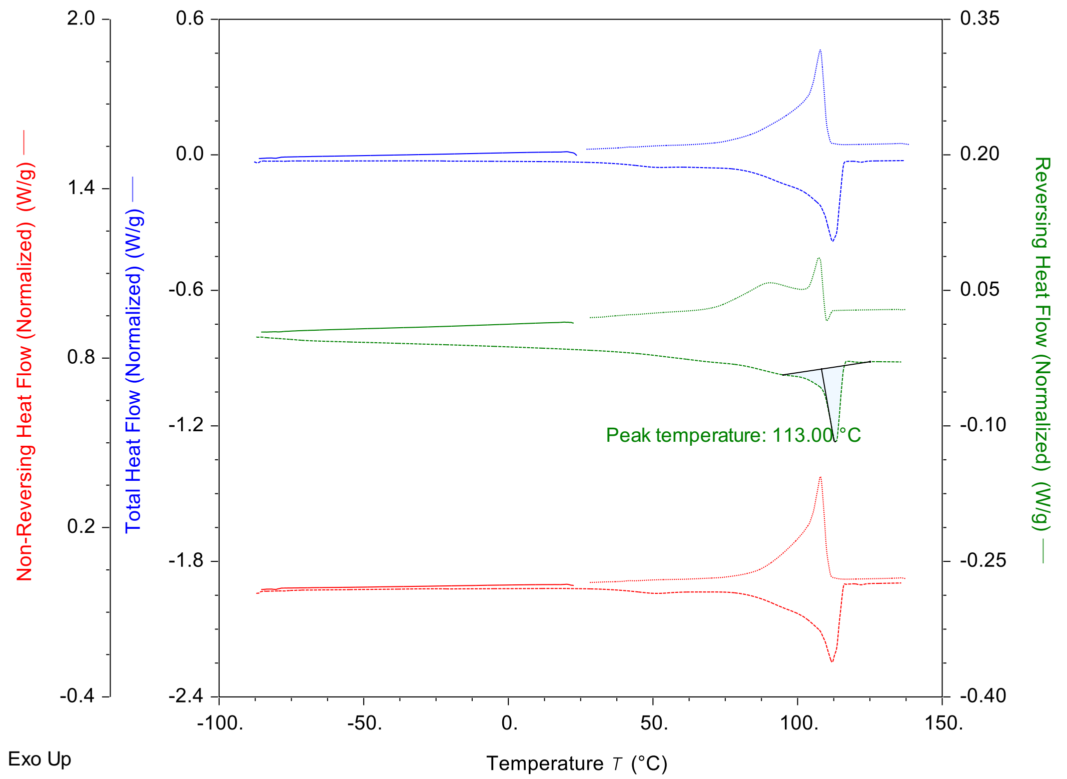

Figure S102: *Differential Scanning Calorimetry (DSC) of the self synthesized polyethylene (PE-S) after the photochemical oximation. The Blue trace depicts the normalized total heat flow, the green trace depicts the normalized reversing heat flow, and the red trace depicts the normalized non-reversed heat flow.*

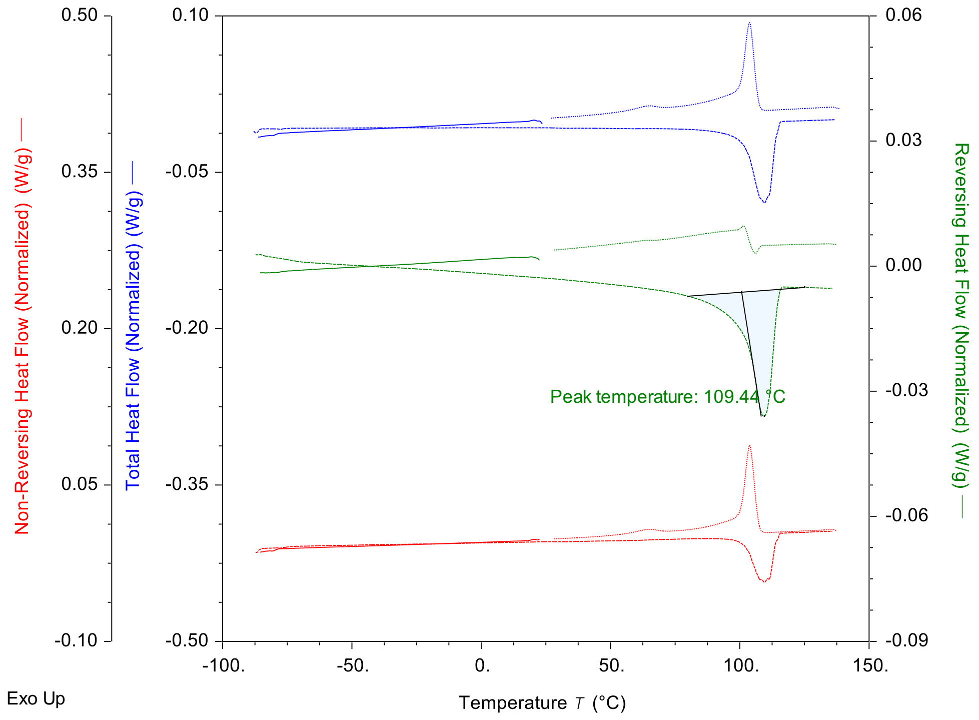

Figure S103: *Differential Scanning Calorimetry (DSC) of the consumer polyethylene (PE-P) after the photochemical oximation. The Blue trace depicts the normalized total heat flow, the green trace depicts the normalized reversing heat flow, and the red trace depicts the normalized non-reversed heat flow.*

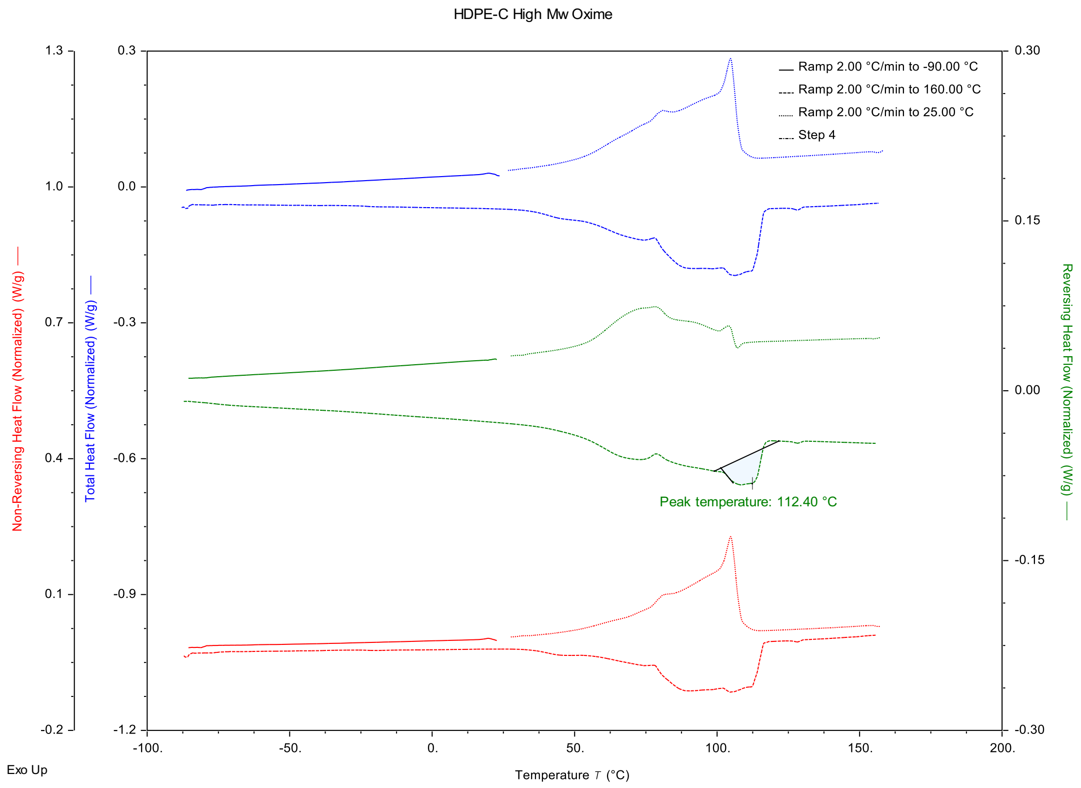

Figure S104: *Differential Scanning Calorimetry (DSC) of the consumer polyethylene (HDPE-C) after the photochemical oximation. The Blue trace depicts the normalized total heat flow, the green trace depicts the normalized reversing heat flow, and the red trace depicts the normalized non-reversed heat flow.*

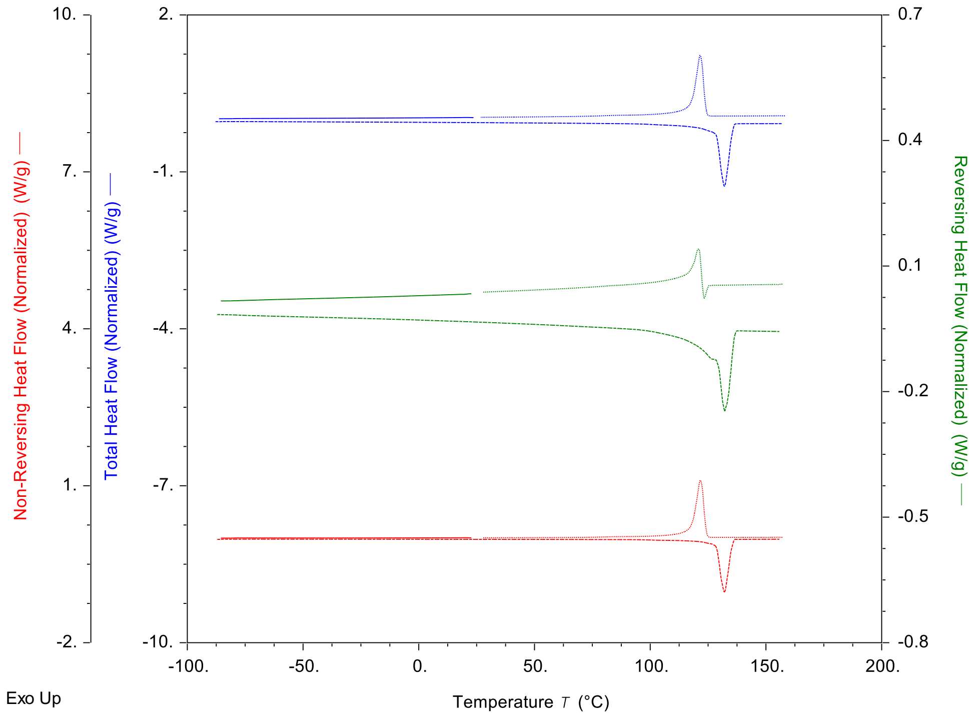

Figure S105: *Differential Scanning Calorimetry (DSC) of the consumer polyethylene (HDPE-C High M_w) after the photochemical oximation. The Blue trace depicts the normalized total heat flow, the green trace depicts the normalized reversing heat flow, and the red trace depicts the normalized non-reversed heat flow.*

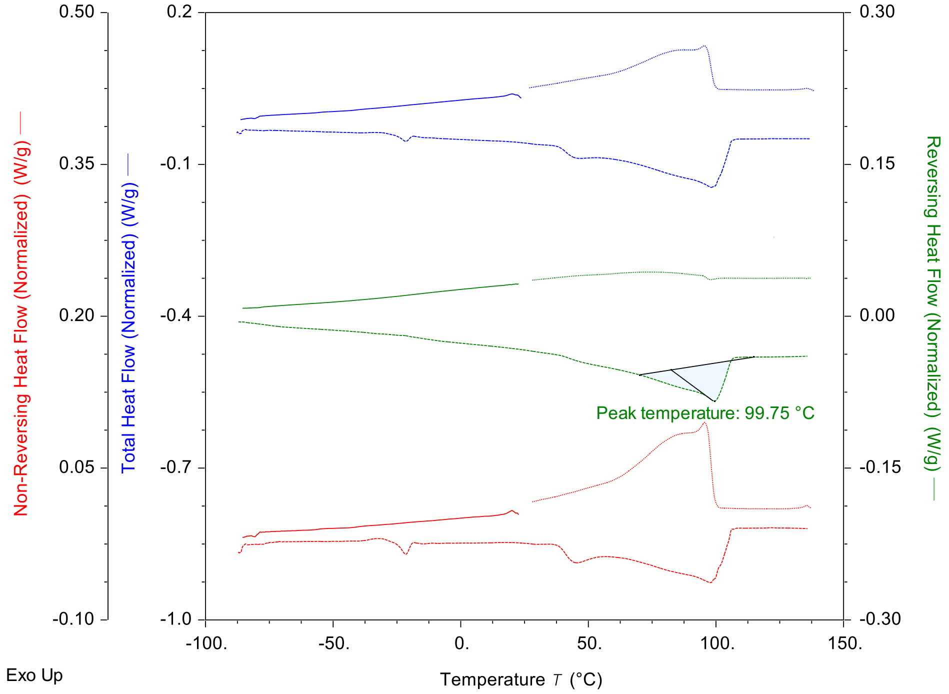

Figure S106: *Differential Scanning Calorimetry (DSC) of the commercial vendor polyethylene (PE-C Low M_w) after the one-pot the photochemical oximation and the reaction with hydroxylamine hydrochloride in pyridine. The Blue trace depicts the normalized total heat flow, the green trace depicts the normalized reversing heat flow, and the red trace depicts the normalized non-reversed heat flow.*

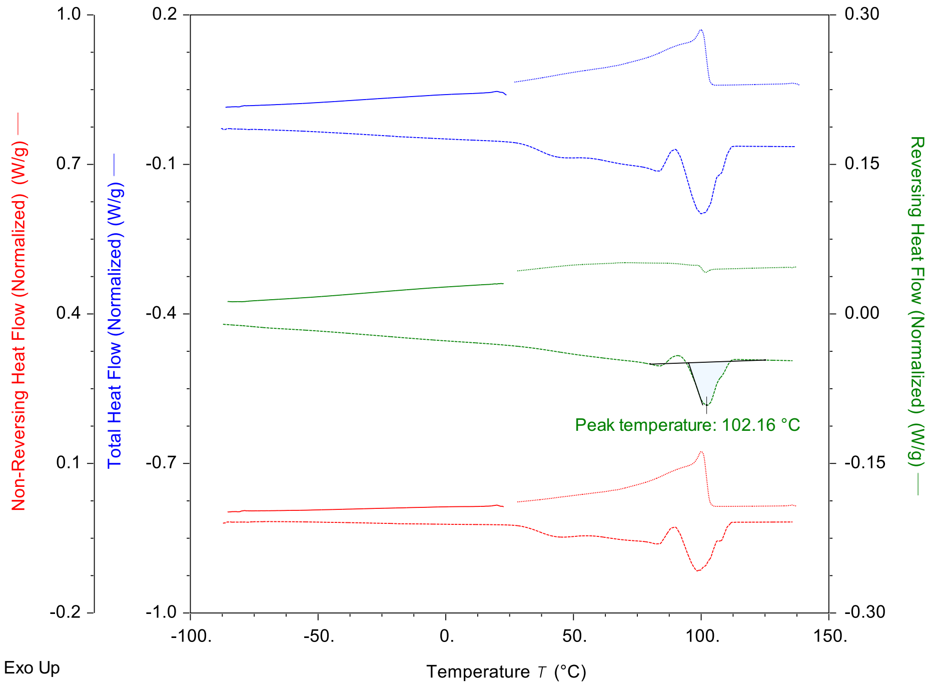

Figure S107: *Differential Scanning Calorimetry (DSC) of the commercial vendor polyethylene (PE-C Low M_w) after the photochemical oximation with an open reflux set-up. The Blue trace depicts the normalized total heat flow, the green trace depicts the normalized reversing heat flow, and the red trace depicts the normalized non-reversed heat flow.*

8. GPC Data

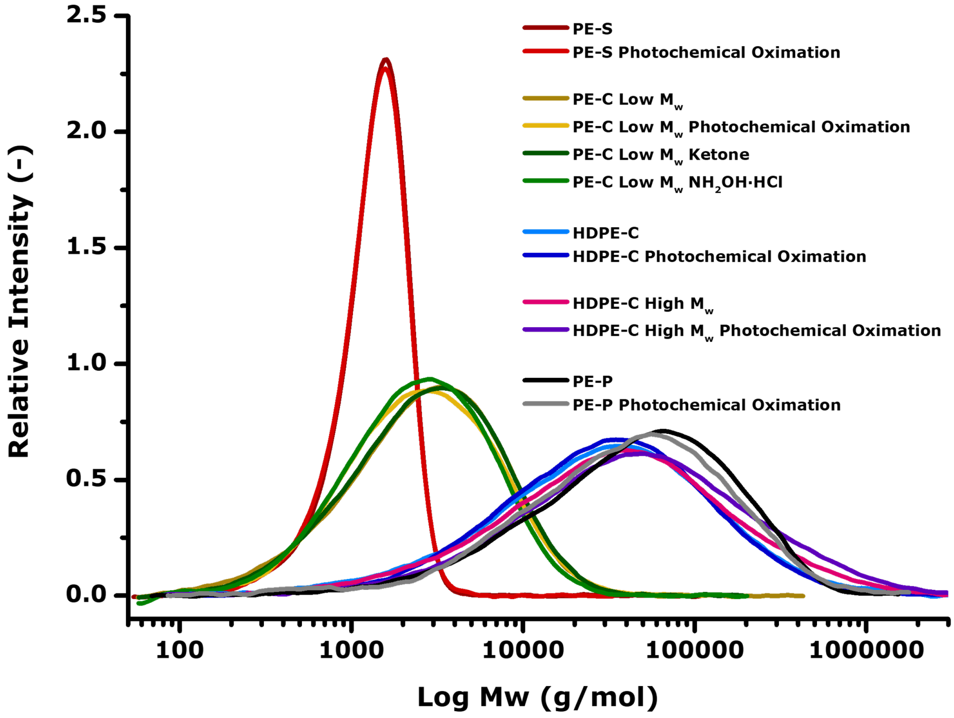

Figure S108: *Gel Permeation Chromatography (GPC) traces of the self synthesized polyethylene (PE-S), low molecular weight commercial vendor polyethylene (PE-C Low M_w), HDPE commercial vendor polyethylene (HDPE-C), higher molecular weight HDPE commercial vendor polyethylene (HDPE-C High M_w) and post consumer polyethylene (PE-P) before and after the photochemical oximation.*

Table S3: *Overview of the GPC data of all PE materials used for the photochemical oximation, with the weight average molecular weight (M_w) and number average molecular weight (M_n) in kDa and the corresponding dispersity index (Đ). Molecular weights are reported against polystyrene standards.*

**Polymer**	**M_w (kDa)**	**M_n (kDa)**	**Đ (M_w/M_n)**
PE-S	1.5	1.1	1.28
PE-S Photochemical Oximation	1.4	1.1	1.30
PE-C Low M_w	4.2	1.4	2.93
PE-C Low M_w Photochemical Oximation	4.1	1.5	2.76
PE-C Low M_w Ketone	4.3	1.5	2.83
PE-C Low M_w $NH_2OH \cdot HCl$	3.8	1.6	2.41
HDPE-C	76.6	6.9	11.15
HDPE-C Photochemical Oximation	75.2	9.4	8.04
HDPE-C High M_w	114.2	9.4	12.15
HDPE-C High M_w Photochemical Oximation	134.3	9.6	14.00
PE-P	87.3	12.2	7.15
PE-P Photochemical Oximation	86.7	9.4	9.27

9. Mechanical Properties

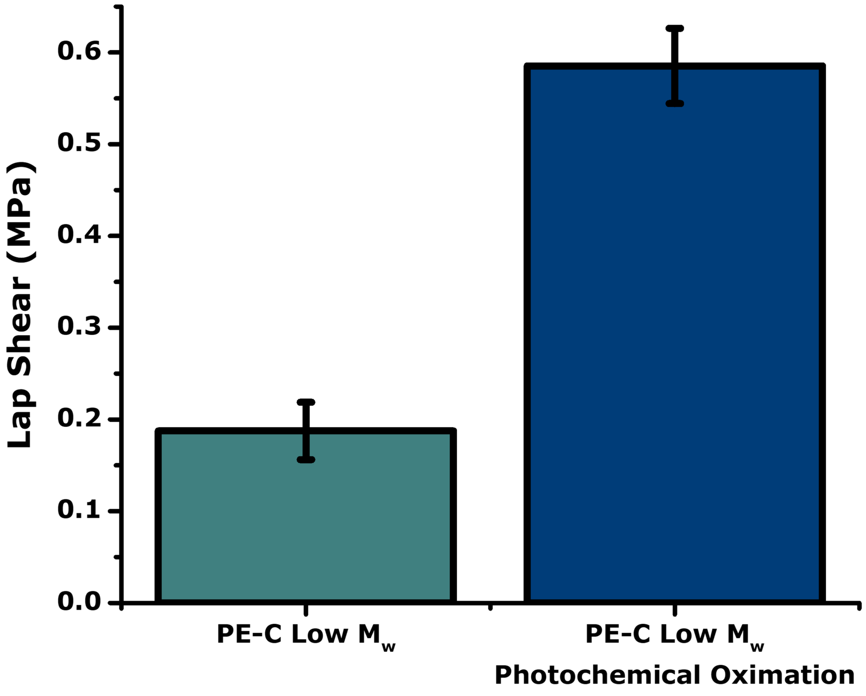

Figure S109: *Lap-shear strength of polycarbonate substrates glued with PE-C Low M_w and PE-C Low M_w Photochemical Oximation.*

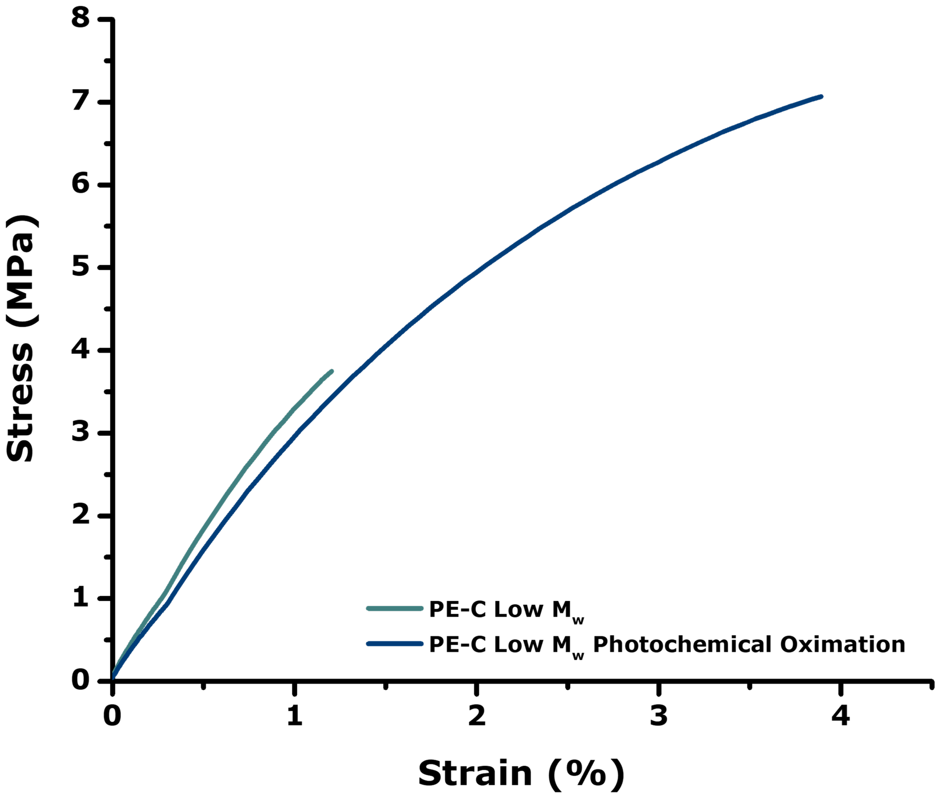

Figure S110: *Stress-strain curves of PE-C Low M_w and PE-C Low M_w Photochemical Oximation.*

10. Data Of Iminoxyl Radical Investigation

All oximes have been reaction with 1 equivalent of *tert*-butyl nitrite under irradiation with the 370 m UVA Light. This resulted in a color change from light yellow to turquoise or blue. From the resulting product and EPR was taken at ambient temperature. The obtained EPR spectra were thereafter also simulated with EasySpin in MATLAB using Garlic.

t = 0 sec 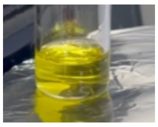 t = 30 sec 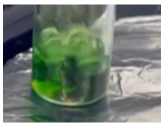 t = 40 sec 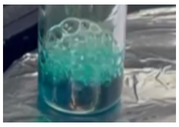

Figure S111: *Color changes observed during the reaction of oximes with t-BuONO.*

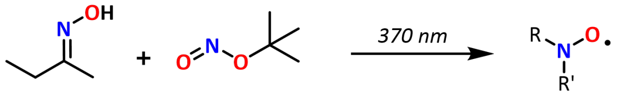

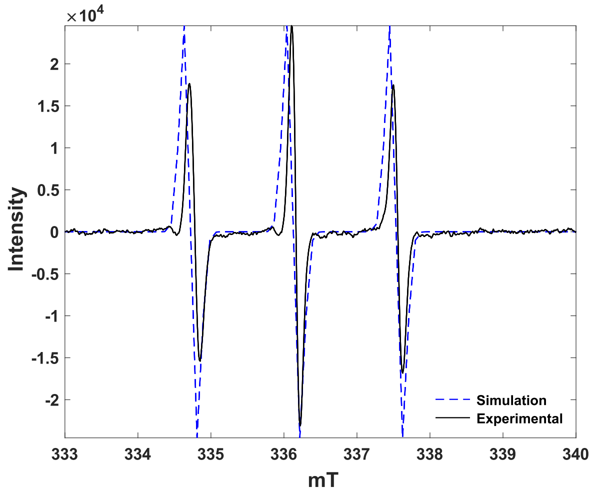


```
Sys1.Nucs = '14N';
a = 2.0192;
Sys1.g = [a a a];
A_N = [39.5 39.5 39.5]; % MHz
Sys1.A = A_N;
Sys1.lw = 0.2;
Sys1.weight = 2;
Exp.mwFreq = 9.5; % GHz
Exp.Range = [280 370]; % mT
```

Figure S112: *EPR trace measured after the reaction of 2-butanone oxime with t-BuONO (black trace) and the simulated spectrum with input (blue dotted trace).*

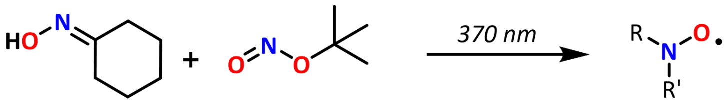

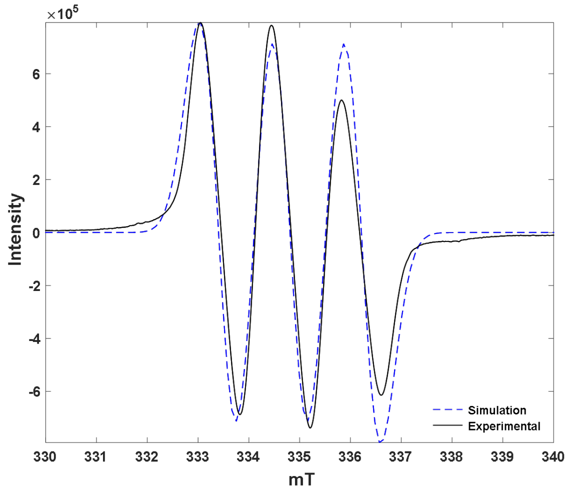


```
Sys1.Nucs = '14N';
a = 2.02719;
Sys1.g = [a a a];
A_N = [40 40 40]; % MHz
Sys1.A = A_N;
Sys1.lw = 0.9;
Sys1.weight = 2;
Exp.mwFreq = 9.5; % GHz
Exp.Range = [280 370]; % mT
```

Figure S113: *EPR trace measured after the reaction of cyclohexanone oxime with t-BuONO (black trace) and the simulated spectrum with input (blue dotted trace).*

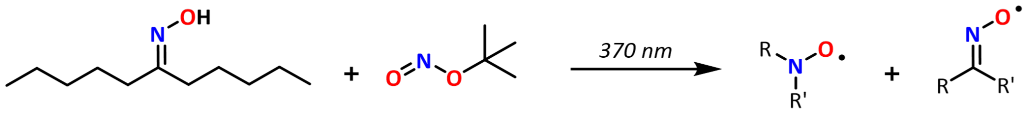

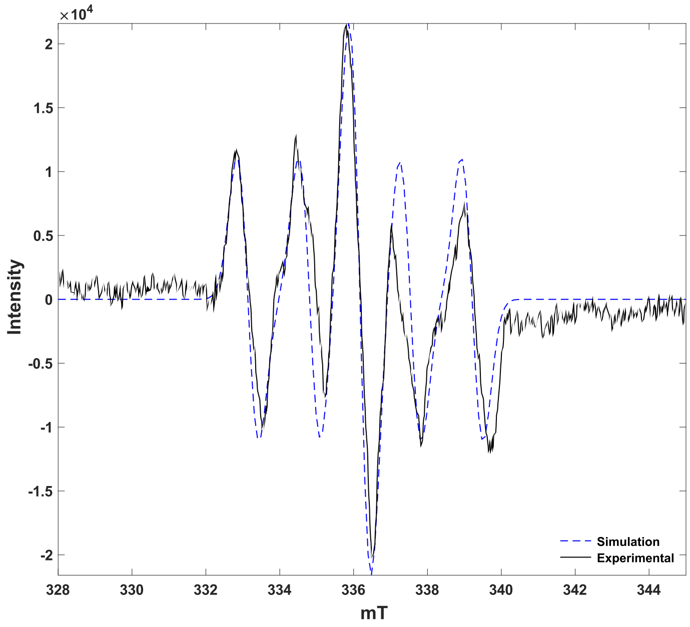


```
Sys1.Nucs = '14N';
Sys2.Nucs = '14N';

a = 2.019;
b = 2.019;

Sys1.g = [a a a];
Sys2.g = [b b b];

A_N = [39.5 39.5 39.5]; % MHz
A_M = [85 85 85]; % MHz

Sys1.A = A_N;
Sys2.A = A_M;

Sys1.lw = 0.7;
Sys2.lw = 0.7;

Sys1.weight = 0.5;
Sys2.weight = 0.5;

Exp.mwFreq = 9.5; % GHz

Exp.Range = [280 370]; % mT
```

Figure S114: *EPR trace measured after the reaction of 6-undecanone oxime with t-BuONO (black trace) and the simulated spectrum with input (blue dotted trace).*

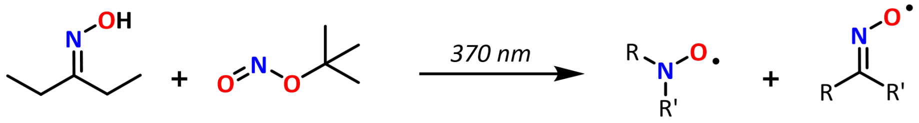

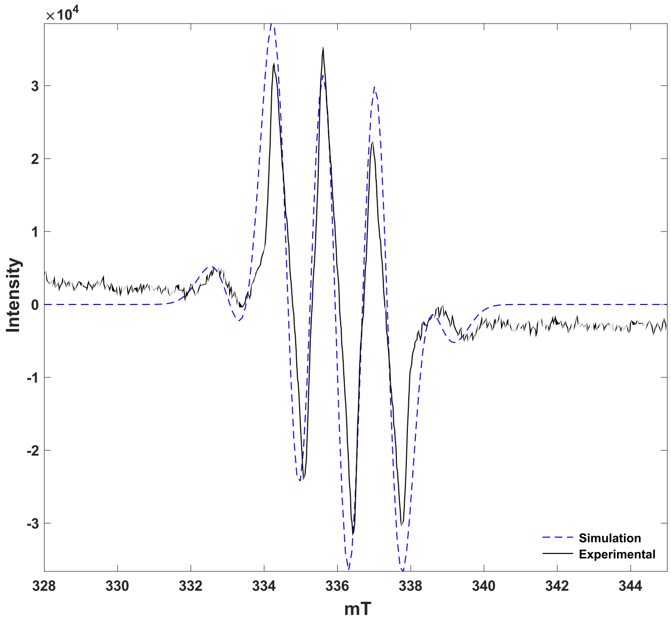


```
Sys1.Nucs = '14N';
Sys2.Nucs = '14N';

a = 2.0202;
b = 2.021;

Sys1.g = [a a a];
Sys2.g = [b b b];

A_N = [38 38 38]; % MHz
A_M = [80 80 80]; % MHz

Sys1.A = A_N;
Sys2.A = A_M;

Sys1.lw = 1.0;
Sys2.lw = 1.2;

Sys1.weight = 160;
Sys2.weight = 30;

Exp.mwFreq = 9.5; % GHz

Exp.Range = [280 370]; % mT
```

Figure S115: *EPR trace measured after the reaction of 3-pentanone oxime with t-BuONO (black trace) and the simulated spectrum with input (blue dotted trace).*

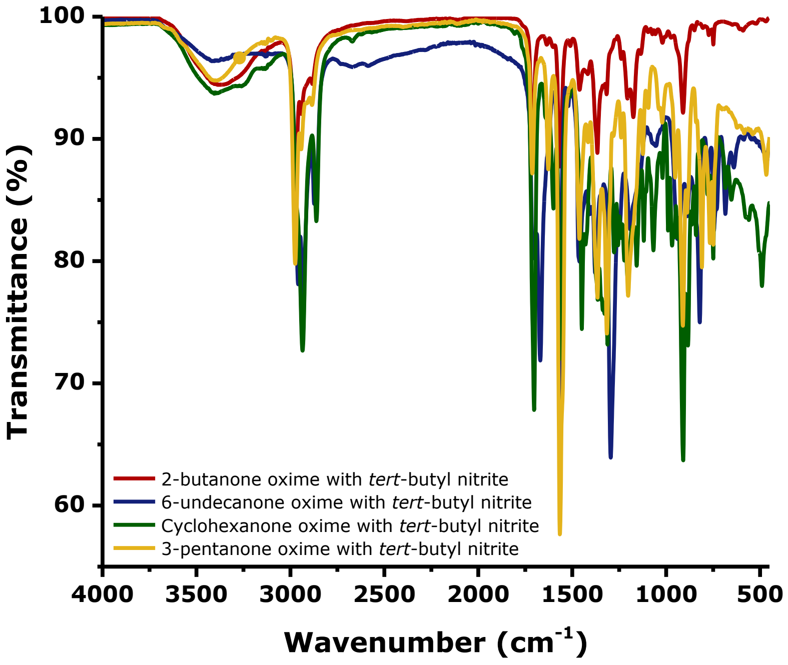

Figure S116: *FTIR (ATR) traces of the mixtures after the reaction of 3-pentanone oxime with t-BuONO.*

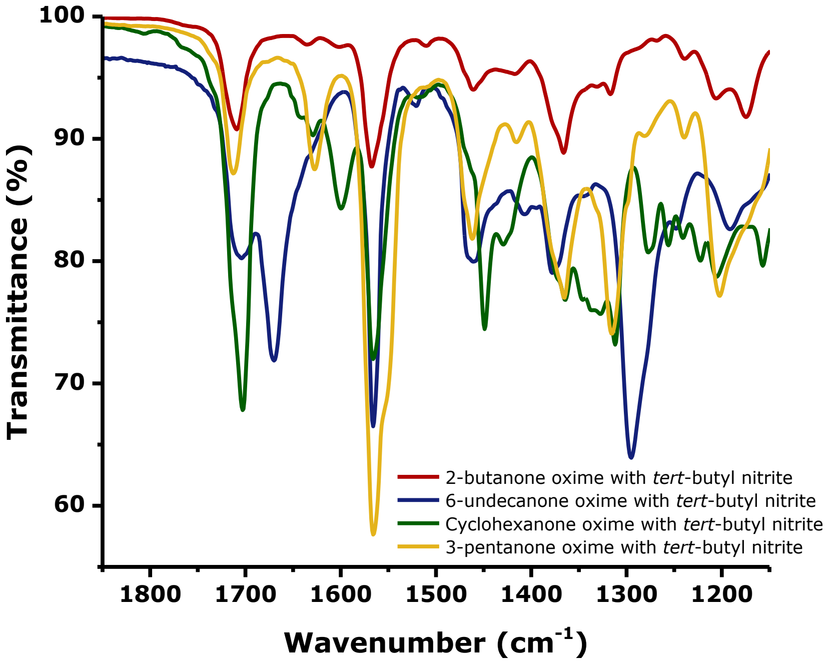

Figure S117: *FTIR (ATR) Zoom in of traces of the mixtures after the reaction of 3-pentanone oxime with t-BuONO.*

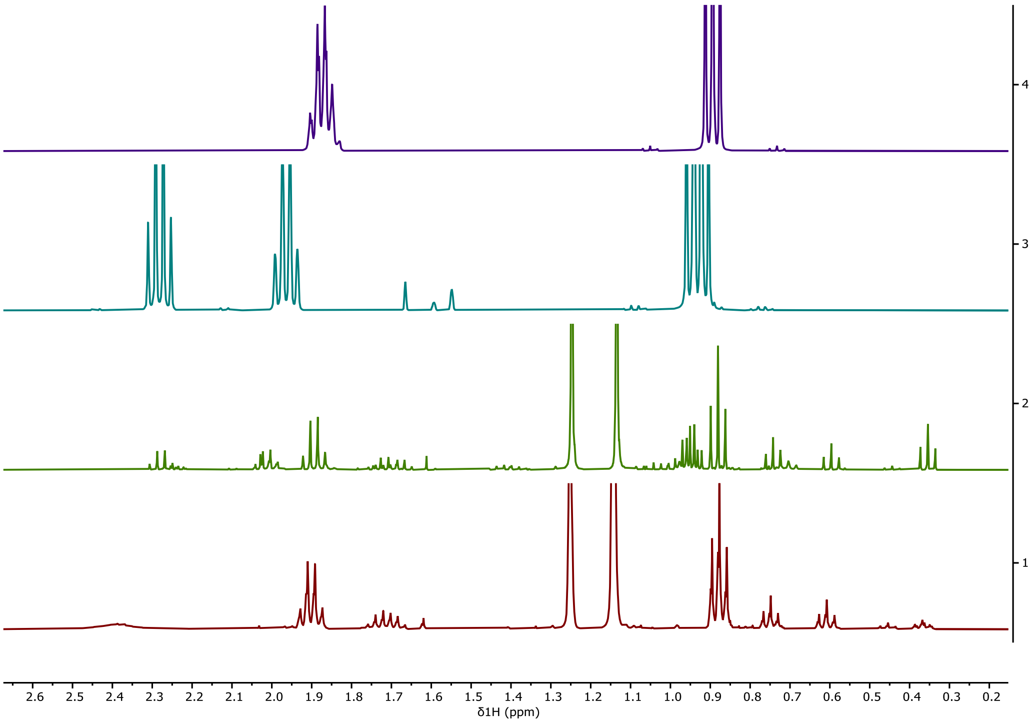

Figure S118: *^{1}H NMR spectra of 3-petanone (purple), 3-pentanone oxime (blue), 3-pentanone oxime measured directly after the addition of t-BuONO (green), 3-pentanone oxime measured after 1 day after the addition of t-BuONO (red).*

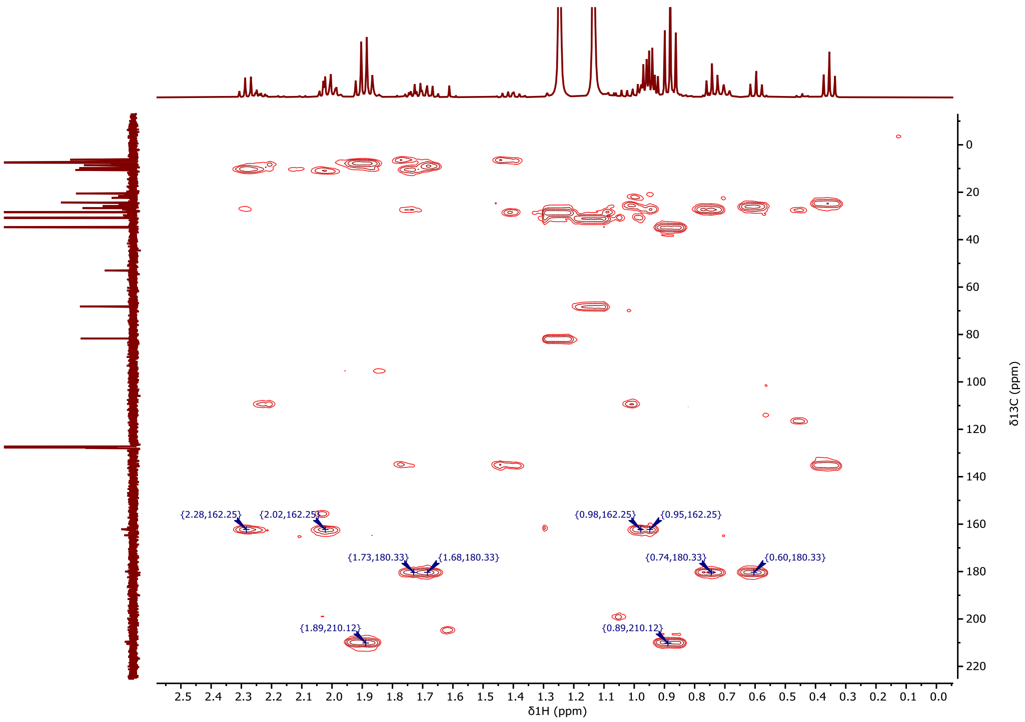

Figure S119: *^{1}H-^{13}C Heteronuclear Multiple Bond Correlation (HMBC) spectrum of 3-pentanone oxime measured directly after the addition of t-BuONO in $CDCl_3$ at 25 °C, with a zoom in on the coupling.*

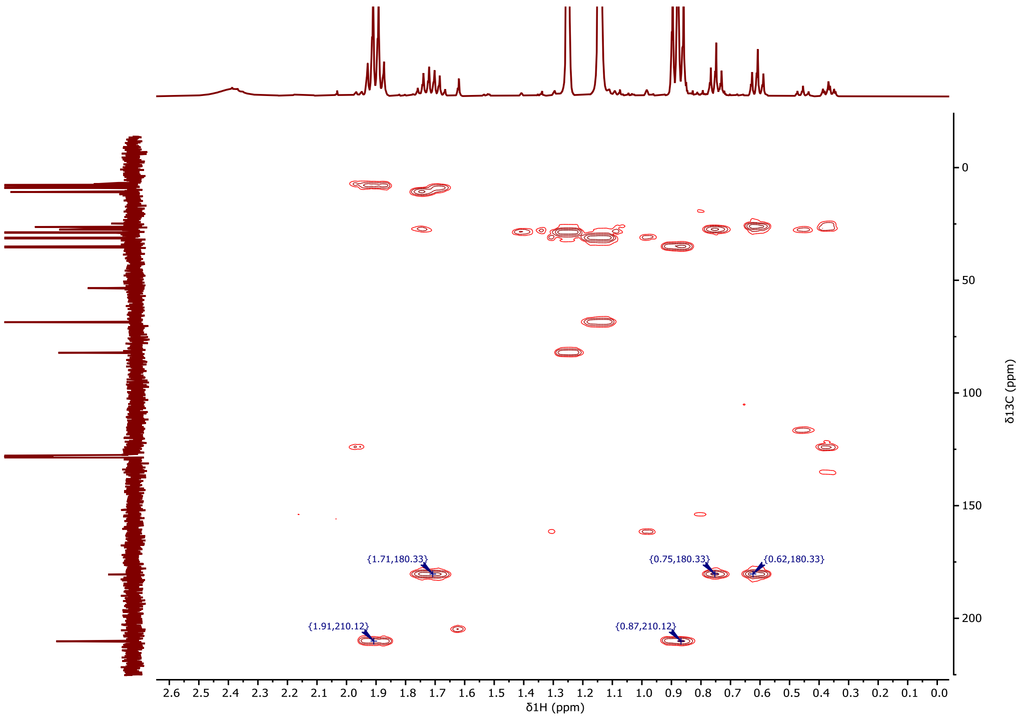

Figure S120: *^{1}H-[13]C Heteronuclear Multiple Bond Correlation (HMBC) spectrum of 3-pentanone oxime measured after 1 day after the addition of t-BuONO in $CDCl_3$ at 25 °C, with a zoom in on the coupling.*

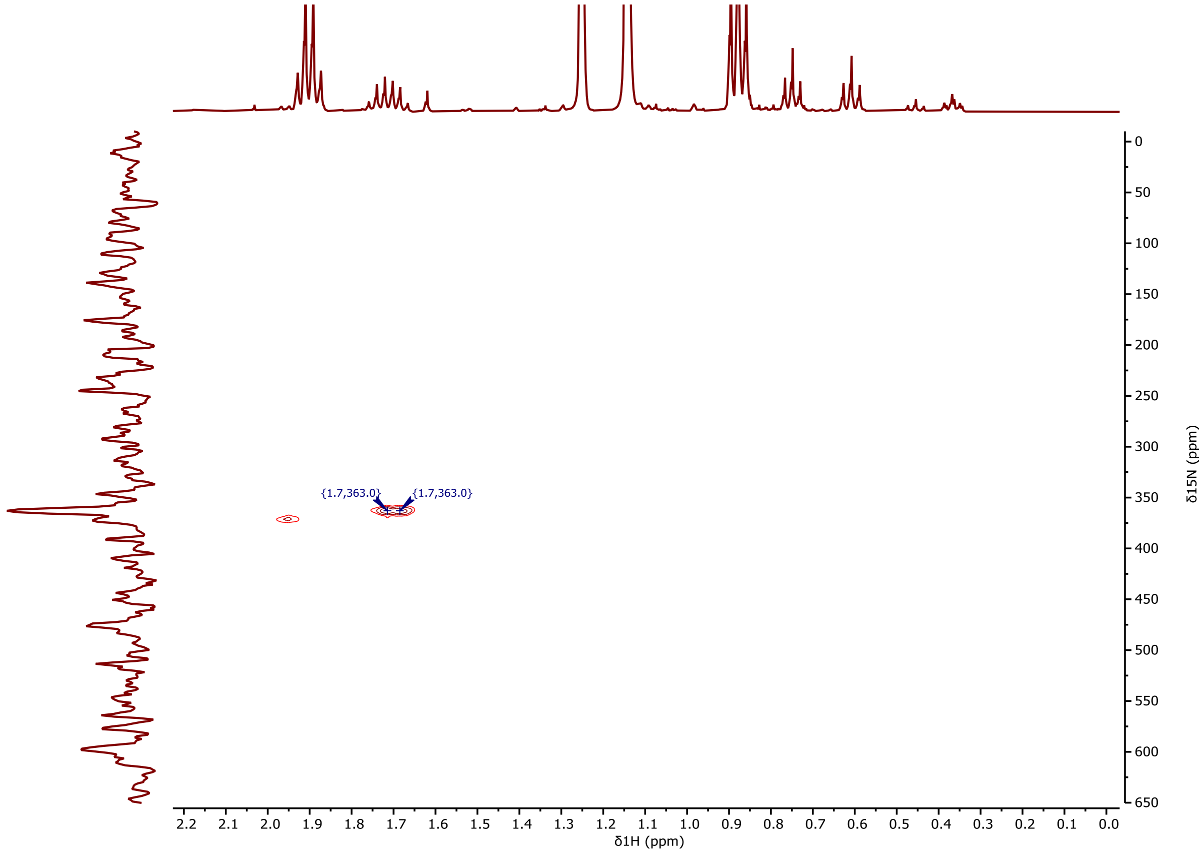

Figure S121: *^{1}H-[15]N Heteronuclear Multiple Bond Correlation (HMBC) spectrum of 3-pentanone oxime measured after 1 day after the addition of t-BuONO in $CDCl_3$ at 25 °C, with a zoom in on the coupling.*

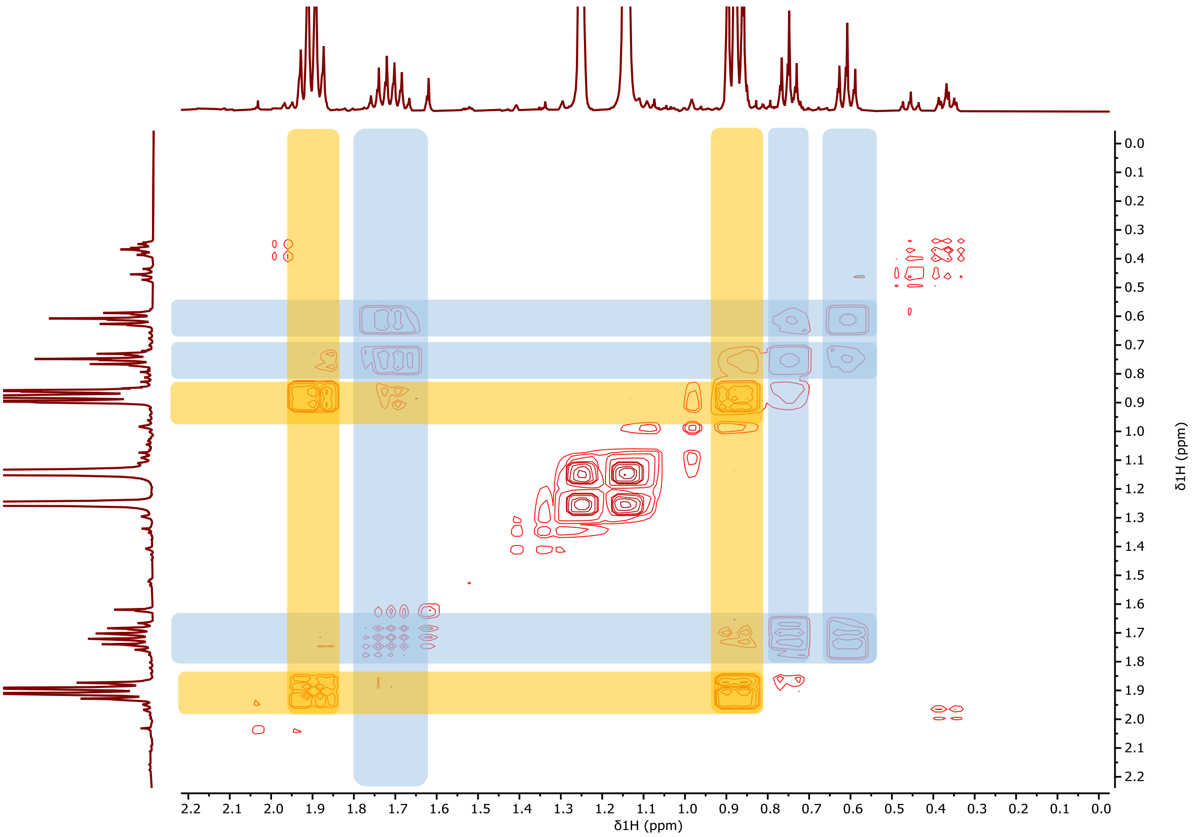

Figure S122: *^{1}H-^{1}H Correlated Spectroscopy (COSY) spectrum of 3-pentanone oxime measured after 1 day after the addition of t-BuONO in $CDCl_3$ at 25 °C.*

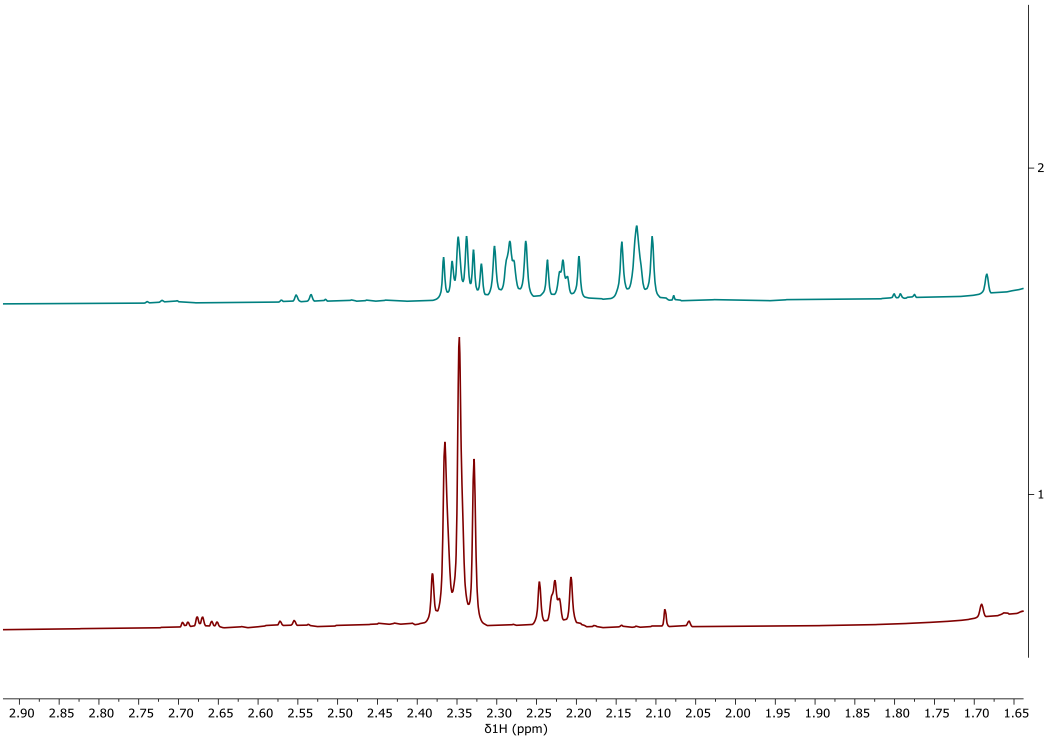

Figure S123: *^{1}H NMR spectra of 6-undecanone oxime after 20 min of heating at 105 °C, but no irradiation (blue), 6-undecanone oxime after 22 hours of heating at 105 °C, but no irradiation (red), in $CDCl_3$ at 25 °C.*

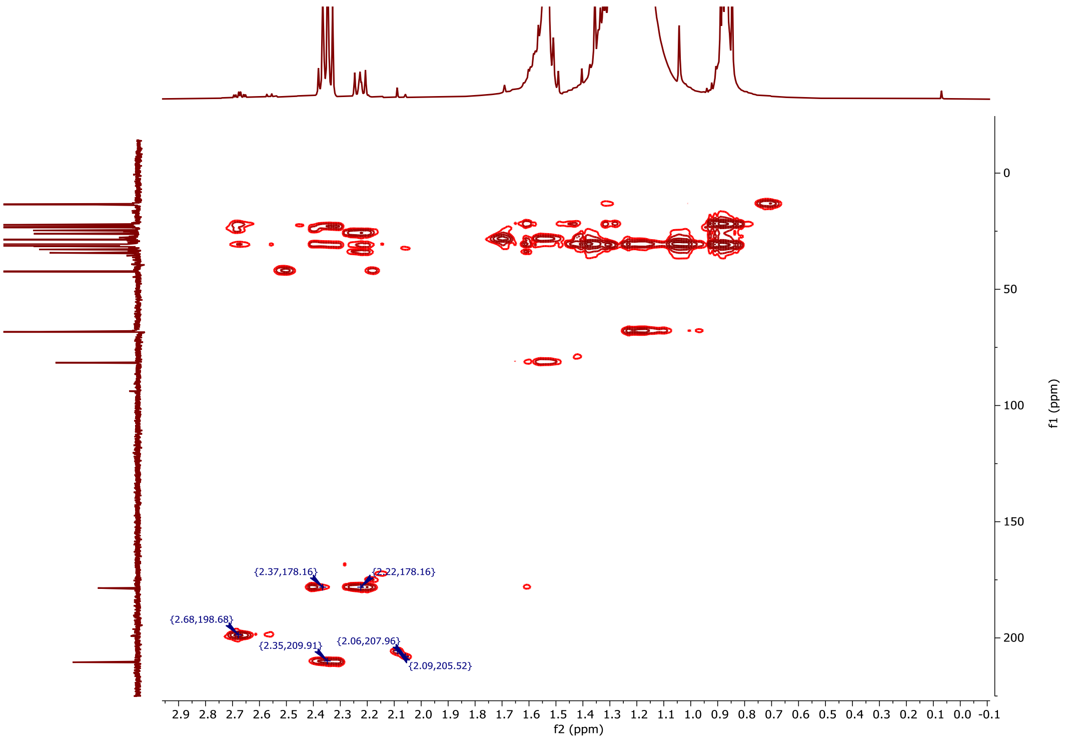

Figure S124: *^{1}H-^{13}C Heteronuclear Multiple Bond Correlation (HMBC) spectrum of 6-undecanone oxime after 22 hours of heating at 105 °C, but no irradiation, in $CDCl_3$ at 25 °C.*

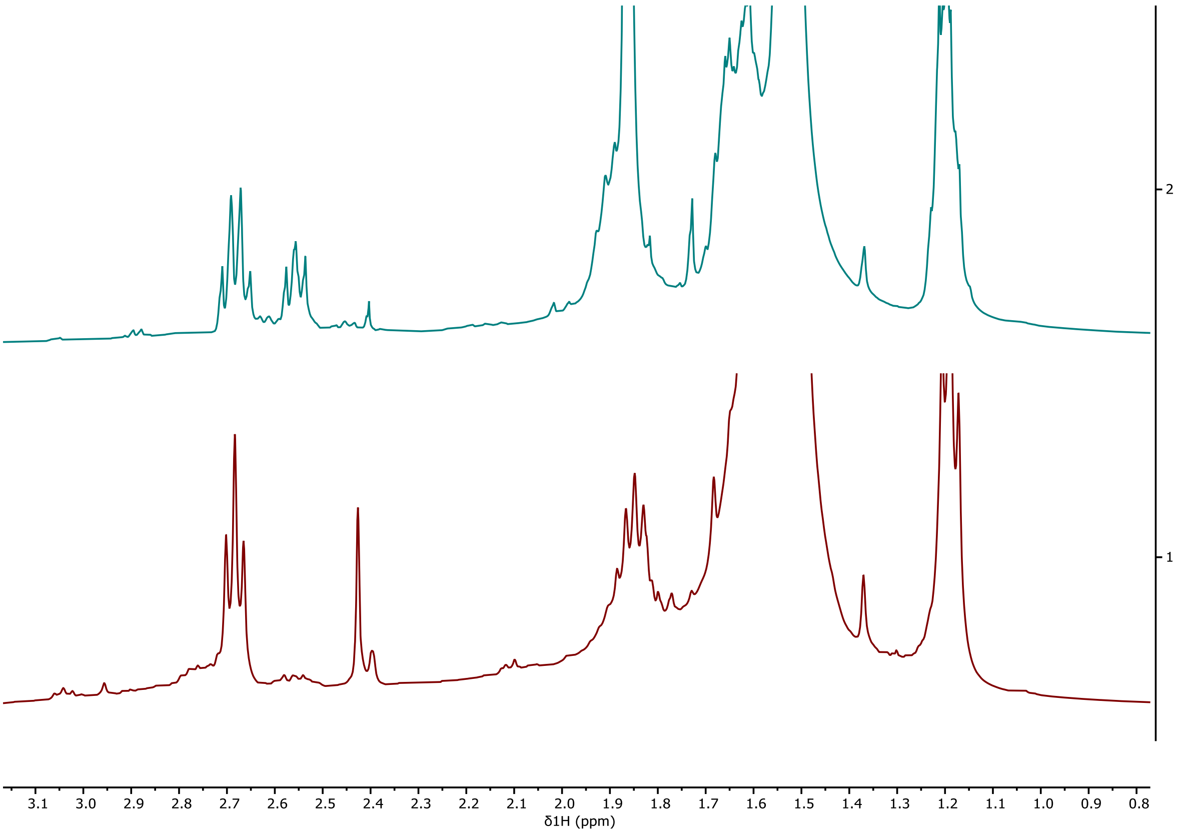

Figure S125: *^{1}H NMR spectra of 6-undecanone oxime after 30 min of irradiation at 370 nm, but no heating (blue), 6-undecanone oxime after 22 hours of irradiation at 370 nm, but no heating (red), in $CDCl_3$ at 25 °C.*

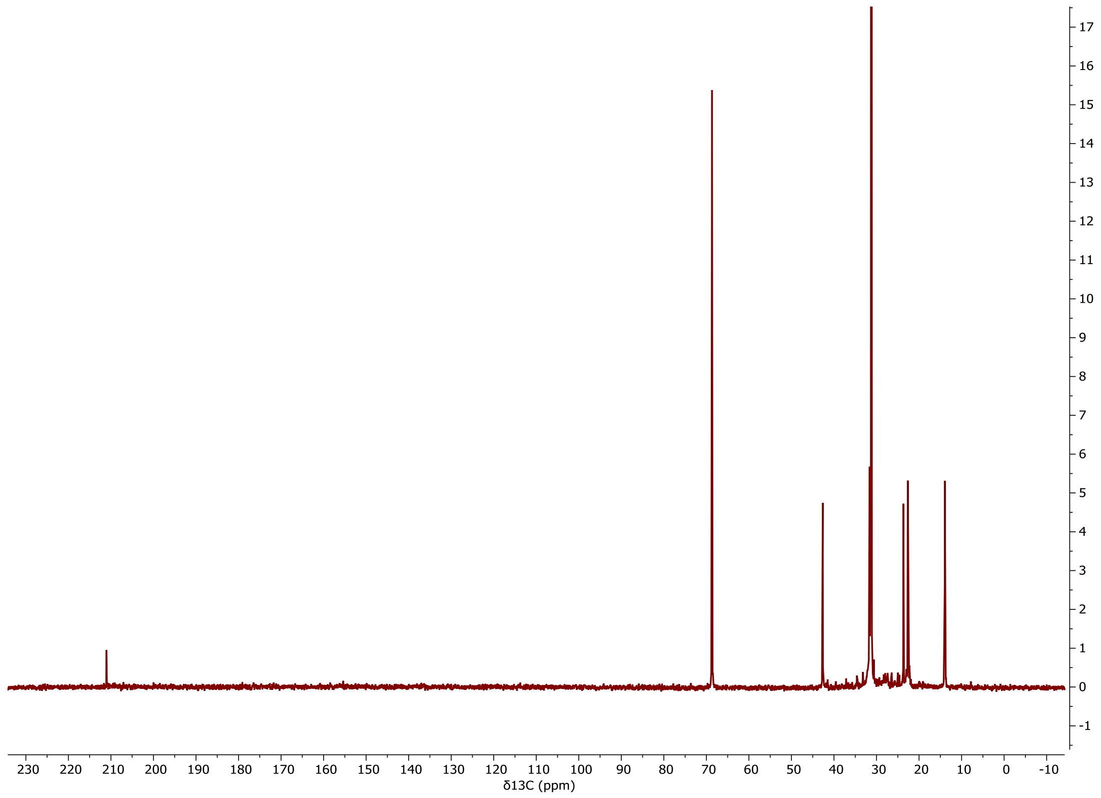

Figure S126: *^{13}C NMR spectra of 6-undecanone oxime after 22 hours of irradiation at 370 nm, but no heating (red), in $CDCl_3$ at 25 °C.*

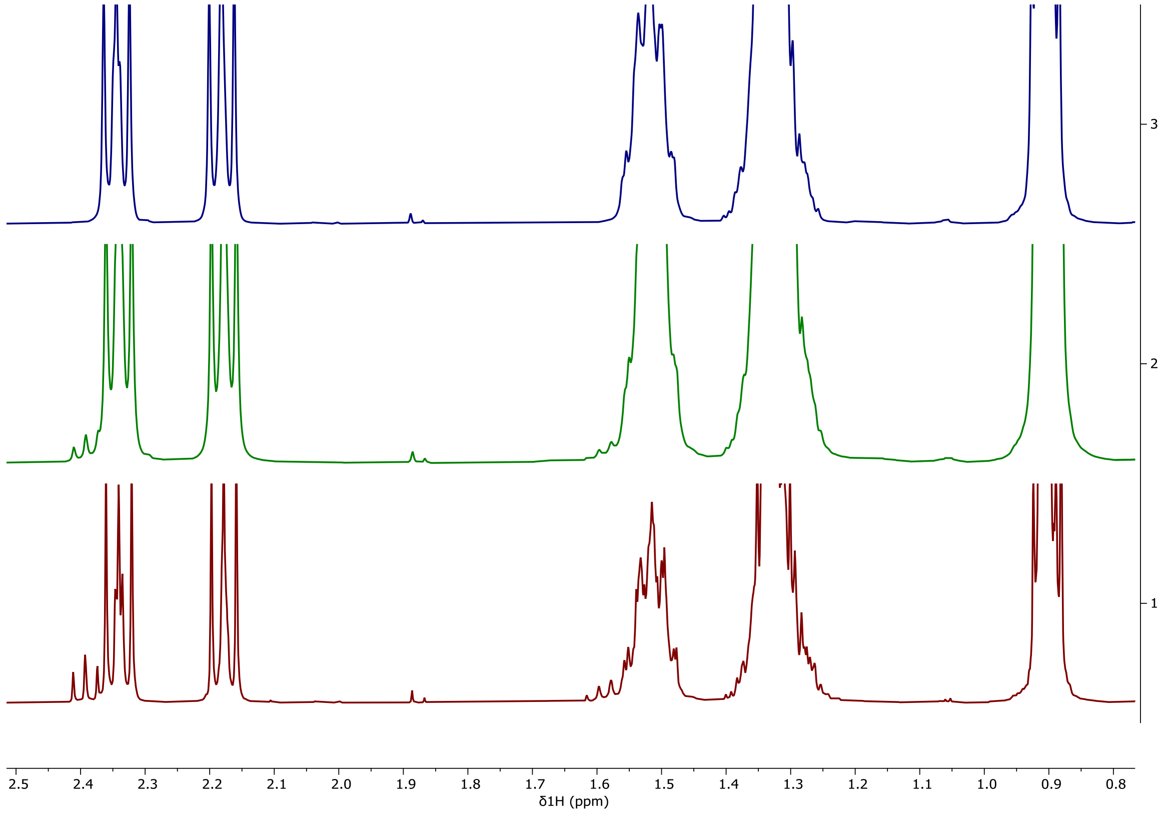

Figure S127: *^{1}H NMR spectra of 6-undecanone oxime (blue), heating and irradiation in the absence of t-BuONO and t-BuOH after 2 hours (green) and heating and irradiation in the absence of t-BuONO and t-BuOH after 15 hours (red), in $CDCl_3$ at 25 °C.*

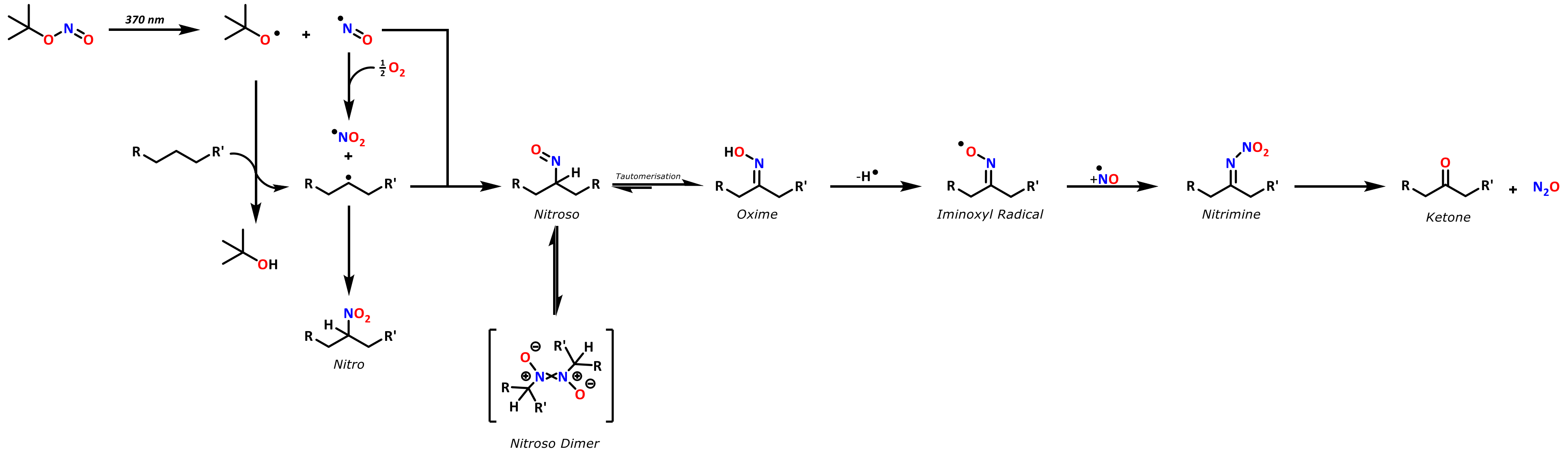

Scheme S1: *Proposed full reaction pathway for the functionalization of PE through the photochemical reaction with the formation of the oxime, ketone and nitro groups.*

References

(1) Chenal, T.; Olonde, X.; Pelletier, J. F.; Bujadoux, K.; Mortreux, A. Controlled Polyethylene Chain Growth on Magnesium Catalyzed by Lanthanidocene: A Living Transfer Polymerization for the Synthesis of Higher Dialkyl-Magnesium. *Polymer.* **2007**, *48* (7), 1844–1856.

(2) Chenal, T.; Visseaux, M. Combining Polyethylene CCG and Stereoregular Isoprene Polymerization: First Synthesis of Poly(Ethylene)-b-(Trans-Isoprene) by Neodymium Catalyzed Sequenced Copolymerization. *Macromolecules* **2012**, *45* (14), 5718–5727.

(3) Pelletier, J. F.; Mortreux, A.; Olonde, X.; Bujadoux, K. Synthesis of New Dialkylmagnesium Compounds by Living Transfer Ethylene Oligo- and Polymerization with Lanthanocene Catalysts. *Angew. Chemie (International Ed. English)* **1996**, *35* (16), 1854–1856.

(4) Junk, P. C.; Smith, M. K. Bis(M2-Chloro)-{bis(Diethylether)-Lithium}-Bis(η 5-Pentamethyl-Cyclopentadienyl)Samarium(III). *Appl. Organomet. Chem.* **2004**, *18* (5), 252.

(5) Rausch, M. D.; Morlarty, K. J.; Atwood, J. L.; Weeks, J. A.; Hunter, W. E.; Brittain, H. G. Synthetic, x-Ray Structural and Photoluminescence Studies on Pentamethylcyclopentadienyl Derivatives of Lanthanum, Cerium and Praseodymium. *Organometallics* **1986**, *5* (6), 1281–1283.

(6) Rothbaum, J. O.; Motta, A.; Kratish, Y.; Marks, T. J. Chemodivergent Organolanthanide-Catalyzed C-H α-Mono-Borylation of Pyridines. *J. Am. Chem. Soc.* **2022**, *144* (37), 17086–17096.

(7) Watson, P. L.; Whitney, J. F.; Harlow, R. L. (Pentamethylcyclopentadienyl)Ytterbium and -Lutetium Complexes by Metal Oxidation and Metathesis. *Inorg. Chem.* **1981**, *20* (10), 3271–3278.

(8) Kortman, G. D.; Orr, M. J.; Hull, K. L. Synthesis and Reactivity of Dioxazirconacyclohexenes: Development of a Zirconium-Oxo-Mediated Alkyne-Aldehyde Coupling Reaction. *Organometallics* **2015**, *34* (6), 1013–1016.

(9) Tilley, T. D.; Andersen, R. A. Pentamethylcyclopentadienyl Derivatives of the Trivalent Lanthanide Elements Neodymium, Samarium, and Ytterbium. *Inorg. Chem.* **1981**, *20* (10), 3267–3270.

(10) Schreurs, A. M. M.; Xian, X.; Kroon-Batenburg, L. M. J. EVAL15: A Diffraction Data Integration Method Based on Ab Initio Predicted Profiles. *J. Appl. Crystallogr.* **2010**, *43* (1), 70–82.

(11) Krause, L.; Herbst-Irmer, R.; Sheldrick, G. M.; Stalke, D. Comparison of Silver and Molybdenum Microfocus X-Ray Sources for Single-Crystal Structure Determination. *J. Appl. Crystallogr.* **2015**, *48* (1), 3–10.

(12) Sheldrick, G. M. Crystal Structure Refinement with SHELXL. *Acta Crystallogr.* **2015**, *C71*, 3–8.

(13) Spek, A. L. Structure Validation in Chemical Crystallography. *Acta Crystallogr.* **2009**, *D65*, 148–155.

(14) Tkachev, A. V.; Chibiryaev, A. M.; Denisov, A. Y.; Gatilov, Y. V. Reaction of Certain α,β-Unsaturated Terpenic Oximes with Sodium Nitrite in Acetic Acid: A Facile Synthesis of Allylic Nitro Compounds. *Tetrahedron* **1995**, *51* (6), 1789–1808.

(15) Wysocki, J.; Teles, J. H.; Dehn, R.; Trapp, O.; Sch, B.; Schaub, T. Photoinduced Direct Conversion of Cyclohexane into Cyclohexanone Oxime Using LEDs. *ChemPhotoChem* **2018**, *2* (22), 22–26.

(16) Mackor, A.; Veenland, J. U.; de Boer, T. J. C-Nitroso Compounds: Part. XI. Trans-Azodioxycyclohexane (Dimeric Nitrosocyclohexane) by Photochemical Nitrosation of Cyclohexane with Alkyl Nitrites. *Recl. des Trav. Chim. des Pays-Bas* **1969**, *88* (10), 1249–1262.
